# Global, regional, and national sex differences in the global burden of tuberculosis by HIV status, 1990–2019: results from the Global Burden of Disease Study 2019

**DOI:** 10.1016/S1473-3099(21)00449-7

**Published:** 2022-02

**Authors:** GBD 2019 Tuberculosis Collaborators, Jorge R Ledesma, Jianing Ma, Avina Vongpradith, Emilie R Maddison, Amanda Novotney, Molly H Biehl, Kate E LeGrand, Jennifer M Ross, Deepa Jahagirdar, Dana Bryazka, Rachel Feldman, Hassan Abolhassani, Akine Eshete Abosetugn, Eman Abu-Gharbieh, Oladimeji M Adebayo, Qorinah Estiningtyas Sakilah Adnani, Saira Afzal, Bright Opoku Ahinkorah, Sajjad Ahmad Ahmad, Sepideh Ahmadi, Tarik Ahmed Rashid, Yusra Ahmed Salih, Addis Aklilu, Chisom Joyqueenet Akunna, Hanadi Al Hamad, Fares Alahdab, Yosef Alemayehu, Kefyalew Addis Alene, Beriwan Abdulqadir Ali, Liaqat Ali, Vahid Alipour, Hesam Alizade, Rajaa M Al-Raddadi, Nelson Alvis-Guzman, Saeed Amini, Arianna Maever L Amit, Jason A Anderson, Sofia Androudi, Carl Abelardo T Antonio, Catherine M Antony, Razique Anwer, Jalal Arabloo, Asrat Arja, Mulusew A Asemahagn, Sachin R Atre, Gulrez Shah Azhar, Darshan B B, Zaheer-Ud-Din Babar, Atif Amin Baig, Maciej Banach, Hiba Jawdat Barqawi, Fabio Barra, Amadou Barrow, Sanjay Basu, Uzma Iqbal Belgaumi, Akshaya Srikanth Bhagavathula, Nikha Bhardwaj, Pankaj Bhardwaj, Natalia V Bhattacharjee, Krittika Bhattacharyya, Ali Bijani, Boris Bikbov, Archith Boloor, Nikolay Ivanovich Briko, Danilo Buonsenso, Sharath Burugina Nagaraja, Zahid A Butt, Austin Carter, Felix Carvalho, Jaykaran Charan, Souranshu Chatterjee, Soosanna Kumary Chattu, Vijay Kumar Chattu, Devasahayam J Christopher, Dinh-Toi Chu, Mareli M Claassens, Omid Dadras, Amare Belachew Dagnew, Xiaochen Dai, Lalit Dandona, Rakhi Dandona, Parnaz Daneshpajouhnejad, Aso Mohammad Darwesh, Deepak Dhamnetiya, Mostafa Dianatinasab, Daniel Diaz, Linh Phuong Doan, Sahar Eftekharzadeh, Muhammed Elhadi, Amir Emami, Shymaa Enany, Emerito Jose A Faraon, Farshad Farzadfar, Eduarda Fernandes, Lorenzo Ferro Desideri, Irina Filip, Florian Fischer, Masoud Foroutan, Tahvi D Frank, Alberto L Garcia-Basteiro, Christian Garcia-Calavaro, Tushar Garg, Biniyam Sahiledengle Geberemariyam, Keyghobad Ghadiri, Ahmad Ghashghaee, Mahaveer Golechha, Amador Goodridge, Bhawna Gupta, Sapna Gupta, Veer Bala Gupta, Vivek Kumar Gupta, Mohammad Rifat Haider, Samer Hamidi, Asif Hanif, Shafiul Haque, Harapan Harapan, Arief Hargono, Ahmed I Hasaballah, Abdiwahab Hashi, Shoaib Hassan, Hadi Hassankhani, Khezar Hayat, Kamal Hezam, Ramesh Holla, Mehdi Hosseinzadeh, Mihaela Hostiuc, Mowafa Househ, Rabia Hussain, Segun Emmanuel Ibitoye, Irena M Ilic, Milena D Ilic, Seyed Sina Naghibi Irvani, Nahlah Elkudssiah Ismail, Ramaiah Itumalla, Jalil Jaafari, Kathryn H Jacobsen, Vardhmaan Jain, Fatemeh Javanmardi, Sathish Kumar Jayapal, Shubha Jayaram, Ravi Prakash Jha, Jost B Jonas, Nitin Joseph, Farahnaz Joukar, Zubair Kabir, Ashwin Kamath, Tanuj Kanchan, Himal Kandel, Patrick DMC Katoto, Gbenga A Kayode, Parkes J Kendrick, Amene Abebe Kerbo, Himanshu Khajuria, Rovshan Khalilov, Khaled Khatab, Abdullah T Khoja, Jagdish Khubchandani, Min Seo Kim, Yun Jin Kim, Adnan Kisa, Sezer Kisa, Soewarta Kosen, Parvaiz A Koul, Sindhura Lakshmi Koulmane Laxminarayana, Ai Koyanagi, Kewal Krishan, Burcu Kucuk Bicer, Avinash Kumar, G Anil Kumar, Narinder Kumar, Nithin Kumar, Alexander Kwarteng, Hassan Mehmood Lak, Dharmesh Kumar Lal, Iván Landires, Savita Lasrado, Shaun Wen Huey Lee, Wei-Chen Lee, Christine Lin, Xuefeng Liu, Platon D Lopukhov, Rafael Lozano, Daiane Borges Machado, Shilpashree Madhava Kunjathur, Deepak Madi, Preetam Bhalchandra Mahajan, Azeem Majeed, Ahmad Azam Malik, Francisco Rogerlândio Martins-Melo, Saurabh Mehta, Ziad A Memish, Walter Mendoza, Ritesh G Menezes, Hayimro Edemealem Merie, Amanual Getnet Mersha, Mohamed Kamal Mesregah, Tomislav Mestrovic, Nour Mheidly Mheidly, Sanjeev Misra, Prasanna Mithra, Masoud Moghadaszadeh, Mokhtar Mohammadi, Abdollah Mohammadian-Hafshejani, Shafiu Mohammed, Mariam Molokhia, Mohammad Ali Moni, Ahmed Al Montasir, Catrin E Moore, Ahamarshan Jayaraman Nagarajan, Sanjeev Nair, Suma Nair, Atta Abbas Naqvi, Sreenivas Narasimha Swamy, Biswa Prakash Nayak, Javad Nazari, Sandhya Neupane Kandel, Trang Huyen Nguyen, Molly R Nixon, Chukwudi A Nnaji, Mpiko Ntsekhe, Virginia Nuñez-Samudio, Bogdan Oancea, Oluwakemi Ololade Odukoya, Andrew T Olagunju, Eyal Oren, Mahesh P A, Ramakrishnan Parthasarathi, Fatemeh Pashazadeh Kan, Sanjay M Pattanshetty, Rajan Paudel, Pintu Paul, Shrikant Pawar, Veincent Christian Filipino Pepito, Norberto Perico, Majid Pirestani, Roman V Polibin, Maarten J Postma, Akram Pourshams, Akila Prashant, Dimas Ria Angga Pribadi, Amir Radfar, Alireza Rafiei, Fakher Rahim, Vafa Rahimi-Movaghar, Mahfuzar Rahman, Mosiur Rahman, Amir Masoud Rahmani, Priyanga Ranasinghe, Chythra R Rao, David Laith Rawaf, Salman Rawaf, Marissa B Reitsma, Giuseppe Remuzzi, Andre M N Renzaho, Melese Abate Reta, Nima Rezaei, Omid Rezahosseini, Mohammad sadegh Rezai, Aziz Rezapour, Gholamreza Roshandel, Denis O Roshchin, Siamak Sabour, KM Saif-Ur-Rahman, Nasir Salam, Hossein Samadi Kafil, Mehrnoosh Samaei, Abdallah M Samy, Satish Saroshe, Benn Sartorius, Brijesh Sathian, Susan M Sawyer, Subramanian Senthilkumaran, Allen Seylani, Omid Shafaat, Masood Ali Shaikh, Kiomars Sharafi, Ranjitha S Shetty, Mika Shigematsu, Jae Il Shin, João Pedro Silva, Jitendra Kumar Singh, Smriti Sinha, Valentin Yurievich Skryabin, Anna Aleksandrovna Skryabina, Emma Elizabeth Spurlock, Chandrashekhar T Sreeramareddy, Paschalis Steiropoulos, Mu'awiyyah Babale Sufiyan, Takahiro Tabuchi, Eyayou Girma Tadesse, Zemenu Tamir, Elvis Enowbeyang Tarkang, Yohannes Tekalegn, Fisaha Haile Tesfay, Belay Tessema, Rekha Thapar, Imad I Tleyjeh, Ruoyan Tobe-Gai, Bach Xuan Tran, Berhan Tsegaye, Gebiyaw Wudie Tsegaye, Anayat Ullah, Chukwuma David Umeokonkwo, Sahel Valadan Tahbaz, Bay Vo, Giang Thu Vu, Yasir Waheed, Magdalene K Walters, Joanna L Whisnant, Mesfin Agachew Woldekidan, Befikadu Legesse Wubishet, Seyed Hossein Yahyazadeh Jabbari, Taklo Simeneh Yazie Yazie, Yigizie Yeshaw, Siyan Yi, Vahit Yiğit, Naohiro Yonemoto, Chuanhua Yu, Ismaeel Yunusa, Mikhail Sergeevich Zastrozhin, Anasthasia Zastrozhina, Zhi-Jiang Zhang, Alimuddin Zumla, Ali H Mokdad, Joshua A Salomon, Robert C Reiner Jr, Stephen S Lim, Mohsen Naghavi, Theo Vos, Simon I Hay, Christopher J L Murray, Hmwe Hmwe Kyu

## Abstract

**Background:**

Tuberculosis is a major contributor to the global burden of disease, causing more than a million deaths annually. Given an emphasis on equity in access to diagnosis and treatment of tuberculosis in global health targets, evaluations of differences in tuberculosis burden by sex are crucial. We aimed to assess the levels and trends of the global burden of tuberculosis, with an emphasis on investigating differences in sex by HIV status for 204 countries and territories from 1990 to 2019.

**Methods:**

We used a Bayesian hierarchical Cause of Death Ensemble model (CODEm) platform to analyse 21 505 site-years of vital registration data, 705 site-years of verbal autopsy data, 825 site-years of sample-based vital registration data, and 680 site-years of mortality surveillance data to estimate mortality due to tuberculosis among HIV-negative individuals. We used a population attributable fraction approach to estimate mortality related to HIV and tuberculosis coinfection. A compartmental meta-regression tool (DisMod-MR 2.1) was then used to synthesise all available data sources, including prevalence surveys, annual case notifications, population-based tuberculin surveys, and tuberculosis cause-specific mortality, to produce estimates of incidence, prevalence, and mortality that were internally consistent. We further estimated the fraction of tuberculosis mortality that is attributable to independent effects of risk factors, including smoking, alcohol use, and diabetes, for HIV-negative individuals. For individuals with HIV and tuberculosis coinfection, we assessed mortality attributable to HIV risk factors including unsafe sex, intimate partner violence (only estimated among females), and injection drug use. We present 95% uncertainty intervals for all estimates.

**Findings:**

Globally, in 2019, among HIV-negative individuals, there were 1·18 million (95% uncertainty interval 1·08–1·29) deaths due to tuberculosis and 8·50 million (7·45–9·73) incident cases of tuberculosis. Among HIV-positive individuals, there were 217 000 (153 000–279 000) deaths due to tuberculosis and 1·15 million (1·01–1·32) incident cases in 2019. More deaths and incident cases occurred in males than in females among HIV-negative individuals globally in 2019, with 342 000 (234 000–425 000) more deaths and 1·01 million (0·82–1·23) more incident cases in males than in females. Among HIV-positive individuals, 6250 (1820–11 400) more deaths and 81 100 (63 300–100 000) more incident cases occurred among females than among males in 2019. Age-standardised mortality rates among HIV-negative males were more than two times greater in 105 countries and age-standardised incidence rates were more than 1·5 times greater in 74 countries than among HIV-negative females in 2019. The fraction of global tuberculosis deaths among HIV-negative individuals attributable to alcohol use, smoking, and diabetes was 4·27 (3·69–5·02), 6·17 (5·48–7·02), and 1·17 (1·07–1·28) times higher, respectively, among males than among females in 2019. Among individuals with HIV and tuberculosis coinfection, the fraction of mortality attributable to injection drug use was 2·23 (2·03–2·44) times greater among males than females, whereas the fraction due to unsafe sex was 1·06 (1·05–1·08) times greater among females than males.

**Interpretation:**

As countries refine national tuberculosis programmes and strategies to end the tuberculosis epidemic, the excess burden experienced by males is important. Interventions are needed to actively communicate, especially to men, the importance of early diagnosis and treatment. These interventions should occur in parallel with efforts to minimise excess HIV burden among women in the highest HIV burden countries that are contributing to excess HIV and tuberculosis coinfection burden for females. Placing a focus on tuberculosis burden among HIV-negative males and HIV and tuberculosis coinfection among females might help to diminish the overall burden of tuberculosis. This strategy will be crucial in reaching both equity and burden targets outlined by global health milestones.

**Funding:**

Bill & Melinda Gates Foundation.

## Introduction

Tuberculosis is an important contributor to morbidity and mortality globally.^1^ Despite being preventable and treatable, tuberculosis is a leading cause of death from a single infectious agent.^2^, ^3^ The Sustainable Development Goals (SDGs) and the WHO End TB Strategy have defined targets for ending the tuberculosis epidemic that aim to reduce tuberculosis mortality by 95% and incidence by 90% by 2035.^4^, ^5^ An understanding of the trends in tuberculosis burden is crucial to evaluate progress towards ending the epidemic and informing policy and programmes.

**Research in context**

**Evidence before this study**

Globally, tuberculosis is a leading cause of death from a single infectious agent, contributing more than a million deaths annually. The global sex-specific burden of tuberculosis has been estimated by various groups, including the WHO Global Tuberculosis Programme and the Global Burden of Diseases, Injuries, and Risk Factors Study (GBD). However, differences in the levels and trends of the global burden of tuberculosis by HIV status, along with the contribution of modifiable risk factors by sex, have not been extensively characterised. We searched PubMed with the terms “tuberculosis” AND (“burden” OR “estimates”) AND (“sex” OR “gender”) AND (“differences” OR “discrepancies” OR “disparities”), with no language restrictions, for publications up to Dec 17, 2020. Our search identified ten studies that reported population-based tuberculosis burden estimates and, of these studies, two closely examined sex differences. One of these studies quantified sex differences in tuberculosis prevalence surveys from 2001 to 2014 for selected countries. Neither of these studies systematically examined sex differences in tuberculosis mortality and incidence, along with the contribution of modifiable risk factors, over time.

**Added value of this study**

This iteration of GBD provides tuberculosis mortality and morbidity estimates for nine new small or island countries and territories (Cook Islands, Monaco, San Marino, Nauru, Niue, Palau, Saint Kitts and Nevis, Tokelau, and Tuvalu). It also includes many new data sources and a multitude of advances in tuberculosis modelling methods. Moreover, we report for the first time how sex differences vary by sociodemographic development and the burden of HIV and tuberculosis coinfection attributable to potentially modifiable HIV risk factors (unsafe sex, intimate partner violence, and injection drug use). We also report a detailed assessment of sex differences across different measures of tuberculosis burden disaggregated by HIV status to enhance understanding of excess tuberculosis burden by sex globally. Finally, we report for the first time age-standardised, risk-deleted mortality rates along with the male-to-female ratio of risk-deleted mortality rates. Our data confirm findings from previous studies that the burden of tuberculosis is greater in males than in females. We found that males experienced substantially greater tuberculosis burden globally, with many countries estimated to have 50% higher incidence and 100% higher mortality rates for males than for females among HIV-negative individuals. Across all sociodemographic development levels, tuberculosis mortality rates were consistently greater among HIV-negative males than HIV-negative females. Among individuals with HIV and tuberculosis coinfection, mortality was higher among females than males, with unsafe sex and intimate partner violence being large contributors to the differences in the countries with the highest HIV burden.

**Implications of all the available evidence**

Smoking, alcohol consumption, and timely access to tuberculosis services represent challenges and opportunities for reducing the excess burden of tuberculosis among men. Developing strategies to prevent tuberculosis among males, while also reducing the burden of HIV and tuberculosis coinfection, especially among females, will be crucial in achieving equity targets outlined by the WHO End TB Strategy.

Despite an emphasis on equity in access to tuberculosis diagnosis and treatment by the WHO End TB Strategy, differences in tuberculosis burden by sex have received little attention. The available evidence indicates that men are less likely to seek or have access to tuberculosis care than women.^6^, ^7^, ^8^ A systematic review and meta-analysis of tuberculosis prevalence surveys in 28 countries found that males have 2·21 times higher prevalence of bacteriologically confirmed tuberculosis than females.^6^ For global case notifications, studies have estimated that males have a 50–70% higher incidence rate than females in selected low-income and middle-income countries.^6^, ^9^ Although previous Global Burden of Diseases, Injuries, and Risk Factors Study (GBD)^2^, ^10^ and WHO^3^ tuberculosis modelling efforts have presented sex-specific tuberculosis burden estimates, there has yet to be a systematic comparable quantification of these sex differences by HIV status, along with the contribution of modifiable risk factors. Few global sex differences are observed in age-adjusted prevalence of latent tuberculosis infection;^11^ this finding is a possible indicator that men have heightened exposure to risk factors for the development of active tuberculosis that might partially explain the sex differences. One study found that cigarette consumption explains 33% of the variation in the sex ratio of tuberculosis notifications.^12^ Although indicative, other important risk factors for tuberculosis, such as diabetes, alcohol consumption, and HIV infection, have yet to be considered in analyses of sex differences at the global level.

For GBD 2019, we assess the levels and trends of the global burden of tuberculosis, with an emphasis on investigating differences by sex. We focus on reporting tuberculosis mortality and incidence by HIV status and sex from 1990 to 2019, for 204 countries and territories. We also analyse how sex differences vary by the Socio-demographic Index (SDI),^13^ a composite indicator based on income, education, and fertility. Finally, we estimate the percentage of tuberculosis deaths, among HIV-negative individuals, attributable to the independent effects of risk factors including smoking, alcohol consumption, and diabetes. We also examine the proportion of deaths among people with HIV and tuberculosis coinfection that are attributable to HIV risk factors, including unsafe sex, intimate partner violence, and injection drug use.

## Methods

### Overview

Detailed methods for GBD and on tuberculosis estimation in GBD have been previously published.^1^, ^2^, ^10^ Here, we provide a brief description of the methods and estimation strategy for tuberculosis and risk factors. Detailed descriptions for each step of the estimation process with flow charts are provided in the appendix (pp 5–6). In compliance with the Guidelines for Accurate and Transparent Health Estimates Reporting, input data sources and code for each step of the estimation process are available on the Global Health Data Exchange. This manuscript was produced as part of the GBD Collaborator Network and in accordance with the GBD protocol.

### Tuberculosis mortality

The GBD Cause of Death database collates all available data from vital registration systems, surveillance systems, and verbal autopsies. For modelling mortality due to tuberculosis among HIV-negative individuals, we included 21 505 site-years of vital registration data, 705 site-years of verbal autopsy data, 825 site-years of sample-based vital registration data, and 680 site-years of mortality surveillance data. We processed raw data to reconcile discrepancies in coding schemes, redistribute garbage codes (eg, ill-defined codes and intermediate causes) to underlying causes of death, disaggregate data by age and sex, and adjust for misclassified HIV deaths.^1^

We modelled tuberculosis mortality among HIV-negative individuals using the Cause of Death Ensemble model (CODEm) framework with separate models by sex.^1^, ^14^ CODEm produces a wide range of submodels with different functional forms for the outcome variable (mortality rate or cause fraction), with varying combinations of predictive covariates (eg, alcohol consumption, diabetes, smoking prevalence, and Healthcare Access and Quality [HAQ] Index^15^). A full list of covariates used in the CODEm framework can be found in the appendix (p 13). The ensemble of models was then selected on the basis of CODEm performance on out-of-sample predictive validity tests. Tuberculosis mortality among HIV-positive individuals was estimated using a population attributable fraction approach consistent with previous GBD cycles.^2^, ^11^

### Tuberculosis morbidity

Consistent with previous GBD iterations,^2^, ^11^ we used a Bayesian meta-regression modelling tool, DisMod-MR 2.1^1^ to simultaneously model tuberculosis incidence, prevalence, and cause-specific mortality. Details on case definitions and how these data are processed are in the appendix (pp 3–4, 20–22).

We made several improvements to our modelling approach in GBD 2019. First, we standardised our approach to adjust prevalence data when the case definition was smear-positive tuberculosis rather than bacteriologically positive tuberculosis, using all available prevalence surveys that provided comparisons. We used the same approach to compute an adjustment factor to recalibrate studies that used symptoms only as a screening method compared with studies using both symptoms and chest x-ray during screening (appendix pp 21–22). Second, we used a novel Bayesian meta-regression method^16^ using age-sex-specific mortality-to-incidence ratios (logit transformed) from locations with high-quality data ratings on cause of death data as input. In our meta-regression analysis, we used the HAQ Index^15^ as the primary covariate, with GBD super-region fixed effects. After model calibration, we predicted age-sex-specific mortality-to-incidence ratios as a function of the HAQ Index for all locations and years similar to the previous GBD iteration.^11^ Third, we used tuberculosis duration and excess mortality rate data as priors in our morbidity modelling strategy to help create coherent estimates in modelling (appendix pp 22–23).

We used age-sex-specific case notifications as input data for locations with high-quality data ratings on causes of death data,^1^ predicted mortality-to-incidence ratio-based incidence for locations with low-quality data ratings for causes of death data, prevalence survey data where available, and estimates of excess mortality rate, remission, and cause-specific mortality as input to DisMod-MR 2.1^17^ to generate estimates that are consistent with each other. Sex-specific results were generated in DisMod-MR 2.1 by using sex-specific inputs and incorporating a covariate for sex. As in previous GBD iterations, we applied the proportion of cases of HIV and tuberculosis coinfection among all cases of tuberculosis to incidence and prevalence to distinguish between HIV and tuberculosis coinfection and tuberculosis without HIV.^4^, ^11^

### Risk factors

Methods for risk factor attribution to tuberculosis disaggregated by HIV status have previously been published in detail.^10^, ^18^ Briefly, we included risk–outcome pairs with convincing or probable evidence to compute the proportion of disease burden attributable to risk factors by estimating the relative risk of the outcome as a function of exposure values, estimating the global prevalence of exposures, and determining the theoretical minimum risk exposure level (TMREL) using all available global data sources (appendix pp 138–170). Next, estimates of attributable mortality were computed by multiplying the number of tuberculosis deaths by the population attributable fraction (PAF) for the risk–outcome pair for a given age, sex, location, and year. The PAF is the fraction of tuberculosis deaths that would be reduced if the exposure to the risk factor was at the TMREL. In addition to PAFs, we computed risk-deleted mortality rates, defined as the death rate that would had been observed if the risk factors of interest were set to their TMREL.^18^ For tuberculosis among HIV-negative individuals, we examined the fractions of deaths attributable to alcohol consumption, smoking, and diabetes. For HIV and tuberculosis coinfection, we focused on the fractions of deaths attributable to HIV risk factors including unsafe sex, intimate partner violence (only estimated among females), and injection drug use.

### SDI and data presentation

The SDI is a composite indicator, ranging from 0 to 1, of a country's total fertility rate for individuals younger than 25 years, average years of schooling, and lag-distributed income per capita.^1^ The index was categorised by quintiles using country-level estimates of the SDI in 2019. To investigate differences by sex, we present male-to-female (sex) ratios as the quotient of tuberculosis burden in males divided by tuberculosis burden in females. These ratios were computed for tuberculosis mortality rates, incidence rates, and PAFs. We use the GBD world population age standard to derive age-standardised rates for mortality and incidence. We present 95% uncertainty intervals (UIs) for every tuberculosis estimate based on the 2·5th and 97·5th percentiles of the posterior distributions that are carried over from each step in analyses.

### Role of the funding source

The funder of the study had no role in study design, data collection, data analysis, data interpretation, or writing of the report.

## Results

Globally, in 2019, among HIV-negative individuals, we estimated that there were 8·50 million (95% UI 7·45–9·73) tuberculosis incident cases and 1·18 million (1·08–1·29) deaths due to tuberculosis. In 1990, these figures were 8·76 million (7·59–10·06) tuberculosis incident cases and 1·78 million (1·63–1·92) deaths. Among HIV-positive individuals, we estimated that there were 1·15 million (1·01–1·32) tuberculosis incident cases and 217 000 (153 000–279 000) deaths due to tuberculosis in 2019 (appendix pp 62–77). The corresponding figures in 1990 for HIV-positive individuals were 507 000 (449 000–571 000) cases and 119 000 (89 700–159 000) deaths. Overall, HIV and tuberculosis coinfection constituted 12·0% (11·3–12·4) of the global 9·65 million (8·48–11·03) incident cases of tuberculosis and 15·5% (9·6–21·3) of the global 1·40 million (1·28–1·53) deaths due to tuberculosis in 2019.

Globally, in 2019, more incident cases and deaths occurred in males than in females among HIV-negative individuals, with 1·01 million (95% UI 0·82–1·23) more incident cases and 342 000 (234 000–425 000) more deaths among males than females (table). Among HIV-positive individuals, however, 81 100 (63 300–100 000) more incident cases and 6250 (1820–11 400) more deaths occurred among females than among males (appendix p 62). Globally, 56·0% (54·4–57·5) of incident cases and 64·5% (59·8–67·8) of deaths occurred among males in 2019.

**Table:** Tuberculosis incident cases and deaths and age-standardised rates of tuberculosis incidence and mortality per 100 000 population among HIV-negative males and females by GBD regions and SDI quintiles, 2019

	**Incidence**				**Mortality**			
	Female cases	Rate (per 100 000 females)	Male cases	Rate (per 100 000 males)	Female deaths	Rate (per 100 000 females)	Male deaths	Rate (per 100 000 males)
**Global**	**3 740 000 (3 270 000–4 310 000)**	**94·9 (83·0–109)**	**4 750 000 (4 160 000–5 440 000)**	**120 (105–136)**	**419 000 (366 000–502 000)**	**10·1 (8·81–12·1)**	**761 000 (688 000–836 000)**	**19·7 (17·9–21·6)**
Low SDI	938 000 (815 000–1 080 000)	196 (173–223)	964 000 (847 000–1 110 000)	228 (201–259)	135 000 (115 000–168 000)	43·4 (37·2–54·7)	234 000 (204 000–269 000)	77·5 (68·7–87·6)
Low-middle SDI	1 460 000 (1 260 000–1 700 000)	170 (148–196)	1 660 000 (1 420 000–1 920 000)	202 (175–233)	176 000 (144 000–220 000)	23·8 (19·5–29·8)	312 000 (271 000–353 000)	45·2 (39·2–51·1)
Middle SDI	1 020 000 (897 000–1 160 000)	83·3 (73·2–94·5)	1 340 000 (1 180 000–1 520 000)	107 (95·3–121)	86 700 (76 700–103 000)	7·06 (6·25–8·42)	168 000 (153 000–186 000)	14·7 (13·5–16·2)
High-middle SDI	265 000 (230 000–304 000)	33·8 (29·4–39·1)	487 000 (423 000–561 000)	58·5 (51·1–67·1)	15 000 (13 400–17 700)	1·53 (1·37–1·79)	38 300 (34 600–42 500)	4·33 (3·92–4·80)
High SDI	49 600 (43 100–56 900)	8·33 (7·20–9·62)	74 500 (65 100–85 600)	11·9 (10·4–13·8)	5450 (4380–6250)	0·470 (0·395–0·551)	8450 (7620–9180)	1·02 (0·918–1·11)
**Central Europe, eastern Europe, and central Asia**	**78 200 (66 300–92 800)**	**34·4 (29·1–41·0)**	**159 000 (133 000–191 000)**	**69·7 (58·3–83·3)**	**5210 (4610–5920)**	**1·95 (1·72–2·22)**	**15 700 (14 100–17 500)**	**6·40 (5·74–7·12)**
Central Asia	21 500 (18 500–24 900)	44·9 (38·8–52·1)	30 600 (26 200–35 800)	67·7 (58·4–78·2)	1910 (1630–2250)	4·09 (3·49–4·81)	4140 (3690–4700)	9·69 (8·67–10·9)
Central Europe	6710 (5790–7790)	9·67 (8·30–11·3)	13 400 (11 300–15 900)	18·8 (16·1–22·1)	519 (451–591)	0·495 (0·430–0·570)	1620 (1390–1850)	1·96 (1·70–2·24)
Eastern Europe	50 000 (41 400–60 500)	43·1 (35·8–52·2)	115 000 (94 400–141 000)	99·2 (81·9–120)	2780 (2290–3290)	1·90 (1·56–2·25)	9960 (8480–11 500)	7·80 (6·65–9·01)
**High income**	**39 600 (34 400–45 500)**	**6·10 (5·26–7·05)**	**58 000 (50 300–67 100)**	**8·95 (7·75–10·4)**	**5430 (4350–6140)**	**0·391 (0·329–0·434)**	**7750 (6990–8280)**	**0·825 (0·748–0·882)**
Australasia	933 (801–1100)	6·19 (5·30–7·34)	929 (808–1070)	6·04 (5·20–7·04)	47·7 (36·3–59·0)	0·166 (0·128–0·202)	64·8 (51·2–82·8)	0·280 (0·221–0·356)
High-income Asia Pacific	18 800 (16 100–21 900)	13·0 (11·1–15·1)	28 500 (24 300–33 600)	19·9 (17·1–23·3)	2830 (2130–3290)	0·797 (0·635–0·908)	4260 (3710–4660)	2·02 (1·79–2·22)
High-income North America	3930 (3360–4610)	1·88 (1·60–2·22)	5990 (5200–6960)	2·85 (2·46–3·32)	440 (393–480)	0·129 (0·117–0·139)	689 (639–725)	0·254 (0·235–0·267)
Southern Latin America	3660 (3180–4260)	10·4 (8·91–12·2)	4660 (4010–5460)	13·7 (11·8–16·0)	469 (411–534)	1·05 (0·930–1·19)	770 (726–817)	2·15 (2·03–2·29)
Western Europe	12 300 (10 600–14 300)	5·78 (4·88–6·83)	17 900 (15 400–20 900)	8·30 (6·98–9·86)	1640 (1350–1870)	0·266 (0·227–0·299)	1970 (1800–2130)	0·483 (0·445–0·519)
**Latin America and Caribbean**	**67 700 (58 200–78 400)**	**21·9 (18·9–25·3)**	**102 000 (87 100–119 000)**	**34·7 (29·9–40·3)**	**6280 (5360–7660)**	**2·01 (1·72–2·45)**	**11 800 (10 500–13 300)**	**4·25 (3·79–4·78)**
Andean Latin America	17 900 (15 300–20 900)	55·9 (47·7–65·2)	24 000 (20 500–28 100)	76·3 (65·5–88·7)	1610 (1270–2070)	5·36 (4·24–6·87)	2620 (2050–3280)	9·26 (7·26–11·5)
Caribbean	8410 (7200–9710)	35·3 (30·1–40·9)	7160 (6190–8280)	30·1 (26·0–34·8)	1010 (755–1510)	4·04 (3·02–6·07)	1300 (1050–1570)	5·49 (4·42–6·59)
Central Latin America	19 400 (16 800–22 300)	14·8 (12·8–16·9)	26 000 (22 400–30 100)	21·6 (18·7–24·9)	2040 (1650–2620)	1·60 (1·29–2·05)	3730 (3150–4410)	3·34 (2·82–3·94)
Tropical Latin America	21 900 (18 500–25 900)	18·0 (15·2–21·2)	44 800 (37 800–53 200)	38·4 (32·5–45·3)	1620 (1440–1810)	1·26 (1·12–1·41)	4110 (3880–4350)	3·64 (3·43–3·86)
**North Africa and Middle East**	**79 600 (68 000–94 200)**	**28·1 (24·1–32·9)**	**73 900 (63 300–86 300)**	**24·6 (21·4–28·4)**	**7470 (5800–10 800)**	**3·37 (2·66–5·09)**	**8030 (6510–9890)**	**3·50 (2·90–4·23)**
**South Asia**	**1 750 000 (1 500 000–2 040 000)**	**201 (174–232)**	**2 070 000 (1 770 000–2 410 000)**	**240 (206–278)**	**188 000 (152 000–239 000)**	**25·6 (20·7–32·8)**	**334 000 (280 000–393 000)**	**46·5 (39·2–54·5)**
**Southeast Asia, east Asia, and Oceania**	**707 000 (628 000–791 000)**	**62·3 (54·9–69·8)**	**1 180 000 (1 060 000–1 320 000)**	**99·5 (88·8–111)**	**67 600 (59 600–77 700)**	**5·30 (4·69–6·09)**	**133 000 (120 000–148 000)**	**11·8 (10·7–13·0)**
East Asia	260 000 (230 000–294 000)	31·7 (27·9–35·5)	503 000 (449 000–567 000)	57·4 (51·4–64·1)	11 400 (9270–14 300)	1·15 (0·945–1·43)	29 300 (24 000–35 200)	3·40 (2·84–4·01)
Oceania	6480 (5630–7440)	107 (95–121)	5840 (5180–6490)	102 (91·9–111)	679 (373–1030)	15·7 (8·75–23·6)	1190 (813–1620)	27·4 (18·9–37·0)
Southeast Asia	440 000 (390 000–495 000)	130 (115–145)	671 000 (593 000–753 000)	207 (185–230)	55 500 (48 200–64 400)	17·9 (15·6–20·7)	103 000 (90 000–117 000)	38·2 (33·9–42·9)
**Sub-Saharan Africa**	**1 020 000 (875 000–1 190 000)**	**209 (182–239)**	**1 110 000 (983 000–1 270 000)**	**276 (245–312)**	**139 000 (115 000–171 000)**	**47·5 (40·0–60·2)**	**251 000 (217 000–290 000)**	**94·3 (82·7–107)**
Central sub-Saharan Africa	169 000 (145 000–195 000)	289 (253–328)	155 000 (136 000–175 000)	325 (291–362)	27 800 (19 600–40 900)	75·3 (51·6–112)	41 400 (31 600–53 400)	136 (105–172)
Eastern sub-Saharan Africa	409 000 (347 000–481 000)	231 (202–266)	502 000 (437 000–579 000)	334 (295–380)	58 900 (47 800–76 500)	59·4 (48·8–77·9)	113 000 (92 200–133 000)	115 (94·6–135)
Southern sub-Saharan Africa	160 000 (130 000–193 000)	372 (307–447)	130 000 (113 000–148 000)	321 (285–360)	10 300 (8860–12 100)	29·2 (25·5–33·9)	24 900 (22 100–27 800)	88·2 (79·8–97·3)
Western sub-Saharan Africa	286 000 (247 000–330 000)	152 (134–174)	328 000 (288 000–379 000)	210 (184–240)	41 600 (29 700–56 600)	36·3 (25·0–53·4)	72 000 (59 000–86 100)	67·6 (56·1–80·2)

95% uncertainty intervals are shown in parentheses. GBD=Global Burden of Diseases, Injuries, and Risk Factors Study. SDI=Socio-demographic Index.

In HIV-negative people, the global age-standardised tuberculosis incidence rate per 100 000 population in 2019 was 120 (95% UI 105–136) among males and 94·9 (83·0–109) among females, and the global age-standardised tuberculosis mortality rate per 100 000 population was 19·7 (17·9–21·6) among males and 10·1 (8·81–12·1) among females (table). At the regional level, among HIV-negative individuals, age-standardised incidence rates were nearly two times higher in eastern Europe, tropical Latin America, and central Europe, and age-standardised mortality rates were more than three times higher in eastern Europe, central Europe, and southern sub-Saharan Africa, among males than among females.

Among HIV-positive individuals, the global age-standardised tuberculosis incidence rate per 100 000 population in 2019 was 13·2 (95% UI 11·6–15·0) among males and 15·5 (13·5–17·7) among females, and the global age-standardised tuberculosis mortality rate per 100 000 population was 2·61 (1·83–3·35) among males and 2·81 (1·98–3·60) among females (appendix pp 61–75). The regions where males with HIV had the largest relative excess burden compared with females with HIV were in high-income Asia Pacific, Australasia, and central Latin America for both incidence and mortality, but females had higher age-standardised rates for both measures in almost every GBD region in Africa.

**Figure 1:**
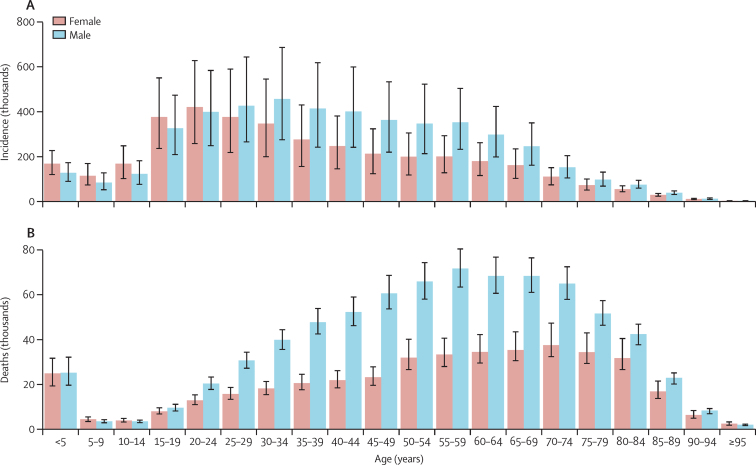
Global age-sex distribution of tuberculosis incident cases (A) and deaths (B) in HIV-negative individuals, 2019

The majority of incident cases (78·0% [95% UI 73·5–81·8]) and deaths (58·3% [56·8–59·8]) in 2019 were in individuals aged 15–64 years (figure 1). For tuberculosis incidence among HIV-negative individuals, females had slightly greater numbers of incident cases for age groups younger than 25 years, but more incident cases occurred in males throughout most adult age groups. The differences between sexes became smaller in the older age groups. For tuberculosis mortality in HIV-negative individuals, trends were similar to those observed for incident infections, but compared with females, males had more deaths starting at age 15–19 years (figure 1). Among HIV-positive people, females generally had more cases and deaths than males in the reproductive age groups, in which most cases and deaths occurred (appendix p 46).

**Figure 2:**
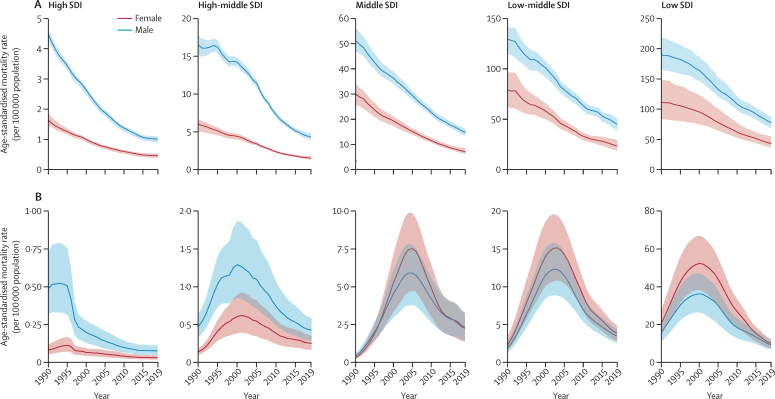
Temporal trends of age-standardised tuberculosis mortality rate per 100 000 population among HIV-negative individuals (A) and HIV-positive individuals (B) by SDI quintile and sex, 1990–2019 SDI=Socio-demographic Index.

Figure 2 shows trends in age-standardised tuberculosis mortality rate per 100 000 population among HIV-negative individuals and HIV-positive individuals by SDI quintile and sex. Across SDI quintiles, sharp declines in age-standardised tuberculosis mortality from 1990 to 2019 occurred in both males and females among HIV-negative individuals. Males consistently had higher mortality than females across the time period. Although the relative gap between males and females without HIV declined for the high SDI quintile, the gap remained the same or slightly increased in the remaining SDI quintiles (appendix p 41). For people with HIV and tuberculosis coinfection, there were sharp declines in age-standardised mortality after each SDI quintile's peak in tuberculosis mortality. In the high SDI and high-middle SDI quintiles, males with HIV and tuberculosis coinfection consistently had higher mortality than females. In the low and middle SDI quintiles, however, females with HIV and tuberculosis coinfection had higher mortality than males throughout most of the time series. The relative gap between females and males diminished substantially in the low and middle SDI quintiles after peaks in HIV and tuberculosis coinfection mortality (appendix p 41).

Among HIV-negative individuals, age-standardised tuberculosis incidence rates were more than 300 per 100 000 population in 21 countries for males and in ten countries for females (appendix pp 42, 48–61). Age-standardised tuberculosis mortality rates were more than 100 per 100 000 population in 19 countries for males and in three for females (appendix pp 43, 48–61). Among HIV-positive individuals, the estimated age-standardised incidence rate was more than 300 per 100 000 population in eight countries for females and four for males (appendix pp 44, 62–77). There were similar sex differences in mortality in people with HIV and tuberculosis coinfection: nine countries had an estimated age-standardised mortality rate of more than 50 per 100 000 population for females compared with eight countries for males (appendix pp 45, 62–77).

**Figure 3:**
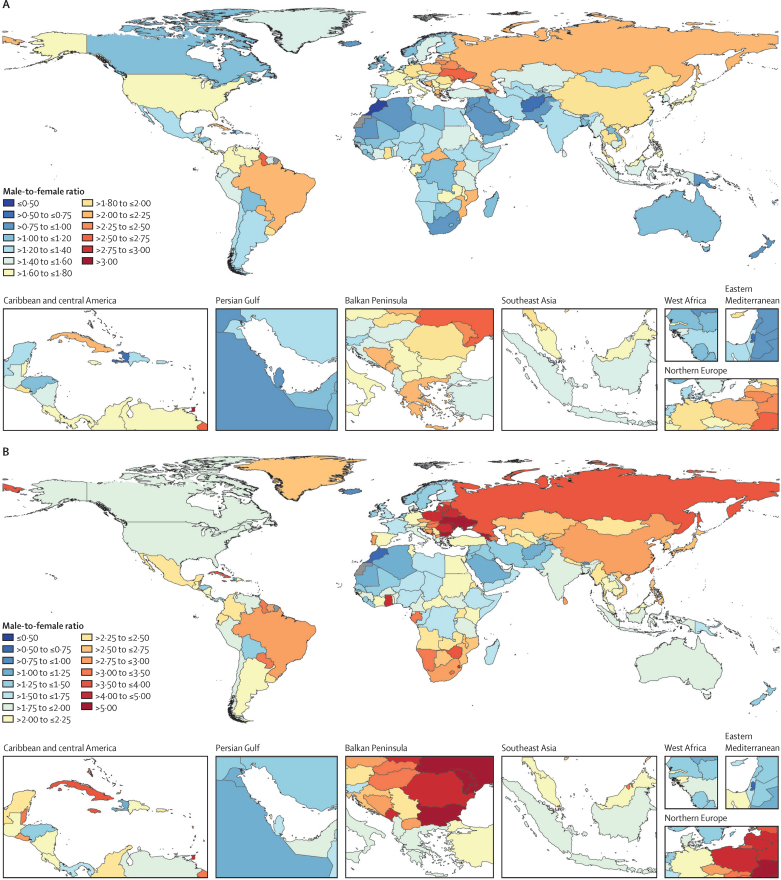
Male-to-female ratio of age-standardised incidence (A) and mortality (B) rates among HIV-negative individuals by geography, 2019

Figure 3 maps the ratio between males and females for age-standardised incidence rates and mortality rates for tuberculosis in HIV-negative individuals in 2019. 74 countries had age-standardised incidence rates that were at least 1·5 times greater and 21 countries had age-standardised incidence rates that were at least two times greater among males than females. The countries with the largest ratios between males and females for age-standardised incidence rates were Armenia, Trinidad and Tobago, Guyana, Ukraine, and Belarus. For age-standardised mortality rates, 105 countries had rates more than two times greater and 17 countries had rates more than four times greater among males than females. Armenia, Georgia, Ukraine, Saint Vincent and the Grenadines, and Moldova had the greatest ratios in age-standardised mortality rates between males and females in 2019. The ratios between males and females for age-standardised incidence and mortality were similar between countries with high and low estimated tuberculosis burdens among HIV-negative individuals. For example, the respective ratios for incidence and mortality were 1·81 (95% UI 1·76–1·88) and 3·04 (2·12–3·98) for China, 1·77 (1·70–1·84) and 2·67 (1·84–3·72) for the Philippines, and 1·24 (1·20–1·29) and 1·91 (1·34–2·62) for India, whereas the respective ratios in countries with lower tuberculosis burden were 1·62 (1·54–1·72) and 2·00 (1·80–2·17) for the USA, 1·61 (1·53–1·69) and 2·55 (2·30–2·96) for Japan, and 1·26 (1·16–1·37) and 1·55 (1·33–2·02) for Finland.

**Figure 4:**
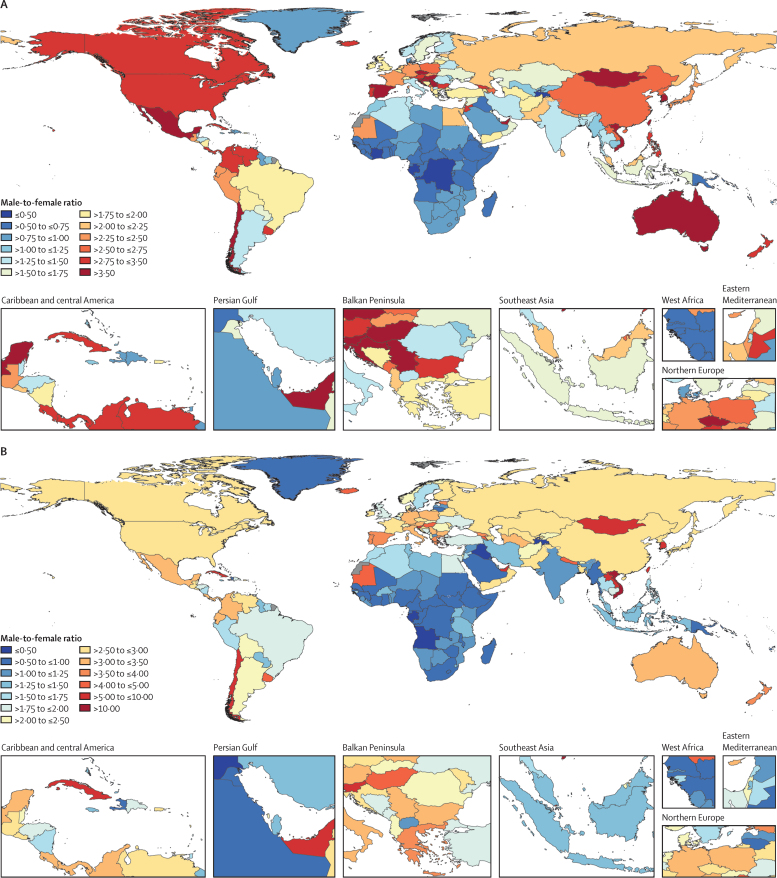
Male-to-female ratio of age-standardised incidence (A) and mortality (B) rates among HIV-positive individuals by geography, 2019

Among HIV-positive individuals, 66 countries and 24 countries had age-standardised incidence rates that were more than two times and three times greater, respectively, among males than females (figure 4A). For age-standardised mortality rates, 87 countries and 16 countries had rates that were more than two times and four times greater, respectively, among males than females (figure 4B). Countries where overall estimated HIV and tuberculosis coinfection burden was low had the largest male-to-female ratios. Conversely, the countries with the highest estimated HIV and tuberculosis coinfection burden (eg, Lesotho, Eswatini, Botswana, Zimbabwe, and South Africa) had male-to-female ratios between 0·90 and 1·24 for age-standardised mortality, but these same countries had ratios below 1 for age-standardised incidence (ratios between 0·76 and 0·94). Overall, 35 and 56 countries had male-to-female ratios below 0·75 (female-to-male ratio 1·33) for age-standardised incidence and mortality among HIV-positive individuals, respectively.

**Figure 5:**
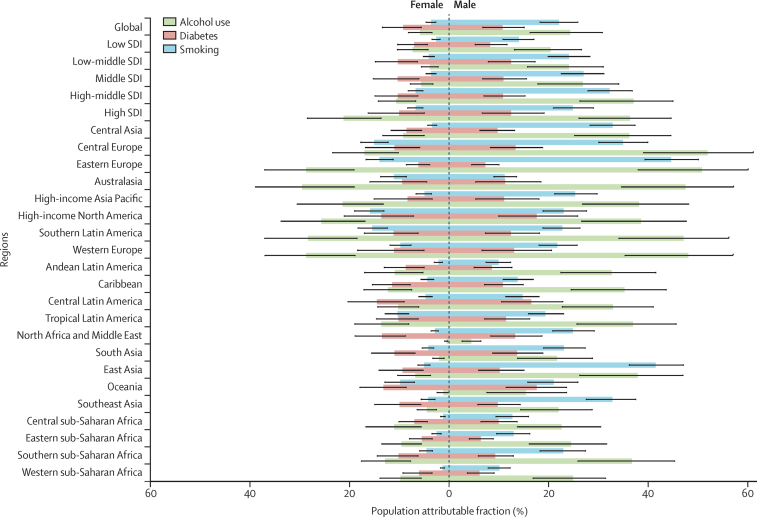
All-age population attributable fractions of tuberculosis deaths due to alcohol use, diabetes, and smoking among HIV-negative men and women by GBD region, 2019 GBD=Global Burden of Diseases, Injuries, and Risk Factors Study.

Globally, in 2019, among HIV-negative individuals, the PAF of tuberculosis deaths due to alcohol was 17·76% (95% UI 11·60–22·83), due to smoking was 15·52% (12·67–18·33), and due to diabetes was 10·20% (6·37–14·47). The global all-age PAFs for tuberculosis deaths due to alcohol, smoking, and diabetes were 4·27 (3·69–5·02), 6·17 (5·48–7·02), and 1·17 (1·07–1·28) times higher, respectively, among males than females in 2019 (figure 5). The regions with the largest sex ratios in the PAF of tuberculosis deaths due to alcohol were in south Asia (11·20 [8·38–15·08]), north Africa and the Middle East (8·53 [6·66–11·53]), and east Asia (5·77 [4·32–7·81]); those due to smoking were in central sub-Saharan Africa (10·59 [8·61–13·08]), central Asia (9·80 [8·18–12·15]), and north Africa and the Middle East (8·86 [7·57–10·99]); and those due to diabetes were in central sub-Saharan Africa (1·44 [1·28–1·62]), high-income Asia Pacific (1·36 [1·14–1·66]), and Oceania (1·34 [1·20–1·50]). If the combined effects of alcohol, smoking, and diabetes were removed, HIV-negative males would still have a modestly higher age-standardised mortality rate than HIV-negative females at the global level (sex ratio 1·28 [1·05–1·49]; appendix pp 104–18).

Among both male and female HIV-positive individuals in 2019, the global PAF of tuberculosis deaths due to unsafe sex was 82·47% (95% UI 80·42–84·25), due to drug use was 5·59% (4·44–7·00), and due to intimate partner violence was 8·04% (4·22–12·42). The global all-age PAF for tuberculosis mortality in people with HIV due to injection drug use was 2·23 (2·03–2·44) times greater among males than females, and the PAF due to unsafe sex was 1·06 (1·05–1·08) times greater among females than males (appendix p 47). The regions with the largest sex ratios in the PAF due to injection drug use between male and females with HIV were in east Asia (5·30 [4·00–6·85]), southeast Asia (5·05 [4·23–5·70]), and central Europe (3·02 [2·82–3·27]). Southern sub-Saharan Africa (19·71% [12·03–29·94]), central sub-Saharan Africa (18·95% [11·06–29·55]), and Oceania (17·41% [11·49–25·16]) are estimated to have the highest PAFs for intimate partner violence among females with HIV (appendix p 47). After removal of the combined effects of unsafe sex, injection drug use, and intimate partner violence, males with HIV would have a 1·21 (1·15–1·28) times higher global age-standardised tuberculosis mortality rate than females with HIV (appendix pp 119–33).

## Discussion

GBD 2019 provides a comprehensive assessment of the global tuberculosis burden, with an emphasis on characterising sex differences across different metrics of tuberculosis burden. We found that age-standardised incidence rates among HIV-negative individuals were more than 1·5 times greater in 74 countries, and age-standardised mortality rates were more than two times greater in 105 countries, in males than in females in 2019. Among HIV-positive individuals, age-standardised incidence and mortality rates were 1·33 times larger in 35 and 56 countries, respectively, for females than males.

These results align with the findings from previous studies that males have a greater burden of tuberculosis than females due to men exhibiting lower levels of health-care use, presenting more advanced stages of the disease when care is sought, and having poorer adherence to anti-tuberculosis treatment.^8^, ^19^, ^20^ Previous work has highlighted that men may delay seeking health care for tuberculosis-suggestive symptoms due to fear or to avoid diagnosis of a serious condition such as tuberculosis or HIV.^20^, ^21^ These fears and anxieties intersect with masculinity expectations, wherein men face societal pressure to neglect symptoms to remain physically and financially strong and capable to satisfy leadership roles.^22^, ^23^, ^24^ The combination of late initiation of tuberculosis treatment and poor treatment adherence might be contributing to age-standardised mortality rates being nearly two times greater among males than among females in the GBD.

Risk factors are also important to the observed sex differences in tuberculosis mortality. Previous work has found that alcohol use^25^ and smoking^12^ are substantial contributors to sex differences in tuberculosis burden. The global fraction of tuberculosis deaths attributable to alcohol consumption and smoking was more than four times and six times higher, respectively, among males than among females. Despite the global diabetes prevalence being very similar for males and females,^11^ we found that the fraction of tuberculosis deaths attributable to diabetes was modestly higher for males. Some evidence suggests that the higher fraction among males might be due to men having poorer tuberculosis treatment outcomes than women in the presence of diabetes comorbidities.^26^, ^27^ Together, the findings underscore that risk factors, particularly alcohol and smoking, along with late treatment initiation, are probably major contributors to the sex differences in tuberculosis mortality among HIV-negative people. Indeed, our results show that the excess tuberculosis mortality among males would decrease from 1·97 (95% UI 1·60–2·26) to 1·28 (1·05–1·49) if the effects of risk factors for disease progression were removed. The heightened exposure to risk factors that contribute to disease progression among men might also be a factor for why males have 50% higher age-standardised incidence rates than females in more than 70 countries.

Although our study found that tuberculosis burden and risk factors are higher among males than females, the burden among females is still unnecessarily large; we estimated more than 500 000 deaths among HIV-negative and HIV-positive females in 2019. Evidence suggests that men indeed delay seeking health care for tuberculosis, contributing to poor case detection among men, but that women generally encounter more barriers to receiving appropriate tuberculosis care.^19^ Across various settings, women, compared with men, face a substantially longer time to tuberculosis diagnosis^28^, ^29^, ^30^, ^31^, ^32^ due to lower priority or attention paid when women present non-specific tuberculosis symptoms,^19^ evidence of tuberculosis diagnostic tools being less sensitive for women,^33^ and gender norms requiring women to negotiate with husbands to seek tuberculosis care.^34^

Moreover, we found that the global burden of tuberculosis was higher among females than among males in HIV-positive individuals. In the countries with the highest HIV and tuberculosis coinfection burden (eg, countries in southern sub-Saharan Africa), our results show that females generally had a greater HIV and tuberculosis coinfection burden than males, with unsafe sex and intimate partner violence being significant contributors. The larger incidence rates among females in these countries might be due to HIV disproportionately affecting women in countries with high HIV burden.^35^, ^36^, ^37^ The larger burden of HIV among women, combined with HIV being the strongest risk factor for progression from latent to active tuberculosis,^38^ compounds the observed differences in these countries.

These findings underscore the need for HIV prevention and treatment to diminish HIV and tuberculosis coinfection burden. Interventions are needed that address multiple causes of women's vulnerability to HIV infection, including poverty, exposure to intimate partner violence, cultural factors that disempower women, such as encouraging marriage to older men, and laws deterring reproductive health services.^39^, ^40^, ^41^

The End TB Strategy sought to reduce tuberculosis incidence to below 85 new cases per 100 000 population by 2020. However, our findings suggest that substantial additional progress needs to be made, with 40 countries having age-standardised incidence rates greater than 170 per 100 000 population, more than double the target of 85 per 100 000, in 2019. Males are particularly at risk for not reaching this target, as the global age-standardised tuberculosis incidence rate for males without HIV was 120 per 100 000 population, compared with 95 per 100 000 population for HIV-negative females in 2019. The excess tuberculosis burden among men found in this study strengthens the view that men should be targeted in screening services and routine diagnostics^6^, ^8^ to achieve equity targets outlined by the SDGs and End TB Strategy. Interventions are needed to engage men in tuberculosis care, with social protections (eg, prevention of loss of employment while receiving care and cash transfers) for families, while actively communicating the importance of early diagnosis to men.^24^ Interventions should also place efforts on improving communication and preventive strategies to support reducing behavioural risk factors such as alcohol use and smoking.

Although the results reported in GBD 2019 reflect tuberculosis burden before the emergence of the COVID-19 pandemic, the worldwide pandemic has important consequences for the global burden of tuberculosis that need to be investigated by tuberculosis programmes. Modelling studies have suggested that tuberculosis deaths could increase up to 20% in the next 5 years.^42^, ^43^ Physical-distancing and mask-wearing policies might help to reduce tuberculosis transmission, but this beneficial effect might be offset by increased opportunities for household transmission.^44^ Prolonged exposure to household contacts is an important risk factor for tuberculosis transmission, and the duration of infectiousness might be further extended if there are disruptions to tuberculosis treatment due to health system overload as a result of COVID-19. Tuberculosis programmes, once they have evaluated the impact of COVID-19 on tuberculosis for any severe setbacks, might need to consider the recalibration of global targets.

Overall, despite different estimation methods, the global burden of tuberculosis estimated by GBD is similar to that produced by WHO.^3^ A concern raised previously was that past iterations of GBD and WHO estimates produced discrepancies in estimated tuberculosis mortality.^45^ For example, in earlier iterations, GBD 2016^2^ estimated 1·45 million tuberculosis deaths, whereas WHO 2017^46^ estimated 1·67 million tuberculosis deaths in 2016. However, the latest estimates show that the differences are decreasing at the global level. Although GBD 2019 estimated slightly lower global incident cases at 9·65 million (95% UI 8·48–11·03) than WHO's estimate of 10·0 million (9·0–11·1), both GBD 2019 and WHO estimated approximately 1·40 million deaths in 2019 (1·40 million deaths by GBD 2019, and 1·39 million deaths by WHO). However, there are some differences in sex-specific estimates: GBD 2019 estimated that 56% of global incident tuberculosis cases and 65% of global tuberculosis deaths were among males, whereas, for the same year, WHO estimated that 63% of global incident tuberculosis cases and 61% of global tuberculosis deaths were among males. The higher percentage for tuberculosis mortality than for tuberculosis incidence in males in GBD 2019 might be expected due to several factors, such as men presenting advanced stages of tuberculosis when care is sought and heightened exposure to risk factors.^19^, ^47^, ^48^, ^49^ Previous work has shown that smoking and alcohol consumption further contribute to poor tuberculosis treatment outcomes and compliance among men.^47^, ^48^, ^49^

The tuberculosis burden results should be interpreted in the context of the following limitations. First, there are gaps in data availability across countries, age groups, and time periods. In locations without reliable vital registration data, mortality estimates are driven by verbal autopsy studies, which have been shown to have modest sensitivity in identifying tuberculosis deaths.^50^, ^51^, ^52^ We believe that this bias has been minimised through excluding verbal autopsy studies from countries with high HIV burden. Second, because the GBD 2019 tuberculosis results are based on available data and modelling, lags in reporting of vital registration data indicate that tuberculosis mortality estimates for most recent years rely on modelling processes. Estimates for locations and years where data are sparse are reflected by wide UIs. Third, we have not yet been able to quantify the burden of tuberculosis attributable to indoor air pollution due to a lack of objectively measured longitudinal data.^53^ Similarly, the burden attributable to malnutrition has not yet been quantified because there is insufficient evidence of a causal relationship and limited data on the risk of tuberculosis across different levels of malnutrition.^54^ Fourth, there are challenges in accurately estimating sex differences in tuberculosis burden due to the way age-sex-specific estimates are reported in tuberculosis prevalence surveys. Prevalence surveys often report data separately by age (for both sexes combined) and by sex (for all ages combined). To extract age-sex-specific data from these surveys, we used the age distribution for both sexes in the study to split the all-age sex-specific data. Data extraction would be more accurate if tuberculosis prevalence surveys reported data by age and sex to improve modelling inputs. Finally, there remain major challenges in our statistical triangulation approach, in which there are difficulties in computing consistent estimates between tuberculosis death rates and prevalence data from national surveys. This is particularly problematic for locations in sub-Saharan Africa where there are few prevalence surveys or reliable cause of death data. Closer analyses have shown that even when prevalence and cause of death data are available, there are inconsistencies in the sources. In Bangladesh, for example, we found that a national prevalence survey^55^ estimated tuberculosis prevalence to be approximately 300 per 100 000 population, but verbal autopsy sources^56^, ^57^ reported mortality rates of less than 10 per 100 000 population. Together, the two sources suggest that Bangladesh has one of the lowest case fatality rates in the world for tuberculosis. In GBD 2019, we resolved inconsistencies in data sources by excluding less reliable sources that contradicted others.

GBD 2019 has made several methodological improvements compared with earlier GBD iterations.^2^, ^10^ First, we used a novel Bayesian meta-regression method to incorporate uncertainty from the input data in our mortality-to-incidence ratio approach. Second, we addressed the potential for misclassification of tuberculosis deaths as pneumonia deaths for children younger than 15 years. We made this adjustment using data from a systematic review^58^, ^59^, ^60^, ^61^, ^62^, ^63^, ^64^, ^65^ to redistribute pneumonia deaths in cause of death data to tuberculosis. Third, we standardised our adjustment to prevalence surveys that used smear-positive tuberculosis as the case definition rather than bacteriologically confirmed tuberculosis, while incorporating a novel adjustment to recalibrate studies that used symptoms only as the screening method compared with using both symptoms and chest x-ray. Finally, our statistical triangulation approach was improved by incorporating data on remission and excess mortality, while also resolving discrepancies in morbidity and mortality data.

As countries refine national tuberculosis programmes and strategies to end the tuberculosis epidemic, the excess burden experienced by males should be more widely realised and monitored. The greater incidence and mortality rates among men found in GBD 2019 indicate that men are not fully accessing tuberculosis services and are remaining infectious in the community for substantial periods of time. Targeting the burden of disease in males will diminish the overall burden of tuberculosis and will be crucial in reaching both equity and burden targets outlined by the SDGs and the End TB Strategy. Furthermore, tuberculosis programmes should examine key risk factors that are contributing to disproportionate tuberculosis burden in males, such as alcohol and smoking, and liaise with risk factor control initiatives aiming to reduce alcohol use and smoking. Reaching equity goals will also require identifying and addressing inequalities in health facilities where women often encounter barriers to accessing tuberculosis diagnostic services. These efforts should occur in parallel with addressing risk factors for women's vulnerability to HIV infection to minimise excess HIV and tuberculosis coinfection burden among females in the countries with highest HIV burden.

## Data sharing

To download the data used in these analyses, please visit the Global Health Data Exchange GBD 2019 website.

## Declaration of interests

JMR reports grants or contracts from the US National Institutes of Health (NIH) and consulting fees from the US Agency for International Development, all outside the submitted work. KEL reports support for the present manuscript from the Bill & Melinda Gates Foundation. CATA reports grants or contracts and consulting fees from Johnson & Johnson (Philippines), all outside the submitted work. IF reports payment or honoraria for lectures, presentations, speaker's bureaus, manuscript writing, or educational events from Avicenna Medical and Clinical Research Institute, all outside the submitted work. AmG reports support for the present manuscript, grants, and contracts from Sistema Nacional de Investigadores de Panamá, outside the submitted work. KeK reports other support from UGC Centre of Advanced Study, CAS II, Department of Anthropology, Panjab University, Chandigarh, India, outside the submitted work. MJP reports grants or contacts from Merck Sharp & Dohme, GlaxoSmithKline, Pfizer, Boehringer Ingelheim, Novavax, Bayer, Bristol Myers Squibb, AstraZeneca, Sanofi, IQVIA, BioMerieux, WHO, EU, Seqirus, FIND, Antilope, DIKTI, LPDP, and Budi; consulting fees from Merck Sharp & Dohme, GlaxoSmithKline, Pfizer, Boehringer Ingelheim, Novavax, Quintiles, Bristol Myers Squibb, AstraZeneca, Sanofi, Novartis, Pharmerit, IQVIA, and Seqirus; participation on a data safety monitoring board or advisory board for Asc Academics as an adviser; and stock or stock options in Ingress Health, Health-Ecore, and Pharmacoeconomics Advice Goningen, all outside the submitted work. AmR reports payment or honoraria for lectures, presentations, speaker's bureaus, manuscript writing, or educational events from Avicenna Medical and Clinical Research Institute, outside the submitted work. OR reports grants or contracts from the Research Foundation of Rigshospitalet and A P Møller Foundation, all outside the submitted work. JAS reports grant support, paid to their institution, for the present manuscript from the Bill & Melinda Gates Foundation.
