# Supplementary material for: Global, regional, and national sex differences in the global burden of tuberculosis by HIV status, 1990–2019: results from the Global Burden of Disease Study 2019
Source: Lancet Infect Dis. 2022 Feb;22(2):222–41. doi: 10.1016/S1473-3099(21)00449-7 (PMC8799634; doi:10.1016/S1473-3099(21)00449-7)
Supplement: Supplementary appendix [file mmc1.pdf]

THE LANCET
Infectious Diseases

Supplementary appendix

This appendix formed part of the original submission and has been peer reviewed. We post it as supplied by the authors.

Supplement to: GBD 2019 Tuberculosis Collaborators. Global, regional, and national sex differences in the global burden of tuberculosis by HIV status, 1990–2019: results from the Global Burden of Disease Study 2019. *Lancet Infect Dis* 2021; published online Sept 23. https://doi.org/10.1016/S1473-3099(21)00449-7.

Supplementary Appendix to “Global, regional, and national sex differences in the global burden of tuberculosis by HIV status, 1990-2019: results from the Global Burden of Disease Study 2019”

This appendix provides further methodological detail and results for “Global, regional, and national sex differences in the global burden of tuberculosis by HIV status, 1990-2019: results from the Global Burden of Disease Study 2019”

All the material in the paper itself is novel although it builds off previous GBD works[1–4]. However, that parts of the supplemental methods appendix include sections adapted from the GBD Capstones published in The Lancet last year.[1,2] References are provided for reproduced sections.

Table of Contents

Methods ... 3

Case Definition ... 3

Overview ... 5

Fatal Tuberculosis ... 6

eFigure 1. Flowchart ... 6

Modelling fatal TB ... 6

Estimating HIV-TB ... 14

eFigure 3. Flowchart ... 14

Non-fatal Tuberculosis ... 16

eFigure 4. Flowchart ... 17

Estimating TB incidence ... 18

Estimating TB prevalence ... 19

HIV-TB incidence and prevalence ... 33

eTable 5. GATHER checklist of information that should be included in reports of global health estimates, with description of compliance and location of information ... 37

Results ... 41

eFigure 5. Temporal trends of male-to-female ratios of age-standardized tuberculosis mortality rates among (A) HIV-negative individuals and (B) HIV-positive individuals by SDI quintile, 1990–2019 ... 41

eFigure 6. Age-standardized incidence rate per 100,000 population among HIV-negative individuals in 2019 by geography for males (A) and females (B) ... 42

eFigure 7. Age-standardized mortality rate per 100,000 population among HIV-negative individuals in 2019 by geography for males (A) and females (B) ... 43

eFigure 8. Age-standardized incidence rate per 100,000 population among HIV-positive individuals in 2019 by geography for males (A) and females (B) ... 44

eFigure 9. Age-standardized mortality rate per 100,000 population among HIV-positive individuals in 2019 by geography for males (A) and females (B) ... 45

eFigure 10. Global age-sex distribution of tuberculosis incidence and deaths in HIV-positive individuals in 2019 ... 46

eFigure 11. All-age population attributable fractions of tuberculosis deaths due to drug use, intimate partner violence, and unsafe sex among HIV-positive men and women by GBD regions in 2019 ... 47

eTable 6. Tuberculosis cases and age-standardized rates of incidence and mortality per 100,000 population among HIV-negative males and females for 204 countries and territories in 2019 ... 48

eTable 7. Tuberculosis cases and age-standardized rates of incidence and mortality per 100,000 population among HIV-positive males and females for 204 countries and territories in 2019 ... 61

eTable 8. All-age population-attributable fractions of tuberculosis due to alcohol use, diabetes, and smoking among HIV-negative men and women for 204 countries and territories in 2019 .... **76**

eTable 9. All-age population-attributable fractions of tuberculosis due to drug use, intimate partner violence, and unsafe sex among HIV-positive men and women in 2019 for 204 countries and territories in 2019 .... **89**

eTable 10. Tuberculosis age-standardized risk-deleted mortality rate for alcohol use, diabetes, smoking, and all three risk factors combined among HIV-negative men and women for 204 countries and territories in 2019.................... **102**

eTable 11. Tuberculosis age-standardized risk-deleted mortality rate for drug use, intimate partner violence, unsafe sex, and all three risk factors combined among HIV-positive men and women for 204 countries and territories in 2019 .... **117**

SDI Analysis **136**

Comparative risk assessment **138**

- **Alcohol use** **138**
- **Smoking** **147**
- **Diabetes** **153**
- **Unsafe sex** **158**
- **Drug use** **162**
- **Intimate partner violence** **166**

Author Contributions …………………………………………………. **172**

Methods

Case definition

Tuberculosis (TB) is an infectious disease caused by Mycobacterium tuberculosis. The case definition includes all forms of TB, including pulmonary TB and extrapulmonary TB, which are bacteriologically confirmed or clinically diagnosed. For TB, the ICD 10 codes are A10-A19.9, B90-B90.9, K67.3, K93.0, M49.0, P37.0, and ICD 9 codes are 010-019.9, 137-137.9, 138.0, 138.9, 139.9, 320.4, 730.4-730.6. For HIV-TB, the ICD 10 code is B20.0.

Latent TB infection is defined as an infection with Mycobacterium tuberculosis, without any symptoms or signs of active TB disease.

We separately estimated the incidence and prevalence of multidrug-resistant tuberculosis and extensively drug-resistant tuberculosis by HIV status. The case definitions are shown below.

(1) Multidrug-resistant TB without extensive drug resistance: a form of TB (among HIV-negative individuals) that is resistant to the two most effective first-line anti-tuberculosis drugs (isoniazid and rifampicin), but is not resistant to any fluoroquinolone and any second-line injectable drugs (amikacin, kanamycin, or capreomycin).

(2) Extensively drug-resistant TB: a form of TB (among HIV-negative individuals) that is resistant to isoniazid and rifampicin, plus any fluoroquinolone and any second-line injectable drugs.

(3) Drug-susceptible TB: TB (among HIV-negative individuals) that is susceptible to isoniazid and rifampicin.

(4) HIV/AIDS - Multidrug-resistant TB without extensive drug resistance: a form of TB (among HIV-positive individuals) that is resistant to the two most effective first-line

anti-tuberculosis drugs (isoniazid and rifampicin), but is not resistant to any fluoroquinolone and any second-line injectable drugs (amikacin, kanamycin, or capreomycin).

(5) HIV/AIDS - Extensively drug-resistant TB: a form of TB (among HIV-positive individuals) that is resistant to isoniazid and rifampicin, plus any fluoroquinolone and any second-line injectable drugs.

(6) HIV/AIDS - Drug-susceptible TB: TB (among HIV-positive individuals) that is susceptible to isoniazid and rifampicin.

Overview

We analyzed vital registration data, verbal autopsy data, sample-based vital registration data, and mortality surveillance data, using the Cause of Death Ensemble model (CODEm) to estimate TB mortality among HIV-negative individuals. We estimated HIV-TB mortality using a population attributable fraction approach taking into account baseline risk. We also used a population attributable fraction approach to estimate the fraction of HIV-negative TB deaths attributable to MDR-TB, and the fraction of HIV-TB deaths attributable to MDR-TB, respectively, and used these fractions to split TB deaths and HIV-TB deaths by drug-resistance type.

For estimating non-fatal TB, we analyzed all available data sources, including annual case notifications, prevalence surveys, population-based tuberculin surveys, and estimated TB cause-specific mortality, to generate internally consistent estimates of incidence, prevalence, and mortality using DisMod-MR 2.1, a Bayesian meta-regression tool. To distinguish HIV-TB from all forms of TB, we applied the proportions of HIV-TB cases among all TB cases estimated from a mixed-effects regression to TB incident and prevalent cases. We then applied the estimated proportions of TB cases with MDR-TB and HIV-TB cases with MDR-TB to our predicted TB cases and HIV-TB cases, respectively, to generate MDR-TB cases by HIV status.

Fatal Tuberculosis[1]

eFigure 1[1]. Tuberculosis (TB) mortality: input data, analytical processes, and outputs

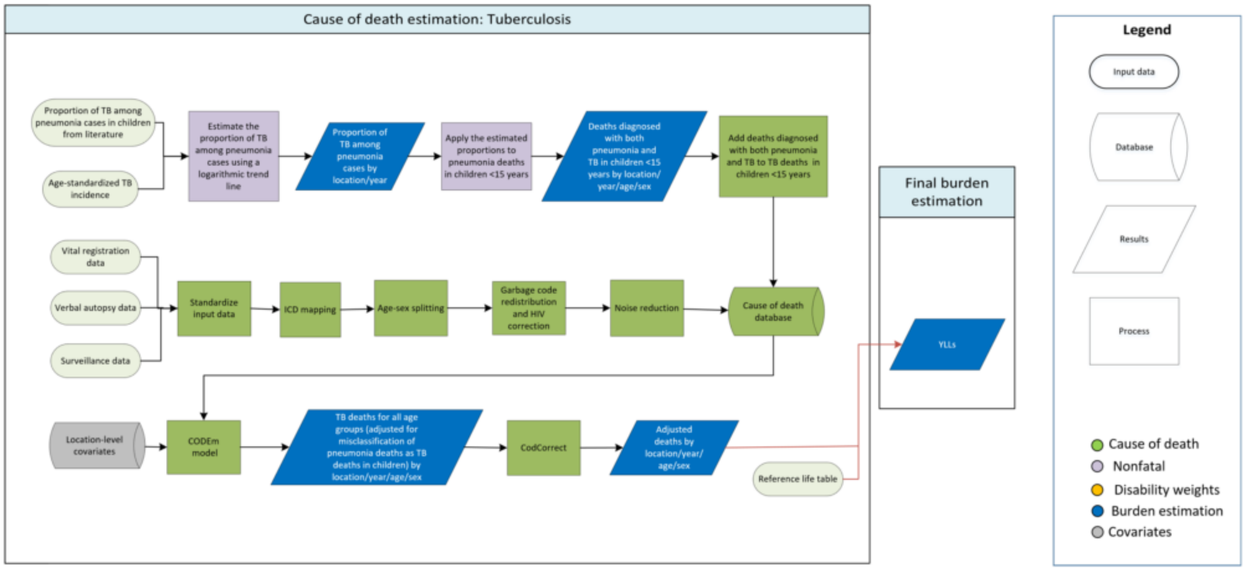

Note: Flowchart with code available at http://ghdx.healthdata.org/gbd-2019/code/cod-10

Modelling fatal TB

Input data for modelling tuberculosis (TB) mortality among HIV-negative individuals include vital registration, verbal autopsy, and surveillance data. The quality and comparability of the cause of death data were assessed and enhanced through multiple steps, including redistribution of garbage codes to underlying causes of death following GBD algorithms and adjustment for misclassified HIV deaths (ie, HIV deaths being assigned to other underlying causes of death such as TB because of stigma or misdiagnosis).[1] Details on redistribution and HIV misclassification adjustment are outlined in the subsequent sections (See pg. S20–S45 of Vos et al 2020[1] for extended details of CoD data processing). Verbal autopsy data in countries with age-standardized HIV prevalence greater than 5% were removed because of a high probability of misclassification, as verbal autopsy studies have poor validity in distinguishing HIV deaths from HIV-TB deaths. GBD 2019 also assessed the overall cause of death (CoD) data quality for each country (based on completeness, garbage coding, cause list detail, and time

periods covered), and assigned a quality score ranging from 0 stars (poorest) to 5 stars (best); a quality score of 4 to 5 is considered high quality (eFigure 2).

eFigure 2. Overall data quality by country

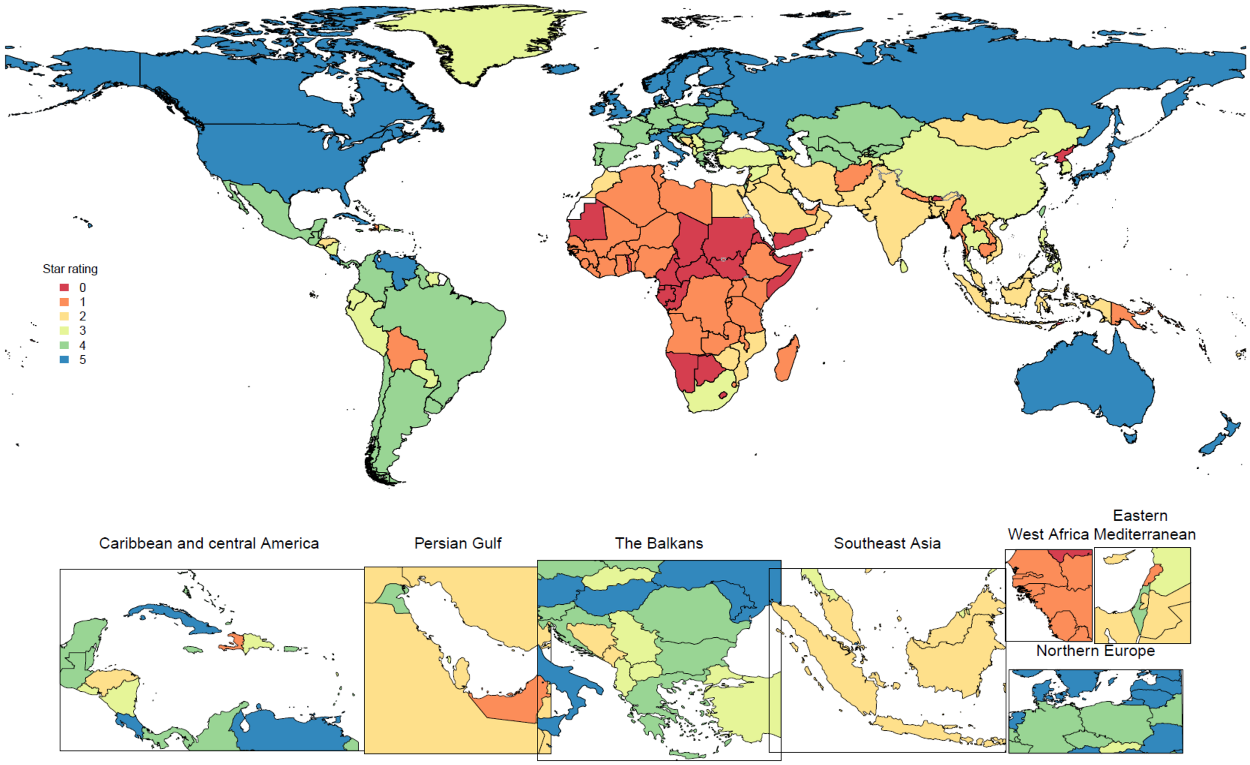

Redistribution[1]

A crucial aspect of enhancing the comparability of data for CoD is to deal with uninformative, so-called garbage codes. Garbage codes to which deaths were assigned should not be considered as the underlying CoD--for example: “heart failure”, “ill-defined cancer site”, “senility”, “ill-defined external causes of injuries”, and “septicaemia”. The methods for redistributing these garbage-coded deaths were outlined in detail in Naghavi et al[5], and the underlying algorithm for redistributing deaths assigned to these codes has not changed since GBD 2013.

Redistribution of TB CoD data: Regress garbage codes versus non-garbage codes[1]

For each redistribution package, we defined the "universe" of data as all deaths coded to either the package's garbage codes or the package's redistribution targets for each country, year, age, and sex. We then ran a regression based on the following equation separately for each target group and sex:

$$TG_{crt} = \alpha + \beta_1 Gar_{crt} + \beta_2 Age_{crt} Gar_{crt} + \theta_r Gar_{crt} + \gamma_r + \varepsilon_{ct}$$

Where:

TG_{crt} = percentage of deaths within the given garbage code's universe that were coded to a given target group, by country

Gar_{crt} = percentage of deaths within the given garbage code's universe that were coded to a given set of garbage codes

Age_{CRT} =age interaction term for the fixed effect on the interaction of garbage and age

α = constant

β_2 = slope coefficient describing the association between the interaction $Age_{crt}Gar_{crt}$ and G_{crt}

β_2 = slope coefficient describing the association between the interaction $Age_{crt}Gar_{crt}$ and G_{crt}

γ_r = region-specific random intercept (or super-region if the random effect on region is not significant)

θ_r = region-specific random slope (or super-region if the random effect on region is not significant)

ε_{ct} = standard error, normally distributed and calculated by bootstrapping

This regression was adjusted from GBD 2013 to include fixed effects on the interaction of garbage and age to ensure smooth age patterns. We made this decision after investigating diagnostic visualisations that showed unlikely gaps between proportions assigned to different age groups.

Redistribution of TB CoD data: Computing redistribution uncertainty[1]

We assigned redistribution variance to each data point by calculating residual variance from a regression predicting the percentage of garbage coded deaths redistributed to a cause, given the proportion of garbage codes we observed for that location, year, age, sex, cause, and the age standardized relative rate of major garbage codes across all causes. If there is a cause that has greater residual variance, we assume greater redistribution uncertainty.

To calculate variance, a dataset was generated containing percent garbage by location, year, age, sex, and cause, where percent garbage is determined by the equation:

$$pct_{garbage} = \frac{deaths_{redistributed} - deaths_{raw}}{deaths_{redistributed}}$$

A mixed-effect linear regression model was then fit to predict the logit percent of deaths from redistribution by age-standardized relative rate of major garbage codes.

$$logit\left(pct_{garbage_{ij}}\right) = \beta_0 + \beta_1 * \log\left(ASR_{majorgarbage_{ij}}\right) + \beta_2 * 15yearage_{ij} + \gamma_{1j} * \log\left(ASR_{majorgarbage_{ij}}\right) + u_j + e_{ij},\ \ \theta_{\{i\}} \sim N(0, \sigma^2)$$

Where:

i indexes dataset-location-year-age-sex-cause data points nested within *j* groups by GBD region

ASR major garbage: age standardized relative rate of major garbage

Residual variance, as estimated by the MAD, was calculated for each cause, sex, and age. The next step was to use the residual variance to calculate uncertainty around each data point in the CoD database. First, we calculated the percent garbage of each data point by treating all deaths that could not be directly mapped to a GBD cause as garbage. Percent garbage was calculated as:

$$pct_{garbage} = \frac{deaths_{redistributed} - deaths_{corrected}}{deaths_{corrected}}$$

Where:

$death_{corrected}$: deaths post misdiagnosis correction

$deaths_{redistributed}$: deaths post redistribution

Residual variance was matched to each data point and 100 draws were sampled from a normal distribution by using the cause, age, sex, specific residual variance, and mean of 0. The logit transformed percent garbage was added to each value in the distribution. Each draw was then transformed out of logit space, and the post-redistribution deaths were calculated as

$$deaths = \frac{deaths_{corrected}}{1 - pct\_garbage}$$

Draws of deaths were processed through noise reduction before calculating the final redistribution variance passed to modeling in CODEm, which was added to the total data variance. The mean of the draws was not used as the final estimate because it was found that the logit transformation biases the distribution of cause fractions higher than if only point estimates are used.

HIV/AIDS misclassification correction[1]

In many location-years, certain causes of death known to be comorbid with HIV/AIDS (e.g., tuberculosis, other infectious diseases) are seen to have age patterns that diverge from those observed in location-years without widespread HIV epidemics and are in fact more reflective of HIV mortality trends. To identify these instances, a global relative age pattern is generated by using all VR deaths in countries with observed HIV prevalence less than 1% by using the following equation

$$RR_{asc} = \frac{R_{asc}}{\bar{x}(R_{65sc}, R_{70sc}, R_{75sc})}$$

Where:

RR_{asc} is the relative death rate for age group a, sex s, cause c

R_{asc} is the rate for that age group

$\bar{x}(R_{65sc}, R_{70sc}, R_{75sc})$ is the mean of the rates in ages 65–69, 60–74, and 75–79 for that sex and cause.

This is preferable to comparing mortality rates because we are able to isolate divergence in age pattern while accounting for varying levels of overall mortality by fixing death rates to age groups that are unlikely to be confounded by the presence of HIV. Expected deaths for an identified cause were then determined by the equation:

$$ED_{lyasc} = \bar{x}\left(R_{ly65sc}, R_{ly70sc}, R_{ly75sc}\right) \times p_{lasc} \times RR_{asc}$$

Where:

ED_{lasc} are deaths for location l, year y, age group a, sex s, and cause c;

$\bar{x}(R_{l65sc}, R_{l70sc}, R_{l75sc})$ is the mean of the rates for ages 65–69, 60–74, and 75–79 for that location-year-sex-cause

p_{lasc} is the population for that location-year-age-sex-cause

RR_{asc} is the global standard relative rate determined in the previous step for that age-sex-cause.

The expected deaths remain attributed to that particular cause, while the difference between observed and expected are reallocated to HIV/AIDS.

Methods for correcting for a potential misclassification of tuberculosis deaths as pneumonia deaths in children

First, we estimated the proportion of tuberculosis among pneumonia cases as a function of age-standardized TB incidence using data from eight clinical studies[6–13] reporting the proportion of pneumonia cases that had tuberculosis (or the data to calculate them) and age-standardized TB incidence estimates. We used a logarithmic trend line to fit these data. In GBD

2019, we applied the estimated proportions to pneumonia deaths reported in data among children younger than 15 years to compute the number of deaths diagnosed with both pneumonia and TB, which were then added to child TB data. Following this correction in our input data, the CODEm model was ran to provide location-year-age-sex specific estimates.

The Cause of Death Ensemble model (CODEm)

TB mortality trends among HIV-negative individuals was modelled using the Cause of Death Ensemble modelling (CODEm) strategy, which is based on five general principles: identifying all available data, enhancing the comparability and quality of the dataset, developing a diverse set of possible models, assessing the predictive validity of all models, and selecting the models with the best performance in out-of- sample predictive validity tests. Possible models were identified using a covariate selection algorithm that yielded many plausible combinations of covariates which were then run through four classes of models. These model classes include modeling natural log rates and logit cause fractions using mixed effects linear models and spatiotemporal Gaussian Process Regression models. This generated a large variety of models that competed in predictive validity tests. An ensemble of CODEm models that performed best on out-of-sample predictive validity tests was then selected. Details on how candidate models were developed, evaluated, and selection of best model are found elsewhere.[14]

eTable 1: Candidate covariates and priors evaluated in CODEm for tuberculosis

	**Covariate**	**Direction**
Level 1	TB prevalence<br>Latent TB infection prevalence<br>TB summary exposure value (SEV) scalar<br>Liters of alcohol consumed per capita<br>Smoking prevalence<br>Cigarettes per capita<br>Fasting plasma glucose<br>TB strain prevalence-weighted transmission risk	+<br>+<br>+<br>+<br>+<br>+<br>+<br>+
Level 2	Healthcare access and quality (HAQ) Index<br>Adult underweight proportion<br>Indoor air pollution<br>Outdoor air pollution<br>Population density	-<br>+<br>+<br>+<br>+
Level 3	Log lag distributed income (LDI) per capita<br>Education (years per capita)<br>Socio-demographic Index (SDI)	-<br>-<br>-

Estimating fatal HIV-TB

eFigure 3[1]. HIV-Tuberculosis mortality: input data, analytical processes, and outputs

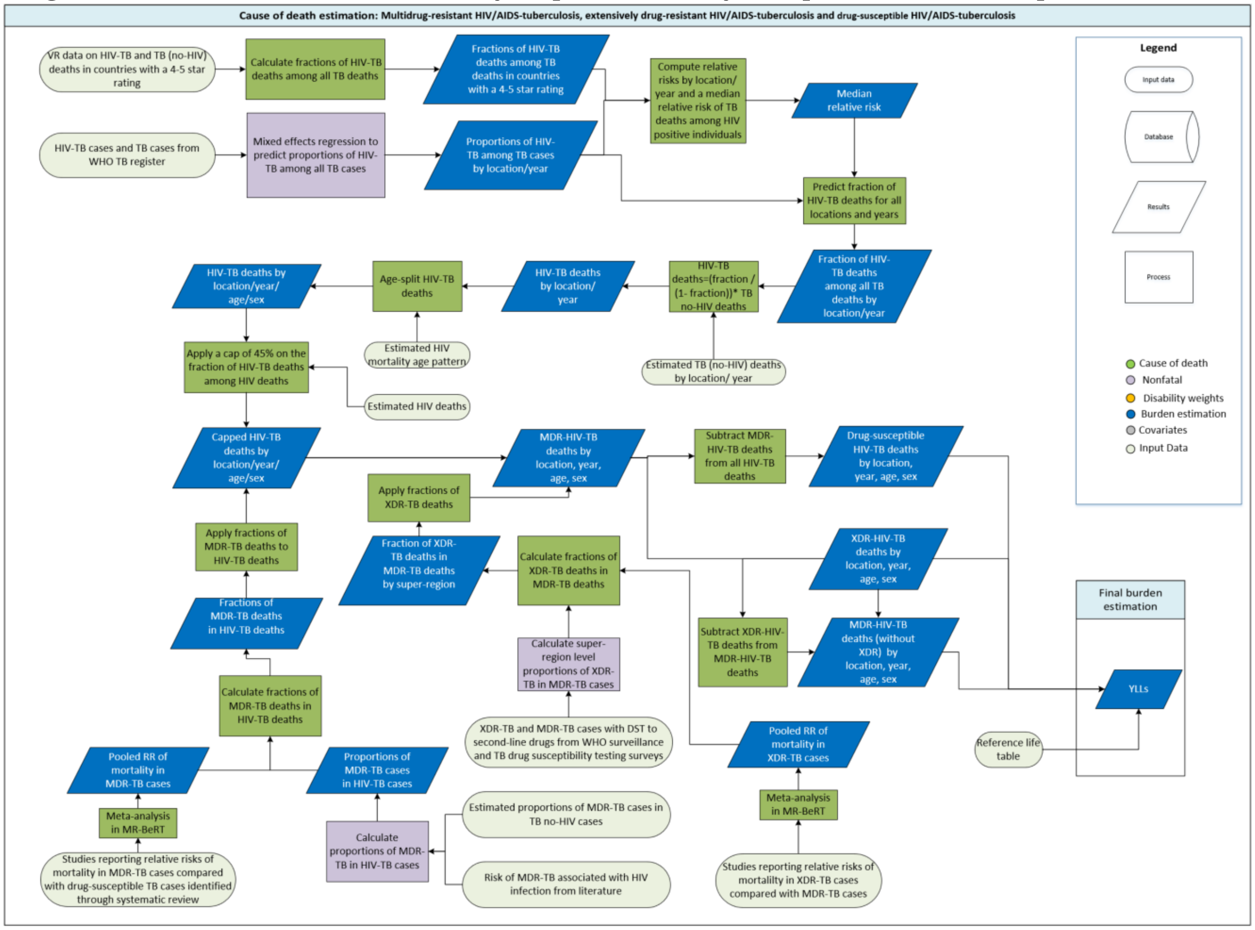

Note: Flowchart with code available at http://ghdx.healthdata.org/gbd-2019/code/cod-6

Input data include: (1) of vital registration data from countries with a four- or five-star rating where cause of death data for directly coded HIV-TB and tuberculosis (TB) were available, and (2) the number of TB cases (new and re-treatment) recorded as HIV-positive and the number of TB cases (new and re-treatment) with an HIV test result recorded in the TB register from the World Health Organization (WHO).

To determine TB deaths in HIV-positive individuals, we first computed the fraction of HIV-TB deaths among all TB deaths using vital registration data from countries with a four-or five-star rating. We also calculated the proportion of TB cases that are HIV-positive. We used

these proportions as input data for a mixed effects regression to predict the proportions of HIV-TB cases among all TB cases for all locations and years using an adult HIV death rate covariate. We estimated the fraction of HIV-TB deaths among all TB deaths in each location and year ($D_{c,y}$), defined by:

$$D_{c,y} = \frac{P_{c,y}RR}{P_{c,y}RR + 1 - P_{c,y}}$$

where $P_{c,y}$ is the proportion of HIV-TB cases among all TB cases and RR is the relative risk of TB deaths in HIV positive individuals, defined by:

$$RR = \frac{D_{c,y}P_{c,y} - D_{c,y}}{D_{c,y}P_{c,y} - P_{c,y}}$$

We took the median relative risk (RR) from each calculation. We then applied the median RR and the predicted proportions of HIV-TB cases among all TB cases to get the fractions of HIV-TB deaths among all TB deaths for all locations and years. Location-year-specific HIV-TB deaths were then calculated using the following equation:

$$Deaths_{HIV-TB} = \frac{D_{c,y}}{1 - D_{c,y}} Deaths_{TB}$$

where $Deaths_{TB}$ is location-year specific deaths from the CODEm TB no-HIV model. Finally, we applied the age-sex pattern of the HIV mortality estimates to these HIV-TB deaths to generate location-year-age-sex-specific HIV-TB deaths. As the HIV-TB deaths were estimated based on the fraction of HIV-TB deaths among all TB deaths, the total number of HIV-TB deaths could exceed the total number of HIV deaths in some locations. To avoid this, we applied a cap of 45% on the fraction of HIV-TB deaths among HIV deaths, based on a review by Cox

and colleagues, 2010[15], and a systematic review and meta-analysis by Ford and colleagues, 2016.[16]

Modelling non-fatal TB[1]

Input data include annual case notifications, data from prevalence surveys, and estimated cause specific mortality (CSMR) of TB among HIV-positive and HIV-negative individuals. We divided the inputs on prevalence, incidence, and CSMR by the estimated latent TB infection (LTBI) prevalence weighted by the risk of progression to active TB in order to model TB among those at risk in each country. From these inputs, we modeled remission and used estimated remission to compute excess mortality to give more guidance to the model. We used DisMod-MR 2.1, the GBD Bayesian meta-regression tool that adjusts for differences in methods between data sources and imposes consistency between data for different parameters. We then multiplied the DisMod-MR 2.1 outputs by the prevalence of LTBI to get population-level estimates of incidence and prevalence. We explain in more detail below the preparation of each of the input data sources and the modelling in DisMod-MR 2.1. Updated systematic reviewers were done in GBD 2019 for TB prevalence surveys and LTBI tuberculin surveys. The search terms, number of studies identified, and number of studies included are shown in the table below:

eFigure 4[1]. Tuberculosis (TB) morbidity: input data, analytical processes, and outputs

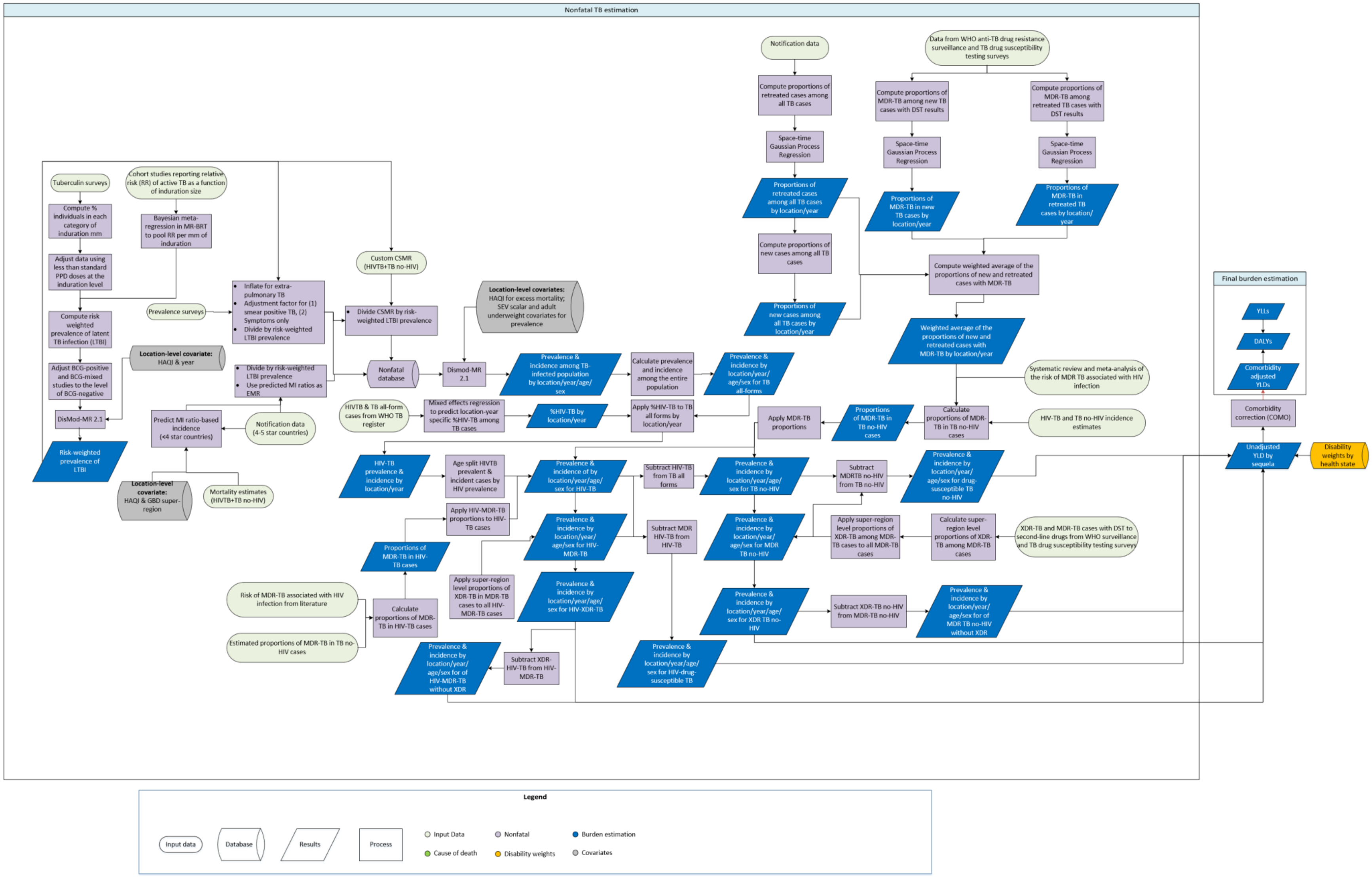

Note: Flowchart with code available at http://ghdx.healthdata.org/gbd-2019/code/nonfatal-13

Outcome	Search Terms	Total number of studies identified	Number of studies included
Tuberculosis	Pubmed: ("tuberculosis"[MeSH] OR tuberculosis[Title/Abstract]) OR TB[Title/Abstract] OR Mycobacterium tuberculosis[Title/Abstract] AND prevalence[Title/Abstract] AND ("2017/09/01"[PDAT] : "2019/02/13[PDAT]) NOT (animals[MESH] NOT humans[MESH])	997	2
LTBI (tuberculin surveys)	Pubmed: ("tuberculin survey"[tiab] OR (("risk"[MeSH Terms] OR "risk"[tiab] OR "risk of"[tiab]) AND ("tuberculosis"[MeSH Terms] OR "tuberculosis"[tiab] OR "tuberculous"[tiab]) AND ("infection"[MeSH Terms] OR "infection"[tiab])) OR (("risk"[MeSH Terms] OR "risk"[tiab] OR "risk of"[tiab]) AND TB[tiab] AND ("infection"[MeSH Terms] OR "infection"[tiab])) OR "latent tuberculosis infection"[tiab] OR "latent TB infection"[tiab] OR "latent tuberculosis"[MESH]) AND ("survey"[tiab] OR "surveys"[tiab]) NOT (animals[MESH] NOT humans[MESH]) ("2017/09/01"[PDAT] : "2019/02/13"[PDAT])<br><br>Google Scholar: ("tuberculin survey" OR "risk of tuberculous infection" OR "risk of tuberculosis infection" OR "risk of TB infection" OR "latent tuberculosis infection" OR "latent TB infection") AND "survey". (09-01-2017 to 02-13-2019).	42	0

PRISMA Diagram of TB All Forms Prevalence in GBD2019

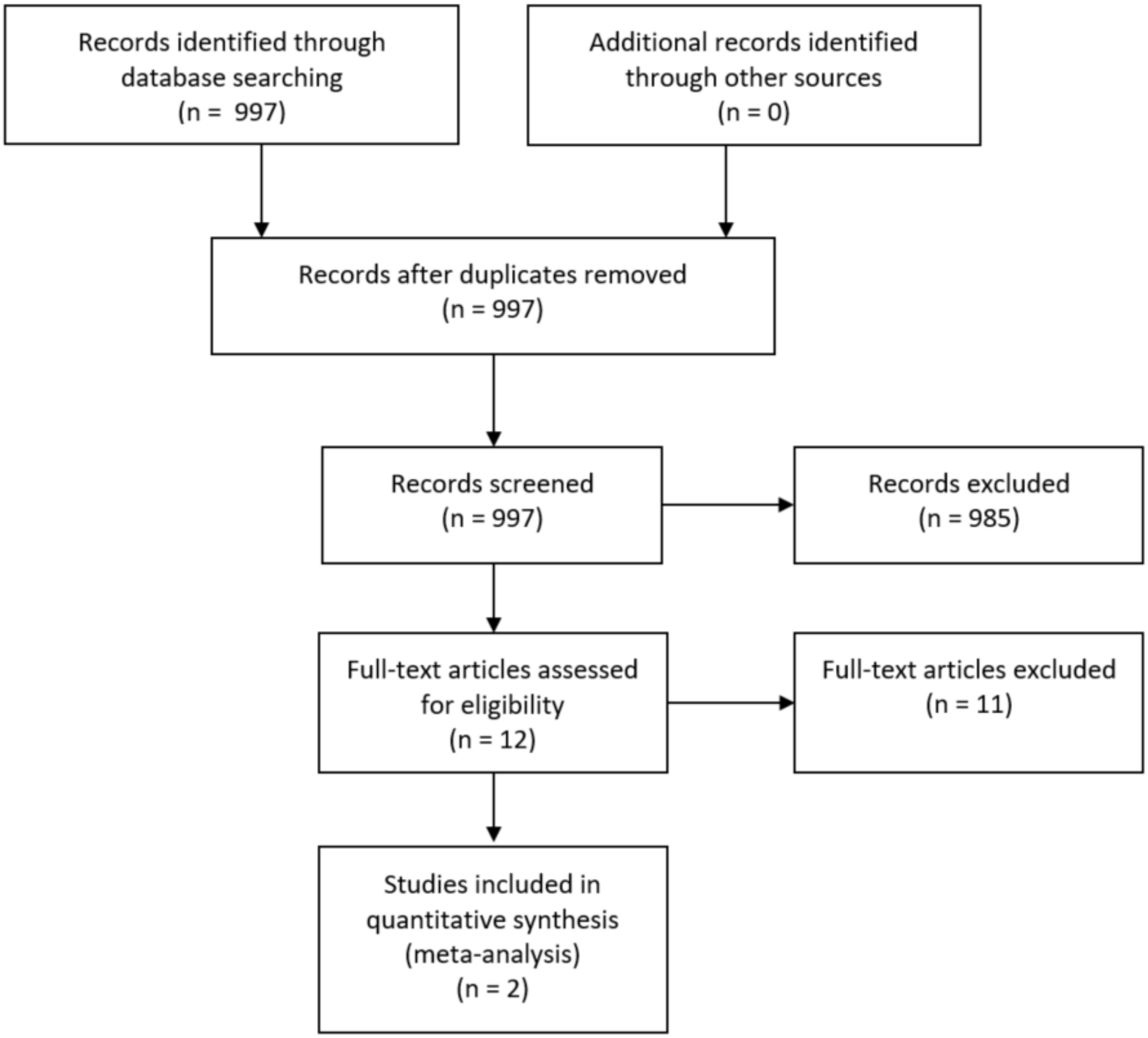

Modelling TB incidence

Incidence inputs were from two different sources: (1) incidence from notification data for countries with a four- or five-star rating on their cause of death data[6] as a proxy for the quality of health-related administrative data systems, and (2) estimated incidence for countries with a less than four-star rating.

We used age- and sex-specific notifications (all new and relapse cases combined) in our analysis for countries with a 4 to 5-star rating. Starting from 2013, notified new cases and relapse cases are aggregated and reported together, and we used the data as they were reported. Prior to 2013, notification data were available by case type (new pulmonary smear-positive, new pulmonary smear-negative, and new extra-pulmonary) and there were missing age data, especially for younger age groups in some countries. We imputed the missing age groups for the three forms of TB notifications. Smear-positive age-specific notifications were inflated with the proportion smear-unknown and relapsed cases only reported at the country-year level. Some countries reported only pulmonary smear-positive cases for selected years. Missing smear-negative and extrapulmonary cases were predicted from the adjusted smear-positive cases using a seemingly unrelated regression. All three types of notifications were added together to represent TB-all-form incidence for countries with a four- or five-star rating.

To generate incidence estimates for locations with a less than four-star rating, we used the Meta-Regression with Bayesian Priors, Regularization, and Trimming (MR-BRT) model[17] as the primary analytical engine to predict MI ratios. In the model, we used age and sex dummies and super-region fixed effects with MI ratios (logit transformed) from locations with a 4- or 5-star rating on causes of death as input data. The HAQ index[18] was used as a covariate and anchoring the lower end of the HAQ index scale with a data point from a cohort study in the

1960s reporting that 49.2% of 126 untreated new pulmonary TB cases were dead at the end of the five-year follow-up period.[19] Our model was then used to predict age-sex-specific MI ratios for all locations and years.

We then used the MI ratios and cause-specific mortality estimates to compute the incidence input for DisMod-MR 2.1 for locations with a less than four-star rating. Finally, we computed the age-sex-specific incidence of TB among the latent TB-infected population, using TB incidence as the numerator and our estimated risk-weighted latent TB infection prevalence as the denominator. Our final incidence estimate that is consistent with prevalence data and CSMR estimates was then generated using DisMod-MR 2.1.

Modelling TB prevalence

Data from prevalence surveys reporting on pulmonary smear-positive TB and bacteriologically positive TB were included. Because incidence data are for all forms of TB, we adjusted prevalence surveys to account for extrapulmonary cases. We ran a spatiotemporal Gaussian process regression to predict location-year-age-sex-specific proportions of extrapulmonary TB among all TB cases using data on the three forms of TB from the incidence data above. We then computed the extrapulmonary inflation factor as 1+(proportion of extrapulmonary TB /(1- proportion of extrapulmonary TB)), and applied it to data from prevalence surveys.

In GBD 2019, we used the MR-BRT model to derive adjustment factors for studies where the case definition was smear-positive TB rather than bacteriologically positive TB (reference). For the adjustment, we identified all prevalence surveys that provided comparisons of smear-positive TB and bacteriologically positive TB from the same sample. Overall, 16 prevalence surveys from Cambodia, China, Ethiopia, Gambia, India, Myanmar, South Korea,

Philippines, Rwanda, and Vietnam were included as inputs in the MR-BRT model.[20–35] The model also contained covariates for sex and age to reflect gradients across demographics. In GBD 2019 we also computed an adjustment factor to adjust studies that used symptoms only as a screening method compared to studies using both symptoms and chest X-ray during screening (reference). To derive the adjustment factor, we ran a MR-BRT model where data from six studies[25,36–40] comparing prevalence between using symptoms only as opposed to symptoms and chest X-ray in the same population as input.

Finally, we computed the prevalence of TB among the TB-infected population, using TB prevalence as the numerator and our estimated risk-weighted LTBI prevalence as the denominator. We included two location-level covariates, namely, age-standardized adult underweight prevalence and log-transformed age-standardized Summary Exposure Variable (SEV) scalar for TB (a summary variable of the exposure levels of TB risk factors weighted by relative risk) to help inform variation of TB prevalence over year and geography.

eTable 2. Crosswalk adjustment factors for Tuberculosis prevalence surveys

Reference or alternative case definition	Gamma	Beta Coefficient, Log (95% CI)	Adjustment factor*
Bacteriologically positive	0.17	---	---
Smear positive		-0.39 (-0.58 to -0.22)	0.67
Symptoms and chest X-ray	0.01	---	---
Symptoms only		-0.38 (-0.50 to -0.25)	0.68

* *Adjustment factor is the transformed Beta coefficient in normal space, and can be interpreted as the factor by which the alternative case definition is adjusted to reflect what it would have been if measured as the reference*.

Modelling TB remission and excess mortality

In GBD 2019 we computed TB duration based on a systematic review of studies during the pre-chemotherapy era finding that duration from onset to cure or death is 3 years.[41] To anchor the lowest end of TB duration we assumed a duration of 6 months based on treatment regimens. We then linearly interpolated between 6 months and 3 years across the HAQ index to

compute TB duration for every country-year. The predicted duration values are shown in eTable 3. We converted duration into remission by taking the inverse (e.g. Remission = 1/duration). Using HAQ-based remission and estimated MI ratios, we computed excess mortality rate (EMR) with the following computation: EMR = MI*Remission (formula derived from Prevalence=Incidence*Duration).

eTable 3. Predicted duration (in years) with 95% uncertainty intervals as a function of the health care access and quality index in 2019 for both sexes, all-ages

**Location**	**Health care access and quality index**	**Duration (years)**
Afghanistan	26.91<br>(25.42 to 28.44)	2.31<br>(2.27 to 2.35)
Albania	78.01<br>(76.81 to 79.24)	1.01<br>(0.98 to 1.04)
Algeria	65.96<br>(64.36 to 67.47)	1.32<br>(1.28 to 1.36)
American Samoa	52.50<br>(51.04 to 54.04)	1.66<br>(1.62 to 1.70)
Andorra	95.80<br>(95.14 to 96.40)	0.55<br>(0.54 to 0.57)
Angola	30.31<br>(28.87 to 31.86)	2.23<br>(2.19 to 2.26)
Antigua and Barbuda	65.24<br>(63.72 to 66.63)	1.33<br>(1.30 to 1.37)
Argentina	66.96<br>(65.55 to 68.33)	1.29<br>(1.25 to 1.33)
Armenia	69.88<br>(68.37 to 71.31)	1.22<br>(1.18 to 1.25)
Australia	95.11<br>(94.48 to 95.76)	0.57<br>(0.55 to 0.59)
Austria	93.68<br>(92.91 to 94.37)	0.61<br>(0.59 to 0.63)
Azerbaijan	59.78<br>(58.12 to 61.31)	1.47<br>(1.43 to 1.52)
Bahrain	74.46<br>(72.86 to 75.97)	1.10<br>(1.06 to 1.14)
Bangladesh	46.14<br>(44.72 to 47.71)	1.82<br>(1.78 to 1.86)
Barbados	66.99<br>(65.39 to 68.44)	1.29<br>(1.25 to 1.33)

Belarus	80.10 (78.56 to 81.64)	0.95 (0.91 to 0.99)
Belgium	93.51 (92.72 to 94.24)	0.61 (0.59 to 0.63)
Belize	54.38 (52.91 to 56.00)	1.61 (1.57 to 1.65)
Benin	31.24 (29.94 to 32.72)	2.20 (2.16 to 2.24)
Bermuda	83.59 (82.49 to 84.61)	0.86 (0.84 to 0.89)
Bhutan	49.65 (48.10 to 51.18)	1.73 (1.69 to 1.77)
Bolivia	46.64 (45.07 to 48.23)	1.81 (1.77 to 1.85)
Bosnia and Herzegovina	74.43 (73.12 to 75.70)	1.10 (1.07 to 1.13)
Botswana	50.78 (49.14 to 52.31)	1.70 (1.66 to 1.74)
Brazil	61.25 (59.88 to 62.78)	1.44 (1.40 to 1.47)
Brunei	68.31 (66.92 to 69.71)	1.26 (1.22 to 1.29)
Bulgaria	73.83 (72.56 to 75.00)	1.11 (1.08 to 1.15)
Burkina Faso	30.57 (29.01 to 32.03)	2.22 (2.18 to 2.26)
Burundi	25.60 (24.33 to 26.87)	2.35 (2.31 to 2.38)
Cambodia	44.39 (42.79 to 45.94)	1.87 (1.83 to 1.91)
Cameroon	35.47 (33.91 to 36.98)	2.09 (2.06 to 2.13)
Canada	94.50 (93.75 to 95.21)	0.59 (0.57 to 0.61)
Cape Verde	56.28 (54.69 to 57.91)	1.56 (1.52 to 1.60)
Central African Republic	12.78 (11.83 to 13.79)	2.67 (2.65 to 2.70)
Chad	23.19 (21.82 to 24.64)	2.41 (2.37 to 2.44)
Chile	77.68 (76.48 to 78.87)	1.02 (0.99 to 1.05)
China	79.84 (78.62 to 81.04)	0.96 (0.93 to 0.99)

Colombia	69.30 (67.88 to 70.79)	1.23 (1.19 to 1.27)
Comoros	31.85 (30.58 to 33.24)	2.19 (2.15 to 2.22)
Congo (Brazzaville)	32.52 (31.07 to 34.00)	2.17 (2.13 to 2.21)
Cook Islands	70.03 (62.21 to 76.89)	1.21 (1.04 to 1.41)
Costa Rica	73.57 (72.12 to 74.86)	1.12 (1.09 to 1.16)
Croatia	88.42 (87.43 to 89.36)	0.74 (0.72 to 0.77)
Cuba	74.42 (73.09 to 75.72)	1.10 (1.07 to 1.13)
Cyprus	93.15 (92.37 to 93.85)	0.62 (0.60 to 0.64)
Czech Republic	87.58 (86.61 to 88.47)	0.76 (0.74 to 0.79)
Côte d'Ivoire	30.55 (29.07 to 32.03)	2.22 (2.18 to 2.26)
DR Congo	26.18 (24.84 to 27.59)	2.33 (2.30 to 2.37)
Denmark	92.62 (91.84 to 93.40)	0.63 (0.61 to 0.65)
Djibouti	36.36 (34.71 to 37.95)	2.07 (2.03 to 2.11)
Dominica	53.14 (51.59 to 54.69)	1.64 (1.60 to 1.68)
Dominican Republic	54.62 (53.06 to 56.18)	1.60 (1.57 to 1.64)
Ecuador	59.51 (57.97 to 60.99)	1.48 (1.44 to 1.52)
Egypt	57.01 (55.37 to 58.70)	1.54 (1.50 to 1.59)
El Salvador	61.96 (60.52 to 63.58)	1.42 (1.38 to 1.45)
Equatorial Guinea	42.43 (40.84 to 44.02)	1.92 (1.88 to 1.96)
Eritrea	25.43 (24.13 to 26.76)	2.35 (2.32 to 2.38)
Estonia	84.12 (82.73 to 85.45)	0.85 (0.82 to 0.89)
Ethiopia	31.93 (30.39 to 33.31)	2.18 (2.15 to 2.22)

Federated States of Micronesia	43.10<br>(41.55 to 44.61)	1.90<br>(1.86 to 1.94)
Fiji	44.71<br>(43.24 to 46.27)	1.86<br>(1.82 to 1.90)
Finland	94.36<br>(93.65 to 95.04)	0.59<br>(0.57 to 0.61)
France	94.18<br>(93.45 to 94.85)	0.59<br>(0.58 to 0.61)
Gabon	40.97<br>(39.35 to 42.52)	1.95<br>(1.91 to 1.99)
Georgia	63.82<br>(62.19 to 65.27)	1.37<br>(1.33 to 1.41)
Georgia	83.03<br>(81.90 to 84.13)	1.37<br>(1.33 to 1.41)
Germany	92.60<br>(91.85 to 93.36)	0.63<br>(0.62 to 0.65)
Ghana	39.45<br>(37.91 to 41.01)	1.99<br>(1.95 to 2.03)
Greece	91.63<br>(90.76 to 92.39)	0.66<br>(0.64 to 0.68)
Greenland	63.55<br>(62.13 to 64.95)	1.38<br>(1.34 to 1.41)
Grenada	57.79<br>(56.21 to 59.33)	1.52<br>(1.48 to 1.56)
Guam	62.22<br>(60.82 to 63.72)	1.41<br>(1.37 to 1.45)
Guatemala	49.47<br>(47.77 to 51.03)	1.74<br>(1.70 to 1.78)
Guinea	25.41<br>(24.11 to 26.73)	2.35<br>(2.32 to 2.38)
Guinea-Bissau	24.30<br>(23.04 to 25.60)	2.38<br>(2.35 to 2.41)
Guyana	46.49<br>(44.85 to 47.99)	1.81<br>(1.77 to 1.85)
Haiti	28.67<br>(27.20 to 30.15)	2.27<br>(2.23 to 2.31)
Honduras	49.23<br>(47.65 to 50.84)	1.74<br>(1.70 to 1.78)
Hungary	82.45<br>(81.35 to 83.55)	0.89<br>(0.87 to 0.92)
Iceland	96.72<br>(96.14 to 97.25)	0.53<br>(0.52 to 0.54)
India	40.41<br>(38.93 to 41.91)	1.97<br>(1.93 to 2.01)

Indonesia	44.74<br>(43.25 to 46.36)	1.86<br>(1.82 to 1.90)
Iran	70.90<br>(69.32 to 72.46)	1.19<br>(1.15 to 1.23)
Iraq	61.85<br>(60.16 to 63.45)	1.42<br>(1.38 to 1.46)
Ireland	96.63<br>(96.06 to 97.15)	0.53<br>(0.52 to 0.55)
Israel	88.54<br>(87.61 to 89.44)	0.74<br>(0.72 to 0.76)
Italy	96.13<br>(95.51 to 96.67)	0.54<br>(0.53 to 0.56)
Jamaica	61.23<br>(59.68 to 62.67)	1.44<br>(1.40 to 1.48)
Japan	93.87<br>(93.15 to 94.56)	0.60<br>(0.58 to 0.62)
Jordan	70.80<br>(69.28 to 72.39)	1.19<br>(1.15 to 1.23)
Kazakhstan	67.47<br>(66.02 to 68.94)	1.28<br>(1.24 to 1.31)
Kenya	34.26<br>(32.70 to 35.72)	2.12<br>(2.09 to 2.16)
Kiribati	28.18<br>(26.83 to 29.62)	2.28<br>(2.24 to 2.31)
Kuwait	82.52<br>(81.20 to 83.87)	0.89<br>(0.86 to 0.93)
Kyrgyzstan	59.78<br>(58.22 to 61.24)	1.47<br>(1.44 to 1.51)
Laos	36.55<br>(35.04 to 38.10)	2.07<br>(2.03 to 2.10)
Latvia	77.25<br>(75.72 to 78.74)	1.03<br>(0.99 to 1.07)
Lebanon	81.12<br>(79.84 to 82.35)	0.93<br>(0.90 to 0.96)
Lesotho	29.68<br>(28.27 to 31.11)	2.24<br>(2.21 to 2.28)
Liberia	32.30<br>(30.84 to 33.83)	2.17<br>(2.14 to 2.21)
Libya	63.06<br>(61.43 to 64.73)	1.39<br>(1.35 to 1.43)
Lithuania	75.77<br>(74.15 to 77.40)	1.06<br>(1.02 to 1.11)
Luxembourg	95.23<br>(94.63 to 95.85)	0.57<br>(0.55 to 0.58)

Madagascar	26.79<br>(25.43 to 28.18)	2.32<br>(2.28 to 2.35)
Malawi	30.03<br>(28.60 to 31.49)	2.23<br>(2.20 to 2.27)
Malaysia	65.52<br>(64.02 to 66.93)	1.33<br>(1.29 to 1.36)
Maldives	71.81<br>(70.46 to 73.06)	1.17<br>(1.13 to 1.20)
Mali	29.98<br>(28.44 to 31.40)	2.23<br>(2.20 to 2.27)
Malta	91.08<br>(90.21 to 91.86)	0.67<br>(0.65 to 0.70)
Marshall Islands	36.45<br>(35.04 to 37.95)	2.07<br>(2.03 to 2.10)
Mauritania	40.49<br>(38.97 to 42.06)	1.97<br>(1.93 to 2.00)
Mauritius	65.89<br>(64.38 to 67.36)	1.32<br>(1.28 to 1.36)
Mexico	62.98<br>(61.47 to 64.50)	1.39<br>(1.35 to 1.43)
Moldova	67.89<br>(66.06 to 69.69)	1.27<br>(1.22 to 1.31)
Monaco	95.75<br>(94.03 to 97.03)	0.55<br>(0.52 to 0.60)
Mongolia	51.70<br>(50.12 to 53.33)	1.68<br>(1.64 to 1.72)
Montenegro	81.08<br>(79.91 to 82.16)	0.93<br>(0.90 to 0.96)
Morocco	54.85<br>(53.11 to 56.73)	1.60<br>(1.55 to 1.64)
Mozambique	27.18<br>(25.82 to 28.52)	2.31<br>(2.27 to 2.34)
Myanmar	42.60<br>(41.05 to 44.06)	1.91<br>(1.87 to 1.95)
Namibia	44.80<br>(43.11 to 46.40)	1.86<br>(1.81 to 1.90)
Nauru	52.18<br>(43.67 to 60.71)	1.67<br>(1.45 to 1.88)
Nepal	41.52<br>(40.05 to 43.03)	1.94<br>(1.90 to 1.98)
Netherlands	96.78<br>(96.19 to 97.29)	0.53<br>(0.51 to 0.54)
New Zealand	91.78<br>(91.00 to 92.53)	0.66<br>(0.64 to 0.68)

Nicaragua	63.54<br>(61.93 to 64.98)	1.38<br>(1.34 to 1.42)
Niger	26.73<br>(25.37 to 28.12)	2.32<br>(2.28 to 2.35)
Niger	24.30<br>(18.30 to 30.98)	2.32<br>(2.28 to 2.35)
Nigeria	35.15<br>(33.63 to 36.62)	2.10<br>(2.06 to 2.14)
Niue	64.45<br>(56.04 to 72.47)	1.35<br>(1.15 to 1.57)
North Korea	54.63<br>(53.05 to 56.20)	1.60<br>(1.56 to 1.65)
North Macedonia	75.42<br>(74.14 to 76.63)	1.07<br>(1.04 to 1.11)
Northern Mariana Islands	68.68<br>(67.27 to 70.19)	1.25<br>(1.21 to 1.28)
Norway	96.67<br>(96.01 to 97.22)	0.53<br>(0.52 to 0.55)
Oman	76.14<br>(74.70 to 77.65)	1.06<br>(1.02 to 1.09)
Pakistan	35.60<br>(34.12 to 37.12)	2.09<br>(2.05 to 2.13)
Palau	64.43<br>(56.38 to 72.43)	1.35<br>(1.15 to 1.56)
Palestine	63.30<br>(61.68 to 65.00)	1.38<br>(1.34 to 1.42)
Panama	68.29<br>(66.85 to 69.79)	1.26<br>(1.22 to 1.29)
Papua New Guinea	31.46<br>(29.97 to 32.89)	2.20<br>(2.16 to 2.23)
Paraguay	59.43<br>(57.88 to 60.93)	1.48<br>(1.44 to 1.52)
Peru	63.49<br>(62.01 to 64.96)	1.38<br>(1.34 to 1.42)
Philippines	46.50<br>(44.86 to 48.05)	1.81<br>(1.77 to 1.85)
Poland	80.88<br>(79.78 to 81.97)	0.93<br>(0.91 to 0.96)
Portugal	90.54<br>(89.70 to 91.33)	0.69<br>(0.67 to 0.71)
Puerto Rico	79.54<br>(78.30 to 80.73)	0.97<br>(0.94 to 1.00)
Qatar	81.24<br>(79.89 to 82.53)	0.92<br>(0.89 to 0.96)

Romania	76.79<br>(75.56 to 78.08)	1.04<br>(1.01 to 1.07)
Russia	75.56<br>(73.90 to 77.05)	1.07<br>(1.03 to 1.11)
Rwanda	34.51<br>(33.12 to 35.99)	2.12<br>(2.08 to 2.15)
Saint Kitts and Nevis	67.52<br>(59.52 to 74.87)	1.28<br>(1.09 to 1.48)
Saint Lucia	60.52<br>(59.05 to 62.14)	1.45<br>(1.41 to 1.49)
Saint Vincent and the Grenadines	54.16<br>(52.62 to 55.72)	1.62<br>(1.58 to 1.66)
Samoa	50.49<br>(48.89 to 52.00)	1.71<br>(1.67 to 1.75)
San Marino	95.27<br>(93.63 to 96.64)	0.57<br>(0.53 to 0.61)
Saudi Arabia	74.29<br>(72.78 to 75.76)	1.10<br>(1.06 to 1.14)
Senegal	32.56<br>(31.10 to 34.02)	2.17<br>(2.13 to 2.21)
Serbia	78.48<br>(77.27 to 79.60)	1.00<br>(0.97 to 1.03)
Seychelles	61.64<br>(60.23 to 63.17)	1.43<br>(1.39 to 1.46)
Sierra Leone	30.48<br>(29.10 to 31.83)	2.22<br>(2.19 to 2.26)
Singapore	90.72<br>(89.87 to 91.47)	0.68<br>(0.66 to 0.70)
Slovakia	82.78<br>(81.67 to 83.90)	0.89<br>(0.86 to 0.91)
Slovenia	91.63<br>(90.82 to 92.46)	0.66<br>(0.64 to 0.68)
Solomon Islands	39.58<br>(38.09 to 41.08)	1.99<br>(1.95 to 2.03)
Somalia	16.12<br>(15.10 to 17.19)	2.59<br>(2.56 to 2.61)
South Africa	46.94<br>(45.38 to 48.51)	1.80<br>(1.76 to 1.84)
South Korea	91.29<br>(90.43 to 92.04)	0.67<br>(0.65 to 0.69)
South Sudan	21.71<br>(20.47 to 23.03)	2.45<br>(2.41 to 2.48)
Spain	95.66<br>(95.01 to 96.26)	0.56<br>(0.54 to 0.57)

Sri Lanka	69.68<br>(68.24 to 70.99)	1.22<br>(1.19 to 1.26)
Sudan	47.20<br>(45.34 to 48.82)	1.79<br>(1.75 to 1.84)
Suriname	50.37<br>(48.71 to 52.05)	1.71<br>(1.67 to 1.76)
Sweden	95.86<br>(95.20 to 96.44)	0.55<br>(0.54 to 0.57)
Switzerland	96.17<br>(95.52 to 96.74)	0.54<br>(0.53 to 0.56)
Syria	65.33<br>(63.73 to 66.96)	1.33<br>(1.29 to 1.37)
Sao Tome and Principe	42.63<br>(41.10 to 44.12)	1.91<br>(1.87 to 1.95)
Taiwan (province of China)	86.74<br>(85.71 to 87.72)	0.78<br>(0.76 to 0.81)
Tajikistan	48.26<br>(46.57 to 49.88)	1.77<br>(1.73 to 1.81)
Tanzania	34.09<br>(32.67 to 35.53)	2.13<br>(2.09 to 2.17)
Thailand	69.32<br>(67.88 to 70.78)	1.23<br>(1.19 to 1.27)
The Bahamas	59.52<br>(58.02 to 61.05)	1.48<br>(1.44 to 1.52)
The Gambia	33.21<br>(31.67 to 34.69)	2.15<br>(2.11 to 2.19)
Timor-Leste	39.93<br>(38.40 to 41.50)	1.98<br>(1.94 to 2.02)
Togo	33.92<br>(32.57 to 35.36)	2.13<br>(2.10 to 2.17)
Tokelau	54.48<br>(46.05 to 62.65)	1.61<br>(1.40 to 1.82)
Tonga	48.96<br>(47.47 to 50.51)	1.75<br>(1.71 to 1.79)
Trinidad and Tobago	58.77<br>(57.26 to 60.31)	1.50<br>(1.46 to 1.54)
Tunisia	73.17<br>(71.65 to 74.56)	1.13<br>(1.10 to 1.17)
Turkey	73.45<br>(72.05 to 74.92)	1.12<br>(1.09 to 1.16)
Turkmenistan	58.69<br>(57.14 to 60.26)	1.50<br>(1.46 to 1.54)
Tuvalu	45.90<br>(37.13 to 54.39)	1.83<br>(1.61 to 2.05)

UK	92.28 (91.53 to 93.02)	0.64 (0.62 to 0.66)
USA	86.88 (85.82 to 87.85)	0.78 (0.76 to 0.81)
Uganda	33.44 (31.96 to 34.77)	2.15 (2.11 to 2.18)
Ukraine	72.81 (71.27 to 74.36)	1.14 (1.10 to 1.18)
United Arab Emirates	61.21 (59.51 to 62.72)	1.44 (1.40 to 1.48)
Uruguay	70.74 (69.44 to 72.01)	1.19 (1.16 to 1.23)
Uzbekistan	59.48 (57.84 to 61.00)	1.48 (1.44 to 1.52)
Vanuatu	32.83 (31.54 to 34.20)	2.16 (2.13 to 2.19)
Venezuela	64.82 (63.34 to 66.35)	1.34 (1.31 to 1.38)
Vietnam	63.04 (61.60 to 64.43)	1.39 (1.35 to 1.43)
Virgin Islands	65.15 (63.52 to 66.59)	1.34 (1.30 to 1.38)
Yemen	42.92 (41.21 to 44.58)	1.90 (1.86 to 1.95)
Zambia	35.49 (33.97 to 37.04)	2.09 (2.05 to 2.13)
Zimbabwe	30.80 (29.30 to 32.31)	2.21 (2.17 to 2.25)
eSwatini	36.20 (34.68 to 37.71)	2.08 (2.04 to 2.11)

DisMod-MR 2.1

DisMod MR 2.1 description

In GBD 2019, no substantial changes were made to DisMod-MR 2.1. The sequence of estimation occurs at five levels: global, super-region, region, country and, where applicable, subnational location. The super-region priors are generated at the global level with mixed-effects, nonlinear regression using all available data; the super-region fit, in turn, informs the region fit, and so on down the cascade. Subnational estimation was informed by the country fit

and country covariates, plus an adjustment based on the average of the residuals between the subnational location's available data and it's prior. This mimicked the impact of a random effect on estimates between subnationals. At each level of the cascade, the DisMod-MR 2.1 enforces consistency between all parameters. Analysts have the choice to branch the cascade in terms of time and sex at different levels depending on data density. We used the default option to model TB, which is to branch by sex after the global fit but to retain all years of data until the lowest level in the cascade.

The coefficients for country covariates were re-estimated at each level of the cascade. For a given location, country coefficients were calculated using both data and prior information available for that location. In GBD 2019, we generated model fits for the years 1990, 1995, 2000, 2005, 2010, 2015, 2017, and 2019, and log-linearly interpolated estimates for the intervening years. The 95% uncertainty intervals were computed based on 1000 draws from the posterior distribution of the model using the 2.5th and 97.5th percentiles of the ordered 1000 values.

DisMod-MR 2.1 likelihood estimation

Analysts have the choice of using a Gaussian, log-Gaussian, Laplace or Log-Laplace likelihood function in DisMod-MR 2.1. We used the default log-Gaussian equation for the data likelihood, which is:

$$-log\left[p\left(y_j|\Phi\right)\right] = \log\left(\sqrt{2\pi}\right) + \log\left(\delta_j + s_j\right) + \frac{1}{2}\left(\frac{\log\left(a_j + \eta_j\right) - \log\left(m_j + \eta_j\right)}{\delta_j + s_j}\right)^2$$

where, y_j is a 'measurement value' (i.e., data point); Φ denotes all model random variables; η_j is the offset value, eta, for a particular 'integrand' (prevalence, incidence, remission, excess

mortality rate, cause-specific mortality rate) and a_j is the adjusted measurement for data point j, defined by:

$$a_j = e^{(-u_j - c_j)} y_j$$

where u_j is the total 'area effect' (i.e., the sum of the random effects at three levels of the cascade: super-region, region and country) and c_j is the total covariate effect (i.e., the mean combined fixed effects for sex, study level and country level covariates), defined by:

$$c_j = \sum_{k=0}^{K[I(j)]-1} \beta_{I(j),k} \hat{X}_{k,j}$$

with standard deviation

$$s_j = \sum_{l=0}^{L[I(j)]-1} \zeta_{I(j),l} \hat{Z}_{k,j}$$

where k denotes the mean value of each data point in relation to a covariate (also called x-covariate); I(j) denotes a data point for a particular integrand, j; $\beta_{I(j),k}$ is the multiplier of the kth x-covariate for the ith integrand; $\hat{X}_{k,j}$ is the covariate value corresponding to the data point j for covariate k; l denotes the standard deviation of each data point in relation to a covariate (also called z-covariate); $\zeta_{I(j),k}$ is the multiplier of the lth z-covariate for the ith integrand; and δ_j is the standard deviation for adjusted measurement j, defined by:

$$\delta_j = log\left[y_j + e^{(-u_j - c_j)}\eta_j + c_j\right] - log\left[y_j + e^{(-u_j - c_j)}\eta_j\right]$$

Where m_j denotes the model for the jth measurement, not counting effects or measurement noise and defined by:

$$m_j = \frac{1}{B(j) - A(j)} \int_{A(j)}^{B(j)} I_j(\mathrm{a})\, \mathrm{da}$$

where A(j) is the lower bound of the age range for a data point; B(j) is the upper bound of the age range for a data point; and I_j denotes the function of age corresponding to the integrand for data point j.

Internally consistent modelling in DisMod-MR 2.1

For each location, we included the following as input in the DisMod model: case notifications for locations with a four- or five-star rating, predicted MI-ratio-based incidence for locations with a less than four-star rating, prevalence survey data where available, predicted excess mortality estimates, HAQ-based remission, and CSMR (TB and HIV-TB combined) by age and sex. DisMod then triangulated all these inputs to generate internally consistent estimates. Final incidence results for locations with a four- or five-star rating may not be identical to notification incidence due to statistical triangulation in DisMod. Beta coefficients and exponentiated values for covariates from the DisMod model are shown in eTable 3. The output from the DisMod model was for all forms of TB in TB-infected populations, including both HIV-negative and HIV-positive individuals. We computed the incidence and prevalence of TB among the entire population, by multiplying the prevalence of LTBI with the DisMod model estimates.

eTable 4. Beta coefficients and exponentiated values from the DisMod model

Covariate	Parameter	Beta (95% CI)	Exponentiated beta (95% CI)
Sex (male)	Prevalence	0.23 (0.19 to 0.26)	1.26 (1.21 to 1.30)
Sex (male)	Incidence	0.35 (0.35 to 0.35)	1.42 (1.42 to 1.42)
Age-standardized proportion adult underweight	Prevalence	2.08 (1.77 to 2.38)	7.97 (5.90 to 10.86)
Age-standardized SEV scalar (log-transformed)	Prevalence	0.75 (0.75 to 0.76)	2.12 (2.12–2.14)

HIV-TB incidence and prevalence

To distinguish HIV-TB from all forms of TB, we first estimated the proportions of HIV-TB cases among all TB cases using data on the number of TB cases recorded as HIV-positive and the number of TB cases with an HIV test result recorded in the WHO TB notifications register. We ran a mixed effects regression using the adult HIV death rate as a covariate to predict location-year-specific HIV-TB proportions, which were then applied to TB incident and prevalent cases from DisMod, to generate HIV-TB incident and prevalent cases by location and year. These cases were then age-sex split based on the age-sex pattern of estimated HIV prevalence by location-year to generate location-year-age-sex-specific HIV-TB incident and prevalent cases.

eTable 5. GATHER checklist of information that should be included in reports of global health estimates, with description of compliance and location of information for "Global, regional, and national sex differences in the global burden of tuberculosis by HIV status, 1990-2019: results from the Global Burden of Disease Study 2019".

#	GATHER checklist item	Description of compliance	Reference
**Objectives and funding**			
1	Define the indicators, populations, and time periods for which estimates were made.	Narrative provided in paper and methods appendix describing indicators, definitions, and populations	Main text (Introduction, pg. 7) and methods appendix (pg. 3–5, 16)
2	List the funding sources for the work.	Funding sources listed in paper	Main text (Methods; pg. 12)
**Data Inputs**			
For all data inputs from multiple sources that are synthesized as part of the study:			
3	Describe how the data were identified and how the data were accessed.	Narrative description of data seeking methods provided	Main text (Methods; pg. 8, 10) and methods appendix (pg. 6, 16, 20)
4	Specify the inclusion and exclusion criteria. Identify all ad-hoc exclusions.	Narrative about inclusion and exclusion criteria by data type provided	Main text (Methods; pg. 8, 10) and methods appendix (pg. 6, 20, 21)
5	Provide information on all included data sources and their main characteristics. For each data source used, report reference information or contact name/institution, population represented, data collection method, year(s) of data collection, sex and age range, diagnostic criteria or measurement method, and sample size, as relevant.	An interactive, online data source tool that provides metadata for data sources by component, geography, cause, risk, or impairment has been developed	Information provided in the following online data citation tools:<br>Mortality sources:<br>http://ghdx.healthdata.org/gbd-2019/data-input-sources?components=4&causes=297&locations=1<br><br>Non-fatal sources:<br>http://ghdx.healthdata.org/gbd-2019/data-input-sources?components=5&causes=297&locations=1

6	Identify and describe any categories of input data that have potentially important biases (e.g., based on characteristics listed in item 5).	Summary of known biases included in methods appendix	Methods appendix (pg 6, 20–22)
For data inputs that contribute to the analysis but were not synthesized as part of the study:			
7	Describe and give sources for any other data inputs.	Included in online data source tool: http://ghdx.healthdata.org/gbd-2019	Information provided in the following online data citation tools:<br><br>Mortality sources: http://ghdx.healthdata.org/gbd-2019/data-input-sources?components=4&causes=297&locations=1<br><br>Non-fatal sources: http://ghdx.healthdata.org/gbd-2019/data-input-sources?components=5&causes=297&locations=1
For all data inputs:			
8	Provide all data inputs in a file format from which data can be efficiently extracted (e.g., a spreadsheet as opposed to a PDF), including all relevant meta-data listed in item 5. For any data inputs that cannot be shared due to ethical or legal, reasons, such as third-party ownership, provide a contact name or the name of the institution that retains the right to the data.	Downloads of input data available through online tools, including data visualization tools and data query tools: http://ghdx.healthdata.org/gbd-2019; input data not available in tools will be made available upon request	Online data visualization tools (https://vizhub.healthdata.org/gbd-compare/), data query tools (http://ghdx.healthdata.org/gbd-2019/data-input-sources) , and the Global Health Data Exchange (http://ghdx.healthdata.org/gbd-2019)

**Data analysis**			
9	Provide a conceptual overview of the data analysis method. A diagram may be helpful.	Flow diagrams of the overall methodological processes, as well as cause-specific modelling processes, have been provided	Main text (Methods; pg 8) and methods appendix (eFigure 1–4)
10	Provide a detailed description of all steps of the analysis, including mathematical formulae. This description should cover, as relevant, data cleaning, data pre-processing, data adjustments and weighting of data sources, and mathematical or statistical model(s).	Flow diagrams and methodological write-ups	Main text (Methods; pg 11–14, 20–35) and methods appendix (eFigure 1, 3, 4)
11	Describe how candidate models were evaluated and how the final model(s) were selected.	Provided in the methodological write-up	Methods appendix (pg. 12)
12	Provide the results of an evaluation of model performance, if done, as well as the results of any relevant sensitivity analysis.	Provided in the methodological write-up	Methods appendix (pg. 12)
13	Describe methods for calculating uncertainty of the estimates. State which sources of uncertainty were, and were not, accounted for in the uncertainty analysis.	Provided in the methodological write-up	Main text (Methods, pg 12) and methods appendix (20–35)
14	State how analytic or statistical source code used to generate estimates can be accessed.	Access statement provided	Code is provided in an online tool, http://ghdx.healthdata.org/gbd-2019/code
**Results and Discussion**			
15	Provide published estimates in a file format from which data can be efficiently extracted.	Results are available through online data visualization tools, the Global Health Data Exchange, and the online data query tool: http://ghdx.healthdata.org/gbd-2019	Main text (table 1), methods appendix (etable 3, 6-12), and online data tools (data visualization tools, data query tools, and the Global Health Data Exchange, http://ghdx.healthdata.org/gbd-2019)
16	Report a quantitative measure of the uncertainty of the estimates (e.g. uncertainty intervals).	Uncertainty intervals are provided with all results	Main text (Results, table 1), methods appendix (etable 3, 6-12), and online data tools

Results

eFigure 5. Temporal trends of male-to-female ratios of age-standardized tuberculosis mortality rates among (A) HIV-negative individuals and (B) HIV-positive individuals by SDI quintile, 1990–2019

A)

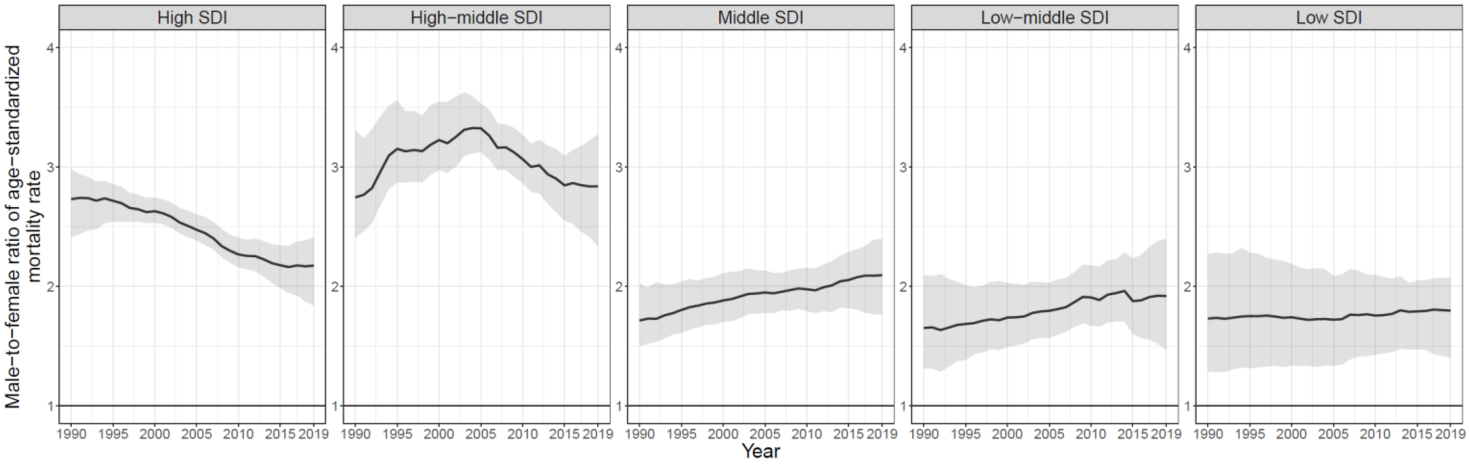

B)

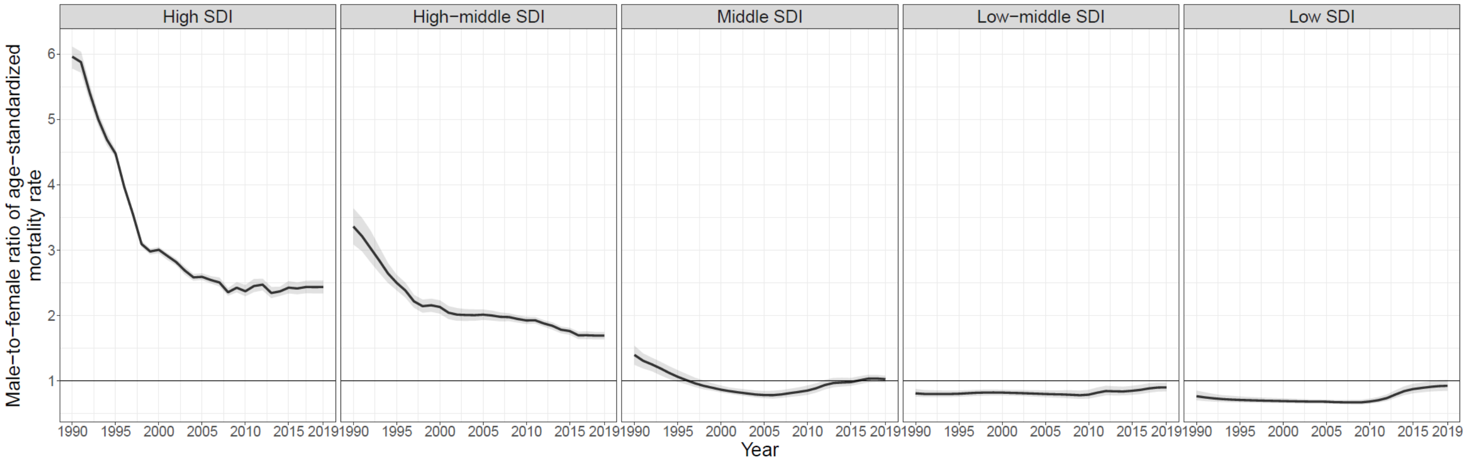

eFigure 6. Age-standardized incidence rate per 100,000 population among HIV-negative individuals in 2019 by geography for males (A) and females (B)

A)

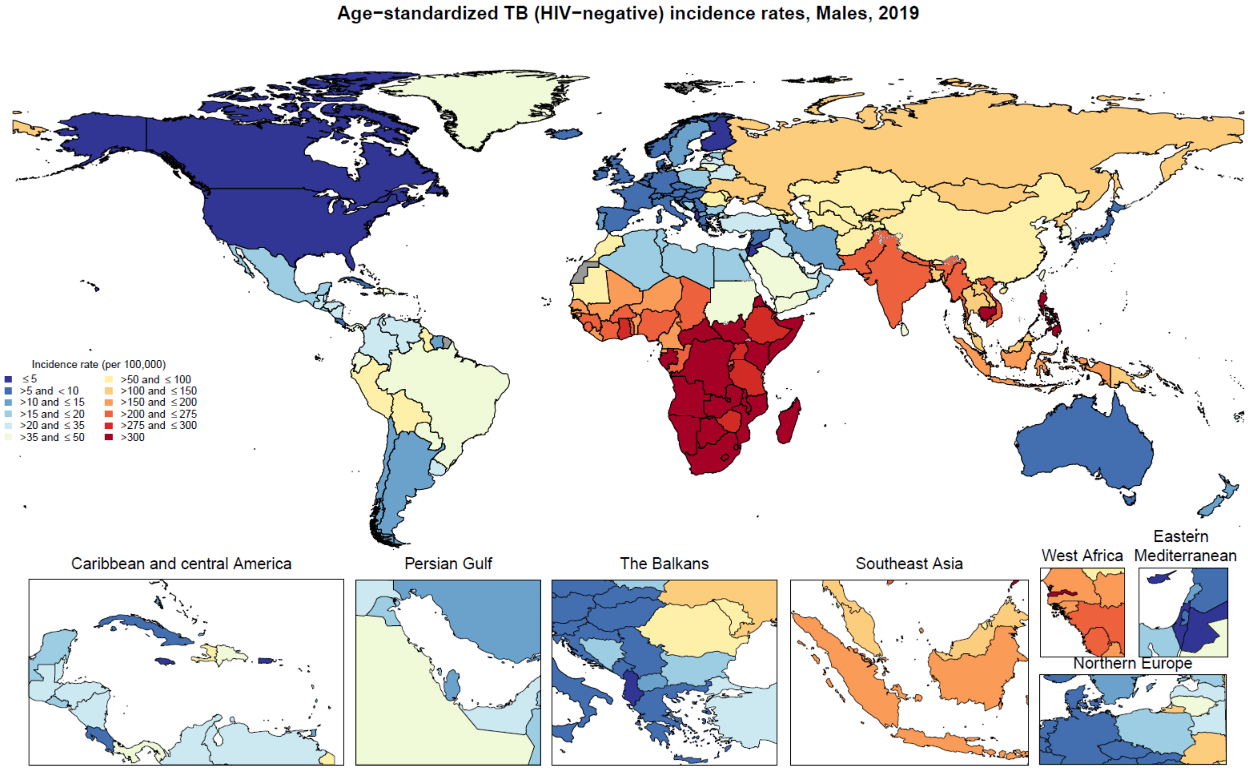

B)

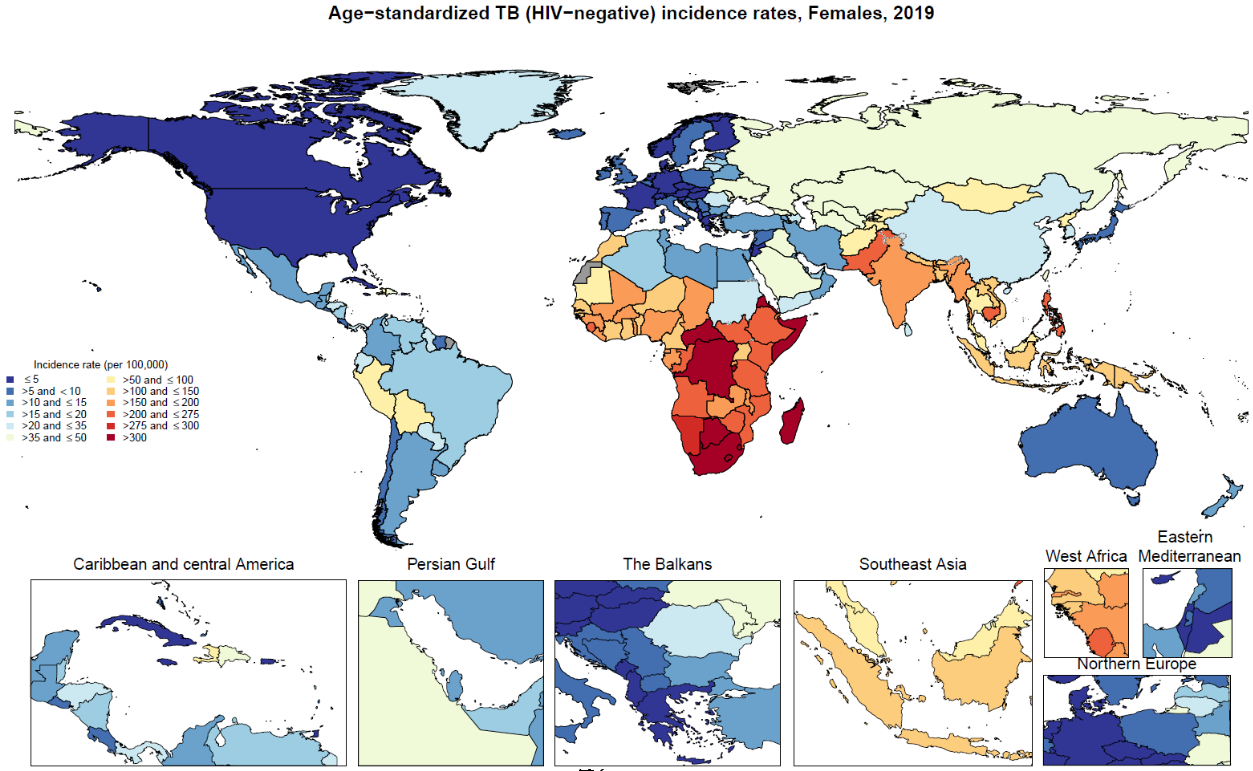

eFigure 7. Age-standardized mortality rate per 100,000 population among HIV-negative individuals in 2019 by geography for males (A) and females (B)

A)

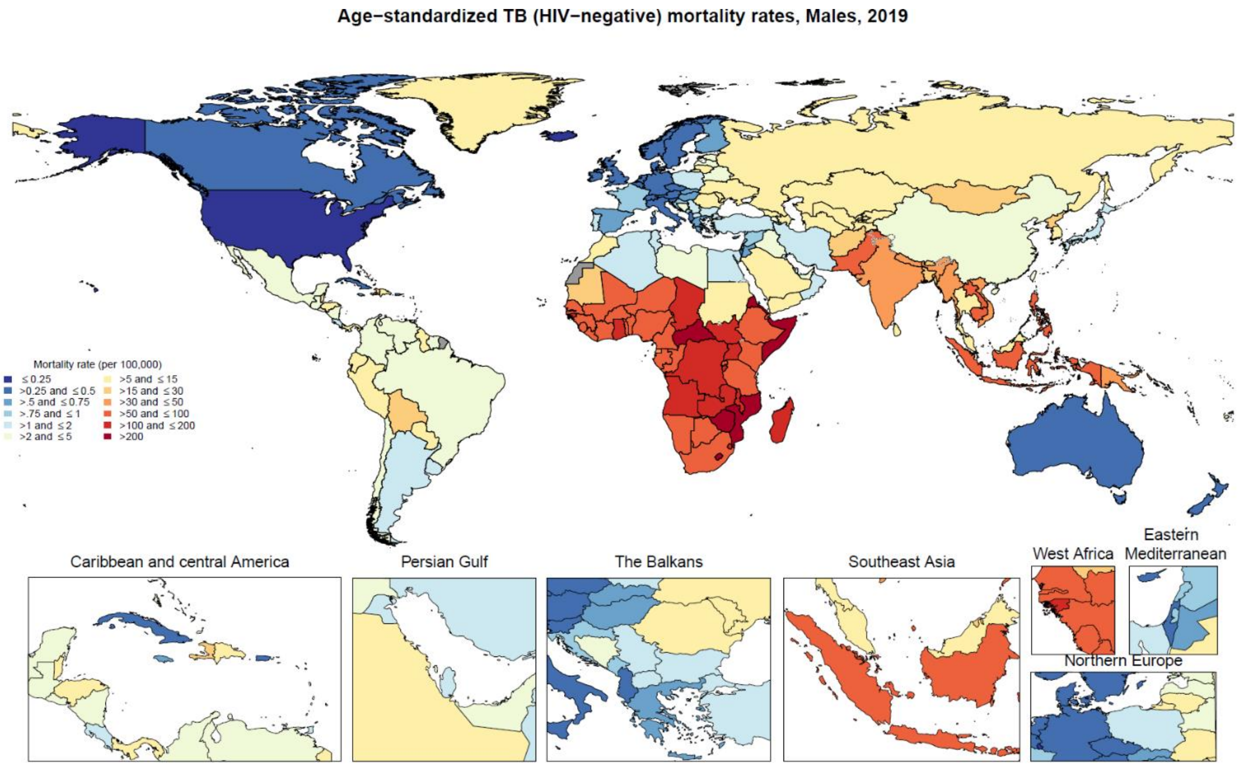

B)

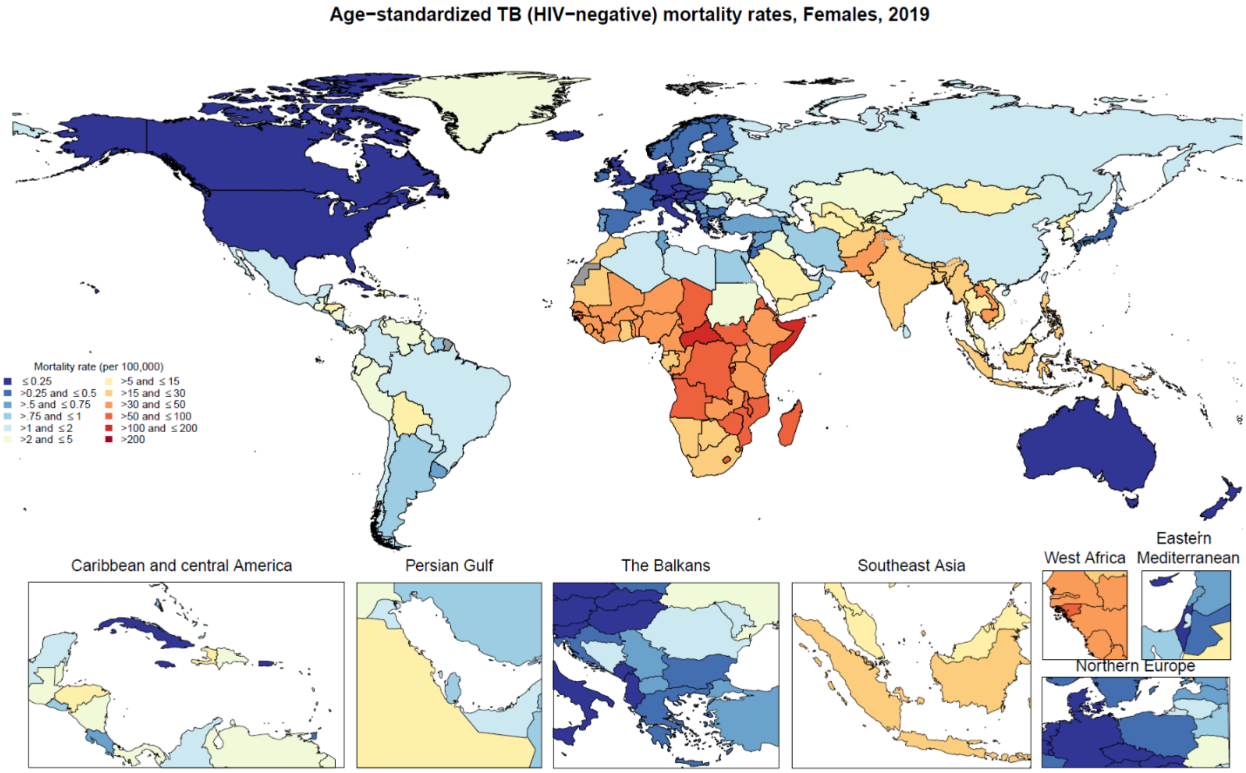

eFigure 8. Age-standardized incidence rate per 100,000 population among HIV-positive individuals in 2019 by geography for males (A) and females (B)

A)

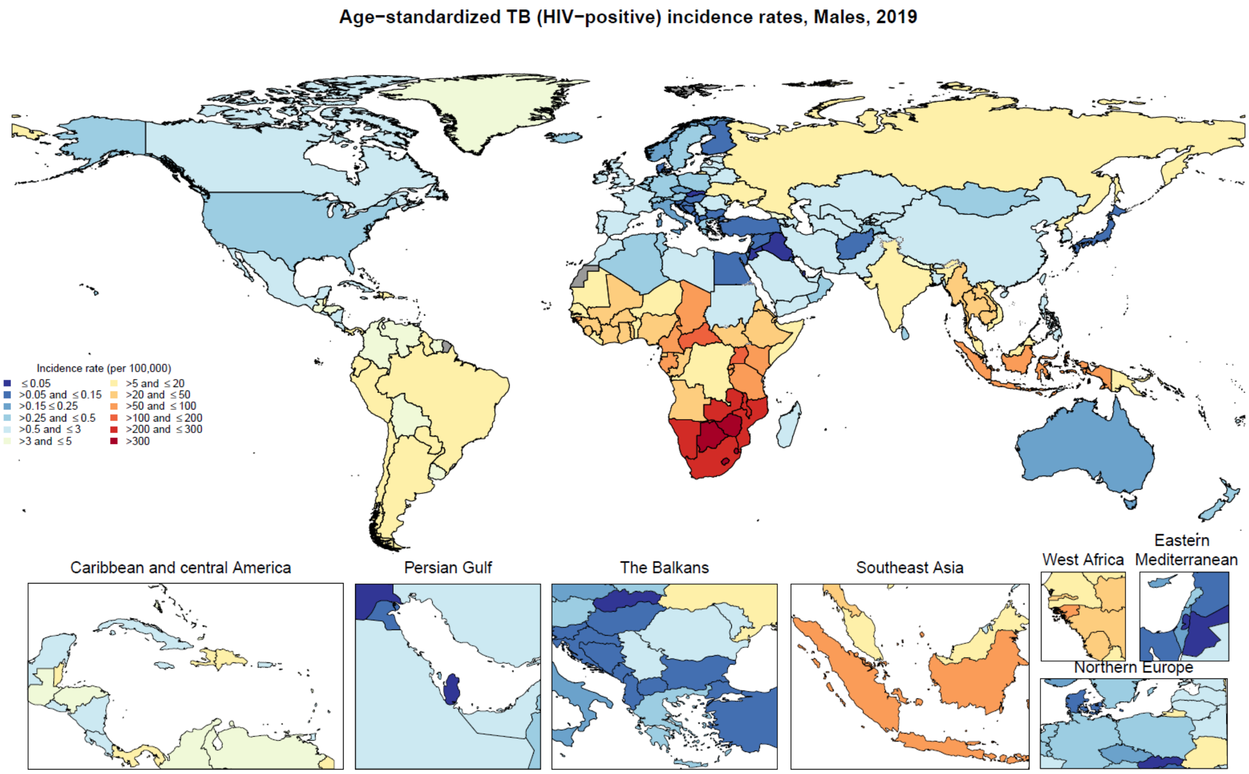

B)

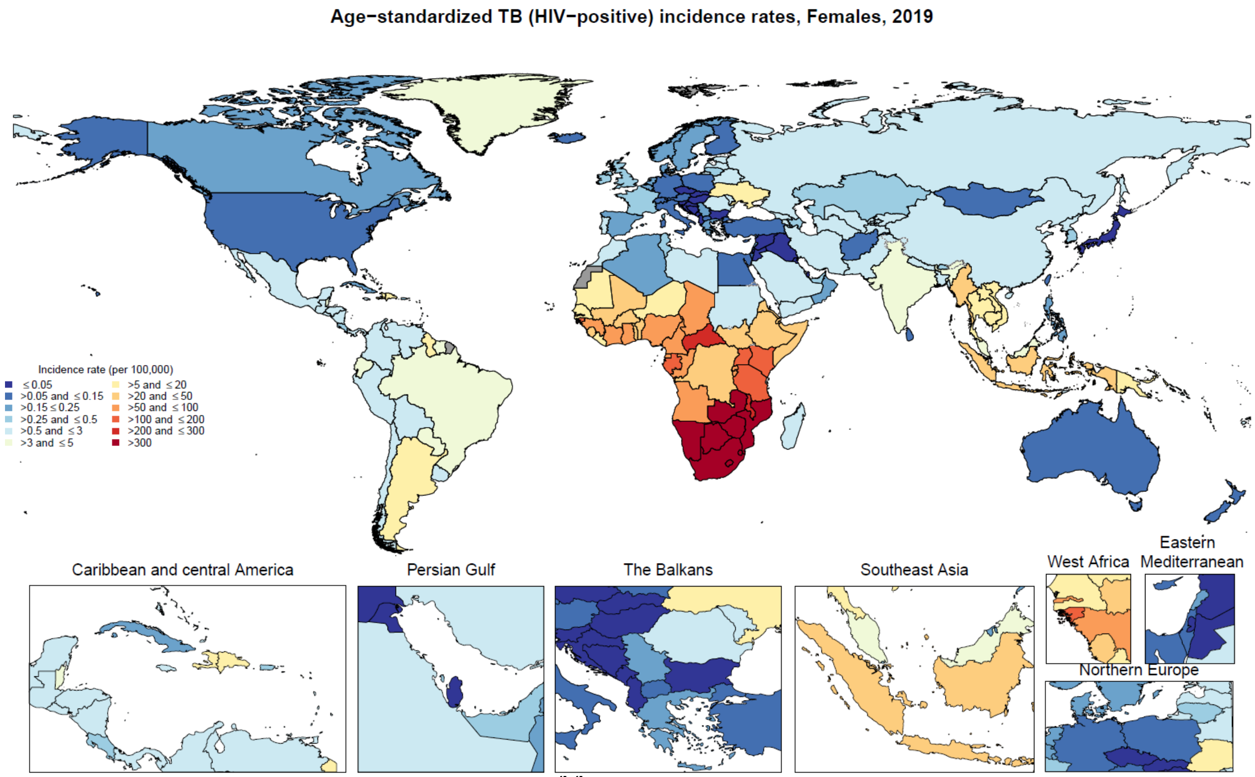

eFigure 9. Age-standardized mortality rate per 100,000 population among HIV-positive individuals in 2019 by geography for males (A) and females (B)

A)

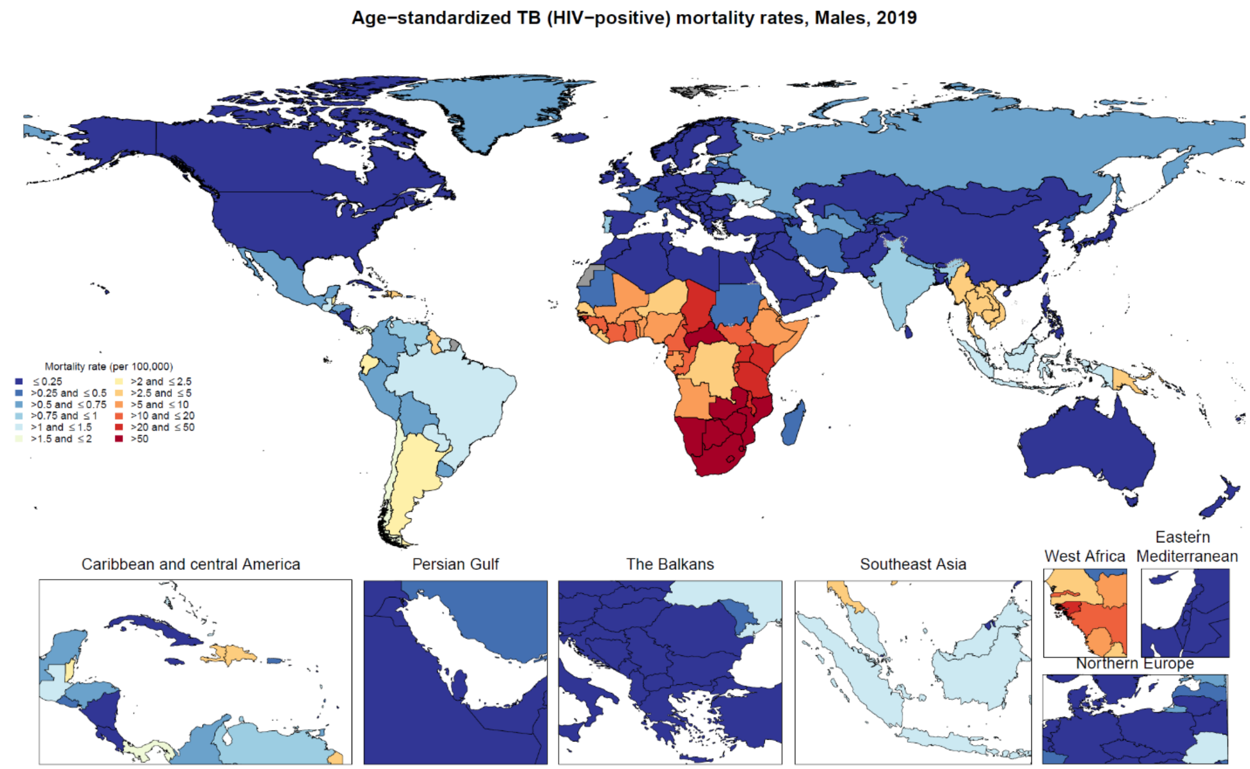

B)

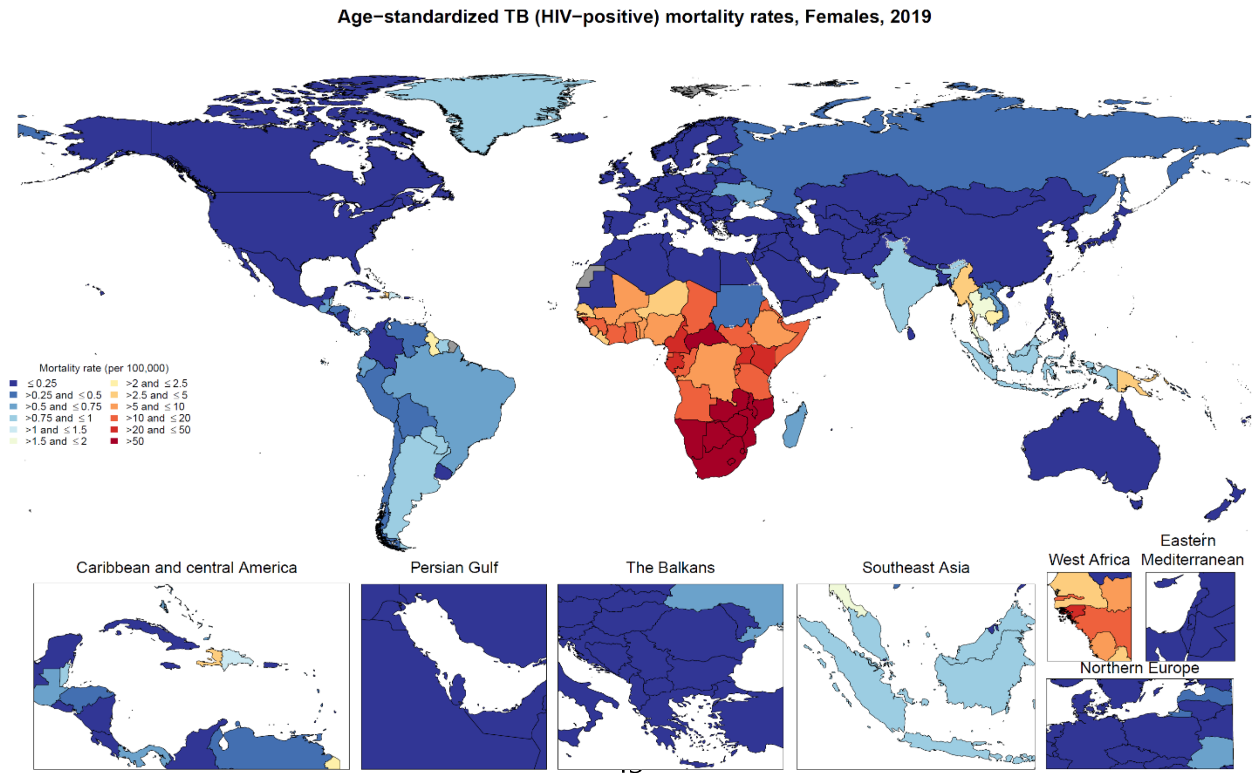

eFigure 10. Global age-sex distribution of tuberculosis incidence (A) and deaths (B) in HIV-positive individuals in 2019

A)

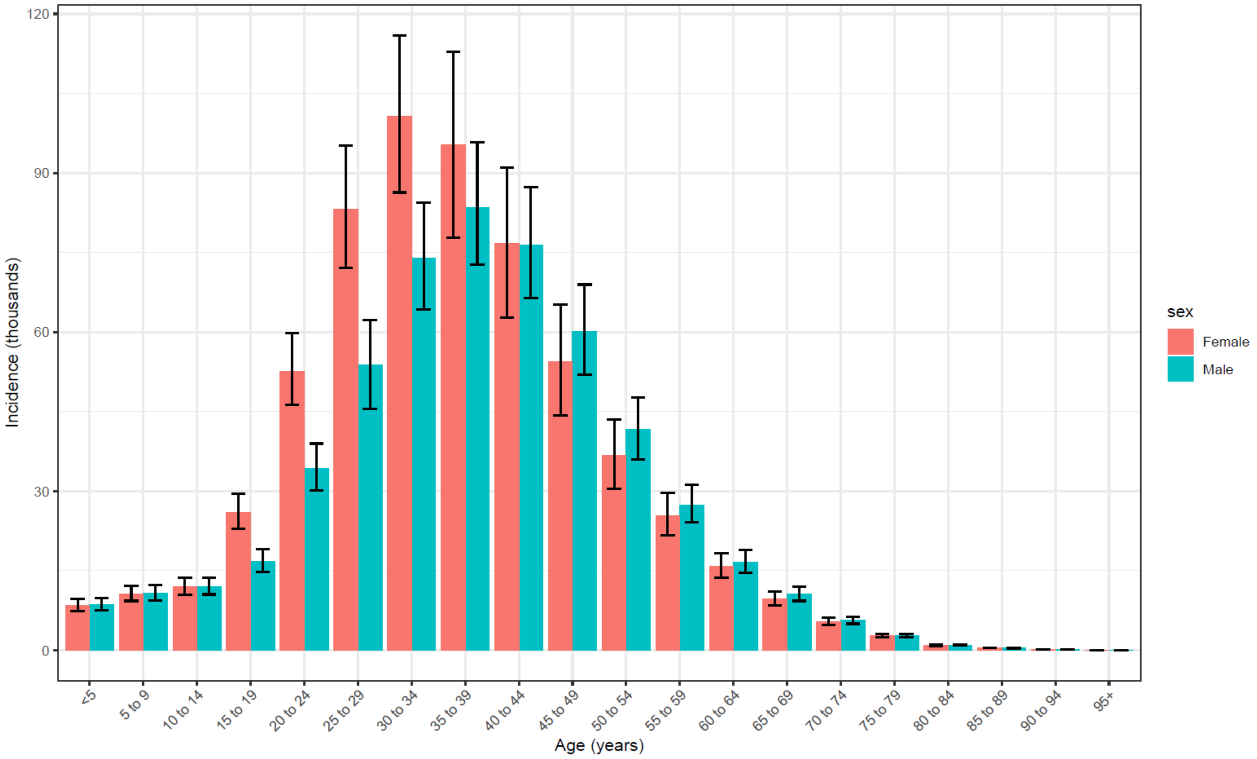

B)

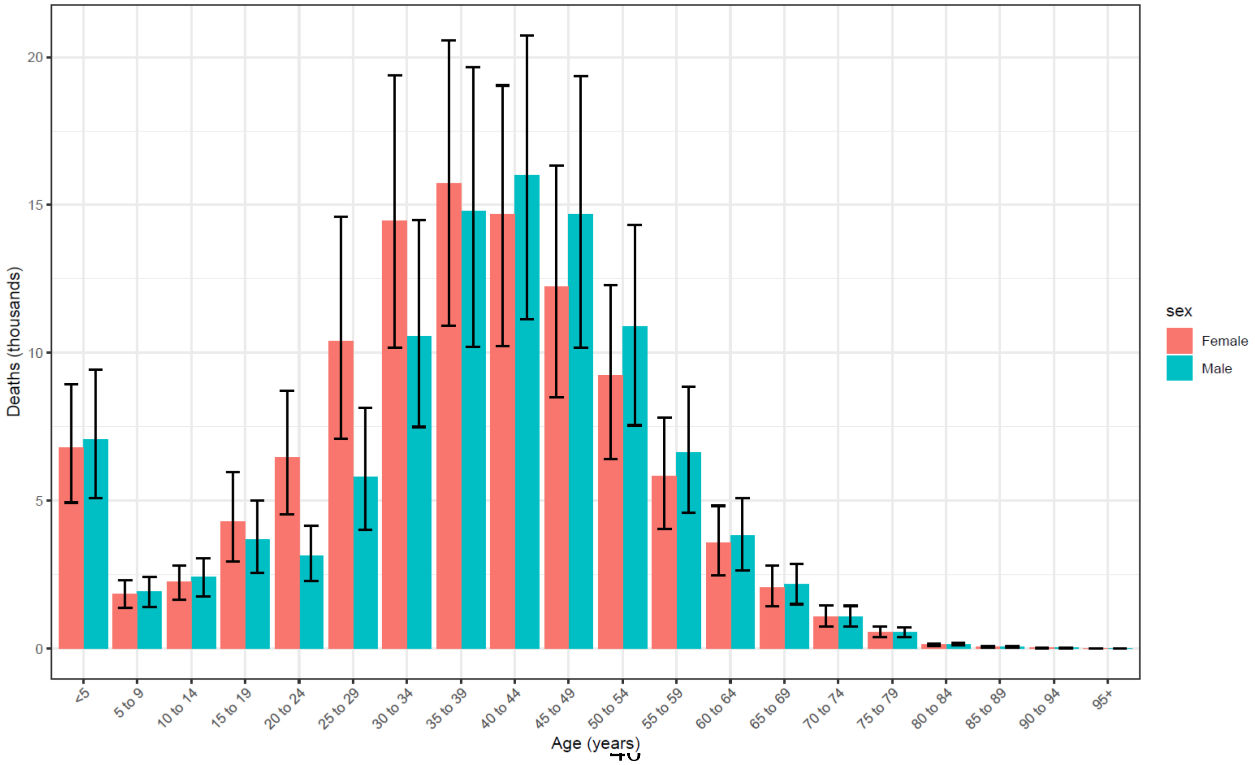

eFigure 11. All-age population attributable fractions of tuberculosis deaths due to drug use, intimate partner violence, and unsafe sex among HIV-positive men and women by GBD regions in 2019

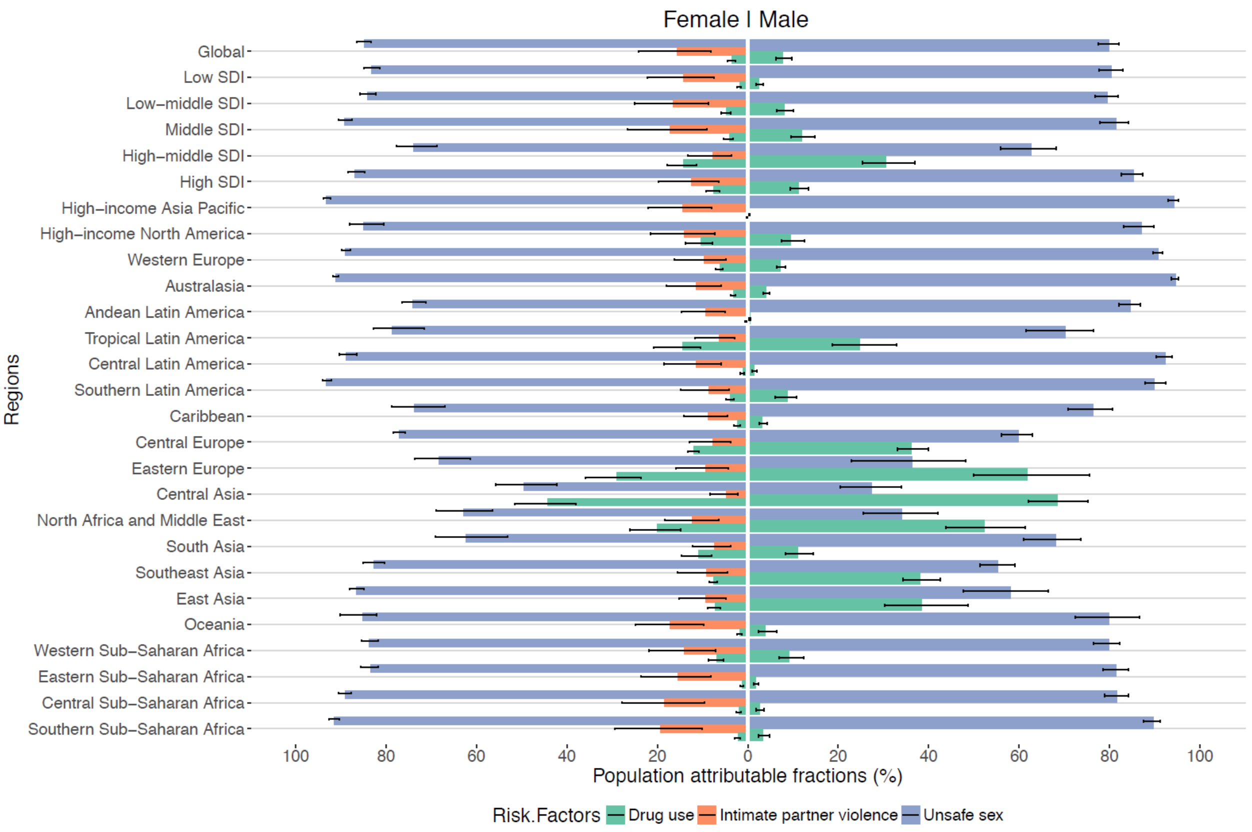

eTable 6. Tuberculosis cases and age-standardized rates of incidence and mortality per 100,000 population with 95% uncertainty intervals among HIV-negative males and females for 204 countries and territories in 2019

	Incidence				Mortality			
	Female		Male		Female		Male	
Location	Cases	Rate (Per 100,000)	Cases	Rate (Per 100,000)	Deaths	Rate (Per 100,000)	Deaths	Rate (Per 100,000)
Afghanistan	14,400 (12,000–17,200)	98·2 (84·2–115)	9,330 (7,910–11,000)	72·3 (62·8–83·3)	1,810 (718–2,610)	20·4 (6·49–29·6)	1,820 (1,260–2,550)	23·5 (16·3–32·4)
Albania	40·3 (34·2–47)	2·45 (2·09–2·87)	55·5 (47·2–65·8)	3·44 (2·96–4·05)	4·1 (3·01–5·57)	0·191 (0·141–0·258)	6·95 (5·2–9·26)	0·376 (0·286–0·492)
Algeria	3,650 (3,110–4,310)	18·4 (15·8–21·6)	3,270 (2,780–3,840)	16·1 (13·8–18·8)	196 (152–266)	1·41 (1·1–1·94)	249 (189–318)	1·64 (1·26–2·08)
American Samoa	3·99 (3·38–4·71)	14·3 (12·2–16·7)	3·56 (3·06–4·14)	13·6 (11·8–15·9)	0·27 (0·208–0·342)	1·12 (0·865–1·41)	0·438 (0·358–0·516)	1·94 (1·62–2·28)
Andorra	1·57 (1·33–1·87)	3·73 (3·1–4·5)	1·48 (1·25–1·79)	3·17 (2·65–3·82)	0·13 (0·0945–0·172)	0·166 (0·121–0·222)	0·138 (0·108–0·171)	0·203 (0·16–0·252)
Angola	32,200 (27,100–37,500)	239 (205–275)	30,800 (26,800–35,100)	301 (268–343)	4,220 (2,930–5,950)	51 (36·3–73·2)	7,530 (5,900–9,340)	115 (93·3–142)
Antigua and Barbuda	9·57 (7·89–11·5)	19·8 (16·4–23·7)	8·39 (6·98–10·1)	18 (15·1–21·5)	0·138 (0·108–0·181)	0·273 (0·214–0·356)	0·234 (0·197–0·278)	0·537 (0·454–0·633)
Argentina	2,500 (2,130–2,960)	10·7 (9·04–12·6)	3,090 (2,620–3,630)	13·8 (11·7–16·1)	271 (238–312)	0·969 (0·853–1·12)	456 (422–494)	1·96 (1·82–2·11)
Armenia	195 (167–227)	11·5 (9·94–13·5)	583 (498–684)	36·4 (31·3–42·9)	11·1 (8·95–13·7)	0·58 (0·469–0·708)	61·7 (50·8–74·3)	3·61 (2·99–4·3)
Australia	671 (578–792)	5·22 (4·48–6·19)	703 (614–813)	5·29 (4·56–6·18)	39·5 (28·5–50·8)	0·161 (0·116–0·205)	55·2 (42·1–73·2)	0·281 (0·215–0·371)
Austria	207 (179–241)	4·66 (3·95–5·5)	325 (279–379)	7·19 (6·12–8·55)	18·8 (16–21·5)	0·173 (0·152–0·194)	31·3 (28·2–34·5)	0·408 (0·368–0·449)
Azerbaijan	1,620 (1,350–1,950)	29·9 (25–35·8)	3,760 (3,110–4,560)	67·2 (56·2–80·5)	134 (99·8–217)	2·75 (2·06–4·42)	502 (393–636)	9·61 (7·67–11·9)

Bahamas	19·1 (16·2–22·6)	9·48 (8·05–11·2)	28·3 (23·7–33·7)	14·6 (12·5–17·2)	1·89 (1·36–2·55)	0·906 (0·649–1·22)	5·47 (4·35–6·81)	3·03 (2·43–3·72)
Bahrain	73·1 (61·1–87·4)	14·1 (11·8–16·5)	170 (141–208)	18·2 (15·6–21·3)	2·85 (2·22–4·1)	1·07 (0·84–1·58)	8·21 (6·39–10·5)	2·18 (1·73–2·68)
Bangladesh	104,000 (88,600–123,000)	131 (112–153)	112,000 (95,300–132,000)	148 (126–173)	10,100 (5,770–18,200)	15·8 (8·98–29·3)	19,000 (15,200–23,600)	28·9 (23–36·1)
Barbados	11·2 (9·3–13·4)	6·98 (5·84–8·34)	9·05 (7·66–10·8)	5·6 (4·7–6·65)	0·581 (0·43–0·752)	0·246 (0·185–0·319)	1·19 (0·96–1·43)	0·603 (0·491–0·723)
Belarus	792 (686–922)	14 (12–16·5)	1,880 (1,580–2,260)	34·5 (29·1–41·2)	64·9 (42·7–93·9)	0·811 (0·537–1·17)	233 (172–310)	3·88 (2·88–5·15)
Belgium	308 (267–359)	5·53 (4·67–6·58)	519 (445–604)	9·23 (7·78–10·9)	34·2 (27·9–39·9)	0·236 (0·197–0·269)	48·3 (42·6–54·2)	0·47 (0·419–0·524)
Belize	36·4 (30·7–43·5)	18·4 (15·8–21·8)	57·6 (48·8–67·7)	31·1 (26·6–36·2)	3·52 (2·86–4·34)	2·12 (1·74–2·6)	11·1 (9·39–13)	6·94 (5·86–8·11)
Benin	6,460 (5,510–7,570)	124 (107–144)	7,440 (6,430–8,640)	175 (151–202)	962 (535–1,540)	30·8 (17–49·4)	1,660 (1,180–2,230)	60·1 (43·6–78·9)
Bermuda	7·97 (6·66–9·6)	22·2 (18·4–26·6)	4·25 (3·49–5·22)	11·8 (9·67–14·2)	0·0375 (0·0278–0·0509)	0·0564 (0·0424–0·0759)	0·0415 (0·0348–0·0491)	0·078 (0·0656–0·092)
Bhutan	399 (334–474)	113 (96·1–133)	326 (273–389)	86·7 (73·6–102)	52·3 (25–118)	19·1 (9·09–43)	54·9 (32·5–101)	18·6 (11·2–33·3)
Bolivia (Plurinational State of)	4,880 (4,150–5,710)	85 (72·8–99·3)	4,970 (4,210–5,810)	90·5 (77·4–105)	682 (459–909)	14·2 (9·59–18·7)	868 (633–1,140)	19·3 (14·2–25·3)
Bosnia and Herzegovina	195 (164–229)	8·37 (7·12–9·75)	370 (308–444)	16·9 (14·4–19·9)	38·4 (30·1–48·4)	1·13 (0·88–1·42)	81·9 (64–103)	3·31 (2·61–4·15)
Botswana	4,340 (3,300–5,570)	331 (257–420)	4,450 (3,560–5,530)	367 (307–445)	258 (154–459)	27·4 (16·6–50·1)	551 (392–783)	72·9 (54–97·4)
Brazil	21,200 (18,000–25,100)	18 (15·2–21·1)	43,400 (36,500–51,700)	38·3 (32·4–45·3)	1,560 (1,390–1,760)	1·25 (1·11–1·4)	3,930 (3,710–4,160)	3·57 (3·37–3·78)
Brunei Darussalam	78·2 (67–91·8)	40·2 (34·8–46·9)	117 (100–137)	63·3 (55·1–73)	7·09 (5·75–8·45)	5·99 (4·87–7·13)	12·2 (10·2–14·8)	19·1 (16·3–22·4)

Bulgaria	419 (364–483)	10·8 (9·4–12·6)	760 (646–897)	17·7 (15·1–20·7)	25·8 (19·8–33)	0·334 (0·257–0·433)	95·7 (74·3–122)	1·7 (1·32–2·16)
Burkina Faso	16,400 (14,100–19,000)	169 (146–194)	17,200 (14,900–20,000)	227 (196–261)	3,060 (2,230–4,050)	47·7 (35·7–60·8)	4,490 (3,480–5,650)	84·8 (68·4–104)
Burundi	19,300 (16,100–22,900)	409 (349–479)	23,200 (19,900–27,100)	551 (480–635)	3,080 (2,030–5,050)	115 (74·7–196)	5,640 (3,910–7,830)	193 (130–268)
Cabo Verde	246 (211–286)	90·5 (77·5–105)	253 (212–303)	96·3 (82·6–114)	28·8 (19·3–54·8)	11·5 (7·62–21·9)	31·6 (24·9–40·1)	14·9 (12–18·4)
Cambodia	21,200 (18,200–24,500)	261 (225–300)	25,900 (22,600–30,100)	380 (333–432)	2,380 (1,460–3,910)	34·9 (21·5–57·5)	3,660 (2,520–4,820)	72·9 (51·2–94·1)
Cameroon	16,300 (13,600–19,500)	131 (111–153)	18,700 (16,000–22,000)	178 (153–208)	2,340 (1,060–3,610)	32·9 (13·9–50)	4,210 (2,710–5,820)	61·9 (39·9–83·3)
Canada	845 (734–974)	4·11 (3·51–4·8)	940 (817–1,090)	4·55 (3·94–5·28)	62·7 (51·9–72·3)	0·158 (0·133–0·181)	86·3 (74·8–99·6)	0·28 (0·243–0·321)
Central African Republic	8,780 (7,480–10,300)	332 (292–375)	13,900 (12,400–15,500)	709 (643–781)	3,190 (2,040–4,590)	190 (118–275)	5,450 (3,970–7,280)	405 (312–511)
Chad	12,500 (10,700–14,700)	192 (165–222)	12,900 (11,100–14,900)	234 (204–269)	2,750 (2,030–3,570)	71·4 (52·6–93·6)	4,240 (3,270–5,440)	109 (84·9–141)
Chile	924 (803–1,070)	8·87 (7·67–10·3)	1,160 (989–1,350)	11·5 (9·93–13·4)	179 (150–209)	1·35 (1·14–1·57)	277 (254–303)	2·65 (2·43–2·89)
China	243,000 (214,000–274,000)	30·6 (27–34·3)	476,000 (424,000–536,000)	56·1 (50·1–62·6)	9,990 (7,960–13,300)	1·05 (0·844–1·39)	26,600 (21,300–32,500)	3·15 (2·58–3·78)
Colombia	3,710 (3,240–4,220)	14 (12·2–15·9)	5,830 (5,040–6,780)	23·9 (20·7–27·7)	310 (227–436)	1·11 (0·808–1·56)	598 (457–771)	2·5 (1·92–3·23)
Comoros	856 (723–1,020)	258 (220–305)	873 (738–1,040)	285 (245–336)	137 (87·4–194)	51·2 (33·5–73·4)	192 (137–249)	80·5 (59·1–102)
Congo	5,540 (4,680–6,480)	230 (198–266)	4,970 (4,280–5,690)	236 (207–266)	774 (386–1,140)	49·1 (24·9–70·7)	1,190 (824–1,530)	81 (57·4–102)
Cook Islands	1·86 (1·58–2·18)	18·4 (15·7–21·6)	1·72 (1·45–2·03)	17·3 (14·6–20·4)	0·106 (0·0813–0·139)	0·869 (0·664–1·14)	0·214 (0·175–0·258)	1·85 (1·51–2·23)

Costa Rica	157 (134–182)	5·93 (5·06–6·85)	229 (198–267)	9·51 (8·25–11)	16·4 (11·5–23·3)	0·592 (0·416–0·841)	30·3 (23·2–39·3)	1·29 (0·98–1·66)
Croatia	168 (145–195)	5·7 (4·9–6·61)	241 (206–284)	8·56 (7·43–9·96)	19·7 (15·1–25)	0·36 (0·276–0·457)	33·7 (26·2–42·7)	0·963 (0·755–1·23)
Cuba	205 (176–241)	3·38 (2·88–4·01)	465 (398–548)	6·89 (5·92–8·04)	10·2 (7·94–12·9)	0·1 (0·0775–0·127)	32·1 (25·5–40·1)	0·37 (0·295–0·461)
Cyprus	13·9 (11·9–16·2)	1·88 (1·61–2·21)	25·8 (21·8–30·8)	3·59 (3·03–4·24)	2·07 (1·69–2·53)	0·212 (0·174–0·256)	2·81 (2·38–3·32)	0·365 (0·313–0·43)
Czechia	195 (168–227)	2·99 (2·55–3·48)	338 (288–398)	5·09 (4·36–5·98)	18·3 (14·8–22·6)	0·144 (0·116–0·178)	36·5 (29·1–44·7)	0·432 (0·346–0·524)
Côte d'Ivoire	14,400 (12,100–17,000)	132 (113–154)	20,900 (18,000–24,400)	204 (177–235)	2,140 (1,020–3,290)	36·1 (15·9–56·7)	4,940 (3,390–6,560)	73·3 (52·6–94·8)
Democratic People's Republic of Korea	12,300 (10,800–14,000)	83·8 (73·3–95·1)	20,000 (17,600–22,700)	147 (130–165)	1,140 (506–1,580)	6·41 (2·98–8·88)	2,110 (1,540–2,840)	16·3 (12·3–21·3)
Democratic Republic of the Congo	120,000 (103,000–138,000)	312 (274–355)	102,000 (88,700–116,000)	320 (284–358)	19,400 (12,400–30,900)	80 (51·3–128)	26,800 (19,100–36,700)	135 (96·6–183)
Denmark	115 (99·9–135)	4·03 (3·42–4·77)	164 (140–191)	5·46 (4·61–6·43)	10·9 (9·08–12·9)	0·165 (0·14–0·195)	16·1 (14·1–18·4)	0·317 (0·279–0·359)
Djibouti	962 (794–1,160)	191 (162–223)	1,460 (1,240–1,740)	275 (236–322)	119 (66·2–185)	40·5 (22·9–61·7)	318 (213–450)	84·3 (62·4–114)
Dominica	6·36 (5·42–7·43)	18·1 (15·4–21·3)	11·3 (9·58–13·3)	29·7 (25·2–35·2)	0·729 (0·514–0·939)	1·72 (1·21–2·23)	2·17 (1·71–2·71)	5·41 (4·28–6·72)
Dominican Republic	1,940 (1,610–2,350)	35·1 (29·1–42)	2,660 (2,230–3,160)	48·8 (41·1–57·8)	239 (169–361)	4·6 (3·27–6·92)	512 (381–697)	10·3 (7·68–13·9)
Ecuador	2,430 (2,030–2,850)	27·4 (23·1–32)	3,660 (3,070–4,330)	42·6 (36·2–50)	228 (182–294)	2·87 (2·3–3·66)	506 (397–660)	6·75 (5·31–8·73)
Egypt	5,280 (4,440–6,270)	11·8 (10·1–13·8)	7,190 (6,210–8,380)	15·2 (13·2–17·6)	258 (168–673)	0·779 (0·506–1·99)	633 (460–886)	1·62 (1·18–2·24)
El Salvador	308 (264–359)	8·94 (7·7–10·4)	452 (383–526)	16·4 (13·9–19)	27·9 (20·7–37·3)	0·798 (0·592–1·07)	61·6 (47·1–80·8)	2·36 (1·8–3·11)

Equatorial Guinea	922 (766–1,110)	155 (132–182)	912 (771–1,070)	174 (151–201)	76·7 (34·7–168)	24 (11·2–53)	117 (74·3–168)	44·2 (29·6–60)
Eritrea	9,720 (8,150–11,500)	363 (309–422)	15,600 (13,200–18,300)	631 (547–728)	1,520 (930–2,450)	91·5 (57·1–145)	3,160 (2,100–4,480)	211 (144–288)
Estonia	64·6 (55·6–75·3)	8·71 (7·37–10·3)	129 (110–154)	17·2 (14·6–20·6)	6·46 (5–8·28)	0·524 (0·397–0·678)	17·9 (13·5–23·2)	2·01 (1·52–2·61)
Eswatini	2,130 (1,590–2,760)	314 (240–404)	1,970 (1,630–2,380)	338 (284–405)	150 (88·4–228)	35·2 (20·5–54·6)	487 (344–660)	154 (116–203)
Ethiopia	100,000 (85,200–117,000)	230 (200–264)	112,000 (96,500–129,000)	279 (246–317)	10,300 (8,050–13,500)	44·9 (35·5–59·4)	19,600 (15,800–23,700)	76·5 (61·3–93·1)
Fiji	157 (136–181)	35·5 (30·8–40·8)	118 (103–133)	27·8 (24·6–31)	16·9 (12·7–22)	4·33 (3·27–5·67)	22·8 (17·5–29·4)	6·94 (5·45–8·71)
Finland	113 (98·2–131)	3·82 (3·22–4·53)	147 (128–170)	4·83 (4·13–5·64)	27·4 (21·2–32·8)	0·328 (0·258–0·386)	27·7 (23·4–32·5)	0·506 (0·428–0·592)
France	1,710 (1,490–1,960)	4·86 (4·1–5·73)	2,670 (2,320–3,110)	8·11 (6·86–9·57)	561 (415–704)	0·488 (0·376–0·603)	492 (420–566)	0·761 (0·658–0·866)
Gabon	1,590 (1,310–1,900)	178 (150–212)	2,230 (1,920–2,600)	308 (268–357)	128 (63·2–188)	21·5 (10·8–31·6)	346 (235–446)	66·8 (43·3–83·9)
Gambia	1,570 (1,350–1,860)	178 (154–209)	2,310 (1,980–2,710)	301 (258–350)	239 (137–342)	45·9 (26·1–66·6)	482 (351–618)	95·1 (71·4–120)
Georgia	703 (605–814)	37·1 (31·5–43·7)	1,460 (1,260–1,720)	75·1 (64·6–87·9)	30·4 (24·4–36·9)	1·24 (0·992–1·51)	145 (117–175)	6·82 (5·53–8·23)
Germany	1,730 (1,500–2,000)	4·29 (3·6–5·08)	3,230 (2,770–3,800)	7·9 (6·55–9·55)	188 (159–217)	0·156 (0·136–0·176)	275 (247–302)	0·325 (0·294–0·356)
Ghana	20,700 (17,200–24,800)	138 (118–160)	32,100 (27,400–37,700)	275 (236–323)	2,310 (1,550–3,210)	24·4 (16·5–36·2)	7,920 (6,130–9,820)	105 (81·8–128)
Greece	154 (134–176)	2·54 (2·17–2·97)	296 (257–341)	5·23 (4·45–6·13)	57 (47·1–67·5)	0·357 (0·305–0·412)	80·3 (68·6–90·8)	0·697 (0·607–0·777)
Greenland	9·51 (7·94–11·3)	32·8 (27·8–38·8)	17·2 (14·4–20·6)	49·3 (42–57·8)	0·826 (0·654–1·04)	2·82 (2·25–3·53)	2·19 (1·74–2·64)	7·32 (5·88–8·71)

Grenada	7·11 (5·96–8·52)	13·5 (11·4–16·1)	5·02 (4·19–5·97)	9·08 (7·67–10·7)	0·142 (0·114–0·177)	0·256 (0·207–0·318)	0·431 (0·376–0·489)	0·854 (0·754–0·958)
Guam	27·9 (23·7–32·5)	33 (28·1–38·6)	22·9 (19·4–26·5)	25·3 (21·6–29·3)	1·65 (1·23–2·04)	1·78 (1·34–2·2)	2·06 (1·66–2·54)	2·29 (1·84–2·82)
Guatemala	1,150 (1,010–1,300)	13·9 (12·3–15·6)	1,480 (1,280–1,720)	20·3 (17·8–23·2)	157 (119–213)	2·18 (1·63–2·93)	262 (202–330)	4·37 (3·37–5·53)
Guinea	8,610 (7,270–10,100)	161 (139–186)	8,830 (7,690–10,200)	203 (176–233)	1,640 (1,030–2,430)	49·2 (30·1–72·5)	2,750 (1,990–3,690)	87·4 (63·6–117)
Guinea-Bissau	1,040 (869–1,250)	128 (109–149)	1,190 (1,010–1,400)	190 (164–220)	246 (150–402)	53·9 (32·9–88·9)	470 (350–600)	117 (89·6–146)
Guyana	91·3 (77·1–107)	22·8 (19·4–26·4)	233 (197–276)	62·5 (53·3–73·6)	15 (10·1–21·8)	4·21 (2·88–6·07)	45·6 (35·2–59·3)	14·1 (11–18)
Haiti	5,610 (4,810–6,500)	89 (77·7–101)	3,110 (2,680–3,590)	57·7 (50·6–65·2)	687 (472–1,040)	14 (9·62–21·1)	598 (390–816)	15·1 (9·7–20·6)
Honduras	1,340 (1,160–1,540)	30·3 (26·7–34·5)	1,200 (1,020–1,380)	33·2 (28·6–38)	254 (178–447)	7·57 (5·38–13·2)	304 (246–386)	10·9 (8·97–13·6)
Hungary	267 (230–311)	4·16 (3·58–4·84)	398 (338–473)	6·5 (5·59–7·66)	21·8 (17·4–27·3)	0·17 (0·137–0·212)	39·5 (32·1–48·2)	0·525 (0·428–0·639)
Iceland	9·25 (7·72–11)	5·51 (4·54–6·59)	9·1 (7·56–11)	5·15 (4·26–6·2)	0·721 (0·572–0·878)	0·214 (0·174–0·256)	0·523 (0·449–0·599)	0·2 (0·171–0·229)
India	1,350,000 (1,150,000–1,590,000)	199 (170–232)	1,690,000 (1,440,000–1,980,000)	248 (212–288)	150,000 (114,000–193,000)	25·3 (19·4–33·3)	273,000 (219,000–328,000)	47·5 (38·3–57·2)
Indonesia	139,000 (121,000–158,000)	113 (99·4–127)	193,000 (170,000–219,000)	165 (147–184)	27,600 (22,300–34,100)	26·6 (21·8–32·1)	48,900 (39,400–60,200)	52 (42·8–61·5)
Iran (Islamic Republic of)	4,450 (3,870–5,120)	11·1 (9·57–12·8)	6,000 (5,140–7,050)	13·5 (11·7–15·5)	334 (297–429)	0·998 (0·878–1·29)	484 (448–523)	1·35 (1·24–1·45)
Iraq	6,780 (5,720–8,160)	35·3 (30·2–41·5)	5,870 (5,010–6,870)	33·1 (28·5–38·5)	416 (313–574)	3·28 (2·51–4·52)	526 (395–671)	4·54 (3·57–5·62)
Ireland	126 (109–147)	5·05 (4·26–5·94)	151 (129–177)	5·97 (5·05–7·06)	14·1 (11·4–17)	0·339 (0·274–0·408)	15·8 (12·6–19·9)	0·467 (0·376–0·585)

Israel	125 (107–145)	2·65 (2·26–3·13)	164 (139–193)	3·57 (3·01–4·23)	11·6 (9·57–13·7)	0·169 (0·142–0·199)	14·4 (12·7–16·2)	0·275 (0·243–0·309)
Italy	1,630 (1,380–1,920)	5·63 (4·64–6·81)	2,770 (2,310–3,310)	9·9 (7·96–12·1)	185 (153–210)	0·196 (0·166–0·221)	241 (211–272)	0·374 (0·332–0·422)
Jamaica	39·8 (32·9–48·4)	2·7 (2·25–3·27)	69·4 (59·2–82·2)	4·79 (4·12–5·6)	2·3 (1·73–3·04)	0·143 (0·106–0·191)	8·14 (6·15–10·4)	0·563 (0·425–0·72)
Japan	6,100 (5,100–7,180)	5·21 (4·36–6·19)	9,220 (7,690–10,900)	8·38 (7·09–9·85)	1,560 (1,100–1,850)	0·479 (0·358–0·553)	2,290 (1,920–2,480)	1·22 (1·04–1·31)
Jordan	232 (190–281)	4·86 (4·06–5·74)	249 (209–297)	4·78 (4·05–5·61)	11·5 (9·02–15·4)	0·461 (0·365–0·623)	18 (14·1–22·8)	0·598 (0·467–0·752)
Kazakhstan	4,420 (3,790–5,170)	45·5 (38·9–53·1)	6,490 (5,490–7,670)	72·3 (61·8–84·7)	233 (186–275)	2·3 (1·84–2·72)	536 (443–628)	5·96 (4·95–6·97)
Kenya	50,800 (42,300–60,400)	223 (192–261)	62,800 (53,800–72,800)	320 (280–368)	5,190 (3,500–7,510)	41·7 (27·8–60·7)	11,600 (8,030–15,600)	93·1 (66–123)
Kiribati	126 (110–143)	225 (200–252)	102 (90–115)	218 (194–241)	31·3 (22·7–43·7)	75 (54·8–105)	37·3 (28–49·8)	109 (84–145)
Kuwait	314 (261–380)	14·3 (12·2–16·8)	521 (433–626)	19·6 (16·9–22·9)	10·3 (7·3–14·7)	1·06 (0·746–1·5)	16·1 (12·6–20·1)	1·27 (0·996–1·6)
Kyrgyzstan	2,290 (1,930–2,700)	70·8 (60·5–82·6)	3,090 (2,620–3,650)	105 (89·9–123)	120 (100–141)	3·9 (3·24–4·58)	285 (246–329)	10·4 (9·02–12·1)
Lao People's Democratic Republic	3,740 (3,210–4,380)	116 (101–137)	3,700 (3,150–4,420)	127 (109–150)	771 (470–1,080)	31·5 (19·4–43·8)	1,320 (957–1,710)	60·5 (44·8–77·7)
Latvia	162 (139–190)	15·4 (12·9–18·2)	332 (278–398)	31 (26·1–36·9)	12·4 (9·19–16·5)	0·646 (0·473–0·863)	40·9 (30·5–53·4)	3·06 (2·29–3·99)
Lebanon	356 (303–419)	13·2 (11·2–15·5)	275 (234–322)	11 (9·37–12·8)	19·6 (10·3–57·3)	0·692 (0·367–2·02)	22·3 (14·5–36·7)	0·927 (0·607–1·52)
Lesotho	4,800 (3,480–6,450)	404 (301–532)	4,920 (4,090–5,940)	467 (398–549)	698 (430–1,010)	83·7 (52·6–120)	1,900 (1,420–2,380)	283 (223–350)
Liberia	3,010 (2,570–3,540)	159 (137–183)	3,220 (2,750–3,770)	177 (153–205)	413 (278–772)	37·2 (25·2–70·4)	650 (477–852)	54·3 (41·7–70·2)

Libya	445 (378–531)	13·8 (11·8–16·2)	573 (481–689)	16·3 (14–19·1)	31 (14·8–43·7)	1·23 (0·588–1·73)	52·6 (32·9–70)	2·01 (1·26–2·69)
Lithuania	340 (294–397)	21·4 (18·3–25)	790 (670–943)	49·9 (42·7–59·3)	33·2 (25·4–41·9)	1·31 (1·03–1·66)	119 (93·7–149)	6·37 (5·05–7·98)
Luxembourg	14·3 (12–16·9)	4·85 (3·97–5·81)	23·3 (19·5–27·8)	7·41 (6·18–8·92)	0·889 (0·712–1·09)	0·142 (0·116–0·172)	0·892 (0·756–1·04)	0·2 (0·171–0·234)
Madagascar	35,900 (30,200–42,800)	333 (285–390)	37,300 (31,800–44,000)	381 (330–444)	4,800 (2,880–7,280)	72 (44·9–108)	7,460 (5,500–10,000)	114 (85–151)
Malawi	17,200 (13,600–21,300)	187 (152–226)	22,000 (18,500–26,100)	322 (272–381)	2,430 (1,690–3,600)	54 (38·2–80·4)	5,430 (4,080–7,000)	144 (112–183)
Malaysia	10,800 (9,120–12,600)	70·8 (60·7–82·2)	19,500 (16,400–22,900)	122 (104–143)	632 (494–824)	5·12 (4·04–6·69)	1,360 (1,060–1,720)	10·9 (8·55–13·7)
Maldives	49 (41·4–57·6)	26·8 (23–31)	93·7 (78·7–112)	35 (30·1–40·5)	3·77 (2·99–4·85)	3·06 (2·4–3·99)	10·2 (8·22–12·5)	6·87 (5·54–8·44)
Mali	13,900 (11,900–16,200)	160 (138–185)	11,800 (10,200–13,600)	162 (140–188)	2,490 (1,770–3,810)	44·6 (30·5–77·3)	3,150 (2,350–4,150)	62·8 (48·5–80·3)
Malta	11·4 (9·54–13·8)	5·95 (4·88–7·19)	23·7 (19·5–28·5)	11·6 (9·37–14·1)	0·397 (0·322–0·487)	0·072 (0·0591–0·0875)	0·796 (0·686–0·928)	0·197 (0·17–0·228)
Marshall Islands	30·4 (26·3–34·6)	117 (102–131)	29·1 (25·7–32·8)	113 (102–126)	3·16 (1·11–4·67)	17·1 (5·75–25)	4·41 (3·28–5·88)	22·8 (17·3–29·9)
Mauritania	1,570 (1,330–1,850)	95·6 (82·2–112)	1,160 (995–1,360)	78 (66·8–91·6)	223 (94·7–376)	21·3 (8·92–36·3)	259 (158–382)	24·5 (15·3–35·2)
Mauritius	69·1 (59·7–80·8)	9·59 (8·27–11·2)	86·4 (73·9–101)	11·7 (10·1–13·6)	3·25 (2·43–4·34)	0·377 (0·282–0·502)	11·4 (8·78–14·8)	1·46 (1·12–1·88)
Mexico	8,970 (7,580–10,500)	13·6 (11·6–15·9)	11,100 (9,360–13,100)	18·2 (15·5–21·4)	833 (648–1,080)	1·31 (1·02–1·7)	1,760 (1,440–2,150)	3·1 (2·53–3·77)
Micronesia (Federated States of)	33·6 (28·8–38·7)	68·3 (59·4–77·5)	27·5 (23·6–31·3)	58·5 (51·6–65·9)	4·61 (2·43–6·75)	12·4 (6·5–17·9)	6·84 (4·39–9·22)	19·1 (13·3–25·3)
Monaco	1·79 (1·54–2·08)	8·62 (7·16–10·3)	2·35 (2·02–2·74)	10·7 (9·01–12·7)	0·297 (0·219–0·385)	0·496 (0·363–0·64)	0·565 (0·452–0·685)	1·3 (1·05–1·58)

Mongolia	1,390 (1,150–1,680)	78·6 (65·7–93·6)	1,830 (1,520–2,210)	107 (89·4–127)	109 (81·6–147)	6·74 (5·06–8·93)	262 (200–344)	16·8 (13·1–21·9)
Montenegro	13·4 (11·4–15·7)	3·76 (3·19–4·45)	29·4 (24·9–35·1)	7·91 (6·72–9·42)	0·896 (0·714–1·13)	0·185 (0·15–0·231)	3·39 (2·71–4·17)	0·819 (0·66–1)
Morocco	19,100 (16,000–22,900)	107 (90·5–127)	9,200 (7,750–10,900)	52·2 (44·5–61·7)	2,700 (1,900–6,130)	18·1 (13–41)	1,240 (917–1,560)	9·08 (6·71–11·4)
Mozambique	28,700 (22,100–37,400)	207 (168–250)	41,200 (35,200–48,900)	433 (368–513)	6,350 (4,360–9,410)	88·7 (61·6–132)	14,400 (10,900–18,600)	235 (184–302)
Myanmar	44,600 (39,100–51,000)	156 (138–177)	55,900 (49,600–63,300)	230 (205–258)	5,010 (3,280–7,020)	19·7 (12·9–27·3)	9,300 (7,030–12,000)	46·5 (35·8–58·6)
Namibia	3,820 (2,960–4,870)	277 (218–349)	3,970 (3,320–4,720)	362 (303–427)	231 (154–351)	25 (17–38·1)	600 (442–771)	83·4 (63·8–104)
Nauru	3·22 (2·72–3·8)	66·7 (57·8–76·7)	2·47 (2·08–2·89)	58·4 (51–66·5)	0·225 (0·144–0·33)	7·99 (5·25–11·3)	0·342 (0·25–0·475)	14 (10·6–18)
Nepal	21,300 (18,000–25,300)	141 (120–165)	25,000 (21,400–29,200)	200 (172–232)	2,410 (1,660–3,270)	20·9 (14·2–28·8)	5,080 (3,870–6,310)	49 (37·5–60·7)
Netherlands	339 (291–395)	4·01 (3·35–4·76)	440 (378–515)	5·25 (4·4–6·29)	50·4 (41·7–58·7)	0·238 (0·2–0·275)	48·5 (42·5–54·5)	0·32 (0·28–0·358)
New Zealand	262 (221–313)	11·6 (9·71–14·1)	226 (191–268)	10·3 (8·58–12·5)	8·25 (6·92–9·57)	0·194 (0·164–0·226)	9·67 (8·54–10·7)	0·271 (0·241–0·299)
Nicaragua	601 (511–698)	19·3 (16·6–22·2)	788 (673–911)	28·8 (24·9–33·3)	52·4 (42·4–65·4)	2·07 (1·7–2·57)	90·1 (71·3–113)	4·36 (3·5–5·35)
Niger	12,700 (11,000–14,800)	147 (127–170)	12,900 (11,100–14,900)	178 (155–204)	2,320 (1,680–3,220)	44·3 (32·2–60·9)	3,560 (2,540–4,920)	75 (55–103)
Nigeria	134,000 (116,000–155,000)	155 (136–178)	154,000 (134,000–178,000)	214 (188–246)	17,000 (11,700–25,100)	33·2 (22·2–59·8)	28,300 (21,400–38,300)	57·6 (42·9–77·5)
Niue	0·348 (0·301–0·408)	38·3 (32·8–45·1)	0·319 (0·271–0·377)	34·2 (29·3–39·8)	0·0288 (0·0207–0·0428)	2·56 (1·82–3·81)	0·0416 (0·0327–0·0511)	4·46 (3·52–5·47)
North Macedonia	99·8 (84·7–116)	8·07 (6·87–9·43)	176 (147–210)	13 (11–15·2)	10 (7·67–12·9)	0·679 (0·522–0·864)	27·6 (21·4–34·8)	1·93 (1·51–2·39)

Northern Mariana Islands	10·2 (8·55–12·1)	43·8 (37·4–51·2)	7·91 (6·66–9·38)	31·1 (26·5–36·3)	0·519 (0·411–0·658)	2·3 (1·82–2·9)	0·641 (0·529–0·772)	2·6 (2·2–3·08)
Norway	137 (114–162)	4·98 (4·06–6·03)	160 (132–190)	5·69 (4·64–6·88)	22 (18·3–25)	0·348 (0·297–0·393)	21·6 (19·5–23·6)	0·482 (0·436–0·526)
Oman	203 (168–244)	14·3 (12·1–16·7)	399 (327–490)	15·1 (13·1–17·5)	5·76 (3·92–9·96)	0·916 (0·61–1·63)	12·5 (8·7–15·9)	1·56 (1·16–1·98)
Pakistan	269,000 (229,000–318,000)	261 (225–302)	239,000 (203,000–282,000)	253 (218–296)	26,000 (20,000–33,800)	36·9 (29·1–48·8)	36,800 (25,600–54,200)	55·3 (38·4–81·5)
Palau	4·3 (3·62–5·09)	45·6 (39–53·1)	4·08 (3·4–4·87)	34·9 (29·6–40·9)	0·344 (0·254–0·449)	3·61 (2·66–4·63)	0·467 (0·359–0·607)	4·39 (3·46–5·57)
Palestine	165 (139–198)	8·09 (6·92–9·43)	101 (85·7–121)	5·06 (4·35–5·9)	15·6 (7·95–20)	1·36 (0·769–1·73)	9·04 (7·64–10·8)	0·887 (0·736–1·07)
Panama	511 (440–588)	24·4 (20·9–28)	848 (728–989)	40·5 (34·8–47·1)	72·2 (50·5–104)	3·42 (2·39–4·95)	116 (87·6–153)	5·71 (4·31–7·5)
Papua New Guinea	5,490 (4,760–6,320)	126 (111–142)	4,980 (4,410–5,540)	120 (108–132)	550 (282–882)	18·6 (9·34–29·8)	989 (637–1,410)	32·6 (21–46)
Paraguay	692 (584–823)	20 (17–23·5)	1,390 (1,170–1,650)	41·1 (35·1–48·3)	56·6 (42·9–75·4)	1·86 (1·4–2·47)	185 (140–244)	6·49 (4·88–8·43)
Peru	10,600 (8,940–12,500)	61·2 (51·6–71·3)	15,400 (13,000–18,100)	89·1 (75·8–104)	696 (492–1,010)	4·09 (2·89–5·94)	1,250 (916–1,670)	7·75 (5·68–10·3)
Philippines	122,000 (107,000–140,000)	235 (208–267)	205,000 (181,000–234,000)	416 (369–469)	8,720 (6,580–11,300)	20·5 (15·6–26·2)	20,500 (15,800–26,200)	53·9 (42·1–68·5)
Poland	1,970 (1,670–2,330)	7·62 (6·47–8·98)	4,240 (3,480–5,170)	17 (14·1–20·5)	127 (101–158)	0·318 (0·25–0·397)	431 (346–537)	1·57 (1·26–1·95)
Portugal	545 (471–628)	9·38 (7·96–11·1)	845 (727–984)	14·2 (12·1–16·7)	75·8 (62·4–90·7)	0·514 (0·437–0·604)	148 (134–163)	1·53 (1·39–1·67)
Puerto Rico	48·2 (40·7–57)	2·41 (2–2·94)	63·7 (54·4–75·2)	3·08 (2·62–3·64)	7·61 (5·47–10·4)	0·194 (0·141–0·267)	13 (10·1–16·7)	0·447 (0·347–0·576)
Qatar	85·7 (70·5–104)	13 (11–15·3)	271 (219–334)	12·7 (10·9–14·7)	1·42 (1·06–2·06)	0·964 (0·736–1·47)	7·21 (5·23–9·71)	1·54 (1·17–1·99)

Republic of Korea	11,900 (10,200–13,800)	31·7 (27·2–37)	17,900 (15,200–21,100)	49·2 (42·7–57·3)	1,240 (977–1,500)	2·43 (1·92–2·92)	1,910 (1,670–2,200)	6·08 (5·32–7·02)
Republic of Moldova	732 (632–868)	35·5 (30·6–42·1)	1,780 (1,490–2,150)	82·8 (70·2–99·4)	30·9 (25·6–37·3)	1·25 (1·05–1·5)	154 (129–179)	6·58 (5·54–7·64)
Romania	2,840 (2,430–3,320)	27 (22·8–31·9)	5,970 (5,050–7,090)	51·8 (44·2–60·7)	192 (152–233)	1·37 (1·1–1·68)	749 (602–917)	5·53 (4·44–6·74)
Russian Federation	37,000 (30,600–44,800)	45·8 (37·8–55·9)	81,500 (66,000–101,000)	101 (82·6–124)	1,940 (1,530–2,400)	1·92 (1·52–2·38)	6,040 (4,910–7,390)	6·83 (5·56–8·33)
Rwanda	9,930 (8,220–11,900)	169 (143–198)	12,200 (10,400–14,600)	255 (221–299)	1,480 (1,030–2,190)	41·1 (29·3–61·9)	2,480 (1,740–3,330)	80·6 (58·5–106)
Saint Kitts and Nevis	4·75 (3·96–5·69)	14·8 (12·5–17·7)	8·55 (7·1–10·4)	25·7 (21·5–30·3)	0·2 (0·14–0·272)	0·637 (0·448–0·858)	0·764 (0·544–0·969)	2·65 (2–3·25)
Saint Lucia	9 (7·62–10·7)	9·57 (8·1–11·3)	14·7 (12·4–17·5)	15·2 (12·9–17·9)	1·02 (0·8–1·31)	0·95 (0·742–1·22)	3·24 (2·72–3·86)	3·36 (2·84–3·97)
Saint Vincent and the Grenadines	8·15 (6·82–9·77)	14·2 (11·8–16·9)	10·4 (8·77–12·3)	16·6 (14·1–19·6)	0·402 (0·336–0·49)	0·652 (0·543–0·797)	2·27 (1·93–2·67)	3·53 (3·02–4·12)
Samoa	59 (49·9–68·6)	60·8 (52·5–69·7)	42·6 (36·9–48·8)	45·6 (39·9–51·7)	5·76 (3·83–9·25)	7·54 (5·03–12·1)	7·08 (5·02–10·1)	9·73 (6·95–13·8)
San Marino	1·02 (0·873–1·19)	5·41 (4·54–6·42)	0·498 (0·427–0·589)	2·78 (2·34–3·34)	0·138 (0·0902–0·198)	0·327 (0·215–0·479)	0·073 (0·0494–0·1)	0·236 (0·159–0·324)
Sao Tome and Principe	59·8 (51–70·2)	71·1 (61·1–82·9)	65·5 (55·7–77·6)	83·7 (71·8–99)	6·97 (4·45–13)	12·5 (8·03–23·3)	12·8 (9·81–16·4)	24 (18·7–29·7)
Saudi Arabia	7,220 (5,980–8,780)	48·7 (41·2–57·9)	7,990 (6,600–9,750)	37·9 (32·6–44)	521 (376–864)	6·57 (4·78–10·7)	780 (603–1,020)	7·5 (6·06–9·26)
Senegal	8,980 (7,650–10,500)	147 (127–172)	9,540 (8,260–11,200)	170 (147–198)	1,560 (1,100–2,240)	39·9 (28·3–57·9)	2,170 (1,570–2,740)	54·2 (41–67·4)
Serbia	347 (299–407)	6·17 (5·29–7·22)	547 (459–653)	9·96 (8·41–11·9)	44·7 (34·7–56·3)	0·538 (0·421–0·669)	83·4 (63·6–106)	1·27 (0·983–1·61)
Seychelles	11·8 (10·2–13·7)	22·6 (19·5–26)	19·5 (16·7–22·9)	33·7 (29·2–38·8)	1·16 (0·942–1·44)	2·12 (1·73–2·67)	2·66 (2·22–3·18)	5·85 (4·88–6·95)

Sierra Leone	7,510 (6,390–8,830)	221 (190–257)	7,870 (6,800–9,230)	258 (224–297)	1,060 (690–1,760)	48 (31·9–79·4)	1,450 (1,060–1,960)	65·7 (50·7–86·4)
Singapore	801 (686–943)	23·4 (20–27·5)	1,320 (1,120–1,550)	36·1 (31·1–41·8)	15·4 (12·2–19·5)	0·391 (0·309–0·493)	42·1 (37–47·6)	1·32 (1·14–1·51)
Slovakia	103 (87·1–122)	2·9 (2·46–3·42)	188 (158–225)	5·49 (4·65–6·43)	9·9 (7·49–13·1)	0·184 (0·14–0·244)	21·1 (15·8–27·1)	0·578 (0·441–0·733)
Slovenia	55 (47·2–64)	3·87 (3·29–4·5)	69·5 (59·5–82·5)	5·09 (4·4–5·96)	6·26 (4·78–8·05)	0·217 (0·167–0·278)	6·43 (4·93–8·41)	0·373 (0·287–0·487)
Solomon Islands	120 (102–138)	42·4 (36·6–47·8)	120 (104–136)	46·2 (41–51·9)	15·8 (12·1–20·2)	8·36 (6·6–10·5)	27·8 (21·5–34·8)	15·5 (12·5–19·1)
Somalia	29,900 (25,000–36,000)	409 (345–487)	33,900 (28,300–41,600)	517 (440–621)	8,260 (4,940–15,400)	178 (106–342)	12,000 (8,820–17,200)	291 (218–454)
South Africa	123,000 (98,800–149,000)	422 (343–507)	95,800 (83,300–110,000)	330 (291–374)	5,920 (5,060–6,950)	22·2 (19·2–26·1)	13,900 (12,600–15,300)	63·3 (57·7–69·1)
South Sudan	8,280 (6,970–9,750)	232 (199–270)	9,910 (8,560–11,500)	315 (277–361)	1,450 (796–2,090)	63·4 (34·3–90·2)	2,940 (2,070–3,970)	123 (87·9–164)
Spain	1,390 (1,200–1,610)	5·89 (4·94–7)	1,890 (1,600–2,220)	7·72 (6·52–9·12)	160 (132–186)	0·25 (0·213–0·285)	219 (195–243)	0·515 (0·466–0·566)
Sri Lanka	3,920 (3,360–4,600)	31·7 (27·2–37·2)	5,220 (4,440–6,170)	45·9 (39·4–53·8)	262 (189–370)	1·99 (1·43–2·79)	611 (439–834)	5·74 (4·19–7·67)
Sudan	4,580 (3,800–5,430)	26 (22·3–30·3)	6,020 (5,140–7,060)	36·8 (31·9–42·6)	342 (137–507)	3·34 (1·19–4·83)	768 (385–1,220)	6·92 (3·43–10·9)
Suriname	25·3 (21·1–30·3)	8·65 (7·17–10·5)	38·6 (32·8–45·6)	13·4 (11·4–15·8)	2·85 (2·25–3·5)	0·921 (0·73–1·14)	7·37 (5·86–9·03)	2·68 (2·14–3·27)
Sweden	358 (301–427)	7·14 (5·87–8·65)	518 (424–629)	10·3 (8·23–12·7)	38·5 (31–45·7)	0·268 (0·219–0·316)	40·7 (35·2–45·8)	0·385 (0·334–0·432)
Switzerland	197 (170–233)	4·65 (3·92–5·55)	301 (256–355)	7·15 (5·92–8·57)	16·1 (13·3–18·8)	0·142 (0·12–0·163)	22·6 (19·7–25·5)	0·277 (0·242–0·311)
Syrian Arab Republic	728 (609–871)	9·73 (8·26–11·4)	616 (523–728)	8·99 (7·74–10·5)	33 (23·8–48·9)	0·617 (0·457–0·92)	47·2 (34·6–62·6)	0·799 (0·6–1·04)

Taiwan (Province of China)	5,000 (4,290–5,810)	35·5 (30·7–41·5)	6,750 (5,850–7,730)	44·9 (39–51·4)	243 (178–319)	1·1 (0·817–1·45)	663 (522–831)	3·67 (2·87–4·59)
Tajikistan	2,370 (1,960–2,820)	52 (44–61)	2,460 (2,060–2,920)	55·4 (47·1–64·6)	309 (245–391)	8·47 (6·78–10·7)	434 (351–535)	12·3 (10–15)
Thailand	35,500 (30,700–41,500)	81·6 (70·7–94·3)	63,400 (53,600–75,800)	149 (127–177)	2,980 (2,220–3,930)	5·45 (4·08–7·19)	4,800 (3,530–6,280)	11·2 (8·23–14·6)
Timor-Leste	660 (573–748)	119 (105–133)	685 (605–773)	133 (118–149)	114 (63·6–167)	27·2 (15·8–40·3)	184 (110–271)	46·5 (28·9–67·7)
Togo	5,400 (4,620–6,400)	155 (134–180)	5,950 (5,060–7,060)	209 (181–245)	820 (444–1,410)	37·4 (19·7–64·6)	1,300 (926–1,740)	79·2 (58·4–103)
Tokelau	0·349 (0·3–0·399)	52·8 (45·4–60·3)	0·181 (0·158–0·208)	27·2 (23·6–31)	0·0348 (0·0248–0·0486)	5·39 (3·86–7·49)	0·0378 (0·029–0·0489)	5·73 (4·4–7·41)
Tonga	16·1 (13·9–18·3)	32·6 (28·2–37·1)	14·6 (12·7–16·6)	33·7 (29·6–38·1)	1·69 (1·19–2·25)	3·88 (2·75–5·16)	2·86 (2·26–3·53)	7·84 (6·23–9·65)
Trinidad and Tobago	34·4 (29·1–40·9)	4·92 (4·11–5·88)	118 (100–138)	14·8 (12·7–17·3)	3·54 (2·53–4·99)	0·39 (0·278–0·552)	16·4 (11·9–21·5)	1·92 (1·42–2·51)
Tunisia	755 (641–882)	12·5 (10·6–14·5)	956 (813–1,120)	15·8 (13·5–18·4)	42·7 (28·1–82·8)	0·703 (0·461–1·37)	78·2 (50·6–115)	1·42 (0·92–2·04)
Turkey	5,930 (5,030–6,990)	13·8 (11·8–16·3)	8,990 (7,670–10,600)	20·2 (17·4–23·5)	278 (216–356)	0·623 (0·485–0·793)	555 (433–684)	1·37 (1·07–1·69)
Turkmenistan	982 (814–1,190)	39·7 (33–47·9)	1,420 (1,180–1,740)	55·1 (46·2–66·7)	125 (86·1–175)	5·18 (3·58–7·25)	312 (248–392)	12·7 (10·1–16)
Tuvalu	3·58 (3·1–4·12)	62·5 (54·8–71·6)	2·73 (2·36–3·07)	47·3 (41·6–53)	0·472 (0·284–0·672)	8·79 (5·25–12·5)	0·619 (0·466–0·82)	12·6 (9·52–16·5)
Uganda	28,900 (23,600–35,300)	135 (114–159)	46,300 (40,900–52,500)	298 (266–333)	4,420 (3,080–6,790)	48·9 (34·5–74·8)	9,470 (6,360–12,600)	121 (81·7–157)
Ukraine	10,900 (9,000–13,200)	44·1 (36·4–54·4)	28,700 (23,500–35,200)	117 (97–142)	693 (502–911)	2·25 (1·63–2·95)	3,350 (2,540–4,310)	12·3 (9·41–15·8)
United Arab Emirates	428 (357–519)	19·3 (16·7–22·6)	1,750 (1,400–2,190)	22 (18·8–25·6)	16 (3·49–27·2)	1·49 (0·353–2·44)	103 (31·2–166)	2·77 (0·864–4·13)

United Kingdom	3,020 (2,490–3,640)	9·76 (8–11·9)	3,190 (2,620–3,850)	9·85 (8·04–12)	164 (143–180)	0·238 (0·212–0·259)	226 (210–238)	0·407 (0·379–0·427)
United Republic of Tanzania	51,000 (42,400–61,200)	201 (172–238)	59,900 (50,900–70,700)	296 (254–348)	7,350 (5,410–11,800)	49·1 (36·5–80·4)	12,800 (9,180–16,900)	91·2 (64·9–118)
United States Virgin Islands	3·5 (2·97–4·1)	6·03 (5·11–7·14)	2·94 (2·49–3·51)	5·17 (4·39–6·12)	0·374 (0·274–0·466)	0·426 (0·313–0·542)	0·454 (0·363–0·562)	0·674 (0·54–0·844)
United States of America	3,070 (2,610–3,630)	1·64 (1·38–1·95)	5,040 (4,340–5,850)	2·66 (2·28–3·1)	377 (335–411)	0·125 (0·113–0·135)	601 (553–633)	0·249 (0·229–0·263)
Uruguay	237 (207–277)	12·8 (11–15)	411 (345–493)	23 (19·2–27·7)	19·6 (16·2–23·6)	0·685 (0·567–0·816)	36·3 (32·8–40)	1·7 (1·53–1·87)
Uzbekistan	7,500 (6,440–8,810)	47·2 (40·9–54·6)	9,520 (8,140–11,200)	63·9 (55·5–74·4)	843 (653–1,070)	5·68 (4·44–7·13)	1,600 (1,310–1,920)	11·9 (9·88–14·2)
Vanuatu	86·9 (75–98·7)	65·6 (57·9–73·1)	82·3 (72·8–91·7)	67·3 (60·2–74·3)	14·3 (7·76–21·2)	15·4 (8·2–22·6)	26·9 (19·2–35·6)	27·9 (20–36·7)
Venezuela (Bolivarian Republic of)	2,680 (2,330–3,080)	18 (15·6–20·7)	4,080 (3,480–4,760)	29·3 (25·1–34·2)	320 (216–458)	2·13 (1·44–3·05)	506 (355–688)	3·81 (2·7–5·15)
Viet Nam	57,900 (51,500–64,300)	112 (99·6–123)	96,600 (85,200–107,000)	204 (182–224)	6,930 (5,250–8,990)	14·1 (10·7–18·4)	11,700 (9,480–14,100)	36 (29·9–43·1)
Yemen	4,360 (3,690–5,200)	33·6 (28·9–39)	4,100 (3,500–4,810)	35·1 (30·5–40·3)	430 (202–669)	5·56 (2·42–8·64)	599 (410–880)	8·41 (5·89–12·2)
Zambia	17,200 (13,500–21,900)	186 (150–227)	22,800 (19,100–27,400)	314 (264–372)	2,000 (1,290–3,040)	48·6 (32·3–72·8)	5,060 (3,370–6,870)	117 (78·4–157)
Zimbabwe	21,400 (16,900–27,400)	239 (190–301)	18,600 (15,500–22,300)	287 (241–340)	3,020 (1,760–4,140)	58·3 (34·3–80·5)	7,450 (5,670–9,440)	213 (168–259)

eTable 7. Tuberculosis cases and age-standardized rates of incidence and mortality per 100,000 population among HIV-positive males and females for 204 countries and territories in 2019

	Incidence				Mortality			
	Female		Male		Female		Male	
Location	Cases	Rate (Per 100,000)	Cases	Rate (Per 100,000)	Deaths	Rate (Per 100,000)	Deaths	Rate (Per 100,000)
**Global**	**617,000 (538,000–708,000)**	**15·5 (13·5–17·7)**	**536,000 (472,000–610,000)**	**13·2 (11·6–15)**	**112,000 (78,600–143,000)**	**2·81 (1·98–3·6)**	**105,000 (74,100–135,000)**	**2·61 (1·83–3·35)**
**Central Europe, Eastern Europe, and Central Asia**	**4,350 (3,610–5,250)**	**1·88 (1·56–2·27)**	**7,910 (6,560–9,590)**	**3·43 (2·84–4·15)**	**504 (311–770)**	**0·216 (0·134–0·33)**	**1,110 (687–1,690)**	**0·477 (0·296–0·725)**
Central Asia	361 (310–420)	0·71 (0·61–0·825)	503 (433–585)	1·02 (0·879–1·19)	46·9 (29·6–70·5)	0·0926 (0·0584–0·139)	113 (71·5–167)	0·231 (0·146–0·342)
Armenia	14·2 (12·3–16·6)	0·782 (0·676–0·917)	24·7 (21·3–28·9)	1·48 (1·28–1·74)	1·23 (0·766–1·76)	0·0718 (0·0446–0·102)	4·75 (2·95–6·76)	0·288 (0·179–0·41)
Azerbaijan	16·8 (14–20·2)	0·276 (0·231–0·333)	45·3 (37·9–54·6)	0·74 (0·618–0·891)	2·86 (1·78–4·11)	0·0497 (0·031–0·0716)	8·86 (5·52–12·8)	0·148 (0·0924–0·213)
Georgia	24·4 (21·1–28·2)	1·04 (0·903–1·21)	64·6 (55·9–74·7)	3·14 (2·72–3·63)	2·2 (1·6–2·48)	0·105 (0·0765–0·118)	7·67 (5·57–8·52)	0·394 (0·286–0·438)
Kazakhstan	51·7 (44·2–60·7)	0·499 (0·427–0·586)	86 (73·5–101)	0·855 (0·731–1)	4·5 (2·72–7·05)	0·0436 (0·0264–0·0682)	11·4 (6·92–17·9)	0·115 (0·0693–0·179)
Kyrgyzstan	60·4 (51·3–71)	1·83 (1·55–2·15)	65·3 (55·4–76·8)	2·11 (1·79–2·48)	4·8 (2·99–7·39)	0·145 (0·0903–0·223)	10·6 (6·62–16·4)	0·341 (0·212–0·524)
Mongolia	1·05 (0·875–1·26)	0·0571 (0·0476–0·0683)	4·49 (3·75–5·38)	0·298 (0·249–0·357)	0·187 (0·108–0·318)	0·0102 (0·00574–0·0173)	0·772 (0·438–1·27)	0·0523 (0·0275–0·0869)
Tajikistan	33·4 (28·1–39·6)	0·773 (0·648–0·914)	14 (11·7–16·5)	0·3 (0·252–0·355)	8 (4·97–12·4)	0·18 (0·112–0·279)	3·21 (2–4·98)	0·0702 (0·0436–0·109)
Turkmenistan	22·4 (18·7–26·9)	0·87 (0·728–1·05)	47·7 (39·9–57·4)	1·72 (1·43–2·07)	4·34 (2·63–6·76)	0·17 (0·103–0·264)	16·7 (10·1–26·1)	0·592 (0·358–0·921)
Uzbekistan	137 (117–160)	0·761 (0·653–0·89)	151 (130–177)	0·855 (0·733–1)	18·8 (11·6–29)	0·104 (0·0643–0·161)	48·9 (30·2–75·3)	0·285 (0·176–0·439)

Central Europe	125 (107–147)	0·211 (0·18–0·248)	262 (224–309)	0·405 (0·346–0·477)	17·7 (10·9–27)	0·0327 (0·0202–0·0502)	52·4 (32·4–80·5)	0·0823 (0·0509–0·126)
Albania	0·549 (0·469–0·643)	0·0406 (0·0347–0·0476)	1·22 (1·04–1·42)	0·084 (0·0717–0·0983)	0·101 (0·0573–0·171)	0·00757 (0·00428–0·0128)	0·235 (0·133–0·397)	0·0157 (0·00886–0·0264)
Bosnia and Herzegovina	1·21 (1·02–1·44)	0·0437 (0·0369–0·0517)	1·67 (1·41–1·97)	0·0861 (0·0727–0·102)	0·309 (0·0968–0·707)	0·0124 (0·00524–0·0256)	0·335 (0·255–0·397)	0·0188 (0·0143–0·0218)
Bulgaria	0·704 (0·604–0·82)	0·0215 (0·0184–0·025)	2·48 (2·12–2·88)	0·0637 (0·0546–0·0741)	0·112 (0·0685–0·175)	0·0032 (0·00195–0·00499)	0·418 (0·255–0·652)	0·00981 (0·00598–0·0153)
Croatia	0·896 (0·774–1·05)	0·0336 (0·0291–0·0392)	3·51 (3·04–4·1)	0·13 (0·112–0·151)	0·226 (0·136–0·364)	0·00937 (0·00564–0·0151)	0·732 (0·441–1·18)	0·0278 (0·0167–0·0448)
Czech Republic	3·06 (2·63–3·58)	0·0487 (0·0419–0·057)	13·5 (11·6–15·8)	0·206 (0·177–0·241)	0·855 (0·516–1·37)	0·0125 (0·00752–0·0201)	2·04 (1·23–3·28)	0·032 (0·0193–0·0515)
Hungary	1·94 (1·67–2·28)	0·029 (0·0249–0·0339)	8·89 (7·65–10·4)	0·137 (0·118–0·16)	0·327 (0·199–0·52)	0·00501 (0·00305–0·00797)	1·37 (0·833–2·18)	0·0211 (0·0128–0·0335)
Montenegro	0·112 (0·0949–0·133)	0·0365 (0·0309–0·0434)	0·333 (0·282–0·396)	0·0943 (0·08–0·112)	0·023 (0·0137–0·0368)	0·00921 (0·00548–0·0148)	0·0526 (0·0313–0·0843)	0·0168 (0·01–0·027)
North Macedonia	0·608 (0·513–0·72)	0·055 (0·0465–0·0652)	0·965 (0·815–1·14)	0·0757 (0·0639–0·0897)	0·149 (0·0833–0·254)	0·0175 (0·00979–0·0301)	0·206 (0·115–0·355)	0·0207 (0·0116–0·0356)
Poland	25·2 (21–30·5)	0·113 (0·0943–0·137)	68·5 (57–82·7)	0·296 (0·246–0·358)	3·3 (2–5·24)	0·0158 (0·00957–0·0251)	11·2 (6·76–17·8)	0·0495 (0·0299–0·0786)
Romania	78·4 (67·1–92·2)	0·878 (0·751–1·03)	111 (95·2–131)	1·15 (0·986–1·36)	9·38 (5·77–14·7)	0·118 (0·0724–0·184)	24·6 (15·1–38·4)	0·25 (0·153–0·391)
Serbia	11·6 (9·91–13·7)	0·247 (0·21–0·29)	48·9 (41·7–57·5)	0·967 (0·825–1·14)	2·83 (1·79–4·26)	0·0699 (0·0436–0·104)	11 (6·82–16)	0·224 (0·139–0·329)
Slovakia	0·163 (0·138–0·193)	0·00565 (0·00479–0·00669)	0·438 (0·371–0·519)	0·0131 (0·0111–0·0155)	0·0291 (0·0162–0·0491)	0·00115 (0·000645–0·00195)	0·0781 (0·0436–0·132)	0·00248 (0·00139–0·00419)
Slovenia	0·17 (0·146–0·2)	0·0135 (0·0116–0·0158)	0·883 (0·761–1·04)	0·066 (0·0569–0·0775)	0·0262 (0·0152–0·0436)	0·00252 (0·00147–0·00418)	0·154 (0·0899–0·257)	0·0128 (0·00744–0·0213)

Eastern Europe	3,860 (3,190–4,700)	3·28 (2·71–3·99)	7,140 (5,890–8,700)	6·12 (5·05–7·45)	439 (268–671)	0·365 (0·223–0·557)	943 (580–1,440)	0·8 (0·492–1·22)
Belarus	40·4 (34·4–48·2)	0·74 (0·631–0·884)	56·6 (48·3–67·5)	1·07 (0·911–1·27)	5·26 (3·26–8·21)	0·0954 (0·0591–0·149)	13 (8·08–20·4)	0·243 (0·15–0·379)
Estonia	6·46 (5·55–7·59)	0·999 (0·858–1·17)	14·5 (12·5–17·1)	2·12 (1·82–2·49)	0·918 (0·576–1·41)	0·148 (0·093–0·228)	3·58 (2·25–5·52)	0·519 (0·325–0·799)
Latvia	19 (16·2–22·4)	1·84 (1·57–2·17)	29·3 (25–34·6)	2·96 (2·53–3·5)	4·22 (2·57–6·37)	0·377 (0·23–0·569)	4·75 (2·89–7·16)	0·464 (0·283–0·701)
Lithuania	7·93 (6·81–9·34)	0·483 (0·415–0·569)	12·8 (11–15·1)	0·912 (0·784–1·07)	3·16 (1·92–4·87)	0·178 (0·108–0·274)	1·62 (0·983–2·49)	0·113 (0·0686–0·174)
Republic of Moldova	37·9 (32·2–45·3)	1·7 (1·44–2·03)	43·2 (36·7–51·7)	1·92 (1·63–2·3)	2·62 (1·62–3·97)	0·119 (0·0735–0·181)	7·42 (4·57–11·2)	0·332 (0·204–0·502)
Russian Federation	2,160 (1,760–2,670)	2·73 (2·22–3·37)	4,740 (3,850–5,830)	5·83 (4·74–7·18)	218 (133–334)	0·273 (0·167–0·419)	573 (351–880)	0·702 (0·429–1·08)
Ukraine	1,580 (1,310–1,930)	5·98 (4·94–7·28)	2,250 (1,860–2,740)	9·02 (7·45–11)	206 (126–322)	0·75 (0·46–1·18)	339 (208–532)	1·33 (0·819–2·09)
**High-income**	**2,830 (2,460–3,290)**	**0·492 (0·428–0·572)**	**5,860 (5,110–6,760)**	**0·954 (0·831–1·1)**	**446 (325–537)**	**0·0744 (0·0544–0·0888)**	**1,270 (936–1,540)**	**0·2 (0·148–0·241)**
Australasia	10·6 (9·13–12·2)	0·0639 (0·0553–0·0743)	40·7 (35·3–47·4)	0·231 (0·201–0·269)	1·19 (0·68–1·94)	0·00702 (0·00402–0·0114)	4·14 (2·37–6·76)	0·0246 (0·0141–0·0402)
Australia	7·8 (6·75–9·05)	0·0545 (0·0472–0·0633)	31·2 (27–36·2)	0·204 (0·176–0·236)	1·02 (0·571–1·7)	0·00704 (0·00393–0·0117)	3·52 (1·96–5·84)	0·0243 (0·0136–0·0404)
New Zealand	2·75 (2·32–3·28)	0·119 (0·1–0·142)	9·49 (8·02–11·3)	0·408 (0·345–0·484)	0·166 (0·0986–0·267)	0·00689 (0·00408–0·011)	0·62 (0·368–0·992)	0·027 (0·0161–0·0433)
High-income Asia Pacific	163 (140–190)	0·133 (0·114–0·155)	646 (555–752)	0·513 (0·44–0·597)	20·3 (14·5–25·5)	0·016 (0·0116–0·0201)	96 (78·4–113)	0·0742 (0·0608–0·0869)
Brunei Darussalam	0·537 (0·464–0·627)	0·228 (0·197–0·266)	1·65 (1·42–1·92)	0·603 (0·52–0·703)	0·101 (0·0636–0·156)	0·0479 (0·0301–0·0736)	0·256 (0·16–0·393)	0·105 (0·0657–0·161)
Japan	35·5 (29·7–41·8)	0·0395 (0·033–0·0465)	89·5 (75–105)	0·0975 (0·0817–0·115)	13·8 (8·35–18·7)	0·0155 (0·00944–0·0212)	37 (22·4–50·5)	0·0423 (0·0256–0·058)

Republic of Korea	109 (93·4–127)	0·309 (0·266–0·361)	502 (431–587)	1·34 (1·15–1·57)	6·15 (5·53–6·77)	0·0174 (0·0158–0·0189)	55·8 (51·3–60·6)	0·145 (0·133–0·156)
Singapore	18·4 (15·8–21·4)	0·486 (0·417–0·566)	52·7 (45·3–61·4)	1·29 (1·1–1·5)	0·276 (0·168–0·441)	0·00752 (0·00456–0·012)	3·03 (1·84–4·83)	0·0719 (0·0436–0·115)
High-income North America	229 (198–266)	0·106 (0·0919–0·124)	671 (581–782)	0·299 (0·259–0·348)	45·4 (28·5–70·1)	0·0213 (0·0134–0·033)	125 (78·8–194)	0·0569 (0·0358–0·0882)
Canada	44·2 (38·4–51)	0·214 (0·186–0·247)	145 (126–167)	0·626 (0·544–0·722)	6·81 (4·18–10·7)	0·0337 (0·0207–0·0527)	20·1 (12·3–31·3)	0·0903 (0·0554–0·141)
Greenland	1·33 (1·13–1·59)	4·47 (3·78–5·34)	1·31 (1·11–1·56)	3·8 (3·22–4·55)	0·236 (0·146–0·343)	0·787 (0·488–1·14)	0·234 (0·145–0·339)	0·682 (0·424–0·98)
United States of America	183 (158–214)	0·0936 (0·0807–0·109)	526 (453–614)	0·261 (0·224–0·304)	38·4 (24–58·8)	0·0198 (0·0124–0·0304)	105 (65·4–161)	0·053 (0·033–0·0813)
Southern Latin America	1,860 (1,600–2,200)	5·13 (4·41–6·07)	3,130 (2,710–3,670)	8·91 (7·71–10·4)	275 (212–292)	0·745 (0·573–0·789)	735 (573–773)	2·06 (1·61–2·17)
Argentina	1,690 (1,440–2,000)	6·96 (5·96–8·25)	2,410 (2,070–2,830)	10·3 (8·86–12·1)	235 (180–250)	0·955 (0·731–1·02)	540 (413–571)	2·3 (1·76–2·43)
Chile	138 (120–160)	1·34 (1·16–1·55)	641 (556–744)	6·38 (5·53–7·4)	36·6 (29·8–38·3)	0·349 (0·284–0·366)	181 (148–189)	1·76 (1·43–1·84)
Uruguay	32·4 (27·8–38·1)	1·7 (1·46–2)	81·2 (69·6–95·6)	4·71 (4·04–5·55)	3·59 (2·19–5·51)	0·171 (0·104–0·263)	13 (7·9–19·9)	0·698 (0·425–1·07)
Western Europe	565 (488–659)	0·222 (0·191–0·259)	1,370 (1,190–1,590)	0·501 (0·434–0·582)	104 (64·7–154)	0·042 (0·0262–0·0622)	315 (198–465)	0·116 (0·0727–0·171)
Andorra	0·135 (0·114–0·162)	0·289 (0·244–0·346)	0·321 (0·272–0·384)	0·598 (0·505–0·715)	0·0198 (0·011–0·0309)	0·0357 (0·0196–0·0557)	0·05 (0·0306–0·0774)	0·0789 (0·0483–0·122)
Austria	5·94 (5·15–6·88)	0·115 (0·0995–0·133)	19·5 (16·9–22·5)	0·349 (0·302–0·404)	0·895 (0·546–1·43)	0·0186 (0·0114–0·0298)	3·14 (1·92–5·02)	0·0584 (0·0356–0·0933)
Belgium	16·2 (14–18·7)	0·235 (0·204–0·272)	31·7 (27·5–36·7)	0·421 (0·365–0·487)	2·52 (1·55–3·94)	0·0395 (0·0242–0·0617)	5·42 (3·32–8·47)	0·0758 (0·0464–0·118)
Cyprus	0·478 (0·407–0·565)	0·0715 (0·0609–0·0846)	1·39 (1·18–1·64)	0·175 (0·15–0·208)	0·0795 (0·0474–0·126)	0·00998 (0·00594–0·0158)	0·276 (0·164–0·437)	0·0335 (0·0199–0·0531)
Denmark	5·03 (4·33–5·86)	0·152 (0·131–0·177)	5·53 (4·77–6·45)	0·145 (0·125–0·17)	0·469 (0·282–0·749)	0·0149 (0·00897–0·0238)	1·23 (0·736–1·95)	0·0329 (0·0198–0·0525)

Finland	2·52 (2·2–2·91)	0·0731 (0·0636–0·0842)	3·54 (3·09–4·08)	0·106 (0·0926–0·123)	0·502 (0·305–0·635)	0·0165 (0·01–0·0208)	1·5 (0·908–1·89)	0·0447 (0·0271–0·0563)
France	102 (89–118)	0·261 (0·228–0·301)	251 (219–289)	0·592 (0·517–0·682)	38·1 (23·3–55)	0·101 (0·0617–0·146)	104 (63·3–148)	0·259 (0·158–0·37)
Germany	71·2 (61·6–82·5)	0·141 (0·122–0·163)	192 (166–223)	0·331 (0·286–0·384)	8·75 (5·32–13·8)	0·0182 (0·0111–0·0287)	32·9 (20–52)	0·0593 (0·0361–0·0935)
Greece	9·25 (8·07–10·5)	0·167 (0·145–0·19)	19·2 (16·7–21·9)	0·331 (0·289–0·377)	1·97 (1·69–2·11)	0·0377 (0·0324–0·0404)	8 (6·86–8·58)	0·138 (0·118–0·148)
Iceland	0·176 (0·147–0·209)	0·0915 (0·0765–0·109)	0·544 (0·454–0·646)	0·281 (0·234–0·333)	0·0152 (0·00894–0·0246)	0·00856 (0·00505–0·0139)	0·0697 (0·0411–0·113)	0·0365 (0·0216–0·0593)
Ireland	9·16 (7·93–10·7)	0·344 (0·298–0·402)	16·7 (14·4–19·4)	0·624 (0·54–0·728)	0·984 (0·714–1·1)	0·0393 (0·0285–0·0439)	2·72 (1·97–3·01)	0·102 (0·0739–0·113)
Israel	4·03 (3·46–4·7)	0·0822 (0·0708–0·0959)	9·46 (8·14–11)	0·2 (0·172–0·234)	0·618 (0·372–0·973)	0·0128 (0·00768–0·0201)	1·44 (0·865–2·26)	0·0305 (0·0184–0·048)
Italy	50·7 (42·6–60·2)	0·138 (0·115–0·163)	81·3 (68·2–96·6)	0·205 (0·172–0·243)	5 (3·12–7·78)	0·0138 (0·00858–0·0214)	17·2 (10·7–26·7)	0·0419 (0·0262–0·0652)
Luxembourg	0·562 (0·474–0·667)	0·154 (0·13–0·183)	1·26 (1·07–1·5)	0·317 (0·268–0·377)	0·0361 (0·0216–0·0576)	0·0104 (0·00625–0·0166)	0·105 (0·0632–0·168)	0·0275 (0·0165–0·0438)
Malta	0·8 (0·666–0·96)	0·319 (0·266–0·383)	0·694 (0·578–0·833)	0·276 (0·23–0·332)	0·0216 (0·013–0·0346)	0·00921 (0·00552–0·0147)	0·0653 (0·0392–0·104)	0·0257 (0·0154–0·041)
Netherlands	19·4 (16·7–22·5)	0·196 (0·168–0·227)	46·5 (40–54)	0·395 (0·34–0·459)	3·9 (2·34–5·95)	0·0423 (0·0253–0·0646)	9·9 (5·93–15·1)	0·0915 (0·0548–0·139)
Norway	4·52 (3·76–5·38)	0·168 (0·14–0·2)	6·73 (5·61–8·04)	0·227 (0·189–0·271)	0·682 (0·416–1·02)	0·0251 (0·0153–0·0375)	1·67 (1·01–2·51)	0·0522 (0·0317–0·0784)
Portugal	59·6 (51·6–68·8)	0·987 (0·854–1·14)	174 (150–201)	2·89 (2·5–3·33)	13·3 (8·29–20·5)	0·208 (0·129–0·32)	50·4 (31·4–77·6)	0·785 (0·488–1·21)
Principality of Monaco	0·192 (0·166–0·224)	0·961 (0·832–1·12)	0·286 (0·247–0·332)	1·27 (1·1–1·47)	0·0546 (0·0213–0·0946)	0·214 (0·0685–0·365)	0·0945 (0·0519–0·147)	0·338 (0·174–0·527)
Republic of San Marino	0·073 (0·063–0·0848)	0·388 (0·335–0·451)	0·0957 (0·0826–0·111)	0·507 (0·438–0·59)	0·0155 (0·00866–0·024)	0·0713 (0·0367–0·111)	0·0233 (0·0147–0·0357)	0·107 (0·0669–0·164)

Spain	63 (54–73·4)	0·217 (0·186–0·253)	238 (204–277)	0·774 (0·664–0·901)	11·5 (7·07–17·9)	0·0411 (0·0252–0·0636)	46·7 (28·6–72·3)	0·147 (0·0899–0·227)
Sweden	10·7 (8·89–12·8)	0·199 (0·166–0·239)	19 (15·8–22·7)	0·319 (0·266–0·383)	1·51 (0·882–2·36)	0·0269 (0·0157–0·042)	2·73 (1·61–4·27)	0·045 (0·0266–0·0707)
Switzerland	8·12 (6·98–9·54)	0·145 (0·125–0·17)	16·5 (14·2–19·4)	0·262 (0·225–0·308)	0·883 (0·539–1·4)	0·0182 (0·0111–0·0289)	2·31 (1·41–3·65)	0·0398 (0·0243–0·063)
United Kingdom of Great Britain and Northern Ireland	121 (99·3–146)	0·311 (0·255–0·375)	237 (195–286)	0·593 (0·487–0·714)	11·8 (7·16–18·7)	0·0319 (0·0195–0·0507)	22·8 (13·9–36·2)	0·0582 (0·0355–0·0923)
**Latin America and Caribbean**	**9,220 (7,910–10,700)**	**2·88 (2·47–3·36)**	**16,100 (13,700–18,700)**	**5·33 (4·56–6·21)**	**1,510 (930–2,280)**	**0·477 (0·294–0·721)**	**2,820 (1,750–4,270)**	**0·94 (0·584–1·42)**
Andean Latin America	931 (796–1,080)	2·85 (2·44–3·31)	2,110 (1,810–2,460)	6·61 (5·66–7·68)	155 (98·7–234)	0·479 (0·305–0·723)	336 (212–503)	1·06 (0·669–1·59)
Ecuador	364 (309–425)	4·03 (3·43–4·71)	828 (705–968)	9·41 (8·01–11)	61·7 (38·2–93·2)	0·692 (0·429–1·04)	180 (112–272)	2·1 (1·3–3·17)
Peru	460 (392–537)	2·6 (2·22–3·04)	1,090 (934–1,280)	6·31 (5·38–7·36)	75·4 (47–115)	0·446 (0·278–0·679)	124 (77·3–189)	0·728 (0·454–1·11)
Plurinational State of Bolivia	108 (91·8–125)	1·82 (1·55–2·12)	192 (164–224)	3·29 (2·81–3·83)	17·8 (3·96–36·1)	0·298 (0·0672–0·596)	31·9 (13·7–55·8)	0·548 (0·241–0·939)
Caribbean	1,680 (1,460–1,930)	6·76 (5·87–7·74)	1,330 (1,150–1,530)	5·51 (4·78–6·33)	348 (207–538)	1·39 (0·828–2·16)	373 (226–571)	1·54 (0·93–2·35)
Antigua and Barbuda	1·02 (0·85–1·22)	2 (1·67–2·4)	1·65 (1·37–1·97)	3·4 (2·83–4·06)	0·0269 (0·0167–0·0415)	0·0507 (0·0316–0·0783)	0·0663 (0·0413–0·102)	0·132 (0·0825–0·204)
Barbados	1·19 (1–1·41)	0·683 (0·576–0·81)	1·78 (1·5–2·11)	1·04 (0·876–1·23)	0·146 (0·0885–0·223)	0·0856 (0·052–0·131)	0·285 (0·173–0·436)	0·166 (0·101–0·253)
Belize	10·1 (8·69–11·8)	4·83 (4·16–5·65)	13·4 (11·5–15·7)	6·7 (5·77–7·84)	2·04 (1·24–3·13)	0·999 (0·606–1·53)	3·94 (2·39–6·05)	2·03 (1·23–3·12)
Bermuda	0·634 (0·531–0·761)	1·93 (1·61–2·31)	1·28 (1·07–1·54)	3·63 (3·04–4·35)	0·00504 (0·00301–0·00769)	0·0128 (0·00765–0·0196)	0·017 (0·0102–0·026)	0·0409 (0·0244–0·0624)
Commonwealth of the Bahamas	6·59 (5·64–7·76)	2·97 (2·55–3·5)	7·41 (6·34–8·72)	3·54 (3·03–4·16)	1·54 (0·927–2·41)	0·694 (0·417–1·08)	2·15 (1·29–3·35)	1·01 (0·606–1·58)

Cuba	10 (8·64–11·7)	0·151 (0·13–0·176)	35·5 (30·6–41·5)	0·52 (0·449–0·608)	0·73 (0·463–1·11)	0·0115 (0·00732–0·0175)	4·07 (2·58–6·17)	0·0613 (0·0389–0·0929)
Dominica	0·911 (0·786–1·06)	2·65 (2·28–3·08)	1·56 (1·34–1·81)	4·19 (3·61–4·88)	0·163 (0·0983–0·253)	0·473 (0·285–0·735)	0·507 (0·306–0·788)	1·35 (0·812–2·09)
Dominican Republic	420 (354–496)	7·73 (6·51–9·13)	420 (353–496)	7·68 (6·46–9·07)	77·1 (46·8–120)	1·43 (0·869–2·23)	137 (83·4–213)	2·54 (1·55–3·95)
Grenada	0·499 (0·422–0·591)	0·93 (0·786–1·1)	0·954 (0·807–1·13)	1·68 (1·42–1·99)	0·0296 (0·0187–0·0452)	0·055 (0·0347–0·084)	0·0798 (0·0504–0·122)	0·137 (0·0864–0·209)
Guyana	43·7 (37·3–51·1)	11·1 (9·51–13)	35·5 (30·2–41·5)	9·59 (8·18–11·2)	9·7 (5·88–15)	2·45 (1·49–3·8)	13·7 (8·29–21·2)	3·66 (2·22–5·68)
Haiti	1,070 (927–1,230)	17·5 (15·2–20·2)	688 (597–791)	13·3 (11·5–15·3)	233 (136–371)	3·99 (2·34–6·35)	179 (105–285)	3·61 (2·12–5·75)
Jamaica	16·6 (14·1–19·5)	1·08 (0·918–1·27)	19 (16·2–22·3)	1·29 (1·1–1·51)	2·35 (1·43–3·61)	0·156 (0·0949–0·24)	3·28 (1·99–5·03)	0·224 (0·136–0·344)
Puerto Rico	8·57 (7·34–9·92)	0·437 (0·374–0·506)	15·1 (12·9–17·5)	0·814 (0·696–0·942)	2·04 (1·24–3·1)	0·0969 (0·059–0·147)	5·32 (3·24–8·08)	0·26 (0·158–0·395)
Saint Kitts and Nevis	1·67 (1·4–1·99)	4·97 (4·17–5·93)	3·5 (2·94–4·18)	10 (8·44–12)	0·185 (0·11–0·289)	0·535 (0·319–0·832)	0·448 (0·266–0·703)	1·23 (0·73–1·92)
Saint Lucia	0·983 (0·837–1·16)	0·986 (0·84–1·16)	1·48 (1·26–1·75)	1·49 (1·27–1·76)	0·259 (0·162–0·396)	0·253 (0·158–0·388)	0·461 (0·288–0·707)	0·45 (0·281–0·689)
Saint Vincent and the Grenadines	1·56 (1·33–1·83)	2·72 (2·32–3·2)	2·57 (2·19–3·02)	4·2 (3·59–4·94)	0·322 (0·198–0·491)	0·546 (0·335–0·832)	0·663 (0·406–1·01)	1·04 (0·638–1·59)
Suriname	10·6 (9·07–12·5)	3·51 (2·99–4·13)	12·2 (10·4–14·4)	4·13 (3·51–4·86)	2·39 (1·47–3·66)	0·789 (0·485–1·21)	3·62 (2·23–5·56)	1·2 (0·741–1·85)
Trinidad and Tobago	21·6 (18·5–25·2)	2·8 (2·4–3·27)	21·9 (18·8–25·4)	2·69 (2·31–3·13)	3·65 (2·22–5·68)	0·477 (0·29–0·742)	5·98 (3·64–9·3)	0·724 (0·44–1·13)
United States Virgin Islands	0·437 (0·375–0·512)	0·786 (0·675–0·922)	0·481 (0·413–0·563)	0·904 (0·776–1·06)	0·0665 (0·0401–0·104)	0·11 (0·0663–0·172)	0·14 (0·0844–0·219)	0·238 (0·144–0·372)
Central Latin America	1,450 (1,260–1,660)	1·08 (0·938–1·23)	4,110 (3,560–4,720)	3·29 (2·85–3·78)	300 (192–449)	0·225 (0·144–0·336)	848 (535–1,280)	0·683 (0·431–1·03)

Bolivarian Republic of Venezuela	239 (206–275)	1·55 (1·33–1·78)	675 (581–777)	4·62 (3·98–5·32)	49 (29·6–75·1)	0·318 (0·192–0·487)	133 (80·3–204)	0·895 (0·541–1·37)
Colombia	280 (243–320)	1·06 (0·92–1·21)	875 (760–999)	3·53 (3·06–4·03)	47 (29–72·4)	0·18 (0·111–0·276)	142 (87·4–218)	0·574 (0·354–0·883)
Costa Rica	8·43 (7·28–9·74)	0·314 (0·271–0·363)	27·1 (23·4–31·3)	1·09 (0·939–1·26)	1·81 (1·11–2·8)	0·0685 (0·042–0·106)	5·22 (3·2–8·07)	0·211 (0·129–0·327)
El Salvador	29 (24·9–33·5)	0·857 (0·736–0·99)	47·3 (40·6–54·6)	1·76 (1·51–2·03)	4·7 (2·96–7·29)	0·144 (0·0904–0·223)	10·1 (6·32–15·6)	0·38 (0·239–0·588)
Guatemala	187 (164–214)	2·14 (1·87–2·44)	346 (303–396)	4·81 (4·21–5·5)	44·7 (27·7–68·6)	0·514 (0·319–0·79)	95·7 (59·3–147)	1·3 (0·805–1·99)
Honduras	118 (102–134)	2·69 (2·33–3·06)	148 (128–168)	3·66 (3·17–4·17)	15·7 (10·7–23·1)	0·325 (0·224–0·471)	24·9 (19·5–31·6)	0·591 (0·473–0·763)
Mexico	511 (437–600)	0·757 (0·647–0·889)	1,790 (1,530–2,110)	2·83 (2·41–3·32)	121 (75·1–184)	0·181 (0·112–0·274)	394 (244–598)	0·624 (0·386–0·948)
Nicaragua	18·4 (15·9–21·2)	0·553 (0·476–0·636)	32·3 (27·9–37·3)	1·03 (0·892–1·19)	3·16 (1·97–4·91)	0·0965 (0·0603–0·15)	5·14 (3·22–8)	0·167 (0·105–0·26)
Panama	58·1 (50·3–67)	2·78 (2·4–3·2)	164 (142–189)	7·73 (6·68–8·9)	12·5 (7·49–19·1)	0·596 (0·357–0·91)	38·8 (23·3–59·2)	1·83 (1·1–2·79)
Tropical Latin America	5,160 (4,360–6,090)	4·01 (3·4–4·75)	8,520 (7,210–10,100)	7·03 (5·95–8·31)	705 (433–1,070)	0·554 (0·34–0·844)	1,260 (774–1,910)	1·04 (0·641–1·58)
Brazil	5,030 (4,250–5,940)	4·02 (3·4–4·75)	8,300 (7,010–9,820)	7·05 (5·96–8·34)	679 (417–1,030)	0·545 (0·335–0·829)	1,220 (750–1,850)	1·04 (0·637–1·57)
Paraguay	128 (109–150)	3·69 (3·14–4·32)	220 (188–258)	6·35 (5·41–7·45)	26·1 (16–40·1)	0·783 (0·477–1·2)	39·5 (24·3–60·4)	1·16 (0·714–1·78)
**North Africa and Middle East**	**2,320 (2,000–2,700)**	**0·775 (0·668–0·905)**	**2,930 (2,530–3,410)**	**0·885 (0·763–1·03)**	**276 (167–417)**	**0·0932 (0·0568–0·141)**	**335 (210–503)**	**0·104 (0·0648–0·157)**
North Africa and Middle East	2,320 (2,000–2,700)	0·775 (0·668–0·905)	2,930 (2,530–3,410)	0·885 (0·763–1·03)	276 (167–417)	0·0932 (0·0568–0·141)	335 (210–503)	0·104 (0·0648–0·157)
Afghanistan	8·97 (7·58–10·6)	0·0528 (0·0446–0·0625)	15 (12·7–17·8)	0·103 (0·0874–0·123)	1·49 (0·272–2·83)	0·00833 (0·0018–0·0151)	2·59 (1·1–4·5)	0·0173 (0·00811–0·0295)
Algeria	46·8 (40–54·9)	0·211 (0·181–0·248)	56·9 (48·6–66·9)	0·27 (0·231–0·317)	3·89 (2·3–6·14)	0·0176 (0·0104–0·0278)	5·7 (2·09–9·07)	0·0272 (0·00939–0·0434)

Bahrain	1·92 (1·6–2·31)	0·293 (0·244–0·353)	5·18 (4·32–6·24)	0·41 (0·342–0·494)	0·103 (0·0625–0·161)	0·0157 (0·00953–0·0246)	0·428 (0·26–0·673)	0·0338 (0·0205–0·0531)
Egypt	30·2 (26–35·4)	0·0624 (0·0537–0·0731)	62·7 (54–73·4)	0·133 (0·114–0·156)	3·96 (2·23–6·61)	0·00804 (0·00455–0·0134)	7·8 (4·39–13·3)	0·0161 (0·00905–0·0274)
Iraq	1·88 (1·6–2·22)	0·0088 (0·00747–0·0104)	1·21 (1·03–1·43)	0·00585 (0·00497–0·00689)	0·257 (0·151–0·417)	0·00127 (0·000744–0·00205)	0·122 (0·0716–0·198)	0·00055 (0·000323–0·000892)
Islamic Republic of Iran	959 (828–1,110)	2·12 (1·83–2·47)	1,480 (1,280–1,720)	2·93 (2·53–3·4)	103 (68·8–146)	0·237 (0·158–0·335)	158 (104–223)	0·324 (0·215–0·458)
Jordan	0·657 (0·548–0·784)	0·0123 (0·0103–0·0147)	1·94 (1·62–2·31)	0·0346 (0·0289–0·0412)	0·0939 (0·0543–0·155)	0·00178 (0·00103–0·00292)	0·166 (0·0962–0·274)	0·00292 (0·00169–0·00481)
Kuwait	0·947 (0·79–1·13)	0·0385 (0·0321–0·046)	1·77 (1·48–2·12)	0·0637 (0·0531–0·0762)	0·0716 (0·0398–0·121)	0·00308 (0·00171–0·0052)	0·0693 (0·0386–0·117)	0·00248 (0·00138–0·0042)
Lebanon	5·54 (4·74–6·49)	0·206 (0·176–0·241)	11·2 (9·6–13·2)	0·437 (0·374–0·513)	0·705 (0·102–1·5)	0·0258 (0·0037–0·0549)	0·892 (0·435–1·74)	0·035 (0·017–0·0681)
Libya	50·6 (43–60·2)	1·37 (1·16–1·62)	52·5 (44·5–62·4)	1·34 (1·14–1·6)	3·67 (0·129–8·03)	0·0996 (0·00354–0·222)	3·56 (0·437–8·28)	0·0929 (0·0111–0·219)
Morocco	364 (308–434)	1·93 (1·63–2·3)	359 (303–428)	1·94 (1·63–2·3)	32·2 (1·28–90)	0·174 (0·00673–0·489)	38·9 (3·81–99·2)	0·212 (0·0208–0·549)
Palestine	0·795 (0·667–0·942)	0·0307 (0·0258–0·0364)	1·11 (0·931–1·31)	0·0434 (0·0364–0·0514)	0·119 (0·066–0·201)	0·00498 (0·00278–0·00844)	0·166 (0·0922–0·28)	0·00711 (0·00396–0·012)
Oman	3·55 (2·94–4·3)	0·204 (0·169–0·247)	10·9 (9·01–13·1)	0·322 (0·267–0·39)	0·121 (0·0729–0·193)	0·00752 (0·00453–0·012)	0·565 (0·341–0·902)	0·0206 (0·0124–0·0329)
Qatar	0·257 (0·21–0·315)	0·0354 (0·0289–0·0433)	0·681 (0·557–0·835)	0·0276 (0·0226–0·0338)	0·00787 (0·00467–0·0131)	0·00102 (0·000604–0·00169)	0·0294 (0·0174–0·0489)	0·00131 (0·000777–0·00218)
Saudi Arabia	206 (171–248)	1·1 (0·914–1·32)	220 (183–265)	0·875 (0·727–1·05)	29·9 (18·4–47)	0·17 (0·105–0·266)	29·6 (18·2–46·2)	0·129 (0·0792–0·2)
Sudan	482 (409–566)	2·62 (2·22–3·07)	350 (297–411)	2·15 (1·82–2·52)	80·8 (40·3–135)	0·483 (0·241–0·808)	57·1 (28·5–95·4)	0·375 (0·187–0·626)

Syrian Arab Republic	3·12 (2·64–3·69)	0·0419 (0·0355–0·0495)	4·49 (3·81–5·3)	0·0656 (0·0557–0·0775)	0·365 (0·21–0·604)	0·00511 (0·00294–0·00847)	0·367 (0·211–0·607)	0·00575 (0·00331–0·00951)
Tunisia	18·3 (15·7–21·4)	0·295 (0·253–0·345)	24 (20·6–28·2)	0·389 (0·334–0·456)	1·77 (0·907–2·95)	0·0276 (0·0148–0·0455)	2·63 (0·875–4·47)	0·0414 (0·0151–0·0697)
Turkey	33·6 (28·8–39·4)	0·0769 (0·0657–0·0901)	68·6 (58·7–80·4)	0·148 (0·127–0·174)	3·22 (1·9–5·07)	0·00785 (0·00464–0·0124)	6·27 (3·71–9·86)	0·0141 (0·00837–0·0223)
United Arab Emirates	7·66 (6·2–9·44)	0·256 (0·208–0·316)	43·6 (35·4–53·8)	1·42 (1·16–1·75)	0·382 (0·0769–0·708)	0·0125 (0·00291–0·0229)	2·63 (1·04–5·01)	0·0939 (0·0139–0·218)
Yemen	89·5 (76·5–105)	0·614 (0·524–0·72)	155 (132–181)	1·19 (1·02–1·4)	9·28 (0·361–23·2)	0·0603 (0·0027–0·149)	17·4 (2·24–41·7)	0·131 (0·018–0·316)
**South Asia**	**36,400 (31,300–42,500)**	**4·05 (3·48–4·72)**	**53,100 (45,600–61,900)**	**5·85 (5·03–6·83)**	**5,710 (4,260–7,250)**	**0·638 (0·479–0·809)**	**6,910 (5,180–8,680)**	**0·763 (0·571–0·953)**
South Asia	36,400 (31,300–42,500)	4·05 (3·48–4·72)	53,100 (45,600–61,900)	5·85 (5·03–6·83)	5,710 (4,260–7,250)	0·638 (0·479–0·809)	6,910 (5,180–8,680)	0·763 (0·571–0·953)
Bangladesh	1,070 (917–1,250)	1·28 (1·09–1·48)	1,510 (1,290–1,740)	1·89 (1·61–2·19)	48 (0·478–240)	0·0587 (0·00056–0·298)	59·6 (1·21–291)	0·0761 (0·00152–0·375)
Bhutan	1·52 (1·29–1·8)	0·396 (0·336–0·47)	3·19 (2·71–3·79)	0·737 (0·624–0·875)	0·229 (0·0524–0·457)	0·0626 (0·0147–0·125)	0·593 (0·177–1·17)	0·146 (0·0414–0·286)
India	34,100 (29,200–39,800)	4·84 (4·15–5·66)	48,900 (41,800–57,100)	6·78 (5·8–7·92)	5,500 (4,120–6,970)	0·789 (0·59–0·998)	6,530 (4,920–8,110)	0·909 (0·687–1·13)
Nepal	263 (225–306)	1·66 (1·42–1·93)	481 (412–560)	3·74 (3·2–4·35)	31·1 (1·98–69·9)	0·197 (0·012–0·44)	91·2 (8·11–184)	0·725 (0·0631–1·47)
Pakistan	1,020 (871–1,200)	1·01 (0·858–1·18)	2,200 (1,880–2,580)	2·19 (1·87–2·57)	129 (4·3–278)	0·128 (0·00389–0·274)	227 (14·8–495)	0·228 (0·0141–0·508)
**Southeast Asia, East Asia, and Oceania**	**81,500 (71,600–92,200)**	**7·32 (6·43–8·28)**	**138,000 (120,000–156,000)**	**11·7 (10·2–13·3)**	**4,420 (3,250–5,660)**	**0·38 (0·28–0·486)**	**8,270 (5,900–11,100)**	**0·666 (0·479–0·88)**
East Asia	6,810 (6,060–7,670)	0·779 (0·694–0·877)	18,700 (16,600–20,900)	2·05 (1·82–2·3)	611 (380–927)	0·0688 (0·043–0·105)	1,820 (1,130–2,760)	0·191 (0·119–0·291)
China	6,470 (5,750–7,270)	0·765 (0·679–0·859)	17,600 (15,700–19,800)	2 (1·78–2·25)	581 (361–880)	0·0673 (0·0419–0·102)	1,720 (1,080–2,610)	0·187 (0·117–0·283)

Democratic People's Republic of Korea	320 (284–359)	2·18 (1·93–2·45)	834 (739–936)	5·52 (4·89–6·2)	27·6 (1·46–58·4)	0·197 (0·0117–0·422)	73·4 (4·69–157)	0·481 (0·031–1·04)
Taiwan (Province of China)	18·8 (16·2–21·3)	0·129 (0·111–0·146)	185 (160–210)	1·31 (1·14–1·49)	2·52 (1·56–3·9)	0·017 (0·0105–0·0263)	23·8 (14·7–36·9)	0·16 (0·0988–0·248)
Oceania	832 (733–934)	13·8 (12·1–15·4)	607 (535–682)	10·1 (8·9–11·3)	184 (98·3–308)	3·15 (1·68–5·25)	152 (80·8–253)	2·59 (1·38–4·3)
American Samoa	0·048 (0·0413–0·0558)	0·185 (0·159–0·215)	0·0451 (0·0388–0·0525)	0·171 (0·147–0·199)	0·00731 (0·00448–0·0113)	0·0285 (0·0174–0·0439)	0·00633 (0·00387–0·00982)	0·024 (0·0147–0·0372)
Cook Islands	0·0748 (0·064–0·0876)	0·808 (0·692–0·947)	0·119 (0·101–0·139)	1·38 (1·18–1·61)	0·00988 (0·00256–0·0157)	0·101 (0·0278–0·161)	0·0179 (0·00672–0·0279)	0·192 (0·0713–0·299)
Federated States of Micronesia	3·04 (2·62–3·47)	6·49 (5·59–7·39)	4·64 (3·98–5·28)	9·7 (8·33–11·1)	0·596 (0·0384–1·04)	1·3 (0·0839–2·26)	0·949 (0·0508–1·68)	2·08 (0·102–3·69)
Fiji	2·99 (2·62–3·39)	0·656 (0·574–0·742)	2·69 (2·35–3·04)	0·572 (0·5–0·648)	0·663 (0·409–1·03)	0·145 (0·0898–0·226)	0·646 (0·399–1)	0·136 (0·0841–0·212)
Guam	0·377 (0·324–0·437)	0·468 (0·403–0·542)	0·814 (0·7–0·943)	0·938 (0·807–1·09)	0·0407 (0·0254–0·0631)	0·0493 (0·0307–0·0765)	0·101 (0·0629–0·157)	0·113 (0·0703–0·176)
Kiribati	1·03 (0·903–1·15)	1·68 (1·47–1·88)	0·796 (0·701–0·894)	1·44 (1·27–1·62)	0·325 (0·213–0·441)	0·511 (0·334–0·693)	0·261 (0·17–0·347)	0·431 (0·281–0·573)
Marshall Islands	0·554 (0·486–0·621)	2 (1·76–2·25)	0·509 (0·447–0·571)	1·77 (1·55–1·99)	0·0609 (0·0032–0·127)	0·228 (0·0113–0·476)	0·0531 (0·00375–0·11)	0·19 (0·0128–0·398)
Northern Mariana Islands	0·132 (0·112–0·156)	0·655 (0·556–0·776)	0·113 (0·0963–0·134)	0·502 (0·426–0·594)	0·0142 (0·009–0·0222)	0·0687 (0·0436–0·107)	0·0105 (0·00653–0·0163)	0·0443 (0·0278–0·0692)
Papua New Guinea	771 (679–866)	17·5 (15·5–19·7)	557 (491–626)	12·7 (11·2–14·3)	172 (91·2–290)	4·08 (2·16–6·87)	141 (73·9–238)	3·32 (1·77–5·6)
Republic of Nauru	0·115 (0·0979–0·134)	2·25 (1·91–2·62)	0·187 (0·159–0·218)	3·84 (3·26–4·48)	0·0134 (0·00152–0·022)	0·278 (0·0281–0·46)	0·0233 (0·00333–0·0368)	0·522 (0·0704–0·82)
Republic of Niue	0·0129 (0·0112–0·015)	1·59 (1·38–1·84)	0·0224 (0·0194–0·0259)	2·66 (2·3–3·08)	0·00167 (0·000222–0·00273)	0·195 (0·0273–0·318)	0·00328 (0·000611–0·00513)	0·369 (0·0696–0·578)
Republic of Palau	0·144 (0·122–0·17)	1·66 (1·4–1·96)	0·317 (0·268–0·374)	2·63 (2·23–3·1)	0·0206 (0·00227–0·0342)	0·214 (0·0263–0·354)	0·051 (0·00856–0·0801)	0·395 (0·0654–0·618)

Samoa	2·56 (2·2–2·92)	2·68 (2·31–3·07)	2·39 (2·05–2·73)	2·47 (2·12–2·82)	0·29 (0·0116–0·644)	0·323 (0·0123–0·725)	0·259 (0·0142–0·583)	0·277 (0·0139–0·636)
Solomon Islands	6·13 (5·33–7)	2·06 (1·79–2·36)	5 (4·34–5·71)	1·74 (1·51–1·99)	0·981 (0·0367–2·15)	0·342 (0·0128–0·77)	0·761 (0·0426–1·67)	0·265 (0·0134–0·595)
Tokelau	0·0105 (0·00914–0·012)	1·63 (1·41–1·85)	0·0179 (0·0156–0·0204)	2·73 (2·38–3·12)	0·0016 (0·000183–0·00269)	0·243 (0·0283–0·408)	0·00311 (0·000475–0·00491)	0·466 (0·0722–0·741)
Tonga	0·219 (0·19–0·249)	0·458 (0·396–0·519)	0·272 (0·236–0·309)	0·625 (0·541–0·709)	0·0413 (0·0257–0·0648)	0·0869 (0·0539–0·136)	0·0677 (0·0423–0·106)	0·158 (0·0984–0·247)
Tuvalu	0·116 (0·103–0·132)	2·09 (1·84–2·37)	0·212 (0·187–0·24)	3·51 (3·09–3·97)	0·0203 (0·00156–0·0356)	0·372 (0·0276–0·653)	0·0443 (0·00412–0·0706)	0·756 (0·0701–1·2)
Vanuatu	4·51 (3·98–5·03)	3·25 (2·88–3·63)	3·54 (3·13–3·95)	2·78 (2·46–3·1)	0·812 (0·0168–2)	0·622 (0·0128–1·55)	0·647 (0·0199–1·59)	0·535 (0·014–1·34)
Southeast Asia	73,800 (64,700–83,700)	20·6 (18·1–23·4)	118,000 (103,000–135,000)	32·7 (28·5–37·2)	3,630 (2,730–4,660)	1·02 (0·764–1·31)	6,290 (4,610–8,150)	1·74 (1·28–2·25)
Cambodia	1,460 (1,280–1,690)	17·9 (15·7–20·7)	1,430 (1,260–1,660)	20·3 (17·8–23·5)	174 (113–258)	2·11 (1·37–3·09)	282 (181–409)	3·91 (2·52–5·63)
Indonesia	53,400 (46,600–60,800)	38·8 (33·9–44·2)	86,300 (74,900–99,000)	61·2 (53·1–70·2)	1,020 (800–1,390)	0·785 (0·607–1·07)	1,500 (1,210–1,980)	1·11 (0·897–1·45)
Lao People's Democratic Republic	409 (351–485)	11·7 (10·1–13·9)	1,040 (889–1,230)	30·7 (26·3–36·4)	23·5 (0·208–136)	0·652 (0·00571–3·96)	91·3 (0·789–499)	2·73 (0·0224–15·3)
Malaysia	787 (672–921)	4·92 (4·2–5·76)	1,940 (1,660–2,280)	10·8 (9·26–12·7)	112 (69·4–171)	0·766 (0·472–1·16)	182 (114–273)	1·11 (0·695–1·67)
Maldives	1·32 (1·12–1·56)	0·642 (0·545–0·759)	0·646 (0·549–0·764)	0·2 (0·17–0·236)	0·234 (0·147–0·312)	0·115 (0·0723–0·153)	0·0633 (0·0398–0·0847)	0·0206 (0·013–0·0277)
Mauritius	3·02 (2·62–3·53)	0·43 (0·373–0·502)	10·3 (8·92–12)	1·37 (1·19–1·6)	0·342 (0·211–0·529)	0·0478 (0·0295–0·0739)	1·78 (1·1–2·75)	0·233 (0·144–0·36)
Myanmar	7,280 (6,460–8,160)	24·3 (21·6–27·3)	6,520 (5,780–7,310)	24·6 (21·9–27·6)	1,220 (755–1,760)	4·05 (2·49–5·87)	831 (664–1,100)	3·11 (2·48–4·14)
Philippines	142 (125–162)	0·248 (0·219–0·283)	497 (439–567)	0·865 (0·764–0·987)	34·1 (20·9–52·1)	0·0618 (0·0379–0·0943)	57·8 (35·5–87·8)	0·1 (0·0615–0·152)

Seychelles	0·678 (0·586–0·787)	1·31 (1·13–1·52)	0·802 (0·692–0·93)	1·25 (1·08–1·45)	0·156 (0·0966–0·238)	0·285 (0·177–0·436)	0·143 (0·0888–0·219)	0·22 (0·136–0·336)
Socialist Republic of Viet Nam	2,980 (2,650–3,310)	5·33 (4·72–5·9)	11,100 (9,880–12,300)	19·8 (17·5–21·9)	192 (121–263)	0·34 (0·215–0·467)	1,980 (1,220–2,680)	3·45 (2·13–4·65)
Sri Lanka	17·3 (14·8–20·2)	0·146 (0·125–0·171)	34·3 (29·3–40·2)	0·301 (0·258–0·353)	2·99 (1·82–4·57)	0·0267 (0·0162–0·041)	5·01 (3·03–7·75)	0·0454 (0·0276–0·0701)
Thailand	7,270 (6,220–8,660)	16·6 (14·2–19·7)	9,330 (7,990–11,100)	22·8 (19·5–27·1)	843 (516–1,300)	1·85 (1·14–2·81)	1,350 (825–2,080)	3·09 (1·89–4·73)
Timor-Leste	5·99 (5·27–6·75)	0·982 (0·864–1·11)	8·62 (7·58–9·71)	1·52 (1·34–1·72)	1·75 (0·295–3·24)	0·3 (0·0523–0·555)	2·51 (0·554–4·46)	0·455 (0·0979–0·809)
**Sub-Saharan Africa**	**481,000 (418,000–552,000)**	**110 (96·1–127)**	**313,000 (273,000–356,000)**	**81·6 (71·4–92·9)**	**98,800 (68,700–127,000)**	**23·3 (16·1–29·8)**	**84,700 (58,500–110,000)**	**22·2 (15·4–28·6)**
Central Sub-Saharan Africa	29,400 (25,800–33,300)	57·3 (50·3–64·8)	14,300 (12,500–16,100)	29·3 (25·7–33·2)	7,020 (5,470–8,920)	14·2 (11–18·1)	3,800 (3,010–4,740)	7·9 (6·32–9·82)
Angola	6,850 (5,940–7,850)	56·6 (49–64·8)	3,080 (2,670–3,530)	29·8 (25·9–34·2)	1,930 (1,160–3,070)	16·9 (10·1–26·8)	841 (506–1,340)	8·24 (4·96–13·1)
Central African Republic	6,240 (5,570–6,990)	289 (258–324)	3,190 (2,830–3,580)	162 (144–182)	1,520 (1,180–2,070)	74·9 (59·2–98·3)	973 (767–1,290)	51·9 (41·1–66·9)
Congo	1,700 (1,460–1,960)	69·5 (59·7–80·3)	876 (752–1,010)	36·5 (31·4–42·2)	457 (256–747)	19·5 (10·9–31·9)	270 (151–442)	11·5 (6·47–18·9)
Democratic Republic of the Congo	13,200 (11,400–15,000)	40 (34·6–45·4)	6,450 (5,590–7,330)	19·6 (17–22·2)	2,870 (2,170–3,820)	8·76 (6·61–11·6)	1,590 (1,240–2,060)	4·72 (3·62–6·21)
Equatorial Guinea	465 (393–548)	82·7 (69·9–97·5)	254 (215–300)	47·5 (40·3–56)	72·3 (39–124)	13·5 (7·31–23·1)	50·7 (27·4–86·7)	10·4 (5·61–17·8)
Gabon	962 (820–1,120)	116 (98·7–135)	416 (358–483)	56·6 (48·7–65·7)	171 (95·6–270)	21·3 (11·9–33·7)	71·6 (40·2–114)	9·81 (5·5–15·5)
Eastern Sub-Saharan Africa	195,000 (168,000–226,000)	121 (104–140)	126,000 (109,000–146,000)	86·2 (74·7–99·7)	41,600 (29,400–52,500)	25·5 (18–31·7)	38,300 (27,000–47,900)	25·8 (18·3–31·8)
Burundi	3,890 (3,330–4,570)	96·7 (82·8–113)	2,320 (1,980–2,720)	54·5 (46·6–63·9)	468 (372–598)	11·2 (8·58–14·7)	450 (363–574)	10·3 (8·03–13·9)

Comoros	11·9 (10·1–14)	3·48 (2·96–4·11)	31 (26·4–36·6)	9·52 (8·09–11·2)	0·16 (0·00237–1·38)	0·0454 (0·000703–0·398)	0·498 (0·00746–4·65)	0·148 (0·00227–1·34)
Djibouti	275 (232–327)	48·2 (40·7–57·3)	184 (156–219)	30·7 (26–36·5)	66·8 (38·9–108)	12·4 (7·22–20·1)	45·1 (26·3–73·2)	7·94 (4·62–12·9)
Eritrea	1,120 (956–1,320)	41·5 (35·3–48·7)	652 (554–765)	25·4 (21·6–29·8)	284 (176–402)	11 (6·78–15·6)	160 (99·1–230)	6·48 (3·99–9·28)
Ethiopia	18,000 (15,500–20,800)	45·7 (39·3–52·6)	11,100 (9,540–12,800)	28·5 (24·5–32·8)	3,480 (2,130–5,070)	8·8 (5·36–12·9)	2,270 (1,380–3,300)	5·61 (3·41–8·17)
Kenya	27,200 (23,100–32,000)	133 (113–156)	16,700 (14,200–19,500)	87·5 (74·7–102)	4,520 (2,700–6,590)	22·8 (13·5–33·4)	4,220 (2,490–6,310)	22·9 (13·5–34·2)
Madagascar	264 (225–313)	2·26 (1·93–2·68)	168 (143–199)	1·56 (1·33–1·85)	79·6 (47·8–122)	0·739 (0·443–1·14)	48·3 (29–74·3)	0·475 (0·285–0·73)
Malawi	18,400 (15,500–21,800)	263 (219–315)	12,300 (10,600–14,400)	205 (176–240)	3,390 (2,710–4,230)	50 (39·8–60·7)	2,920 (2,360–3,770)	48·6 (38·3–64·1)
Mozambique	46,200 (38,800–54,500)	376 (316–446)	29,700 (25,100–34,900)	291 (247–342)	14,000 (9,510–18,400)	113 (76·5–148)	12,700 (8,620–16,600)	124 (83·2–166)
Rwanda	4,120 (3,520–4,850)	79·3 (67·6–93·3)	2,300 (1,960–2,720)	53·4 (45·5–63·2)	809 (582–1,010)	15·9 (11·5–19·7)	471 (342–594)	11·1 (8·09–14·3)
Somalia	1,730 (1,450–2,090)	22·3 (18·7–26·9)	1,240 (1,040–1,500)	16·3 (13·6–19·6)	748 (504–996)	10·3 (6·94–13·7)	507 (343–730)	7·06 (4·72–10·1)
South Sudan	1,590 (1,370–1,850)	42·9 (37·1–49·9)	962 (830–1,120)	29·7 (25·6–34·5)	591 (350–918)	16·7 (9·89–26)	424 (249–658)	13·1 (7·71–20·4)
Uganda	25,200 (22,200–28,800)	166 (146–191)	17,200 (15,100–19,400)	141 (124–159)	4,340 (3,220–5,940)	27·5 (21–37·1)	4,650 (3,480–6,170)	36 (26·1–49·2)
United Republic of Tanzania	25,000 (21,300–29,200)	114 (96·8–133)	14,700 (12,700–17,200)	75·1 (64·5–87·7)	4,370 (2,670–6,050)	19·7 (12–27·1)	4,810 (2,940–6,600)	24·6 (15·1–34·1)
Zambia	21,500 (18,000–25,700)	305 (254–366)	16,400 (13,900–19,200)	257 (218–302)	4,430 (2,940–5,740)	62·2 (41·1–80·2)	4,590 (3,070–5,920)	69·8 (46·5–90·4)
Southern Sub-Saharan Africa	166,000 (145,000–190,000)	401 (349–458)	118,000 (103,000–135,000)	323 (283–368)	31,200 (21,900–41,600)	78·2 (54·8–104)	27,100 (18,600–36,600)	76·1 (51·9–103)

Botswana	5,370 (4,410–6,540)	439 (363–536)	4,170 (3,520–4,950)	398 (337–474)	774 (437–1,180)	67·3 (38–103)	801 (458–1,190)	79·1 (45·1–117)
Kingdom of Eswatini	3,300 (2,730–3,960)	588 (492–706)	2,530 (2,130–2,980)	575 (487–674)	722 (574–859)	139 (110–163)	729 (553–967)	173 (131–229)
Lesotho	8,800 (7,390–10,400)	857 (725–1,010)	7,350 (6,230–8,640)	801 (682–939)	2,570 (1,940–3,410)	266 (205–345)	2,270 (1,750–3,020)	260 (205–336)
Namibia	3,600 (3,020–4,280)	324 (272–387)	2,510 (2,130–2,930)	266 (227–312)	591 (343–876)	55 (31·8–81·8)	526 (307–767)	56·7 (33·1–83·1)
South Africa	120,000 (104,000–138,000)	393 (342–450)	83,700 (72,800–94,700)	299 (260–339)	21,200 (12,400–30,700)	71·2 (41·6–103)	18,900 (11,100–27,500)	69·5 (40·7–102)
Zimbabwe	24,800 (21,000–29,600)	372 (314–445)	18,200 (15,400–21,500)	342 (290–404)	5,370 (4,530–6,390)	85·6 (70·7–101)	3,950 (3,310–4,930)	77·1 (62·4–99·6)
Western Sub-Saharan Africa	90,200 (78,400–104,000)	50·5 (43·9–58·4)	53,800 (46,800–62,200)	34·4 (29·9–39·7)	18,900 (11,800–27,400)	10·9 (6·79–15·8)	15,500 (9,850–22,100)	10·2 (6·44–14·5)
Benin	1,240 (1,070–1,440)	26·8 (23·2–31)	662 (574–768)	15·7 (13·6–18·2)	244 (143–370)	5·12 (2·99–7·81)	251 (147–379)	6 (3·51–9·08)
Burkina Faso	3,310 (2,870–3,810)	38·6 (33·4–44·4)	1,910 (1,660–2,200)	25·5 (22·1–29·4)	580 (433–748)	6·5 (4·87–8·43)	670 (511–834)	8·99 (6·84–11·4)
Cameroon	10,900 (9,290–12,800)	92·2 (78·5–108)	5,920 (5,080–6,930)	53·9 (46·2–63·1)	2,460 (1,330–4,000)	22·4 (12·1–36·5)	1,830 (994–2,990)	18·1 (9·71–29·5)
Chad	3,820 (3,300–4,400)	69·9 (60·5–80·7)	2,510 (2,170–2,890)	50·7 (43·9–58·5)	972 (645–1,350)	18 (12·1–24·7)	1,010 (713–1,370)	20·9 (14·9–28·9)
Ghana	10,400 (8,940–12,200)	70·3 (60·4–82·2)	4,690 (4,030–5,480)	37·4 (32·2–43·7)	2,360 (1,400–3,600)	16·6 (9·75–25·5)	1,430 (851–2,200)	12 (7·07–18·5)
Guinea	3,030 (2,610–3,480)	59·6 (51·3–68·5)	1,570 (1,350–1,800)	38·6 (33·3–44·4)	855 (542–1,200)	17·7 (11·1–24·7)	604 (381–841)	15·2 (9·56–21·1)
Guinea-Bissau	847 (720–995)	107 (90–125)	482 (410–561)	73·4 (62·5–85·5)	189 (114–282)	24·7 (14·9–36·6)	226 (139–331)	37 (22·2–54·4)
Liberia	280 (242–326)	13·7 (11·8–15·9)	175 (151–204)	8·7 (7·5–10·1)	55·9 (32·4–87·9)	2·87 (1·66–4·5)	61·1 (35·4–96)	3·2 (1·85–5·03)

Mali	2,420 (2,090–2,800)	31·2 (27–36·1)	1,440 (1,240–1,660)	20·3 (17·5–23·5)	632 (378–967)	8·46 (5·05–13)	491 (293–750)	7·16 (4·27–10·9)
Mauritania	81·6 (69·9–95·7)	5·13 (4·4–6·02)	175 (150–205)	12·4 (10·6–14·5)	1·26 (0·0421–7·38)	0·0715 (0·00261–0·409)	4·67 (0·149–38·7)	0·318 (0·0108–2·53)
Niger	1,220 (1,070–1,420)	16·8 (14·7–19·5)	910 (793–1,050)	14·1 (12·3–16·4)	286 (190–389)	4·05 (2·63–5·53)	298 (207–397)	4·91 (3·39–6·72)
Nigeria	42,500 (36,900–49,200)	50·5 (43·9–58·5)	27,900 (24,200–32,300)	39·4 (34·2–45·6)	8,030 (5,030–12,000)	9·77 (6·1–14·7)	6,530 (4,150–9,680)	9·4 (5·97–13·8)
Republic of Cabo Verde	20·5 (17·5–24)	7·4 (6·34–8·67)	14·5 (12·4–17)	5·25 (4·5–6·15)	2·71 (1·64–4·34)	1 (0·605–1·6)	3·55 (2·13–5·73)	1·33 (0·799–2·14)
Republic of Côte d'Ivoire	6,170 (5,320–7,140)	62·8 (54·3–72·8)	3,320 (2,870–3,840)	31·1 (26·9–36)	1,450 (817–2,310)	14·8 (8·31–23·7)	1,380 (779–2,210)	13·2 (7·42–21·2)
Republic of the Gambia	457 (394–534)	50·3 (43·3–58·7)	282 (243–330)	34·8 (29·9–40·6)	91 (53·2–144)	10·7 (6·24–16·9)	88·2 (51·6–137)	11·8 (6·87–18·3)
Sao Tome and Principe	2·3 (1·98–2·69)	2·7 (2·33–3·16)	6·65 (5·73–7·79)	8·26 (7·12–9·67)	0·0298 (0·0139–0·0495)	0·0316 (0·0156–0·0512)	0·101 (0·0534–0·165)	0·115 (0·0617–0·189)
Senegal	1,030 (886–1,200)	17·1 (14·7–20)	509 (438–595)	9·26 (7·97–10·8)	237 (141–356)	4·03 (2·4–6·1)	150 (89·5–229)	2·79 (1·67–4·26)
Sierra Leone	1,060 (912–1,230)	29·8 (25·8–34·7)	665 (575–773)	20·7 (17·9–24)	203 (117–324)	6·09 (3·51–9·7)	202 (116–322)	6·75 (3·89–10·8)
Togo	1,370 (1,180–1,600)	39·4 (33·9–46·2)	697 (601–817)	23·3 (20·1–27·4)	304 (170–488)	8·91 (4·99–14·3)	259 (145–418)	8·94 (5·01–14·5)

eTable 8. All-age population-attributable fractions of tuberculosis due to alcohol use, diabetes, and smoking among HIV-negative men and women with 95% uncertainty intervals for 204 countries and territories in 2019

	Smoking		Alcohol use		Diabetes	
	Male	Female	Male	Female	Male	Female
**Afghanistan**	0.16 (0.13 - 0.19)	0.02 (0.02 - 0.03)	0.01 (0.00 - 0.02)	0.00 (0.00 - 0.00)	0.12 (0.08 - 0.16)	0.13 (0.09 - 0.19)
**Albania**	0.43 (0.37 - 0.49)	0.10 (0.07 - 0.13)	0.35 (0.23 - 0.45)	0.08 (0.04 - 0.13)	0.09 (0.04 - 0.14)	0.07 (0.04 - 0.12)
**Algeria**	0.31 (0.26 - 0.36)	0.02 (0.02 - 0.03)	0.08 (0.04 - 0.11)	0.01 (0.01 - 0.02)	0.15 (0.09 - 0.22)	0.16 (0.09 - 0.23)
**American Samoa**	0.28 (0.22 - 0.33)	0.12 (0.08 - 0.16)	0.09 (0.02 - 0.17)	0.01 (0.00 - 0.01)	0.30 (0.20 - 0.40)	0.27 (0.18 - 0.36)
**Andorra**	0.25 (0.20 - 0.30)	0.11 (0.08 - 0.15)	0.48 (0.36 - 0.58)	0.29 (0.18 - 0.39)	0.12 (0.06 - 0.18)	0.10 (0.05 - 0.17)
**Angola**	0.18 (0.14 - 0.21)	0.02 (0.02 - 0.03)	0.37 (0.26 - 0.45)	0.22 (0.14 - 0.29)	0.10 (0.06 - 0.14)	0.06 (0.04 - 0.09)
**Antigua and Barbuda**	0.15 (0.12 - 0.18)	0.06 (0.04 - 0.07)	0.35 (0.24 - 0.44)	0.13 (0.07 - 0.19)	0.17 (0.10 - 0.23)	0.16 (0.10 - 0.23)
**Argentina**	0.26 (0.21 - 0.30)	0.18 (0.15 - 0.22)	0.48 (0.35 - 0.58)	0.29 (0.19 - 0.38)	0.11 (0.07 - 0.17)	0.10 (0.06 - 0.14)
**Armenia**	0.47 (0.41 - 0.53)	0.03 (0.02 - 0.04)	0.29 (0.19 - 0.39)	0.16 (0.10 - 0.23)	0.11 (0.07 - 0.16)	0.11 (0.07 - 0.15)
**Australia**	0.10 (0.08 - 0.12)	0.10 (0.08 - 0.13)	0.48 (0.35 - 0.58)	0.29 (0.18 - 0.39)	0.11 (0.05 - 0.19)	0.09 (0.04 - 0.16)
**Austria**	0.31 (0.26 - 0.37)	0.15 (0.12 - 0.18)	0.51 (0.38 - 0.60)	0.30 (0.20 - 0.41)	0.12 (0.06 - 0.18)	0.10 (0.05 - 0.17)
**Azerbaijan**	0.41 (0.35 - 0.47)	0.02 (0.01 - 0.03)	0.40 (0.27 - 0.50)	0.14 (0.07 - 0.22)	0.09 (0.06 - 0.13)	0.09 (0.06 - 0.13)
**Bahamas**	0.15 (0.11 - 0.19)	0.04 (0.03 - 0.06)	0.34 (0.17 - 0.47)	0.12 (0.04 - 0.22)	0.15 (0.10 - 0.20)	0.13 (0.09 - 0.18)
**Bahrain**	0.28 (0.22 - 0.34)	0.05 (0.04 - 0.08)	0.12 (0.07 - 0.17)	0.02 (0.01 - 0.03)	0.25 (0.16 - 0.34)	0.23 (0.14 - 0.31)

**Bangladesh**	0.27 (0.22 - 0.32)	0.02 (0.02 - 0.03)	0.03 (0.00 - 0.06)	0.01 (0.00 - 0.01)	0.10 (0.06 - 0.14)	0.09 (0.06 - 0.14)
**Barbados**	0.15 (0.11 - 0.18)	0.03 (0.02 - 0.04)	0.41 (0.29 - 0.50)	0.12 (0.07 - 0.18)	0.15 (0.09 - 0.23)	0.16 (0.09 - 0.23)
**Belarus**	0.45 (0.39 - 0.51)	0.13 (0.10 - 0.16)	0.55 (0.41 - 0.64)	0.26 (0.16 - 0.35)	0.07 (0.04 - 0.10)	0.06 (0.04 - 0.09)
**Belgium**	0.26 (0.21 - 0.31)	0.13 (0.11 - 0.17)	0.49 (0.36 - 0.59)	0.35 (0.24 - 0.46)	0.12 (0.06 - 0.18)	0.11 (0.05 - 0.18)
**Belize**	0.19 (0.15 - 0.23)	0.04 (0.03 - 0.05)	0.38 (0.26 - 0.47)	0.10 (0.06 - 0.15)	0.10 (0.06 - 0.14)	0.11 (0.07 - 0.15)
**Benin**	0.10 (0.07 - 0.13)	0.01 (0.01 - 0.02)	0.20 (0.12 - 0.28)	0.09 (0.04 - 0.14)	0.07 (0.04 - 0.10)	0.07 (0.04 - 0.11)
**Bermuda**	0.15 (0.12 - 0.19)	0.07 (0.05 - 0.09)	0.37 (0.26 - 0.47)	0.15 (0.09 - 0.22)	0.13 (0.07 - 0.21)	0.11 (0.05 - 0.17)
**Bhutan**	0.14 (0.10 - 0.18)	0.04 (0.03 - 0.05)	0.12 (0.05 - 0.19)	0.01 (0.00 - 0.02)	0.10 (0.06 - 0.15)	0.10 (0.06 - 0.15)
**Bolivia (Plurinational State of)**	0.13 (0.09 - 0.16)	0.02 (0.01 - 0.03)	0.31 (0.20 - 0.41)	0.10 (0.04 - 0.16)	0.10 (0.06 - 0.14)	0.09 (0.05 - 0.14)
**Bosnia and Herzegovina**	0.40 (0.35 - 0.46)	0.18 (0.14 - 0.22)	0.41 (0.29 - 0.50)	0.08 (0.04 - 0.13)	0.18 (0.10 - 0.27)	0.16 (0.07 - 0.25)
**Botswana**	0.26 (0.21 - 0.30)	0.06 (0.04 - 0.07)	0.37 (0.26 - 0.47)	0.11 (0.06 - 0.17)	0.09 (0.06 - 0.13)	0.09 (0.06 - 0.13)
**Brazil**	0.19 (0.16 - 0.23)	0.10 (0.08 - 0.13)	0.37 (0.25 - 0.45)	0.14 (0.08 - 0.19)	0.11 (0.07 - 0.16)	0.10 (0.06 - 0.15)
**Brunei Darussalam**	0.27 (0.22 - 0.33)	0.07 (0.05 - 0.09)	0.03 (0.00 - 0.07)	0.01 (0.00 - 0.03)	0.26 (0.16 - 0.36)	0.18 (0.11 - 0.26)
**Bulgaria**	0.38 (0.32 - 0.43)	0.14 (0.11 - 0.17)	0.53 (0.40 - 0.63)	0.22 (0.13 - 0.31)	0.13 (0.08 - 0.20)	0.11 (0.06 - 0.18)
**Burkina Faso**	0.10 (0.07 - 0.12)	0.01 (0.00 - 0.01)	0.31 (0.20 - 0.40)	0.21 (0.12 - 0.30)	0.06 (0.04 - 0.09)	0.06 (0.04 - 0.09)
**Burundi**	0.12 (0.08 - 0.16)	0.02 (0.01 - 0.03)	0.40 (0.27 - 0.50)	0.11 (0.06 - 0.16)	0.06 (0.04 - 0.09)	0.05 (0.03 - 0.08)

**Cabo Verde**	0.11 (0.08 - 0.13)	0.02 (0.01 - 0.03)	0.33 (0.22 - 0.42)	0.16 (0.09 - 0.23)	0.10 (0.06 - 0.14)	0.13 (0.07 - 0.20)
**Cambodia**	0.40 (0.34 - 0.46)	0.06 (0.04 - 0.08)	0.41 (0.29 - 0.52)	0.11 (0.06 - 0.18)	0.12 (0.07 - 0.17)	0.11 (0.07 - 0.17)
**Cameroon**	0.13 (0.10 - 0.16)	0.01 (0.01 - 0.01)	0.33 (0.23 - 0.42)	0.18 (0.11 - 0.25)	0.07 (0.04 - 0.10)	0.07 (0.04 - 0.10)
**Canada**	0.19 (0.16 - 0.24)	0.14 (0.11 - 0.17)	0.39 (0.27 - 0.49)	0.26 (0.16 - 0.36)	0.11 (0.05 - 0.17)	0.09 (0.04 - 0.15)
**Central African Republic**	0.12 (0.09 - 0.15)	0.01 (0.01 - 0.02)	0.21 (0.11 - 0.31)	0.09 (0.04 - 0.15)	0.10 (0.07 - 0.14)	0.07 (0.04 - 0.09)
**Chad**	0.10 (0.07 - 0.13)	0.01 (0.01 - 0.02)	0.17 (0.08 - 0.25)	0.10 (0.03 - 0.18)	0.06 (0.03 - 0.09)	0.06 (0.03 - 0.09)
**Chile**	0.17 (0.14 - 0.21)	0.11 (0.08 - 0.14)	0.46 (0.33 - 0.56)	0.27 (0.17 - 0.37)	0.14 (0.08 - 0.21)	0.14 (0.07 - 0.22)
**China**	0.42 (0.37 - 0.48)	0.05 (0.04 - 0.06)	0.38 (0.27 - 0.48)	0.07 (0.04 - 0.11)	0.10 (0.06 - 0.15)	0.09 (0.05 - 0.14)
**Colombia**	0.12 (0.09 - 0.14)	0.06 (0.04 - 0.08)	0.29 (0.20 - 0.37)	0.07 (0.04 - 0.12)	0.15 (0.09 - 0.21)	0.12 (0.07 - 0.18)
**Comoros**	0.16 (0.12 - 0.21)	0.02 (0.01 - 0.03)	0.05 (0.01 - 0.12)	0.02 (0.00 - 0.06)	0.07 (0.04 - 0.10)	0.06 (0.03 - 0.08)
**Congo**	0.17 (0.13 - 0.21)	0.01 (0.01 - 0.02)	0.33 (0.20 - 0.44)	0.21 (0.11 - 0.31)	0.11 (0.07 - 0.16)	0.09 (0.06 - 0.13)
**Cook Islands**	0.24 (0.19 - 0.29)	0.11 (0.07 - 0.14)	0.41 (0.28 - 0.52)	0.06 (0.02 - 0.12)	0.23 (0.13 - 0.32)	0.22 (0.13 - 0.32)
**Costa Rica**	0.18 (0.15 - 0.22)	0.06 (0.05 - 0.08)	0.31 (0.21 - 0.40)	0.09 (0.04 - 0.13)	0.17 (0.10 - 0.24)	0.13 (0.08 - 0.20)
**Croatia**	0.33 (0.28 - 0.39)	0.16 (0.13 - 0.20)	0.50 (0.36 - 0.59)	0.18 (0.10 - 0.27)	0.15 (0.09 - 0.23)	0.13 (0.06 - 0.21)
**Cuba**	0.31 (0.25 - 0.36)	0.12 (0.09 - 0.16)	0.33 (0.22 - 0.41)	0.10 (0.06 - 0.14)	0.17 (0.10 - 0.25)	0.13 (0.07 - 0.20)
**Cyprus**	0.31 (0.26 - 0.37)	0.09 (0.06 - 0.11)	0.48 (0.34 - 0.58)	0.16 (0.08 - 0.25)	0.16 (0.08 - 0.26)	0.16 (0.07 - 0.26)

**Czechia**	0.32 (0.27 - 0.37)	0.14 (0.11 - 0.17)	0.55 (0.42 - 0.64)	0.30 (0.20 - 0.40)	0.22 (0.13 - 0.31)	0.17 (0.08 - 0.28)
**CÃ´te d'Ivoire**	0.17 (0.14 - 0.21)	0.03 (0.02 - 0.04)	0.34 (0.23 - 0.44)	0.14 (0.07 - 0.23)	0.07 (0.04 - 0.10)	0.07 (0.04 - 0.11)
**Democratic People's Republic of Korea**	0.38 (0.32 - 0.44)	0.05 (0.03 - 0.07)	0.34 (0.23 - 0.45)	0.04 (0.02 - 0.07)	0.09 (0.06 - 0.14)	0.09 (0.05 - 0.13)
**Democratic Republic of the Congo**	0.11 (0.07 - 0.15)	0.01 (0.01 - 0.01)	0.18 (0.09 - 0.28)	0.08 (0.03 - 0.15)	0.10 (0.06 - 0.14)	0.07 (0.04 - 0.10)
**Denmark**	0.31 (0.25 - 0.37)	0.22 (0.17 - 0.26)	0.48 (0.35 - 0.58)	0.37 (0.25 - 0.47)	0.10 (0.05 - 0.16)	0.09 (0.04 - 0.16)
**Djibouti**	0.25 (0.18 - 0.31)	0.03 (0.02 - 0.04)	0.05 (0.01 - 0.10)	0.01 (0.00 - 0.03)	0.07 (0.04 - 0.10)	0.06 (0.04 - 0.09)
**Dominica**	0.14 (0.11 - 0.17)	0.04 (0.02 - 0.05)	0.37 (0.26 - 0.46)	0.14 (0.08 - 0.21)	0.20 (0.13 - 0.28)	0.15 (0.09 - 0.22)
**Dominican Republic**	0.18 (0.14 - 0.22)	0.10 (0.07 - 0.12)	0.36 (0.25 - 0.45)	0.17 (0.10 - 0.24)	0.08 (0.05 - 0.11)	0.07 (0.05 - 0.10)
**Ecuador**	0.13 (0.09 - 0.16)	0.03 (0.02 - 0.04)	0.29 (0.19 - 0.37)	0.07 (0.04 - 0.11)	0.10 (0.06 - 0.15)	0.11 (0.06 - 0.16)
**Egypt**	0.36 (0.31 - 0.42)	0.01 (0.01 - 0.02)	0.04 (0.02 - 0.08)	0.00 (0.00 - 0.01)	0.11 (0.07 - 0.16)	0.11 (0.07 - 0.16)
**El Salvador**	0.13 (0.10 - 0.16)	0.04 (0.02 - 0.05)	0.26 (0.17 - 0.33)	0.07 (0.04 - 0.11)	0.15 (0.09 - 0.22)	0.13 (0.08 - 0.20)
**Equatorial Guinea**	0.15 (0.10 - 0.19)	0.01 (0.01 - 0.02)	0.38 (0.25 - 0.48)	0.24 (0.14 - 0.33)	0.10 (0.07 - 0.14)	0.09 (0.05 - 0.13)
**Eritrea**	0.14 (0.10 - 0.18)	0.00 (0.00 - 0.01)	0.16 (0.07 - 0.25)	0.05 (0.02 - 0.09)	0.07 (0.04 - 0.09)	0.07 (0.04 - 0.10)
**Estonia**	0.38 (0.32 - 0.44)	0.16 (0.13 - 0.20)	0.56 (0.43 - 0.65)	0.29 (0.19 - 0.38)	0.10 (0.06 - 0.14)	0.08 (0.05 - 0.12)
**Eswatini**	0.10 (0.07 - 0.13)	0.02 (0.01 - 0.03)	0.35 (0.25 - 0.44)	0.11 (0.06 - 0.16)	0.09 (0.06 - 0.12)	0.09 (0.05 - 0.13)
**Ethiopia**	0.07 (0.05 - 0.09)	0.01 (0.00 - 0.01)	0.22 (0.12 - 0.32)	0.09 (0.04 - 0.16)	0.05 (0.03 - 0.07)	0.05 (0.03 - 0.07)

**Fiji**	0.26 (0.20 - 0.31)	0.07 (0.05 - 0.10)	0.25 (0.16 - 0.33)	0.02 (0.01 - 0.04)	0.27 (0.17 - 0.37)	0.24 (0.15 - 0.32)
**Finland**	0.16 (0.13 - 0.20)	0.08 (0.06 - 0.11)	0.39 (0.28 - 0.50)	0.21 (0.12 - 0.30)	0.14 (0.07 - 0.23)	0.14 (0.06 - 0.24)
**France**	0.18 (0.14 - 0.22)	0.07 (0.05 - 0.10)	0.52 (0.38 - 0.61)	0.33 (0.21 - 0.44)	0.08 (0.03 - 0.13)	0.07 (0.03 - 0.13)
**Gabon**	0.15 (0.11 - 0.19)	0.02 (0.01 - 0.02)	0.42 (0.30 - 0.52)	0.27 (0.17 - 0.36)	0.14 (0.09 - 0.19)	0.10 (0.06 - 0.14)
**Gambia**	0.17 (0.14 - 0.21)	0.01 (0.01 - 0.01)	0.24 (0.14 - 0.32)	0.10 (0.05 - 0.16)	0.07 (0.04 - 0.11)	0.08 (0.05 - 0.13)
**Georgia**	0.45 (0.39 - 0.51)	0.06 (0.04 - 0.08)	0.44 (0.31 - 0.53)	0.06 (0.02 - 0.11)	0.14 (0.09 - 0.19)	0.11 (0.07 - 0.15)
**Germany**	0.23 (0.19 - 0.28)	0.12 (0.09 - 0.15)	0.54 (0.41 - 0.63)	0.39 (0.27 - 0.49)	0.16 (0.08 - 0.25)	0.16 (0.07 - 0.25)
**Ghana**	0.09 (0.07 - 0.12)	0.02 (0.01 - 0.03)	0.34 (0.22 - 0.44)	0.13 (0.06 - 0.20)	0.09 (0.06 - 0.13)	0.09 (0.05 - 0.13)
**Greece**	0.32 (0.26 - 0.37)	0.13 (0.10 - 0.16)	0.46 (0.34 - 0.56)	0.12 (0.05 - 0.18)	0.12 (0.06 - 0.20)	0.10 (0.04 - 0.17)
**Greenland**	0.31 (0.26 - 0.37)	0.28 (0.21 - 0.34)	0.36 (0.21 - 0.48)	0.21 (0.10 - 0.33)	0.13 (0.07 - 0.19)	0.08 (0.04 - 0.12)
**Grenada**	0.15 (0.11 - 0.19)	0.04 (0.03 - 0.05)	0.42 (0.30 - 0.51)	0.18 (0.11 - 0.25)	0.18 (0.11 - 0.26)	0.16 (0.10 - 0.22)
**Guam**	0.22 (0.17 - 0.27)	0.12 (0.08 - 0.15)	0.20 (0.02 - 0.38)	0.02 (0.00 - 0.06)	0.15 (0.09 - 0.22)	0.14 (0.08 - 0.20)
**Guatemala**	0.13 (0.09 - 0.17)	0.03 (0.02 - 0.04)	0.23 (0.15 - 0.30)	0.06 (0.03 - 0.09)	0.14 (0.09 - 0.19)	0.13 (0.09 - 0.18)
**Guinea**	0.18 (0.14 - 0.22)	0.02 (0.01 - 0.02)	0.12 (0.06 - 0.18)	0.03 (0.01 - 0.06)	0.06 (0.04 - 0.10)	0.06 (0.04 - 0.10)
**Guinea-Bissau**	0.09 (0.07 - 0.11)	0.01 (0.01 - 0.01)	0.27 (0.17 - 0.37)	0.12 (0.06 - 0.17)	0.07 (0.04 - 0.10)	0.08 (0.05 - 0.11)
**Guyana**	0.20 (0.15 - 0.23)	0.03 (0.02 - 0.05)	0.44 (0.32 - 0.54)	0.07 (0.03 - 0.11)	0.18 (0.12 - 0.25)	0.16 (0.11 - 0.22)

**Haiti**	0.08 (0.06 - 0.11)	0.02 (0.01 - 0.03)	0.34 (0.23 - 0.42)	0.11 (0.06 - 0.15)	0.12 (0.08 - 0.16)	0.13 (0.09 - 0.17)
**Honduras**	0.21 (0.16 - 0.26)	0.05 (0.03 - 0.07)	0.25 (0.16 - 0.33)	0.05 (0.03 - 0.07)	0.17 (0.10 - 0.24)	0.14 (0.09 - 0.21)
**Hungary**	0.28 (0.24 - 0.33)	0.11 (0.09 - 0.14)	0.52 (0.39 - 0.62)	0.17 (0.10 - 0.26)	0.16 (0.09 - 0.25)	0.12 (0.06 - 0.20)
**Iceland**	0.22 (0.17 - 0.29)	0.13 (0.09 - 0.17)	0.42 (0.30 - 0.53)	0.24 (0.14 - 0.33)	0.12 (0.06 - 0.20)	0.10 (0.04 - 0.17)
**India**	0.23 (0.19 - 0.27)	0.04 (0.03 - 0.06)	0.25 (0.16 - 0.33)	0.02 (0.01 - 0.04)	0.14 (0.09 - 0.20)	0.11 (0.07 - 0.16)
**Indonesia**	0.34 (0.28 - 0.39)	0.03 (0.02 - 0.04)	0.04 (0.00 - 0.09)	0.00 (0.00 - 0.01)	0.09 (0.05 - 0.13)	0.09 (0.05 - 0.13)
**Iran (Islamic Republic of)**	0.23 (0.19 - 0.27)	0.04 (0.03 - 0.05)	0.06 (0.03 - 0.08)	0.01 (0.01 - 0.02)	0.13 (0.08 - 0.19)	0.14 (0.08 - 0.21)
**Iraq**	0.35 (0.30 - 0.42)	0.05 (0.04 - 0.07)	0.06 (0.03 - 0.11)	0.01 (0.00 - 0.02)	0.16 (0.10 - 0.22)	0.15 (0.10 - 0.22)
**Ireland**	0.22 (0.18 - 0.27)	0.17 (0.13 - 0.20)	0.46 (0.33 - 0.55)	0.29 (0.18 - 0.38)	0.13 (0.06 - 0.20)	0.10 (0.05 - 0.17)
**Israel**	0.21 (0.17 - 0.25)	0.10 (0.07 - 0.12)	0.19 (0.11 - 0.29)	0.06 (0.03 - 0.11)	0.12 (0.06 - 0.19)	0.12 (0.06 - 0.20)
**Italy**	0.21 (0.18 - 0.26)	0.10 (0.07 - 0.12)	0.45 (0.32 - 0.55)	0.23 (0.14 - 0.32)	0.15 (0.07 - 0.23)	0.13 (0.06 - 0.21)
**Jamaica**	0.21 (0.17 - 0.25)	0.06 (0.04 - 0.07)	0.31 (0.21 - 0.40)	0.10 (0.06 - 0.15)	0.16 (0.10 - 0.23)	0.15 (0.09 - 0.21)
**Japan**	0.21 (0.17 - 0.25)	0.04 (0.03 - 0.06)	0.33 (0.22 - 0.44)	0.18 (0.09 - 0.27)	0.10 (0.04 - 0.17)	0.07 (0.03 - 0.13)
**Jordan**	0.38 (0.33 - 0.44)	0.09 (0.06 - 0.11)	0.06 (0.03 - 0.10)	0.01 (0.00 - 0.02)	0.17 (0.10 - 0.25)	0.14 (0.08 - 0.21)
**Kazakhstan**	0.39 (0.33 - 0.44)	0.07 (0.04 - 0.09)	0.42 (0.30 - 0.52)	0.20 (0.11 - 0.28)	0.10 (0.07 - 0.14)	0.11 (0.07 - 0.15)
**Kenya**	0.14 (0.10 - 0.17)	0.02 (0.01 - 0.02)	0.32 (0.21 - 0.42)	0.09 (0.05 - 0.13)	0.07 (0.04 - 0.09)	0.05 (0.03 - 0.07)

Kiribati	0.39 (0.33 - 0.45)	0.24 (0.18 - 0.29)	0.14 (0.03 - 0.28)	0.00 (0.00 - 0.01)	0.23 (0.16 - 0.31)	0.18 (0.12 - 0.25)
Kuwait	0.28 (0.23 - 0.34)	0.04 (0.03 - 0.05)	0.01 (0.00 - 0.02)	0.00 (0.00 - 0.00)	0.21 (0.12 - 0.30)	0.17 (0.10 - 0.24)
Kyrgyzstan	0.42 (0.36 - 0.48)	0.06 (0.04 - 0.07)	0.38 (0.27 - 0.48)	0.11 (0.06 - 0.17)	0.07 (0.04 - 0.09)	0.06 (0.04 - 0.08)
Lao People's Democratic Republic	0.33 (0.27 - 0.38)	0.05 (0.03 - 0.07)	0.35 (0.23 - 0.46)	0.16 (0.09 - 0.24)	0.12 (0.07 - 0.17)	0.12 (0.07 - 0.17)
Latvia	0.38 (0.32 - 0.43)	0.13 (0.09 - 0.16)	0.54 (0.41 - 0.63)	0.24 (0.15 - 0.33)	0.10 (0.06 - 0.15)	0.09 (0.05 - 0.13)
Lebanon	0.38 (0.32 - 0.45)	0.23 (0.18 - 0.29)	0.12 (0.07 - 0.17)	0.02 (0.01 - 0.03)	0.16 (0.10 - 0.23)	0.15 (0.08 - 0.23)
Lesotho	0.30 (0.25 - 0.35)	0.03 (0.02 - 0.04)	0.35 (0.23 - 0.45)	0.12 (0.06 - 0.19)	0.08 (0.05 - 0.11)	0.09 (0.06 - 0.13)
Liberia	0.11 (0.08 - 0.14)	0.02 (0.01 - 0.02)	0.30 (0.18 - 0.39)	0.15 (0.08 - 0.22)	0.09 (0.06 - 0.13)	0.09 (0.05 - 0.14)
Libya	0.32 (0.27 - 0.37)	0.01 (0.01 - 0.01)	0.04 (0.01 - 0.05)	0.01 (0.00 - 0.01)	0.18 (0.11 - 0.25)	0.18 (0.11 - 0.25)
Lithuania	0.35 (0.30 - 0.41)	0.12 (0.09 - 0.15)	0.55 (0.41 - 0.64)	0.32 (0.21 - 0.42)	0.09 (0.05 - 0.12)	0.06 (0.04 - 0.09)
Luxembourg	0.24 (0.19 - 0.30)	0.13 (0.09 - 0.16)	0.52 (0.39 - 0.61)	0.31 (0.20 - 0.42)	0.17 (0.09 - 0.27)	0.15 (0.07 - 0.24)
Madagascar	0.12 (0.08 - 0.16)	0.01 (0.01 - 0.02)	0.18 (0.09 - 0.28)	0.06 (0.02 - 0.10)	0.06 (0.04 - 0.08)	0.05 (0.03 - 0.08)
Malawi	0.17 (0.12 - 0.22)	0.02 (0.01 - 0.03)	0.24 (0.15 - 0.33)	0.09 (0.05 - 0.15)	0.08 (0.05 - 0.11)	0.06 (0.04 - 0.10)
Malaysia	0.28 (0.23 - 0.32)	0.03 (0.02 - 0.04)	0.13 (0.07 - 0.21)	0.02 (0.01 - 0.04)	0.14 (0.08 - 0.21)	0.14 (0.08 - 0.21)
Maldives	0.32 (0.26 - 0.37)	0.04 (0.02 - 0.06)	0.10 (0.02 - 0.20)	0.01 (0.00 - 0.02)	0.12 (0.06 - 0.19)	0.11 (0.06 - 0.18)
Mali	0.13 (0.09 - 0.17)	0.01 (0.01 - 0.02)	0.07 (0.04 - 0.10)	0.06 (0.03 - 0.10)	0.06 (0.03 - 0.09)	0.06 (0.03 - 0.10)

**Malta**	0.22 (0.18 - 0.28)	0.09 (0.07 - 0.12)	0.42 (0.29 - 0.52)	0.20 (0.11 - 0.29)	0.15 (0.07 - 0.23)	0.15 (0.07 - 0.25)
**Marshall Islands**	0.24 (0.19 - 0.29)	0.07 (0.05 - 0.09)	0.23 (0.12 - 0.36)	0.02 (0.00 - 0.05)	0.28 (0.20 - 0.37)	0.27 (0.19 - 0.34)
**Mauritania**	0.13 (0.10 - 0.17)	0.03 (0.02 - 0.04)	0.00 (0.00 - 0.00)	0.00 (0.00 - 0.00)	0.05 (0.03 - 0.09)	0.07 (0.04 - 0.11)
**Mauritius**	0.30 (0.25 - 0.35)	0.03 (0.02 - 0.04)	0.35 (0.23 - 0.46)	0.05 (0.01 - 0.10)	0.22 (0.14 - 0.30)	0.20 (0.13 - 0.28)
**Mexico**	0.14 (0.10 - 0.18)	0.04 (0.02 - 0.05)	0.37 (0.26 - 0.46)	0.14 (0.08 - 0.19)	0.18 (0.12 - 0.24)	0.17 (0.11 - 0.23)
**Micronesia (Federated States of)**	0.32 (0.25 - 0.38)	0.17 (0.12 - 0.22)	0.23 (0.14 - 0.32)	0.01 (0.00 - 0.02)	0.21 (0.14 - 0.28)	0.20 (0.13 - 0.27)
**Monaco**	0.23 (0.18 - 0.29)	0.10 (0.07 - 0.14)	0.33 (0.00 - 0.57)	0.17 (0.00 - 0.37)	0.11 (0.06 - 0.18)	0.10 (0.05 - 0.17)
**Mongolia**	0.36 (0.30 - 0.41)	0.06 (0.04 - 0.07)	0.45 (0.32 - 0.55)	0.13 (0.07 - 0.20)	0.05 (0.03 - 0.07)	0.04 (0.03 - 0.06)
**Montenegro**	0.43 (0.37 - 0.51)	0.24 (0.19 - 0.29)	0.49 (0.36 - 0.59)	0.19 (0.10 - 0.28)	0.16 (0.10 - 0.22)	0.14 (0.08 - 0.21)
**Morocco**	0.20 (0.16 - 0.24)	0.01 (0.01 - 0.02)	0.05 (0.03 - 0.08)	0.00 (0.00 - 0.00)	0.15 (0.09 - 0.22)	0.14 (0.08 - 0.20)
**Mozambique**	0.14 (0.10 - 0.19)	0.03 (0.02 - 0.04)	0.17 (0.08 - 0.27)	0.06 (0.02 - 0.11)	0.08 (0.05 - 0.10)	0.06 (0.03 - 0.08)
**Myanmar**	0.26 (0.19 - 0.31)	0.07 (0.04 - 0.10)	0.28 (0.17 - 0.37)	0.03 (0.01 - 0.05)	0.11 (0.07 - 0.17)	0.13 (0.07 - 0.18)
**Namibia**	0.13 (0.09 - 0.17)	0.07 (0.05 - 0.10)	0.40 (0.28 - 0.50)	0.20 (0.11 - 0.28)	0.08 (0.05 - 0.12)	0.08 (0.05 - 0.13)
**Nauru**	0.25 (0.19 - 0.30)	0.19 (0.14 - 0.24)	0.37 (0.25 - 0.47)	0.05 (0.02 - 0.10)	0.18 (0.13 - 0.24)	0.15 (0.10 - 0.20)
**Nepal**	0.24 (0.18 - 0.29)	0.13 (0.09 - 0.16)	0.23 (0.10 - 0.35)	0.06 (0.02 - 0.10)	0.13 (0.08 - 0.18)	0.10 (0.06 - 0.15)
**Netherlands**	0.22 (0.18 - 0.26)	0.14 (0.11 - 0.18)	0.47 (0.35 - 0.56)	0.32 (0.21 - 0.42)	0.10 (0.05 - 0.17)	0.09 (0.04 - 0.15)

**New Zealand**	0.17 (0.13 - 0.20)	0.15 (0.12 - 0.18)	0.45 (0.32 - 0.54)	0.31 (0.20 - 0.40)	0.11 (0.05 - 0.17)	0.10 (0.05 - 0.16)
**Nicaragua**	0.17 (0.13 - 0.21)	0.03 (0.02 - 0.04)	0.29 (0.20 - 0.37)	0.05 (0.03 - 0.08)	0.15 (0.09 - 0.20)	0.14 (0.08 - 0.20)
**Niger**	0.08 (0.06 - 0.10)	0.01 (0.00 - 0.01)	0.04 (0.01 - 0.07)	0.01 (0.00 - 0.03)	0.04 (0.02 - 0.06)	0.04 (0.02 - 0.07)
**Nigeria**	0.07 (0.05 - 0.08)	0.01 (0.01 - 0.02)	0.27 (0.18 - 0.35)	0.08 (0.04 - 0.14)	0.05 (0.03 - 0.08)	0.05 (0.03 - 0.08)
**Niue**	0.23 (0.18 - 0.29)	0.09 (0.06 - 0.12)	0.29 (0.10 - 0.44)	0.03 (0.00 - 0.07)	0.27 (0.17 - 0.36)	0.24 (0.15 - 0.34)
**North Macedonia**	0.39 (0.34 - 0.45)	0.20 (0.16 - 0.25)	0.47 (0.35 - 0.56)	0.09 (0.04 - 0.15)	0.18 (0.10 - 0.26)	0.15 (0.09 - 0.23)
**Northern Mariana Islands**	0.28 (0.22 - 0.34)	0.11 (0.07 - 0.16)	0.16 (0.00 - 0.34)	0.01 (0.00 - 0.04)	0.20 (0.12 - 0.29)	0.19 (0.12 - 0.26)
**Norway**	0.11 (0.09 - 0.14)	0.06 (0.05 - 0.08)	0.38 (0.26 - 0.49)	0.21 (0.11 - 0.31)	0.13 (0.06 - 0.22)	0.12 (0.05 - 0.21)
**Oman**	0.17 (0.14 - 0.21)	0.02 (0.01 - 0.03)	0.04 (0.02 - 0.07)	0.01 (0.00 - 0.01)	0.15 (0.09 - 0.21)	0.14 (0.08 - 0.20)
**Pakistan**	0.22 (0.18 - 0.26)	0.03 (0.02 - 0.04)	0.08 (0.05 - 0.12)	0.01 (0.00 - 0.01)	0.11 (0.07 - 0.15)	0.08 (0.06 - 0.12)
**Palau**	0.25 (0.20 - 0.31)	0.07 (0.05 - 0.10)	0.23 (0.08 - 0.38)	0.02 (0.00 - 0.04)	0.24 (0.16 - 0.33)	0.23 (0.14 - 0.32)
**Palestine**	0.32 (0.27 - 0.39)	0.04 (0.02 - 0.05)	0.11 (0.07 - 0.15)	0.03 (0.02 - 0.04)	0.16 (0.10 - 0.23)	0.16 (0.09 - 0.23)
**Panama**	0.13 (0.10 - 0.17)	0.04 (0.03 - 0.05)	0.36 (0.25 - 0.44)	0.13 (0.08 - 0.19)	0.15 (0.09 - 0.21)	0.12 (0.07 - 0.16)
**Papua New Guinea**	0.20 (0.14 - 0.25)	0.09 (0.06 - 0.12)	0.15 (0.07 - 0.24)	0.01 (0.00 - 0.02)	0.17 (0.11 - 0.23)	0.12 (0.08 - 0.17)
**Paraguay**	0.27 (0.21 - 0.32)	0.10 (0.07 - 0.13)	0.46 (0.33 - 0.55)	0.14 (0.08 - 0.21)	0.11 (0.07 - 0.16)	0.10 (0.06 - 0.14)
**Peru**	0.07 (0.05 - 0.09)	0.02 (0.01 - 0.03)	0.35 (0.24 - 0.46)	0.13 (0.07 - 0.21)	0.07 (0.04 - 0.11)	0.07 (0.04 - 0.11)

**Philippines**	0.32 (0.27 - 0.37)	0.07 (0.05 - 0.09)	0.39 (0.28 - 0.48)	0.14 (0.08 - 0.20)	0.09 (0.05 - 0.13)	0.09 (0.06 - 0.14)
**Poland**	0.33 (0.28 - 0.38)	0.16 (0.12 - 0.19)	0.52 (0.39 - 0.62)	0.11 (0.06 - 0.16)	0.15 (0.10 - 0.21)	0.12 (0.06 - 0.18)
**Portugal**	0.21 (0.17 - 0.25)	0.05 (0.03 - 0.06)	0.51 (0.38 - 0.61)	0.21 (0.13 - 0.29)	0.16 (0.09 - 0.24)	0.15 (0.07 - 0.25)
**Puerto Rico**	0.17 (0.13 - 0.21)	0.07 (0.05 - 0.09)	0.31 (0.21 - 0.39)	0.09 (0.05 - 0.14)	0.20 (0.12 - 0.30)	0.18 (0.10 - 0.27)
**Qatar**	0.24 (0.19 - 0.29)	0.02 (0.01 - 0.02)	0.09 (0.05 - 0.13)	0.01 (0.00 - 0.02)	0.24 (0.16 - 0.31)	0.18 (0.12 - 0.24)
**Republic of Korea**	0.31 (0.26 - 0.36)	0.06 (0.04 - 0.09)	0.45 (0.32 - 0.55)	0.26 (0.16 - 0.38)	0.12 (0.06 - 0.20)	0.10 (0.04 - 0.17)
**Republic of Moldova**	0.46 (0.40 - 0.55)	0.07 (0.05 - 0.09)	0.53 (0.40 - 0.63)	0.31 (0.21 - 0.42)	0.10 (0.06 - 0.14)	0.09 (0.06 - 0.12)
**Romania**	0.36 (0.30 - 0.41)	0.14 (0.11 - 0.17)	0.53 (0.41 - 0.63)	0.23 (0.14 - 0.32)	0.11 (0.07 - 0.15)	0.08 (0.05 - 0.11)
**Russian Federation**	0.46 (0.41 - 0.52)	0.15 (0.12 - 0.18)	0.52 (0.39 - 0.61)	0.28 (0.18 - 0.37)	0.07 (0.04 - 0.09)	0.06 (0.04 - 0.08)
**Rwanda**	0.18 (0.13 - 0.23)	0.10 (0.07 - 0.14)	0.43 (0.30 - 0.54)	0.21 (0.12 - 0.31)	0.06 (0.04 - 0.09)	0.06 (0.03 - 0.08)
**Saint Kitts and Nevis**	0.12 (0.09 - 0.15)	0.03 (0.02 - 0.04)	0.23 (0.01 - 0.44)	0.07 (0.00 - 0.18)	0.18 (0.11 - 0.25)	0.16 (0.09 - 0.23)
**Saint Lucia**	0.17 (0.14 - 0.21)	0.04 (0.03 - 0.05)	0.42 (0.30 - 0.51)	0.19 (0.11 - 0.27)	0.19 (0.12 - 0.26)	0.20 (0.13 - 0.28)
**Saint Vincent and the Grenadines**	0.16 (0.12 - 0.21)	0.03 (0.02 - 0.05)	0.40 (0.29 - 0.49)	0.17 (0.11 - 0.23)	0.19 (0.12 - 0.26)	0.17 (0.11 - 0.24)
**Samoa**	0.36 (0.31 - 0.43)	0.13 (0.10 - 0.17)	0.25 (0.14 - 0.36)	0.02 (0.00 - 0.05)	0.19 (0.12 - 0.27)	0.19 (0.12 - 0.27)
**San Marino**	0.21 (0.15 - 0.27)	0.09 (0.06 - 0.13)	0.44 (0.04 - 0.60)	0.26 (0.01 - 0.41)	0.12 (0.06 - 0.20)	0.11 (0.05 - 0.19)
**Sao Tome and Principe**	0.10 (0.07 - 0.12)	0.01 (0.01 - 0.02)	0.35 (0.23 - 0.44)	0.20 (0.12 - 0.28)	0.09 (0.05 - 0.13)	0.09 (0.05 - 0.14)

**Saudi Arabia**	0.25 (0.20 - 0.31)	0.02 (0.02 - 0.03)	0.03 (0.01 - 0.07)	0.01 (0.00 - 0.02)	0.18 (0.12 - 0.24)	0.15 (0.10 - 0.20)
**Senegal**	0.15 (0.11 - 0.18)	0.01 (0.01 - 0.01)	0.06 (0.03 - 0.09)	0.02 (0.01 - 0.03)	0.10 (0.06 - 0.14)	0.11 (0.06 - 0.17)
**Serbia**	0.35 (0.30 - 0.41)	0.18 (0.15 - 0.22)	0.47 (0.33 - 0.57)	0.11 (0.06 - 0.17)	0.17 (0.10 - 0.25)	0.14 (0.07 - 0.22)
**Seychelles**	0.32 (0.26 - 0.38)	0.04 (0.03 - 0.06)	0.40 (0.27 - 0.52)	0.07 (0.03 - 0.13)	0.21 (0.12 - 0.29)	0.20 (0.11 - 0.30)
**Sierra Leone**	0.16 (0.11 - 0.20)	0.03 (0.02 - 0.04)	0.26 (0.16 - 0.33)	0.12 (0.07 - 0.19)	0.04 (0.02 - 0.06)	0.05 (0.03 - 0.08)
**Singapore**	0.17 (0.13 - 0.20)	0.04 (0.03 - 0.06)	0.14 (0.08 - 0.22)	0.06 (0.03 - 0.10)	0.14 (0.07 - 0.22)	0.10 (0.05 - 0.17)
**Slovakia**	0.30 (0.25 - 0.35)	0.08 (0.06 - 0.11)	0.54 (0.41 - 0.63)	0.16 (0.08 - 0.25)	0.12 (0.07 - 0.18)	0.10 (0.05 - 0.16)
**Slovenia**	0.28 (0.23 - 0.34)	0.11 (0.09 - 0.14)	0.41 (0.22 - 0.57)	0.13 (0.03 - 0.26)	0.12 (0.07 - 0.18)	0.10 (0.05 - 0.17)
**Solomon Islands**	0.31 (0.26 - 0.36)	0.12 (0.09 - 0.15)	0.15 (0.07 - 0.24)	0.01 (0.00 - 0.03)	0.19 (0.13 - 0.25)	0.15 (0.10 - 0.20)
**Somalia**	0.13 (0.09 - 0.18)	0.02 (0.01 - 0.03)	0.00 (0.00 - 0.00)	0.00 (0.00 - 0.00)	0.06 (0.04 - 0.08)	0.05 (0.03 - 0.08)
**South Africa**	0.21 (0.17 - 0.26)	0.05 (0.04 - 0.07)	0.41 (0.29 - 0.49)	0.16 (0.10 - 0.22)	0.10 (0.06 - 0.14)	0.11 (0.06 - 0.15)
**South Sudan**	0.13 (0.09 - 0.17)	0.02 (0.01 - 0.02)	0.04 (0.01 - 0.08)	0.01 (0.00 - 0.02)	0.06 (0.04 - 0.09)	0.05 (0.03 - 0.07)
**Spain**	0.25 (0.21 - 0.30)	0.07 (0.05 - 0.08)	0.44 (0.31 - 0.53)	0.19 (0.11 - 0.27)	0.15 (0.08 - 0.23)	0.13 (0.06 - 0.22)
**Sri Lanka**	0.20 (0.14 - 0.25)	0.02 (0.01 - 0.02)	0.37 (0.25 - 0.46)	0.06 (0.03 - 0.10)	0.20 (0.12 - 0.29)	0.20 (0.11 - 0.28)
**Sudan**	0.23 (0.18 - 0.30)	0.02 (0.02 - 0.03)	0.00 (0.00 - 0.01)	0.00 (0.00 - 0.00)	0.12 (0.08 - 0.18)	0.12 (0.07 - 0.17)
**Suriname**	0.27 (0.22 - 0.32)	0.07 (0.05 - 0.09)	0.36 (0.25 - 0.45)	0.14 (0.08 - 0.20)	0.19 (0.13 - 0.25)	0.16 (0.11 - 0.23)

**Sweden**	0.19 (0.14 - 0.25)	0.14 (0.10 - 0.18)	0.43 (0.31 - 0.53)	0.36 (0.24 - 0.46)	0.12 (0.05 - 0.21)	0.11 (0.04 - 0.19)
**Switzerland**	0.23 (0.19 - 0.28)	0.13 (0.10 - 0.17)	0.49 (0.36 - 0.59)	0.38 (0.26 - 0.49)	0.12 (0.06 - 0.20)	0.11 (0.05 - 0.19)
**Syrian Arab Republic**	0.32 (0.27 - 0.38)	0.05 (0.03 - 0.07)	0.06 (0.03 - 0.09)	0.01 (0.00 - 0.01)	0.14 (0.09 - 0.20)	0.13 (0.08 - 0.18)
**Taiwan (Province of China)**	0.25 (0.20 - 0.30)	0.01 (0.01 - 0.02)	0.31 (0.20 - 0.41)	0.04 (0.02 - 0.07)	0.12 (0.06 - 0.19)	0.11 (0.05 - 0.19)
**Tajikistan**	0.22 (0.18 - 0.28)	0.02 (0.02 - 0.03)	0.25 (0.17 - 0.33)	0.05 (0.02 - 0.08)	0.08 (0.05 - 0.12)	0.09 (0.06 - 0.12)
**Thailand**	0.28 (0.22 - 0.33)	0.03 (0.02 - 0.04)	0.41 (0.29 - 0.50)	0.08 (0.05 - 0.13)	0.12 (0.06 - 0.20)	0.12 (0.06 - 0.19)
**Timor-Leste**	0.30 (0.23 - 0.36)	0.03 (0.02 - 0.04)	0.27 (0.17 - 0.37)	0.03 (0.01 - 0.06)	0.11 (0.06 - 0.17)	0.10 (0.05 - 0.15)
**Togo**	0.18 (0.14 - 0.22)	0.03 (0.02 - 0.04)	0.23 (0.14 - 0.31)	0.10 (0.04 - 0.16)	0.06 (0.03 - 0.08)	0.06 (0.03 - 0.08)
**Tokelau**	0.26 (0.20 - 0.32)	0.10 (0.07 - 0.14)	0.26 (0.14 - 0.38)	0.03 (0.00 - 0.05)	0.19 (0.11 - 0.27)	0.20 (0.12 - 0.27)
**Tonga**	0.33 (0.27 - 0.39)	0.09 (0.06 - 0.11)	0.14 (0.05 - 0.25)	0.01 (0.00 - 0.02)	0.19 (0.11 - 0.27)	0.18 (0.11 - 0.25)
**Trinidad and Tobago**	0.23 (0.18 - 0.28)	0.05 (0.04 - 0.07)	0.38 (0.27 - 0.47)	0.16 (0.09 - 0.23)	0.22 (0.14 - 0.30)	0.20 (0.12 - 0.28)
**Tunisia**	0.37 (0.31 - 0.42)	0.03 (0.02 - 0.04)	0.12 (0.07 - 0.16)	0.03 (0.02 - 0.05)	0.19 (0.11 - 0.28)	0.16 (0.08 - 0.24)
**Turkey**	0.32 (0.27 - 0.38)	0.08 (0.06 - 0.10)	0.12 (0.08 - 0.17)	0.05 (0.03 - 0.08)	0.12 (0.07 - 0.18)	0.11 (0.06 - 0.18)
**Turkmenistan**	0.31 (0.25 - 0.36)	0.04 (0.03 - 0.05)	0.40 (0.28 - 0.49)	0.09 (0.05 - 0.15)	0.08 (0.06 - 0.12)	0.08 (0.05 - 0.11)
**Tuvalu**	0.29 (0.23 - 0.34)	0.12 (0.08 - 0.16)	0.19 (0.10 - 0.30)	0.01 (0.00 - 0.03)	0.20 (0.13 - 0.27)	0.20 (0.12 - 0.28)
**Uganda**	0.10 (0.07 - 0.13)	0.03 (0.02 - 0.04)	0.43 (0.31 - 0.52)	0.23 (0.15 - 0.32)	0.08 (0.05 - 0.11)	0.07 (0.04 - 0.10)

**Ukraine**	0.42 (0.37 - 0.47)	0.12 (0.09 - 0.15)	0.49 (0.36 - 0.59)	0.31 (0.21 - 0.40)	0.08 (0.05 - 0.11)	0.06 (0.04 - 0.09)
**United Arab Emirates**	0.25 (0.20 - 0.31)	0.06 (0.04 - 0.08)	0.14 (0.08 - 0.22)	0.03 (0.01 - 0.07)	0.18 (0.12 - 0.23)	0.17 (0.11 - 0.23)
**United Kingdom**	0.21 (0.17 - 0.25)	0.16 (0.13 - 0.19)	0.44 (0.31 - 0.54)	0.25 (0.16 - 0.35)	0.17 (0.09 - 0.26)	0.15 (0.08 - 0.24)
**United Republic of Tanzania**	0.20 (0.15 - 0.24)	0.06 (0.04 - 0.08)	0.36 (0.25 - 0.45)	0.17 (0.10 - 0.23)	0.06 (0.04 - 0.08)	0.05 (0.03 - 0.08)
**United States Virgin Islands**	0.14 (0.11 - 0.19)	0.07 (0.05 - 0.10)	0.30 (0.01 - 0.50)	0.17 (0.00 - 0.33)	0.17 (0.10 - 0.25)	0.16 (0.09 - 0.24)
**United States of America**	0.24 (0.19 - 0.28)	0.16 (0.13 - 0.19)	0.38 (0.27 - 0.48)	0.26 (0.16 - 0.34)	0.19 (0.11 - 0.27)	0.14 (0.08 - 0.22)
**Uruguay**	0.27 (0.23 - 0.32)	0.14 (0.11 - 0.17)	0.44 (0.32 - 0.54)	0.27 (0.17 - 0.36)	0.10 (0.06 - 0.15)	0.07 (0.04 - 0.12)
**Uzbekistan**	0.28 (0.23 - 0.32)	0.02 (0.02 - 0.03)	0.33 (0.23 - 0.42)	0.06 (0.03 - 0.10)	0.11 (0.07 - 0.15)	0.09 (0.06 - 0.12)
**Vanuatu**	0.21 (0.16 - 0.26)	0.03 (0.02 - 0.04)	0.17 (0.09 - 0.24)	0.01 (0.00 - 0.02)	0.18 (0.12 - 0.25)	0.16 (0.10 - 0.22)
**Venezuela (Bolivarian Republic of)**	0.17 (0.13 - 0.21)	0.07 (0.05 - 0.09)	0.34 (0.24 - 0.43)	0.11 (0.06 - 0.16)	0.15 (0.09 - 0.22)	0.13 (0.08 - 0.19)
**Viet Nam**	0.35 (0.30 - 0.40)	0.02 (0.01 - 0.03)	0.47 (0.34 - 0.57)	0.05 (0.02 - 0.08)	0.11 (0.06 - 0.17)	0.12 (0.06 - 0.19)
**Yemen**	0.31 (0.25 - 0.37)	0.09 (0.07 - 0.11)	0.05 (0.03 - 0.08)	0.01 (0.00 - 0.01)	0.09 (0.06 - 0.13)	0.09 (0.05 - 0.13)
**Zambia**	0.14 (0.09 - 0.19)	0.04 (0.02 - 0.06)	0.34 (0.22 - 0.44)	0.15 (0.08 - 0.21)	0.07 (0.04 - 0.10)	0.06 (0.04 - 0.08)
**Zimbabwe**	0.26 (0.20 - 0.31)	0.03 (0.02 - 0.05)	0.30 (0.19 - 0.40)	0.07 (0.03 - 0.12)	0.09 (0.06 - 0.13)	0.10 (0.06 - 0.14)

eTable 9. All-age population-attributable fractions of tuberculosis due to unsafe sex, drug use, and intimate partner violence, among HIV-positive men and women with 95% uncertainty intervals in 2019 for 204 countries and territories in 2019

	**Unsafe sex**		**Drug use**		**Intimate partner violence**	
	Male	Female	Male	Female	Male	Female
**Afghanistan**	0.34 (0.25 - 0.45)	0.58 (0.42 - 0.73)	0.43 (0.32 - 0.53)	0.07 (0.05 - 0.10)	-	0.11 (0.06 - 0.20)
**Albania**	0.90 (0.89 - 0.91)	0.86 (0.85 - 0.87)	0.02 (0.01 - 0.02)	0.00 (0.00 - 0.00)	-	0.06 (0.03 - 0.10)
**Algeria**	0.39 (0.31 - 0.47)	0.77 (0.72 - 0.82)	0.52 (0.44 - 0.60)	0.10 (0.08 - 0.13)	-	0.14 (0.08 - 0.22)
**American Samoa**	0.76 (0.70 - 0.82)	0.83 (0.79 - 0.86)	0.03 (0.02 - 0.05)	0.02 (0.01 - 0.02)	-	0.13 (0.07 - 0.22)
**Andorra**	0.91 (0.79 - 0.97)	0.90 (0.80 - 0.96)	0.09 (0.03 - 0.22)	0.08 (0.02 - 0.19)	-	0.11 (0.06 - 0.19)
**Angola**	0.83 (0.82 - 0.84)	0.91 (0.90 - 0.92)	0.03 (0.02 - 0.04)	0.02 (0.02 - 0.02)	-	0.21 (0.12 - 0.31)
**Antigua and Barbuda**	0.79 (0.74 - 0.84)	0.77 (0.71 - 0.83)	0.03 (0.03 - 0.04)	0.02 (0.02 - 0.03)	-	0.10 (0.06 - 0.18)
**Argentina**	0.88 (0.84 - 0.90)	0.93 (0.92 - 0.94)	0.11 (0.08 - 0.14)	0.04 (0.03 - 0.05)	-	0.07 (0.04 - 0.14)
**Armenia**	0.50 (0.49 - 0.52)	0.78 (0.77 - 0.80)	0.47 (0.46 - 0.49)	0.14 (0.13 - 0.16)	-	0.08 (0.04 - 0.12)
**Australia**	0.95 (0.94 - 0.95)	0.92 (0.91 - 0.92)	0.04 (0.03 - 0.04)	0.03 (0.03 - 0.04)	-	0.11 (0.06 - 0.18)
**Austria**	0.83 (0.82 - 0.85)	0.81 (0.79 - 0.82)	0.14 (0.13 - 0.16)	0.14 (0.13 - 0.16)	-	0.06 (0.03 - 0.11)
**Azerbaijan**	0.24 (0.22 - 0.26)	0.53 (0.51 - 0.56)	0.71 (0.69 - 0.73)	0.35 (0.33 - 0.37)	-	0.06 (0.03 - 0.09)
**Bahamas**	0.80 (0.74 - 0.85)	0.77 (0.70 - 0.82)	0.04 (0.03 - 0.05)	0.02 (0.02 - 0.03)	-	0.11 (0.06 - 0.19)
**Bahrain**	0.25 (0.22 - 0.27)	0.63 (0.56 - 0.68)	0.72 (0.70 - 0.74)	0.26 (0.21 - 0.31)	-	0.12 (0.07 - 0.19)

**Bangladesh**	0.67 (0.47 - 0.83)	0.64 (0.51 - 0.75)	0.08 (0.06 - 0.12)	0.09 (0.06 - 0.12)	-	0.12 (0.07 - 0.19)
**Barbados**	0.81 (0.76 - 0.86)	0.77 (0.71 - 0.83)	0.03 (0.03 - 0.04)	0.02 (0.02 - 0.03)	-	0.11 (0.06 - 0.18)
**Belarus**	0.57 (0.55 - 0.58)	0.85 (0.85 - 0.86)	0.42 (0.41 - 0.43)	0.13 (0.13 - 0.14)	-	0.13 (0.09 - 0.19)
**Belgium**	0.94 (0.94 - 0.95)	0.91 (0.90 - 0.92)	0.03 (0.03 - 0.04)	0.04 (0.03 - 0.04)	-	0.12 (0.07 - 0.20)
**Belize**	0.76 (0.71 - 0.82)	0.73 (0.66 - 0.80)	0.03 (0.02 - 0.04)	0.02 (0.02 - 0.03)	-	0.08 (0.04 - 0.14)
**Benin**	0.79 (0.78 - 0.81)	0.80 (0.79 - 0.81)	0.04 (0.03 - 0.05)	0.03 (0.02 - 0.03)	-	0.13 (0.07 - 0.22)
**Bermuda**	0.82 (0.77 - 0.86)	0.80 (0.74 - 0.85)	0.03 (0.03 - 0.04)	0.02 (0.02 - 0.03)	-	0.14 (0.08 - 0.22)
**Bhutan**	0.82 (0.74 - 0.89)	0.71 (0.57 - 0.82)	0.02 (0.01 - 0.02)	0.01 (0.01 - 0.01)	-	0.05 (0.03 - 0.09)
**Bolivia (Plurinational State of)**	0.86 (0.75 - 0.92)	0.80 (0.67 - 0.90)	0.00 (0.00 - 0.00)	0.00 (0.00 - 0.00)	-	0.13 (0.07 - 0.20)
**Bosnia and Herzegovina**	0.95 (0.94 - 0.96)	0.95 (0.91 - 0.98)	0.01 (0.01 - 0.02)	0.00 (0.00 - 0.00)	-	0.09 (0.05 - 0.16)
**Botswana**	0.95 (0.94 - 0.96)	0.96 (0.95 - 0.96)	0.03 (0.02 - 0.04)	0.02 (0.02 - 0.03)	-	0.24 (0.15 - 0.34)
**Brazil**	0.71 (0.63 - 0.77)	0.79 (0.74 - 0.84)	0.25 (0.18 - 0.32)	0.15 (0.10 - 0.20)	-	0.07 (0.03 - 0.12)
**Brunei Darussalam**	0.79 (0.75 - 0.84)	0.75 (0.72 - 0.79)	0.00 (0.00 - 0.00)	0.00 (0.00 - 0.00)	-	0.09 (0.04 - 0.14)
**Bulgaria**	0.45 (0.43 - 0.47)	0.76 (0.74 - 0.77)	0.54 (0.52 - 0.56)	0.17 (0.16 - 0.19)	-	0.10 (0.06 - 0.16)
**Burkina Faso**	0.78 (0.75 - 0.82)	0.78 (0.75 - 0.81)	0.03 (0.03 - 0.04)	0.03 (0.02 - 0.03)	-	0.08 (0.04 - 0.15)
**Burundi**	0.79 (0.72 - 0.86)	0.82 (0.77 - 0.86)	0.01 (0.01 - 0.01)	0.01 (0.01 - 0.01)	-	0.13 (0.07 - 0.21)

**Cabo Verde**	0.87 (0.86 - 0.88)	0.85 (0.84 - 0.86)	0.04 (0.03 - 0.05)	0.03 (0.02 - 0.04)	-	0.14 (0.08 - 0.23)
**Cambodia**	0.61 (0.56 - 0.66)	0.84 (0.81 - 0.87)	0.32 (0.28 - 0.37)	0.06 (0.05 - 0.07)	-	0.08 (0.04 - 0.13)
**Cameroon**	0.91 (0.90 - 0.91)	0.93 (0.92 - 0.93)	0.01 (0.01 - 0.02)	0.01 (0.01 - 0.01)	-	0.20 (0.13 - 0.30)
**Canada**	0.81 (0.76 - 0.86)	0.79 (0.73 - 0.84)	0.18 (0.14 - 0.23)	0.18 (0.13 - 0.24)	-	0.08 (0.05 - 0.13)
**Central African Republic**	0.90 (0.88 - 0.92)	0.93 (0.91 - 0.94)	0.03 (0.02 - 0.04)	0.02 (0.02 - 0.03)	-	0.18 (0.10 - 0.29)
**Chad**	0.82 (0.78 - 0.85)	0.82 (0.80 - 0.85)	0.04 (0.03 - 0.05)	0.03 (0.02 - 0.03)	-	0.12 (0.07 - 0.19)
**Chile**	0.98 (0.97 - 0.98)	0.96 (0.96 - 0.96)	0.02 (0.01 - 0.02)	0.02 (0.01 - 0.02)	-	0.20 (0.11 - 0.30)
**China**	0.58 (0.50 - 0.66)	0.87 (0.85 - 0.88)	0.39 (0.30 - 0.47)	0.07 (0.06 - 0.09)	-	0.10 (0.06 - 0.16)
**Colombia**	0.94 (0.92 - 0.95)	0.92 (0.90 - 0.93)	0.02 (0.01 - 0.02)	0.01 (0.01 - 0.02)	-	0.17 (0.10 - 0.25)
**Comoros**	0.92 (0.78 - 0.98)	0.89 (0.72 - 0.97)	0.01 (0.01 - 0.01)	0.01 (0.00 - 0.01)	-	0.06 (0.03 - 0.10)
**Congo**	0.81 (0.80 - 0.82)	0.89 (0.88 - 0.89)	0.03 (0.02 - 0.03)	0.02 (0.02 - 0.03)	-	0.19 (0.12 - 0.29)
**Cook Islands**	0.86 (0.80 - 0.90)	0.88 (0.85 - 0.91)	0.04 (0.02 - 0.06)	0.02 (0.01 - 0.02)	-	0.16 (0.09 - 0.24)
**Costa Rica**	0.92 (0.91 - 0.94)	0.89 (0.87 - 0.90)	0.02 (0.01 - 0.02)	0.01 (0.01 - 0.02)	-	0.14 (0.08 - 0.21)
**Croatia**	0.87 (0.85 - 0.88)	0.91 (0.90 - 0.91)	0.10 (0.09 - 0.12)	0.03 (0.03 - 0.04)	-	0.10 (0.06 - 0.17)
**Cuba**	0.80 (0.75 - 0.84)	0.78 (0.73 - 0.83)	0.03 (0.03 - 0.04)	0.02 (0.02 - 0.03)	-	0.11 (0.07 - 0.18)
**Cyprus**	0.95 (0.94 - 0.96)	0.94 (0.93 - 0.95)	0.04 (0.03 - 0.05)	0.04 (0.03 - 0.05)	-	0.07 (0.04 - 0.11)

**Czechia**	0.86 (0.85 - 0.87)	0.92 (0.92 - 0.92)	0.09 (0.08 - 0.10)	0.03 (0.02 - 0.03)	-	0.13 (0.08 - 0.20)
**CÃ´te d'Ivoire**	0.82 (0.81 - 0.84)	0.85 (0.84 - 0.86)	0.04 (0.03 - 0.05)	0.03 (0.02 - 0.04)	-	0.16 (0.09 - 0.25)
**Democratic People's Republic of Korea**	0.53 (0.43 - 0.64)	0.81 (0.70 - 0.89)	0.40 (0.31 - 0.52)	0.08 (0.06 - 0.10)	-	0.09 (0.05 - 0.15)
**Democratic Republic of the Congo**	0.75 (0.70 - 0.80)	0.86 (0.83 - 0.88)	0.02 (0.02 - 0.03)	0.02 (0.01 - 0.02)	-	0.18 (0.11 - 0.27)
**Denmark**	0.92 (0.91 - 0.93)	0.89 (0.88 - 0.90)	0.06 (0.05 - 0.07)	0.07 (0.06 - 0.08)	-	0.13 (0.08 - 0.20)
**Djibouti**	0.95 (0.95 - 0.96)	0.97 (0.97 - 0.97)	0.00 (0.00 - 0.00)	0.00 (0.00 - 0.00)	-	0.18 (0.11 - 0.28)
**Dominica**	0.78 (0.73 - 0.83)	0.74 (0.68 - 0.79)	0.03 (0.02 - 0.04)	0.02 (0.01 - 0.03)	-	0.09 (0.05 - 0.16)
**Dominican Republic**	0.80 (0.74 - 0.84)	0.77 (0.71 - 0.83)	0.03 (0.03 - 0.04)	0.02 (0.02 - 0.03)	-	0.09 (0.05 - 0.15)
**Ecuador**	0.92 (0.90 - 0.94)	0.89 (0.86 - 0.91)	0.01 (0.00 - 0.01)	0.00 (0.00 - 0.01)	-	0.09 (0.05 - 0.16)
**Egypt**	0.39 (0.33 - 0.45)	0.69 (0.65 - 0.73)	0.48 (0.42 - 0.55)	0.08 (0.07 - 0.10)	-	0.08 (0.05 - 0.14)
**El Salvador**	0.87 (0.85 - 0.88)	0.78 (0.77 - 0.80)	0.01 (0.01 - 0.02)	0.01 (0.01 - 0.02)	-	0.10 (0.06 - 0.17)
**Equatorial Guinea**	0.84 (0.83 - 0.85)	0.89 (0.89 - 0.90)	0.03 (0.02 - 0.03)	0.02 (0.01 - 0.02)	-	0.21 (0.13 - 0.32)
**Eritrea**	0.87 (0.84 - 0.89)	0.92 (0.90 - 0.94)	0.01 (0.01 - 0.01)	0.01 (0.01 - 0.01)	-	0.16 (0.08 - 0.26)
**Estonia**	0.49 (0.46 - 0.53)	0.82 (0.80 - 0.83)	0.50 (0.46 - 0.53)	0.16 (0.15 - 0.18)	-	0.09 (0.06 - 0.13)
**Eswatini**	0.89 (0.86 - 0.91)	0.90 (0.88 - 0.91)	0.03 (0.02 - 0.04)	0.02 (0.02 - 0.02)	-	0.18 (0.11 - 0.26)
**Ethiopia**	0.74 (0.72 - 0.75)	0.83 (0.82 - 0.84)	0.01 (0.01 - 0.01)	0.01 (0.01 - 0.01)	-	0.14 (0.09 - 0.22)

**Fiji**	0.74 (0.69 - 0.79)	0.75 (0.71 - 0.80)	0.00 (0.00 - 0.01)	0.00 (0.00 - 0.00)	-	0.12 (0.07 - 0.20)
**Finland**	0.87 (0.86 - 0.88)	0.84 (0.83 - 0.86)	0.08 (0.07 - 0.10)	0.08 (0.06 - 0.09)	-	0.13 (0.08 - 0.20)
**France**	0.97 (0.96 - 0.97)	0.94 (0.94 - 0.95)	0.02 (0.02 - 0.02)	0.02 (0.02 - 0.02)	-	0.12 (0.06 - 0.19)
**Gabon**	0.87 (0.86 - 0.88)	0.93 (0.92 - 0.93)	0.03 (0.02 - 0.04)	0.02 (0.02 - 0.03)	-	0.24 (0.15 - 0.34)
**Gambia**	0.90 (0.88 - 0.91)	0.91 (0.90 - 0.91)	0.04 (0.03 - 0.05)	0.03 (0.02 - 0.04)	-	0.12 (0.06 - 0.19)
**Georgia**	0.30 (0.30 - 0.31)	0.66 (0.65 - 0.67)	0.66 (0.65 - 0.67)	0.29 (0.28 - 0.30)	-	0.02 (0.01 - 0.03)
**Germany**	0.91 (0.90 - 0.92)	0.88 (0.87 - 0.89)	0.05 (0.05 - 0.06)	0.05 (0.05 - 0.06)	-	0.12 (0.08 - 0.19)
**Ghana**	0.89 (0.88 - 0.90)	0.92 (0.91 - 0.92)	0.04 (0.03 - 0.05)	0.03 (0.03 - 0.04)	-	0.20 (0.12 - 0.30)
**Greece**	0.62 (0.60 - 0.65)	0.60 (0.57 - 0.62)	0.33 (0.30 - 0.36)	0.29 (0.27 - 0.32)	-	0.06 (0.03 - 0.11)
**Greenland**	0.74 (0.71 - 0.78)	0.75 (0.72 - 0.78)	0.11 (0.09 - 0.14)	0.12 (0.10 - 0.15)	-	0.10 (0.06 - 0.16)
**Grenada**	0.80 (0.74 - 0.85)	0.76 (0.70 - 0.82)	0.03 (0.03 - 0.04)	0.02 (0.02 - 0.03)	-	0.10 (0.05 - 0.17)
**Guam**	0.81 (0.76 - 0.86)	0.79 (0.76 - 0.83)	0.04 (0.02 - 0.06)	0.02 (0.01 - 0.02)	-	0.13 (0.07 - 0.20)
**Guatemala**	0.89 (0.87 - 0.90)	0.83 (0.81 - 0.84)	0.01 (0.00 - 0.01)	0.00 (0.00 - 0.01)	-	0.06 (0.03 - 0.10)
**Guinea**	0.85 (0.83 - 0.87)	0.89 (0.87 - 0.90)	0.04 (0.03 - 0.05)	0.03 (0.02 - 0.04)	-	0.15 (0.08 - 0.24)
**Guinea-Bissau**	0.89 (0.86 - 0.91)	0.89 (0.87 - 0.90)	0.04 (0.03 - 0.05)	0.03 (0.02 - 0.04)	-	0.14 (0.08 - 0.24)
**Guyana**	0.77 (0.72 - 0.82)	0.74 (0.67 - 0.80)	0.03 (0.02 - 0.04)	0.02 (0.02 - 0.03)	-	0.11 (0.06 - 0.18)

**Haiti**	0.73 (0.68 - 0.77)	0.72 (0.67 - 0.78)	0.03 (0.02 - 0.04)	0.02 (0.02 - 0.03)	-	0.09 (0.05 - 0.15)
**Honduras**	0.89 (0.82 - 0.94)	0.87 (0.78 - 0.92)	0.02 (0.01 - 0.02)	0.01 (0.01 - 0.01)	-	0.08 (0.04 - 0.13)
**Hungary**	0.96 (0.95 - 0.97)	0.96 (0.96 - 0.97)	0.03 (0.03 - 0.04)	0.01 (0.01 - 0.01)	-	0.08 (0.04 - 0.13)
**Iceland**	0.47 (0.40 - 0.54)	0.49 (0.43 - 0.56)	0.51 (0.43 - 0.58)	0.43 (0.36 - 0.49)	-	0.02 (0.01 - 0.05)
**India**	0.69 (0.64 - 0.74)	0.63 (0.56 - 0.70)	0.10 (0.08 - 0.13)	0.11 (0.08 - 0.14)	-	0.08 (0.05 - 0.12)
**Indonesia**	0.60 (0.51 - 0.69)	0.74 (0.69 - 0.79)	0.22 (0.15 - 0.31)	0.04 (0.03 - 0.05)	-	0.09 (0.05 - 0.16)
**Iran (Islamic Republic of)**	0.23 (0.15 - 0.33)	0.39 (0.30 - 0.49)	0.61 (0.52 - 0.70)	0.37 (0.29 - 0.45)	-	0.09 (0.06 - 0.13)
**Iraq**	0.21 (0.18 - 0.25)	0.64 (0.61 - 0.66)	0.23 (0.20 - 0.27)	0.08 (0.07 - 0.10)	-	0.06 (0.03 - 0.11)
**Ireland**	0.87 (0.86 - 0.89)	0.84 (0.82 - 0.86)	0.10 (0.08 - 0.11)	0.09 (0.08 - 0.11)	-	0.08 (0.04 - 0.13)
**Israel**	0.79 (0.77 - 0.80)	0.72 (0.71 - 0.73)	0.15 (0.14 - 0.16)	0.12 (0.11 - 0.13)	-	0.11 (0.07 - 0.17)
**Italy**	0.90 (0.86 - 0.92)	0.88 (0.84 - 0.91)	0.09 (0.06 - 0.12)	0.09 (0.06 - 0.12)	-	0.15 (0.09 - 0.24)
**Jamaica**	0.79 (0.73 - 0.83)	0.76 (0.70 - 0.81)	0.03 (0.03 - 0.04)	0.02 (0.02 - 0.03)	-	0.10 (0.06 - 0.17)
**Japan**	0.95 (0.94 - 0.95)	0.94 (0.93 - 0.94)	0.00 (0.00 - 0.01)	0.00 (0.00 - 0.00)	-	0.18 (0.11 - 0.28)
**Jordan**	0.59 (0.56 - 0.63)	0.63 (0.61 - 0.64)	0.18 (0.15 - 0.21)	0.01 (0.01 - 0.01)	-	0.07 (0.04 - 0.11)
**Kazakhstan**	0.29 (0.27 - 0.30)	0.64 (0.63 - 0.65)	0.69 (0.67 - 0.71)	0.31 (0.30 - 0.33)	-	0.06 (0.04 - 0.09)
**Kenya**	0.82 (0.80 - 0.85)	0.86 (0.84 - 0.87)	0.09 (0.07 - 0.11)	0.07 (0.05 - 0.08)	-	0.18 (0.11 - 0.28)

**Kiribati**	0.52 (0.46 - 0.57)	0.62 (0.57 - 0.67)	0.02 (0.01 - 0.03)	0.01 (0.01 - 0.01)	-	0.10 (0.06 - 0.16)
**Kuwait**	0.37 (0.30 - 0.45)	0.64 (0.62 - 0.67)	0.50 (0.42 - 0.58)	0.09 (0.07 - 0.11)	-	0.11 (0.06 - 0.17)
**Kyrgyzstan**	0.20 (0.19 - 0.22)	0.50 (0.48 - 0.52)	0.71 (0.70 - 0.72)	0.36 (0.34 - 0.38)	-	0.07 (0.05 - 0.10)
**Lao People's Democratic Republic**	0.84 (0.43 - 0.98)	0.78 (0.41 - 0.97)	0.03 (0.02 - 0.05)	0.01 (0.00 - 0.01)	-	0.07 (0.03 - 0.14)
**Latvia**	0.36 (0.34 - 0.38)	0.73 (0.72 - 0.75)	0.63 (0.61 - 0.65)	0.26 (0.25 - 0.28)	-	0.11 (0.07 - 0.16)
**Lebanon**	0.34 (0.25 - 0.44)	0.67 (0.53 - 0.78)	0.45 (0.34 - 0.55)	0.09 (0.07 - 0.11)	-	0.11 (0.06 - 0.18)
**Lesotho**	0.91 (0.89 - 0.93)	0.92 (0.91 - 0.93)	0.03 (0.02 - 0.04)	0.02 (0.02 - 0.03)	-	0.21 (0.13 - 0.31)
**Liberia**	0.90 (0.89 - 0.92)	0.91 (0.90 - 0.92)	0.04 (0.03 - 0.06)	0.03 (0.02 - 0.04)	-	0.19 (0.12 - 0.29)
**Libya**	0.40 (0.32 - 0.48)	0.80 (0.75 - 0.84)	0.51 (0.43 - 0.60)	0.10 (0.08 - 0.13)	-	0.14 (0.07 - 0.22)
**Lithuania**	0.17 (0.16 - 0.19)	0.47 (0.45 - 0.49)	0.80 (0.79 - 0.81)	0.52 (0.50 - 0.54)	-	0.05 (0.03 - 0.07)
**Luxembourg**	0.79 (0.76 - 0.81)	0.77 (0.74 - 0.80)	0.15 (0.13 - 0.17)	0.14 (0.12 - 0.16)	-	0.08 (0.04 - 0.14)
**Madagascar**	0.86 (0.85 - 0.86)	0.91 (0.91 - 0.91)	0.01 (0.01 - 0.01)	0.01 (0.01 - 0.01)	-	0.17 (0.09 - 0.27)
**Malawi**	0.85 (0.79 - 0.89)	0.88 (0.85 - 0.90)	0.01 (0.01 - 0.01)	0.01 (0.01 - 0.01)	-	0.17 (0.10 - 0.25)
**Malaysia**	0.31 (0.28 - 0.34)	0.53 (0.49 - 0.57)	0.48 (0.44 - 0.52)	0.15 (0.13 - 0.17)	-	0.05 (0.03 - 0.09)
**Maldives**	0.62 (0.56 - 0.69)	0.90 (0.89 - 0.91)	0.21 (0.15 - 0.28)	0.04 (0.03 - 0.05)	-	0.10 (0.05 - 0.17)
**Mali**	0.83 (0.82 - 0.84)	0.86 (0.85 - 0.87)	0.04 (0.03 - 0.05)	0.03 (0.02 - 0.04)	-	0.12 (0.07 - 0.20)

**Malta**	0.84 (0.81 - 0.87)	0.82 (0.80 - 0.85)	0.10 (0.08 - 0.12)	0.09 (0.07 - 0.10)	-	0.07 (0.04 - 0.12)
**Marshall Islands**	0.76 (0.60 - 0.89)	0.81 (0.69 - 0.90)	0.07 (0.04 - 0.10)	0.04 (0.03 - 0.05)	-	0.10 (0.05 - 0.18)
**Mauritania**	0.92 (0.86 - 0.96)	0.92 (0.85 - 0.96)	0.04 (0.03 - 0.05)	0.03 (0.02 - 0.03)	-	0.15 (0.08 - 0.25)
**Mauritius**	0.69 (0.58 - 0.79)	0.94 (0.92 - 0.95)	0.30 (0.20 - 0.41)	0.05 (0.04 - 0.06)	-	0.11 (0.06 - 0.18)
**Mexico**	0.92 (0.91 - 0.94)	0.89 (0.87 - 0.91)	0.02 (0.01 - 0.02)	0.01 (0.01 - 0.02)	-	0.13 (0.07 - 0.19)
**Micronesia (Federated States of)**	0.79 (0.53 - 0.94)	0.82 (0.54 - 0.95)	0.04 (0.02 - 0.07)	0.02 (0.01 - 0.03)	-	0.16 (0.09 - 0.25)
**Monaco**	0.91 (0.89 - 0.94)	0.91 (0.86 - 0.94)	0.08 (0.05 - 0.10)	0.08 (0.05 - 0.11)	-	0.10 (0.05 - 0.17)
**Mongolia**	0.31 (0.24 - 0.40)	0.54 (0.47 - 0.61)	0.66 (0.57 - 0.74)	0.37 (0.31 - 0.44)	-	0.05 (0.03 - 0.08)
**Montenegro**	0.64 (0.60 - 0.67)	0.58 (0.57 - 0.60)	0.17 (0.14 - 0.21)	0.06 (0.05 - 0.07)	-	0.05 (0.03 - 0.09)
**Morocco**	0.38 (0.28 - 0.47)	0.73 (0.61 - 0.83)	0.47 (0.34 - 0.60)	0.09 (0.07 - 0.12)	-	0.12 (0.07 - 0.21)
**Mozambique**	0.78 (0.72 - 0.82)	0.80 (0.77 - 0.83)	0.01 (0.01 - 0.01)	0.01 (0.00 - 0.01)	-	0.13 (0.08 - 0.20)
**Myanmar**	0.51 (0.47 - 0.54)	0.86 (0.85 - 0.88)	0.44 (0.39 - 0.49)	0.10 (0.10 - 0.11)	-	0.13 (0.08 - 0.21)
**Namibia**	0.87 (0.85 - 0.88)	0.89 (0.88 - 0.90)	0.03 (0.02 - 0.04)	0.02 (0.02 - 0.03)	-	0.16 (0.09 - 0.24)
**Nauru**	0.82 (0.75 - 0.88)	0.84 (0.78 - 0.89)	0.04 (0.02 - 0.06)	0.02 (0.01 - 0.02)	-	0.15 (0.08 - 0.24)
**Nepal**	0.73 (0.67 - 0.78)	0.65 (0.57 - 0.72)	0.08 (0.06 - 0.09)	0.08 (0.07 - 0.10)	-	0.10 (0.06 - 0.15)
**Netherlands**	0.96 (0.96 - 0.97)	0.92 (0.91 - 0.92)	0.02 (0.02 - 0.03)	0.03 (0.02 - 0.03)	-	0.12 (0.07 - 0.20)

**New Zealand**	0.93 (0.90 - 0.95)	0.87 (0.85 - 0.89)	0.05 (0.03 - 0.07)	0.03 (0.02 - 0.04)	-	0.17 (0.10 - 0.26)
**Nicaragua**	0.88 (0.87 - 0.90)	0.85 (0.83 - 0.87)	0.01 (0.01 - 0.02)	0.01 (0.01 - 0.02)	-	0.10 (0.06 - 0.16)
**Niger**	0.86 (0.82 - 0.88)	0.86 (0.84 - 0.88)	0.04 (0.03 - 0.05)	0.03 (0.02 - 0.04)	-	0.14 (0.08 - 0.23)
**Nigeria**	0.72 (0.67 - 0.77)	0.78 (0.75 - 0.80)	0.17 (0.13 - 0.23)	0.13 (0.10 - 0.16)	-	0.12 (0.07 - 0.19)
**Niue**	0.86 (0.79 - 0.91)	0.88 (0.84 - 0.91)	0.04 (0.02 - 0.06)	0.02 (0.01 - 0.02)	-	0.16 (0.09 - 0.25)
**North Macedonia**	0.61 (0.58 - 0.65)	0.62 (0.61 - 0.63)	0.10 (0.07 - 0.13)	0.02 (0.02 - 0.03)	-	0.07 (0.03 - 0.11)
**Northern Mariana Islands**	0.80 (0.73 - 0.86)	0.86 (0.83 - 0.89)	0.04 (0.02 - 0.06)	0.02 (0.01 - 0.02)	-	0.14 (0.08 - 0.22)
**Norway**	0.92 (0.89 - 0.93)	0.87 (0.84 - 0.89)	0.06 (0.05 - 0.08)	0.06 (0.04 - 0.09)	-	0.09 (0.04 - 0.16)
**Oman**	0.75 (0.70 - 0.79)	0.89 (0.88 - 0.90)	0.21 (0.17 - 0.25)	0.02 (0.02 - 0.03)	-	0.16 (0.09 - 0.26)
**Pakistan**	0.36 (0.26 - 0.48)	0.29 (0.21 - 0.40)	0.35 (0.24 - 0.49)	0.35 (0.25 - 0.48)	-	0.07 (0.04 - 0.11)
**Palau**	0.86 (0.80 - 0.91)	0.89 (0.86 - 0.92)	0.04 (0.02 - 0.06)	0.02 (0.02 - 0.03)	-	0.16 (0.10 - 0.25)
**Palestine**	0.35 (0.28 - 0.43)	0.65 (0.62 - 0.68)	0.44 (0.36 - 0.51)	0.08 (0.07 - 0.10)	-	0.10 (0.06 - 0.16)
**Panama**	0.93 (0.91 - 0.95)	0.91 (0.89 - 0.93)	0.01 (0.01 - 0.02)	0.01 (0.01 - 0.02)	-	0.12 (0.07 - 0.21)
**Papua New Guinea**	0.80 (0.72 - 0.86)	0.85 (0.81 - 0.89)	0.04 (0.02 - 0.06)	0.02 (0.01 - 0.02)	-	0.18 (0.12 - 0.26)
**Paraguay**	0.57 (0.50 - 0.63)	0.63 (0.59 - 0.67)	0.26 (0.20 - 0.32)	0.13 (0.10 - 0.17)	-	0.05 (0.03 - 0.08)
**Peru**	0.73 (0.72 - 0.75)	0.61 (0.60 - 0.62)	0.00 (0.00 - 0.01)	0.00 (0.00 - 0.01)	-	0.09 (0.06 - 0.13)

**Philippines**	0.78 (0.75 - 0.80)	0.77 (0.76 - 0.77)	0.07 (0.05 - 0.11)	0.01 (0.01 - 0.01)	-	0.07 (0.04 - 0.12)
**Poland**	0.63 (0.52 - 0.72)	0.83 (0.81 - 0.85)	0.34 (0.25 - 0.45)	0.09 (0.07 - 0.11)	-	0.07 (0.03 - 0.13)
**Portugal**	0.86 (0.84 - 0.87)	0.86 (0.84 - 0.87)	0.13 (0.12 - 0.14)	0.12 (0.11 - 0.14)	-	0.06 (0.03 - 0.11)
**Puerto Rico**	0.83 (0.78 - 0.87)	0.80 (0.74 - 0.85)	0.04 (0.03 - 0.04)	0.03 (0.02 - 0.03)	-	0.11 (0.06 - 0.18)
**Qatar**	0.40 (0.32 - 0.48)	0.74 (0.72 - 0.77)	0.53 (0.45 - 0.60)	0.10 (0.08 - 0.13)	-	0.12 (0.06 - 0.20)
**Republic of Korea**	0.94 (0.93 - 0.96)	0.92 (0.91 - 0.94)	0.00 (0.00 - 0.00)	0.00 (0.00 - 0.00)	-	0.08 (0.04 - 0.13)
**Republic of Moldova**	0.73 (0.71 - 0.74)	0.91 (0.90 - 0.91)	0.27 (0.26 - 0.28)	0.09 (0.08 - 0.09)	-	0.13 (0.09 - 0.18)
**Romania**	0.47 (0.45 - 0.49)	0.74 (0.72 - 0.75)	0.50 (0.47 - 0.52)	0.16 (0.15 - 0.18)	-	0.09 (0.05 - 0.14)
**Russian Federation**	0.36 (0.28 - 0.45)	0.64 (0.59 - 0.70)	0.61 (0.53 - 0.69)	0.31 (0.26 - 0.37)	-	0.12 (0.08 - 0.17)
**Rwanda**	0.91 (0.89 - 0.93)	0.94 (0.94 - 0.95)	0.01 (0.01 - 0.01)	0.01 (0.01 - 0.01)	-	0.19 (0.11 - 0.29)
**Saint Kitts and Nevis**	0.80 (0.75 - 0.85)	0.78 (0.71 - 0.83)	0.03 (0.03 - 0.04)	0.02 (0.02 - 0.03)	-	0.11 (0.06 - 0.18)
**Saint Lucia**	0.79 (0.74 - 0.84)	0.77 (0.71 - 0.83)	0.03 (0.02 - 0.04)	0.02 (0.02 - 0.03)	-	0.10 (0.05 - 0.17)
**Saint Vincent and the Grenadines**	0.79 (0.74 - 0.84)	0.76 (0.69 - 0.81)	0.03 (0.03 - 0.04)	0.02 (0.02 - 0.03)	-	0.10 (0.05 - 0.17)
**Samoa**	0.77 (0.57 - 0.90)	0.82 (0.69 - 0.92)	0.03 (0.01 - 0.05)	0.01 (0.01 - 0.02)	-	0.09 (0.04 - 0.16)
**San Marino**	0.91 (0.88 - 0.93)	0.89 (0.86 - 0.92)	0.08 (0.06 - 0.10)	0.09 (0.07 - 0.12)	-	0.10 (0.06 - 0.17)
**Sao Tome and Principe**	0.89 (0.79 - 0.95)	0.84 (0.64 - 0.95)	0.04 (0.03 - 0.05)	0.03 (0.02 - 0.04)	-	0.13 (0.07 - 0.21)

**Saudi Arabia**	0.40 (0.32 - 0.48)	0.81 (0.77 - 0.85)	0.53 (0.45 - 0.61)	0.11 (0.09 - 0.14)	-	0.15 (0.08 - 0.22)
**Senegal**	0.85 (0.84 - 0.87)	0.90 (0.89 - 0.91)	0.04 (0.03 - 0.05)	0.03 (0.02 - 0.04)	-	0.15 (0.08 - 0.23)
**Serbia**	0.72 (0.69 - 0.75)	0.73 (0.70 - 0.76)	0.22 (0.19 - 0.25)	0.08 (0.07 - 0.10)	-	0.06 (0.03 - 0.10)
**Seychelles**	0.41 (0.33 - 0.50)	0.83 (0.78 - 0.86)	0.57 (0.49 - 0.64)	0.15 (0.11 - 0.20)	-	0.10 (0.05 - 0.17)
**Sierra Leone**	0.91 (0.90 - 0.92)	0.90 (0.89 - 0.91)	0.02 (0.02 - 0.03)	0.02 (0.01 - 0.02)	-	0.19 (0.12 - 0.29)
**Singapore**	0.92 (0.90 - 0.94)	0.89 (0.87 - 0.92)	0.02 (0.01 - 0.02)	0.02 (0.01 - 0.02)	-	0.12 (0.07 - 0.19)
**Slovakia**	0.85 (0.83 - 0.86)	0.79 (0.78 - 0.79)	0.07 (0.05 - 0.08)	0.01 (0.01 - 0.02)	-	0.09 (0.05 - 0.14)
**Slovenia**	0.83 (0.81 - 0.86)	0.81 (0.80 - 0.82)	0.08 (0.06 - 0.10)	0.02 (0.02 - 0.03)	-	0.09 (0.05 - 0.15)
**Solomon Islands**	0.71 (0.47 - 0.89)	0.78 (0.62 - 0.91)	0.03 (0.01 - 0.05)	0.01 (0.01 - 0.02)	-	0.15 (0.08 - 0.24)
**Somalia**	0.78 (0.74 - 0.83)	0.85 (0.83 - 0.88)	0.01 (0.01 - 0.01)	0.01 (0.01 - 0.01)	-	0.15 (0.09 - 0.23)
**South Africa**	0.91 (0.89 - 0.92)	0.93 (0.92 - 0.94)	0.04 (0.03 - 0.05)	0.02 (0.02 - 0.03)	-	0.20 (0.12 - 0.31)
**South Sudan**	0.90 (0.88 - 0.91)	0.93 (0.92 - 0.94)	0.01 (0.01 - 0.02)	0.01 (0.01 - 0.01)	-	0.17 (0.10 - 0.26)
**Spain**	0.88 (0.86 - 0.89)	0.86 (0.84 - 0.87)	0.10 (0.09 - 0.11)	0.10 (0.09 - 0.11)	-	0.04 (0.02 - 0.08)
**Sri Lanka**	0.86 (0.82 - 0.89)	0.82 (0.77 - 0.87)	0.02 (0.02 - 0.02)	0.00 (0.00 - 0.00)	-	0.10 (0.06 - 0.16)
**Sudan**	0.39 (0.31 - 0.48)	0.81 (0.78 - 0.84)	0.53 (0.45 - 0.61)	0.11 (0.09 - 0.14)	-	0.17 (0.10 - 0.26)
**Suriname**	0.78 (0.73 - 0.83)	0.74 (0.68 - 0.80)	0.03 (0.03 - 0.04)	0.02 (0.02 - 0.03)	-	0.10 (0.05 - 0.17)

**Sweden**	0.84 (0.80 - 0.88)	0.82 (0.77 - 0.86)	0.13 (0.10 - 0.17)	0.13 (0.09 - 0.17)	-	0.10 (0.05 - 0.17)
**Switzerland**	0.92 (0.91 - 0.93)	0.89 (0.88 - 0.90)	0.06 (0.05 - 0.07)	0.07 (0.06 - 0.07)	-	0.06 (0.03 - 0.10)
**Syrian Arab Republic**	0.70 (0.66 - 0.74)	0.78 (0.75 - 0.80)	0.05 (0.03 - 0.06)	0.00 (0.00 - 0.00)	-	0.12 (0.07 - 0.19)
**Taiwan (Province of China)**	0.55 (0.46 - 0.65)	0.86 (0.85 - 0.88)	0.43 (0.34 - 0.52)	0.09 (0.07 - 0.11)	-	0.09 (0.05 - 0.16)
**Tajikistan**	0.22 (0.20 - 0.24)	0.56 (0.53 - 0.59)	0.72 (0.70 - 0.74)	0.40 (0.37 - 0.42)	-	0.07 (0.04 - 0.10)
**Thailand**	0.73 (0.68 - 0.77)	0.94 (0.93 - 0.95)	0.26 (0.21 - 0.31)	0.05 (0.04 - 0.06)	-	0.05 (0.03 - 0.09)
**Timor-Leste**	0.59 (0.46 - 0.73)	0.71 (0.50 - 0.87)	0.21 (0.14 - 0.31)	0.03 (0.02 - 0.05)	-	0.12 (0.06 - 0.19)
**Togo**	0.87 (0.86 - 0.88)	0.89 (0.88 - 0.90)	0.04 (0.03 - 0.05)	0.03 (0.02 - 0.04)	-	0.12 (0.06 - 0.21)
**Tokelau**	0.84 (0.77 - 0.89)	0.85 (0.80 - 0.90)	0.04 (0.02 - 0.06)	0.02 (0.01 - 0.02)	-	0.15 (0.09 - 0.24)
**Tonga**	0.80 (0.73 - 0.86)	0.80 (0.74 - 0.85)	0.03 (0.02 - 0.06)	0.02 (0.01 - 0.02)	-	0.11 (0.06 - 0.18)
**Trinidad and Tobago**	0.79 (0.73 - 0.84)	0.75 (0.68 - 0.81)	0.03 (0.03 - 0.04)	0.02 (0.02 - 0.03)	-	0.10 (0.05 - 0.17)
**Tunisia**	0.53 (0.44 - 0.61)	0.84 (0.79 - 0.89)	0.31 (0.24 - 0.39)	0.02 (0.02 - 0.03)	-	0.15 (0.08 - 0.23)
**Turkey**	0.83 (0.81 - 0.85)	0.81 (0.80 - 0.82)	0.04 (0.03 - 0.05)	0.00 (0.00 - 0.00)	-	0.15 (0.09 - 0.22)
**Turkmenistan**	0.31 (0.23 - 0.41)	0.56 (0.47 - 0.63)	0.67 (0.56 - 0.75)	0.40 (0.33 - 0.48)	-	0.05 (0.03 - 0.09)
**Tuvalu**	0.85 (0.79 - 0.90)	0.86 (0.80 - 0.90)	0.04 (0.02 - 0.06)	0.02 (0.01 - 0.02)	-	0.15 (0.09 - 0.24)
**Uganda**	0.85 (0.79 - 0.89)	0.85 (0.82 - 0.88)	0.00 (0.00 - 0.01)	0.00 (0.00 - 0.00)	-	0.19 (0.12 - 0.29)

**Ukraine**	0.36 (0.28 - 0.45)	0.72 (0.68 - 0.75)	0.64 (0.55 - 0.72)	0.27 (0.24 - 0.31)	-	0.07 (0.04 - 0.11)
**United Arab Emirates**	0.40 (0.32 - 0.49)	0.78 (0.72 - 0.82)	0.56 (0.47 - 0.64)	0.11 (0.08 - 0.13)	-	0.13 (0.07 - 0.22)
**United Kingdom**	0.91 (0.89 - 0.93)	0.88 (0.85 - 0.90)	0.05 (0.04 - 0.07)	0.05 (0.03 - 0.07)	-	0.13 (0.07 - 0.21)
**United Republic of Tanzania**	0.86 (0.84 - 0.88)	0.85 (0.84 - 0.87)	0.01 (0.01 - 0.01)	0.01 (0.01 - 0.01)	-	0.14 (0.09 - 0.23)
**United States Virgin Islands**	0.83 (0.78 - 0.86)	0.79 (0.74 - 0.84)	0.03 (0.03 - 0.04)	0.02 (0.02 - 0.03)	-	0.11 (0.06 - 0.17)
**United States of America**	0.88 (0.85 - 0.91)	0.86 (0.82 - 0.89)	0.08 (0.06 - 0.10)	0.09 (0.07 - 0.12)	-	0.16 (0.09 - 0.23)
**Uruguay**	0.72 (0.68 - 0.76)	0.75 (0.72 - 0.78)	0.27 (0.22 - 0.31)	0.24 (0.20 - 0.27)	-	0.06 (0.04 - 0.11)
**Uzbekistan**	0.26 (0.20 - 0.34)	0.37 (0.29 - 0.46)	0.70 (0.62 - 0.77)	0.57 (0.50 - 0.65)	-	0.04 (0.02 - 0.06)
**Vanuatu**	0.63 (0.42 - 0.82)	0.69 (0.55 - 0.81)	0.08 (0.04 - 0.15)	0.07 (0.05 - 0.11)	-	0.13 (0.08 - 0.21)
**Venezuela (Bolivarian Republic of)**	0.94 (0.92 - 0.95)	0.91 (0.89 - 0.93)	0.02 (0.01 - 0.02)	0.01 (0.01 - 0.02)	-	0.12 (0.07 - 0.21)
**Viet Nam**	0.41 (0.38 - 0.43)	0.77 (0.75 - 0.79)	0.59 (0.57 - 0.61)	0.22 (0.20 - 0.24)	-	0.10 (0.06 - 0.16)
**Yemen**	0.81 (0.59 - 0.94)	0.71 (0.51 - 0.86)	0.01 (0.01 - 0.01)	0.01 (0.01 - 0.02)	-	0.12 (0.06 - 0.22)
**Zambia**	0.81 (0.75 - 0.86)	0.82 (0.79 - 0.84)	0.01 (0.01 - 0.01)	0.01 (0.01 - 0.01)	-	0.19 (0.12 - 0.28)
**Zimbabwe**	0.83 (0.79 - 0.87)	0.86 (0.84 - 0.88)	0.03 (0.02 - 0.04)	0.02 (0.02 - 0.02)	-	0.17 (0.11 - 0.26)

eTable 10. Tuberculosis age-standardized risk-deleted mortality rate for alcohol use, diabetes, smoking, and all three risk factors combined among HIV-negative men and women for 204 countries and territories in 2019

	**Alcohol**			**Diabetes**			**Smoking**			**All risk factors**		
	Male	Female	Ratio	Male	Female	Ratio	Male	Female	Ratio	Male	Female	Ratio
**Global**	15.03 (13.12-17.08)	9.51 (8.25-11.48)	1.59 (1.3-1.87)	17.62 (15.84-19.48)	9.19 (8-11.08)	1.93 (1.57-2.21)	15.47 (13.79-17.18)	9.73 (8.48-11.74)	1.6 (1.3-1.85)	10.7 (9.25-12.36)	8.38 (7.27-10.18)	1.28 (1.05-1.49)
**Central Europe, Eastern Europe, and Central Asia**	3.43 (2.75-4.25)	1.55 (1.33-1.8)	2.21 (1.84-2.61)	5.88 (5.25-6.53)	1.83 (1.61-2.09)	3.23 (2.75-3.76)	3.92 (3.43-4.5)	1.76 (1.56-2.01)	2.23 (1.9-2.61)	1.99 (1.59-2.51)	1.32 (1.13-1.53)	1.51 (1.25-1.79)
**Central Asia**	6.33 (5.26-7.52)	3.73 (3.15-4.42)	1.7 (1.45-1.99)	8.71 (7.76-9.83)	3.74 (3.16-4.4)	2.34 (2.07-2.6)	6.54 (5.71-7.45)	3.95 (3.39-4.66)	1.66 (1.45-1.88)	3.95 (3.19-4.82)	3.31 (2.78-3.92)	1.2 (1.01-1.41)
**Armenia**	2.58 (2.03-3.24)	0.49 (0.39-0.61)	5.29 (4.64-6.03)	3.21 (2.64-3.86)	0.53 (0.42-0.64)	6.13 (5.51-6.87)	1.95 (1.55-2.39)	0.56 (0.46-0.69)	3.47 (3-4.05)	1.28 (0.98-1.63)	0.43 (0.35-0.53)	2.95 (2.52-3.49)
**Azerbaijan**	5.92 (4.43-7.79)	2.42 (1.79-3.91)	2.52 (1.44-3.48)	8.71 (6.92-10.89)	2.52 (1.88-4.09)	3.57 (2.05-4.93)	5.79 (4.55-7.3)	2.71 (2.04-4.35)	2.21 (1.25-3.09)	3.38 (2.49-4.45)	2.19 (1.62-3.57)	1.59 (0.91-2.2)
**Georgia**	3.85 (2.94-5.02)	1.15 (0.93-1.41)	3.35 (2.65-4.26)	5.91 (4.81-7.15)	1.13 (0.9-1.39)	5.27 (4.46-6.21)	3.87 (3.08-4.73)	1.17 (0.94-1.43)	3.33 (2.74-3.99)	1.98 (1.48-2.58)	0.99 (0.79-1.22)	2 (1.56-2.52)
**Kazakhstan**	3.55 (2.79-4.43)	1.86 (1.44-2.25)	1.91 (1.6-2.32)	5.35 (4.43-6.32)	2.06 (1.63-2.45)	2.61 (2.27-3.17)	3.73 (3.07-4.43)	2.16 (1.73-2.55)	1.73 (1.49-2.12)	2.03 (1.58-2.6)	1.57 (1.21-1.89)	1.3 (1.08-1.59)
**Kyrgyzstan**	6.54 (5.31-7.99)	3.48 (2.87-4.11)	1.88 (1.61-2.24)	9.7 (8.37-11.2)	3.65 (3.03-4.31)	2.66 (2.39-3.04)	5.99 (5.01-7.04)	3.67 (3.04-4.31)	1.64 (1.41-1.92)	3.59 (2.87-4.46)	3.08 (2.54-3.66)	1.17 (0.98-1.41)
**Mongolia**	9.61 (7.02-12.97)	5.94 (4.4-8.04)	1.63 (1.27-2)	15.98 (12.39-20.73)	6.46 (4.83-8.57)	2.49 (2-3.02)	10.95 (8.46-14.16)	6.36 (4.8-8.45)	1.73 (1.37-2.11)	6.07 (4.45-8.28)	5.39 (4.02-7.27)	1.13 (0.88-1.42)
**Tajikistan**	9.4 (7.43-11.98)	8.1 (6.48-10.18)	1.16 (0.96-1.39)	11.07 (8.88-13.65)	7.63 (6.06-9.61)	1.46 (1.24-1.71)	9.35 (7.48-11.57)	8.29 (6.65-10.47)	1.13 (0.93-1.34)	6.61 (5.08-8.45)	7.14 (5.64-9.02)	0.93 (0.75-1.12)
**Turkmenistan**	7.81 (5.91-10.24)	4.72 (3.21-6.55)	1.7 (1.3-2.27)	11.61 (9.25-14.7)	4.77 (3.27-6.72)	2.5 (1.95-3.14)	8.9 (6.95-11.22)	4.98 (3.43-6.93)	1.83 (1.42-2.36)	5.11 (3.82-6.63)	4.2 (2.86-5.84)	1.25 (0.94-1.68)
**Uzbekistan**	8.33 (6.56-10.26)	5.36 (4.17-6.76)	1.56 (1.3-1.88)	10.53 (8.76-12.63)	5.16 (4.06-6.48)	2.05 (1.77-2.33)	8.69 (7.23-10.43)	5.56 (4.36-6.98)	1.58 (1.34-1.83)	5.5 (4.35-6.7)	4.78 (3.7-6.03)	1.16 (0.95-1.39)
**Central Europe**	0.95 (0.74-1.22)	0.41 (0.35-0.47)	2.34 (1.98-2.79)	1.71 (1.47-1.97)	0.45 (0.38-0.52)	3.81 (3.49-4.18)	1.28 (1.1-1.5)	0.41 (0.36-0.47)	3.1 (2.8-3.45)	0.56 (0.43-0.72)	0.31 (0.26-0.36)	1.79 (1.52-2.13)
**Albania**	0.25 (0.18-0.34)	0.18 (0.13-0.24)	1.42 (1.07-1.78)	0.34 (0.26-0.46)	0.18 (0.13-0.25)	1.96 (1.48-2.37)	0.22 (0.16-0.29)	0.17 (0.13-0.23)	1.29 (0.95-1.59)	0.13 (0.1-0.19)	0.15 (0.11-0.2)	0.92 (0.67-1.17)

**Bosnia and Herzegovina**	1.98 (1.48-2.61)	1.03 (0.8-1.3)	1.93 (1.55-2.4)	2.74 (2.11-3.53)	0.95 (0.73-1.2)	2.87 (2.41-3.38)	2.01 (1.57-2.57)	0.92 (0.72-1.16)	2.2 (1.82-2.64)	1.02 (0.74-1.38)	0.72 (0.55-0.92)	1.43 (1.12-1.79)
**Bulgaria**	0.79 (0.57-1.1)	0.26 (0.19-0.33)	3.11 (2.52-3.88)	1.48 (1.14-1.92)	0.3 (0.23-0.39)	4.94 (4.34-5.86)	1.05 (0.81-1.35)	0.28 (0.21-0.36)	3.81 (3.24-4.59)	0.44 (0.31-0.62)	0.19 (0.15-0.25)	2.28 (1.84-2.86)
**Croatia**	0.48 (0.34-0.66)	0.29 (0.22-0.38)	1.67 (1.38-2.03)	0.82 (0.63-1.06)	0.32 (0.24-0.41)	2.6 (2.35-2.99)	0.64 (0.49-0.81)	0.29 (0.22-0.38)	2.18 (1.93-2.52)	0.27 (0.19-0.38)	0.21 (0.15-0.27)	1.31 (1.07-1.62)
**Czechia**	0.2 (0.14-0.26)	0.1 (0.08-0.13)	1.98 (1.67-2.34)	0.34 (0.27-0.43)	0.12 (0.09-0.15)	2.84 (2.52-3.18)	0.3 (0.24-0.36)	0.12 (0.1-0.15)	2.44 (2.17-2.79)	0.11 (0.08-0.15)	0.07 (0.05-0.09)	1.53 (1.28-1.81)
**Hungary**	0.25 (0.19-0.34)	0.14 (0.11-0.18)	1.8 (1.51-2.18)	0.44 (0.35-0.55)	0.15 (0.12-0.19)	2.96 (2.73-3.23)	0.38 (0.31-0.46)	0.15 (0.12-0.18)	2.6 (2.38-2.87)	0.15 (0.11-0.21)	0.11 (0.08-0.14)	1.46 (1.21-1.77)
**Montenegro**	0.42 (0.31-0.57)	0.15 (0.12-0.19)	2.83 (2.16-3.56)	0.69 (0.56-0.86)	0.16 (0.13-0.2)	4.31 (3.42-5.22)	0.47 (0.37-0.59)	0.14 (0.11-0.18)	3.41 (2.64-4.18)	0.21 (0.15-0.29)	0.1 (0.08-0.13)	2.12 (1.57-2.72)
**North Macedonia**	1.04 (0.76-1.38)	0.62 (0.48-0.79)	1.68 (1.33-2.13)	1.6 (1.24-2.05)	0.58 (0.45-0.75)	2.76 (2.32-3.24)	1.22 (0.94-1.53)	0.55 (0.42-0.69)	2.24 (1.87-2.67)	0.56 (0.41-0.77)	0.43 (0.33-0.55)	1.31 (1.04-1.67)
**Poland**	0.75 (0.55-1.03)	0.27 (0.21-0.35)	2.76 (1.92-3.92)	1.34 (1.05-1.69)	0.28 (0.22-0.35)	4.81 (3.44-6.48)	1.07 (0.84-1.36)	0.26 (0.2-0.33)	4.16 (2.99-5.63)	0.44 (0.32-0.61)	0.2 (0.15-0.25)	2.22 (1.54-3.16)
**Romania**	2.61 (1.94-3.53)	1.07 (0.85-1.34)	2.43 (2.11-2.86)	4.98 (4.02-6.13)	1.28 (1.04-1.57)	3.88 (3.62-4.13)	3.6 (2.87-4.47)	1.17 (0.95-1.42)	3.08 (2.83-3.33)	1.58 (1.16-2.11)	0.87 (0.69-1.08)	1.81 (1.56-2.12)
**Serbia**	0.68 (0.49-0.92)	0.47 (0.36-0.59)	1.44 (1.14-1.77)	1.06 (0.82-1.36)	0.47 (0.36-0.59)	2.29 (1.91-2.71)	0.83 (0.63-1.06)	0.43 (0.34-0.54)	1.91 (1.58-2.29)	0.38 (0.27-0.51)	0.33 (0.25-0.42)	1.13 (0.89-1.41)
**Slovakia**	0.27 (0.19-0.39)	0.15 (0.11-0.21)	1.78 (1.35-2.3)	0.51 (0.39-0.65)	0.17 (0.13-0.22)	3.08 (2.4-3.76)	0.41 (0.32-0.53)	0.17 (0.13-0.22)	2.49 (1.95-3.07)	0.17 (0.12-0.24)	0.12 (0.09-0.17)	1.38 (1.05-1.81)
**Slovenia**	0.22 (0.15-0.31)	0.19 (0.14-0.24)	1.18 (0.94-1.48)	0.33 (0.25-0.43)	0.19 (0.15-0.25)	1.69 (1.45-2.01)	0.27 (0.2-0.35)	0.18 (0.14-0.24)	1.46 (1.24-1.74)	0.14 (0.09-0.2)	0.14 (0.1-0.19)	0.97 (0.76-1.23)
**Eastern Europe**	3.85 (2.94-4.97)	1.32 (1.04-1.63)	2.93 (2.27-3.75)	7.26 (6.18-8.39)	1.8 (1.48-2.14)	4.07 (3.18-5.14)	4.46 (3.71-5.31)	1.63 (1.34-1.94)	2.76 (2.14-3.55)	2.08 (1.56-2.74)	1.08 (0.85-1.34)	1.93 (1.49-2.52)
**Belarus**	1.76 (1.19-2.51)	0.58 (0.37-0.84)	3.1 (2.07-4.26)	3.62 (2.68-4.8)	0.76 (0.5-1.11)	4.86 (3.45-6.57)	2.17 (1.55-2.9)	0.68 (0.45-0.99)	3.26 (2.24-4.51)	0.93 (0.62-1.33)	0.47 (0.3-0.69)	2.03 (1.34-2.85)
**Estonia**	0.88 (0.61-1.26)	0.35 (0.26-0.48)	2.5 (2.13-2.95)	1.82 (1.36-2.33)	0.48 (0.37-0.63)	3.76 (3.42-4.24)	1.25 (0.94-1.64)	0.42 (0.32-0.55)	2.96 (2.64-3.38)	0.5 (0.34-0.72)	0.27 (0.19-0.36)	1.88 (1.58-2.27)
**Latvia**	1.4 (0.98-2)	0.47 (0.33-0.65)	3.05 (2-4.35)	2.76 (2.06-3.6)	0.59 (0.43-0.8)	4.75 (3.18-6.68)	1.91 (1.44-2.56)	0.55 (0.4-0.74)	3.56 (2.37-5.06)	0.81 (0.55-1.16)	0.37 (0.26-0.51)	2.23 (1.47-3.21)
**Lithuania**	2.88 (2.06-3.98)	0.85 (0.64-1.11)	3.4 (2.81-4.08)	5.84 (4.66-7.29)	1.24 (0.97-1.57)	4.75 (4.08-5.3)	4.11 (3.2-5.16)	1.11 (0.87-1.41)	3.7 (3.16-4.24)	1.72 (1.23-2.35)	0.68 (0.51-0.9)	2.52 (2.06-3.03)
**Republic of Moldova**	3.11 (2.39-4.02)	0.87 (0.69-1.09)	3.6 (3.01-4.3)	5.94 (4.93-6.91)	1.16 (0.97-1.39)	5.15 (4.43-5.97)	3.61 (2.89-4.38)	1.17 (0.98-1.41)	3.09 (2.5-3.72)	1.58 (1.17-2.09)	0.75 (0.6-0.94)	2.1 (1.67-2.59)

**Russian Federation**	3.33 (2.46-4.45)	1.36 (1.03-1.76)	2.48 (1.77-3.31)	6.4 (5.22-7.75)	1.82 (1.43-2.26)	3.58 (2.58-4.77)	3.79 (3.02-4.71)	1.62 (1.29-2.02)	2.37 (1.69-3.19)	1.76 (1.27-2.36)	1.1 (0.84-1.43)	1.62 (1.15-2.2)
**Ukraine**	6.3 (4.55-8.61)	1.49 (1.07-2.03)	4.31 (2.88-6.32)	11.36 (8.68-14.6)	2.12 (1.54-2.81)	5.47 (3.71-7.96)	7.42 (5.61-9.53)	1.98 (1.44-2.63)	3.82 (2.61-5.58)	3.53 (2.51-4.86)	1.25 (0.9-1.71)	2.87 (1.93-4.25)
**High-income**	0.48 (0.39-0.58)	0.29 (0.24-0.34)	1.66 (1.48-1.88)	0.73 (0.64-0.8)	0.35 (0.3-0.4)	2.06 (1.88-2.28)	0.62 (0.56-0.68)	0.35 (0.3-0.39)	1.78 (1.61-1.97)	0.32 (0.26-0.4)	0.24 (0.19-0.28)	1.37 (1.21-1.56)
**Australasia**	0.15 (0.11-0.2)	0.12 (0.09-0.15)	1.28 (0.95-1.88)	0.25 (0.19-0.32)	0.15 (0.11-0.18)	1.67 (1.27-2.39)	0.25 (0.19-0.32)	0.15 (0.11-0.18)	1.71 (1.3-2.47)	0.12 (0.08-0.16)	0.09 (0.07-0.12)	1.26 (0.94-1.88)
**Australia**	0.15 (0.1-0.21)	0.11 (0.08-0.15)	1.32 (0.92-2.1)	0.25 (0.19-0.33)	0.15 (0.1-0.19)	1.74 (1.26-2.67)	0.25 (0.19-0.33)	0.14 (0.1-0.18)	1.8 (1.3-2.74)	0.12 (0.08-0.17)	0.09 (0.06-0.12)	1.31 (0.92-2.07)
**New Zealand**	0.15 (0.12-0.19)	0.13 (0.11-0.17)	1.12 (0.95-1.3)	0.24 (0.21-0.27)	0.18 (0.15-0.21)	1.38 (1.2-1.56)	0.22 (0.2-0.25)	0.16 (0.14-0.19)	1.38 (1.21-1.55)	0.11 (0.09-0.14)	0.1 (0.08-0.13)	1.09 (0.93-1.26)
**High-income Asia Pacific**	1.23 (1-1.49)	0.61 (0.49-0.72)	2.02 (1.75-2.34)	1.8 (1.55-2.01)	0.73 (0.58-0.84)	2.47 (2.2-2.78)	1.49 (1.29-1.66)	0.76 (0.6-0.86)	1.98 (1.76-2.24)	0.82 (0.65-1.01)	0.53 (0.41-0.64)	1.54 (1.34-1.81)
**Brunei Darussalam**	18.77 (15.99-22.07)	5.94 (4.82-7.08)	3.18 (2.53-3.93)	14.25 (10.95-17.73)	4.84 (3.83-5.96)	2.96 (2.31-3.69)	15.54 (13.05-18.39)	5.63 (4.57-6.7)	2.78 (2.17-3.43)	11.44 (8.67-14.44)	4.51 (3.56-5.57)	2.55 (1.96-3.2)
**Japan**	0.8 (0.64-0.97)	0.39 (0.29-0.47)	2.09 (1.8-2.51)	1.1 (0.92-1.21)	0.45 (0.33-0.52)	2.47 (2.23-2.86)	0.95 (0.8-1.04)	0.45 (0.34-0.52)	2.1 (1.88-2.45)	0.57 (0.44-0.7)	0.34 (0.25-0.41)	1.67 (1.42-2.02)
**Republic of Korea**	3.43 (2.67-4.3)	1.79 (1.34-2.22)	1.93 (1.53-2.36)	5.36 (4.59-6.27)	2.19 (1.72-2.69)	2.46 (2.02-2.92)	4.35 (3.72-5.06)	2.28 (1.8-2.74)	1.92 (1.56-2.29)	2.18 (1.65-2.81)	1.52 (1.13-1.92)	1.45 (1.13-1.79)
**Singapore**	1.15 (0.97-1.33)	0.37 (0.29-0.47)	3.16 (2.54-3.85)	1.14 (0.96-1.33)	0.35 (0.27-0.45)	3.29 (2.65-3.96)	1.13 (0.97-1.3)	0.37 (0.3-0.47)	3.05 (2.46-3.65)	0.85 (0.7-1.02)	0.32 (0.25-0.41)	2.73 (2.18-3.31)
**High-income North America**	0.16 (0.13-0.19)	0.09 (0.08-0.11)	1.65 (1.48-1.82)	0.21 (0.19-0.23)	0.11 (0.1-0.12)	1.88 (1.73-2.02)	0.2 (0.18-0.21)	0.11 (0.1-0.12)	1.83 (1.67-1.97)	0.1 (0.08-0.12)	0.07 (0.06-0.08)	1.47 (1.31-1.63)
**Canada**	0.17 (0.13-0.21)	0.11 (0.09-0.14)	1.48 (1.29-1.8)	0.25 (0.21-0.29)	0.14 (0.12-0.17)	1.74 (1.56-2.05)	0.23 (0.19-0.26)	0.13 (0.11-0.15)	1.69 (1.5-2)	0.12 (0.1-0.16)	0.09 (0.07-0.11)	1.39 (1.2-1.68)
**United States of America**	0.15 (0.13-0.18)	0.09 (0.08-0.1)	1.67 (1.48-1.87)	0.2 (0.18-0.23)	0.11 (0.09-0.12)	1.9 (1.71-2.08)	0.19 (0.17-0.21)	0.1 (0.09-0.11)	1.85 (1.66-2.02)	0.1 (0.08-0.12)	0.07 (0.05-0.08)	1.48 (1.31-1.66)
**Southern Latin America**	1.15 (0.94-1.43)	0.75 (0.62-0.91)	1.53 (1.33-1.76)	1.89 (1.72-2.05)	0.94 (0.82-1.09)	2.01 (1.82-2.2)	1.68 (1.56-1.81)	0.88 (0.77-1.02)	1.91 (1.72-2.09)	0.8 (0.65-1)	0.57 (0.46-0.7)	1.4 (1.22-1.61)
**Argentina**	1.02 (0.83-1.27)	0.68 (0.55-0.83)	1.5 (1.3-1.7)	1.73 (1.57-1.9)	0.88 (0.77-1.02)	1.98 (1.79-2.15)	1.47 (1.34-1.61)	0.79 (0.69-0.91)	1.87 (1.69-2.04)	0.69 (0.55-0.87)	0.51 (0.41-0.63)	1.35 (1.17-1.55)
**Chile**	1.45 (1.17-1.79)	0.98 (0.79-1.21)	1.48 (1.26-1.73)	2.28 (2.01-2.56)	1.17 (0.96-1.4)	1.96 (1.72-2.19)	2.21 (1.99-2.44)	1.19 (1-1.4)	1.86 (1.64-2.07)	1.05 (0.83-1.33)	0.76 (0.6-0.95)	1.39 (1.18-1.64)

**Uruguay**	0.95 (0.77-1.18)	0.5 (0.39-0.62)	1.92 (1.62-2.29)	1.53 (1.37-1.7)	0.64 (0.53-0.76)	2.42 (2.1-2.76)	1.23 (1.1-1.38)	0.57 (0.47-0.68)	2.18 (1.88-2.51)	0.63 (0.5-0.79)	0.39 (0.3-0.49)	1.63 (1.36-1.95)
**Western Europe**	0.25 (0.2-0.31)	0.19 (0.15-0.22)	1.33 (1.16-1.55)	0.42 (0.37-0.46)	0.24 (0.2-0.27)	1.78 (1.59-2.01)	0.37 (0.34-0.41)	0.23 (0.2-0.27)	1.6 (1.42-1.8)	0.17 (0.13-0.22)	0.15 (0.12-0.18)	1.15 (1-1.35)
**Andorra**	0.1 (0.08-0.14)	0.12 (0.08-0.16)	0.91 (0.7-1.16)	0.18 (0.14-0.22)	0.15 (0.11-0.2)	1.22 (0.95-1.5)	0.15 (0.12-0.19)	0.15 (0.11-0.2)	1.07 (0.82-1.32)	0.07 (0.05-0.1)	0.09 (0.06-0.13)	0.77 (0.58-0.99)
**Austria**	0.2 (0.16-0.25)	0.12 (0.1-0.14)	1.69 (1.48-1.92)	0.36 (0.32-0.41)	0.16 (0.14-0.18)	2.32 (2.15-2.51)	0.28 (0.24-0.31)	0.14 (0.12-0.16)	1.97 (1.78-2.17)	0.12 (0.09-0.16)	0.09 (0.07-0.11)	1.39 (1.2-1.59)
**Belgium**	0.24 (0.19-0.3)	0.15 (0.12-0.19)	1.57 (1.39-1.83)	0.42 (0.36-0.47)	0.21 (0.18-0.25)	1.97 (1.81-2.25)	0.35 (0.3-0.39)	0.2 (0.16-0.23)	1.76 (1.6-2.01)	0.16 (0.12-0.2)	0.11 (0.09-0.14)	1.37 (1.19-1.6)
**Cyprus**	0.19 (0.15-0.25)	0.18 (0.14-0.22)	1.1 (0.84-1.39)	0.31 (0.25-0.37)	0.18 (0.14-0.22)	1.73 (1.41-2.11)	0.26 (0.22-0.31)	0.19 (0.16-0.24)	1.35 (1.08-1.66)	0.12 (0.09-0.16)	0.14 (0.11-0.18)	0.86 (0.65-1.11)
**Denmark**	0.17 (0.13-0.21)	0.1 (0.08-0.13)	1.58 (1.35-1.79)	0.29 (0.24-0.33)	0.15 (0.13-0.18)	1.91 (1.68-2.15)	0.22 (0.19-0.25)	0.13 (0.11-0.15)	1.73 (1.5-1.95)	0.1 (0.08-0.14)	0.07 (0.06-0.09)	1.41 (1.2-1.6)
**Finland**	0.31 (0.24-0.38)	0.26 (0.19-0.32)	1.21 (0.99-1.6)	0.43 (0.36-0.51)	0.28 (0.21-0.34)	1.56 (1.32-2.01)	0.42 (0.35-0.49)	0.29 (0.23-0.35)	1.44 (1.23-1.87)	0.22 (0.17-0.28)	0.2 (0.15-0.25)	1.13 (0.93-1.51)
**France**	0.37 (0.28-0.47)	0.32 (0.23-0.43)	1.14 (0.93-1.4)	0.7 (0.6-0.81)	0.45 (0.35-0.57)	1.56 (1.31-1.87)	0.62 (0.52-0.71)	0.44 (0.34-0.55)	1.4 (1.17-1.68)	0.28 (0.21-0.36)	0.27 (0.2-0.37)	1.01 (0.82-1.25)
**Germany**	0.15 (0.12-0.19)	0.09 (0.07-0.11)	1.61 (1.42-1.83)	0.27 (0.24-0.31)	0.13 (0.11-0.15)	2.07 (1.9-2.25)	0.25 (0.22-0.27)	0.13 (0.11-0.15)	1.85 (1.7-2.02)	0.1 (0.07-0.13)	0.07 (0.05-0.09)	1.41 (1.23-1.61)
**Greece**	0.37 (0.29-0.47)	0.31 (0.25-0.36)	1.19 (0.98-1.43)	0.61 (0.53-0.7)	0.32 (0.27-0.38)	1.91 (1.69-2.12)	0.46 (0.39-0.53)	0.3 (0.25-0.35)	1.54 (1.36-1.71)	0.22 (0.16-0.28)	0.24 (0.19-0.28)	0.92 (0.75-1.1)
**Iceland**	0.11 (0.09-0.14)	0.16 (0.12-0.2)	0.71 (0.6-0.86)	0.18 (0.15-0.2)	0.19 (0.16-0.23)	0.92 (0.79-1.05)	0.15 (0.13-0.18)	0.18 (0.15-0.22)	0.85 (0.73-0.99)	0.08 (0.06-0.1)	0.13 (0.1-0.16)	0.63 (0.52-0.77)
**Ireland**	0.25 (0.19-0.34)	0.24 (0.19-0.3)	1.07 (0.81-1.46)	0.41 (0.32-0.52)	0.3 (0.24-0.37)	1.35 (1.06-1.83)	0.36 (0.29-0.46)	0.28 (0.23-0.34)	1.31 (1.03-1.75)	0.17 (0.12-0.24)	0.18 (0.14-0.23)	0.98 (0.75-1.34)
**Israel**	0.22 (0.19-0.26)	0.16 (0.13-0.19)	1.41 (1.21-1.64)	0.24 (0.21-0.28)	0.15 (0.12-0.18)	1.64 (1.42-1.86)	0.22 (0.19-0.25)	0.15 (0.13-0.18)	1.44 (1.25-1.65)	0.16 (0.13-0.19)	0.12 (0.1-0.15)	1.26 (1.08-1.46)
**Italy**	0.21 (0.16-0.26)	0.15 (0.12-0.17)	1.39 (1.16-1.74)	0.32 (0.27-0.37)	0.17 (0.14-0.2)	1.87 (1.61-2.26)	0.29 (0.25-0.33)	0.17 (0.15-0.19)	1.69 (1.46-2.04)	0.14 (0.11-0.18)	0.11 (0.09-0.14)	1.2 (1.01-1.5)
**Luxembourg**	0.1 (0.07-0.13)	0.1 (0.07-0.12)	0.99 (0.87-1.15)	0.17 (0.14-0.2)	0.12 (0.1-0.15)	1.37 (1.25-1.49)	0.15 (0.13-0.18)	0.12 (0.1-0.15)	1.27 (1.15-1.38)	0.06 (0.05-0.08)	0.07 (0.05-0.09)	0.87 (0.75-1.01)
**Malta**	0.11 (0.09-0.14)	0.06 (0.05-0.07)	2 (1.65-2.41)	0.17 (0.14-0.2)	0.06 (0.05-0.08)	2.76 (2.35-3.19)	0.15 (0.13-0.18)	0.06 (0.05-0.08)	2.42 (2.05-2.8)	0.08 (0.06-0.1)	0.04 (0.03-0.06)	1.79 (1.47-2.15)
**Monaco**	0.87 (0.52-1.37)	0.41 (0.27-0.57)	2.13 (1.53-3)	1.16 (0.92-1.42)	0.45 (0.33-0.58)	2.62 (2.06-3.37)	0.98 (0.78-1.19)	0.43 (0.32-0.56)	2.29 (1.76-2.97)	0.59 (0.35-0.92)	0.32 (0.21-0.45)	1.82 (1.26-2.59)

**Netherlands**	0.17 (0.14-0.21)	0.16 (0.13-0.2)	1.07 (0.92-1.24)	0.29 (0.25-0.33)	0.22 (0.18-0.25)	1.33 (1.2-1.49)	0.25 (0.22-0.29)	0.2 (0.17-0.24)	1.25 (1.13-1.43)	0.12 (0.1-0.15)	0.12 (0.1-0.16)	0.98 (0.84-1.15)
**Norway**	0.3 (0.24-0.36)	0.27 (0.22-0.33)	1.1 (0.92-1.29)	0.42 (0.36-0.47)	0.31 (0.25-0.35)	1.37 (1.21-1.56)	0.43 (0.38-0.47)	0.32 (0.27-0.36)	1.33 (1.18-1.51)	0.23 (0.18-0.29)	0.22 (0.18-0.27)	1.05 (0.88-1.24)
**Portugal**	0.74 (0.59-0.94)	0.4 (0.32-0.48)	1.87 (1.56-2.27)	1.3 (1.14-1.45)	0.44 (0.36-0.53)	2.96 (2.62-3.31)	1.17 (1.05-1.3)	0.48 (0.4-0.56)	2.48 (2.17-2.77)	0.49 (0.38-0.63)	0.32 (0.25-0.39)	1.55 (1.28-1.87)
**San Marino**	0.13 (0.08-0.26)	0.24 (0.15-0.39)	0.55 (0.41-0.75)	0.21 (0.14-0.29)	0.29 (0.19-0.44)	0.72 (0.57-0.9)	0.18 (0.12-0.25)	0.29 (0.19-0.43)	0.64 (0.5-0.81)	0.09 (0.05-0.18)	0.19 (0.12-0.32)	0.48 (0.35-0.66)
**Spain**	0.28 (0.23-0.36)	0.2 (0.16-0.23)	1.44 (1.24-1.68)	0.44 (0.39-0.5)	0.22 (0.18-0.26)	2.01 (1.81-2.22)	0.38 (0.34-0.42)	0.23 (0.19-0.26)	1.68 (1.51-1.87)	0.18 (0.14-0.23)	0.16 (0.13-0.19)	1.16 (0.99-1.35)
**Sweden**	0.22 (0.17-0.28)	0.17 (0.13-0.22)	1.28 (1.09-1.49)	0.34 (0.29-0.39)	0.24 (0.19-0.29)	1.42 (1.25-1.61)	0.31 (0.27-0.36)	0.23 (0.18-0.27)	1.39 (1.21-1.59)	0.16 (0.12-0.2)	0.13 (0.1-0.17)	1.22 (1.03-1.43)
**Switzerland**	0.14 (0.11-0.18)	0.09 (0.07-0.11)	1.63 (1.43-1.82)	0.24 (0.21-0.28)	0.13 (0.11-0.15)	1.93 (1.76-2.08)	0.21 (0.18-0.24)	0.12 (0.1-0.14)	1.76 (1.6-1.92)	0.09 (0.07-0.13)	0.07 (0.05-0.09)	1.44 (1.26-1.63)
**United Kingdom**	0.23 (0.18-0.28)	0.17 (0.14-0.2)	1.31 (1.16-1.51)	0.34 (0.3-0.37)	0.2 (0.17-0.23)	1.66 (1.52-1.85)	0.32 (0.29-0.34)	0.2 (0.17-0.22)	1.63 (1.49-1.82)	0.15 (0.12-0.19)	0.12 (0.1-0.15)	1.21 (1.06-1.4)
**Latin America and Caribbean**	2.83 (2.39-3.37)	1.78 (1.5-2.19)	1.59 (1.33-1.84)	3.72 (3.28-4.24)	1.79 (1.5-2.21)	2.09 (1.77-2.33)	3.62 (3.2-4.1)	1.9 (1.62-2.33)	1.91 (1.61-2.13)	2.13 (1.77-2.59)	1.5 (1.24-1.87)	1.42 (1.19-1.65)
**Andean Latin America**	6.33 (4.8-8.2)	4.8 (3.73-6.12)	1.32 (1.1-1.54)	8.43 (6.57-10.7)	4.88 (3.82-6.26)	1.73 (1.46-1.99)	8.33 (6.5-10.43)	5.25 (4.16-6.73)	1.59 (1.34-1.83)	5.21 (3.91-6.9)	4.28 (3.31-5.54)	1.22 (1.02-1.42)
**Bolivia (Plurinational State of)**	13.63 (9.73-18.29)	12.85 (8.73-17.09)	1.08 (0.77-1.53)	17.29 (12.67-22.75)	12.73 (8.63-17.01)	1.38 (1.01-1.94)	16.91 (12.4-21.87)	13.85 (9.41-18.26)	1.24 (0.91-1.76)	10.77 (7.66-14.31)	11.31 (7.68-15.16)	0.97 (0.7-1.37)
**Ecuador**	4.92 (3.76-6.4)	2.68 (2.14-3.47)	1.84 (1.56-2.14)	6.02 (4.69-7.79)	2.54 (1.99-3.29)	2.38 (2.02-2.7)	5.89 (4.65-7.6)	2.79 (2.23-3.57)	2.12 (1.81-2.41)	3.84 (2.91-4.98)	2.31 (1.81-2.98)	1.67 (1.41-1.94)
**Peru**	5.08 (3.55-7.09)	3.56 (2.48-5.27)	1.44 (1.04-1.82)	7.17 (5.21-9.73)	3.79 (2.67-5.55)	1.92 (1.38-2.37)	7.21 (5.25-9.68)	4.02 (2.84-5.83)	1.82 (1.31-2.25)	4.37 (3.05-6.1)	3.24 (2.24-4.83)	1.37 (0.98-1.73)
**Caribbean**	3.6 (2.77-4.55)	3.56 (2.63-5.39)	1.03 (0.69-1.3)	4.91 (3.92-5.94)	3.61 (2.71-5.47)	1.39 (0.94-1.72)	4.75 (3.83-5.72)	3.88 (2.9-5.83)	1.25 (0.84-1.56)	2.84 (2.19-3.62)	3.06 (2.28-4.69)	0.95 (0.63-1.2)
**Antigua and Barbuda**	0.36 (0.29-0.45)	0.24 (0.19-0.32)	1.53 (1.19-1.91)	0.45 (0.37-0.54)	0.23 (0.18-0.31)	1.97 (1.58-2.36)	0.47 (0.39-0.55)	0.26 (0.2-0.34)	1.83 (1.46-2.18)	0.27 (0.21-0.33)	0.19 (0.15-0.26)	1.4 (1.08-1.75)
**Bahamas**	2.07 (1.49-2.77)	0.8 (0.59-1.06)	2.63 (2-3.58)	2.59 (2.05-3.22)	0.79 (0.56-1.07)	3.33 (2.66-4.23)	2.61 (2.09-3.24)	0.87 (0.63-1.18)	3.05 (2.44-3.88)	1.54 (1.12-2.08)	0.67 (0.48-0.91)	2.33 (1.78-3.18)
**Barbados**	0.35 (0.27-0.45)	0.21 (0.16-0.28)	1.68 (1.33-2.26)	0.52 (0.42-0.63)	0.21 (0.16-0.28)	2.48 (2.01-3.13)	0.52 (0.42-0.62)	0.24 (0.18-0.31)	2.19 (1.77-2.78)	0.26 (0.2-0.34)	0.18 (0.13-0.24)	1.48 (1.17-1.99)

**Belize**	4.47 (3.62-5.56)	1.93 (1.58-2.39)	2.32 (1.91-2.81)	6.2 (5.21-7.26)	1.85 (1.51-2.28)	3.36 (2.85-3.75)	5.62 (4.75-6.61)	2.04 (1.66-2.5)	2.78 (2.34-3.15)	3.26 (2.61-4.04)	1.63 (1.32-2.01)	2.01 (1.64-2.42)
**Cuba**	0.25 (0.19-0.32)	0.09 (0.07-0.11)	2.8 (2.31-3.36)	0.31 (0.24-0.38)	0.09 (0.07-0.11)	3.55 (2.96-4.06)	0.26 (0.2-0.32)	0.09 (0.07-0.11)	2.97 (2.48-3.46)	0.15 (0.11-0.19)	0.07 (0.05-0.09)	2.15 (1.77-2.6)
**Dominica**	3.43 (2.57-4.48)	1.46 (1.04-1.93)	2.38 (1.85-3.16)	4.36 (3.44-5.52)	1.48 (1.06-1.94)	2.98 (2.39-3.88)	4.69 (3.73-5.81)	1.66 (1.17-2.15)	2.86 (2.28-3.74)	2.44 (1.82-3.21)	1.21 (0.88-1.6)	2.03 (1.58-2.71)
**Dominican Republic**	6.75 (4.94-9.16)	3.86 (2.76-5.79)	1.79 (1.23-2.24)	9.48 (7.02-12.83)	4.26 (3.03-6.45)	2.28 (1.56-2.84)	8.43 (6.21-11.2)	4.12 (2.94-6.14)	2.09 (1.45-2.59)	5.09 (3.67-6.93)	3.2 (2.29-4.83)	1.63 (1.13-2.04)
**Grenada**	0.51 (0.42-0.63)	0.21 (0.17-0.26)	2.47 (2.03-2.97)	0.7 (0.59-0.81)	0.22 (0.17-0.27)	3.25 (2.77-3.74)	0.74 (0.65-0.84)	0.25 (0.2-0.31)	3.02 (2.58-3.49)	0.37 (0.29-0.46)	0.17 (0.13-0.22)	2.15 (1.76-2.6)
**Guyana**	8.11 (6.02-10.97)	3.92 (2.7-5.66)	2.11 (1.54-2.81)	11.47 (8.82-14.79)	3.5 (2.39-5.11)	3.35 (2.58-4.17)	11.54 (8.97-14.77)	4.06 (2.78-5.86)	2.91 (2.22-3.64)	5.5 (4.04-7.47)	3.16 (2.13-4.6)	1.78 (1.29-2.38)
**Haiti**	9.98 (6.37-13.98)	12.52 (8.46-18.82)	0.82 (0.46-1.16)	13 (8.47-17.79)	11.93 (8.21-17.9)	1.12 (0.62-1.58)	13.88 (8.88-18.82)	13.68 (9.38-20.62)	1.04 (0.58-1.47)	8.02 (5.14-11.06)	10.45 (7.05-15.81)	0.79 (0.44-1.12)
**Jamaica**	0.39 (0.29-0.51)	0.13 (0.09-0.17)	3.08 (2.37-3.86)	0.47 (0.36-0.61)	0.12 (0.09-0.16)	3.94 (3.08-4.74)	0.44 (0.34-0.57)	0.13 (0.1-0.18)	3.34 (2.62-4.05)	0.26 (0.19-0.35)	0.1 (0.07-0.14)	2.59 (1.99-3.25)
**Saint Kitts and Nevis**	2.12 (1.44-2.9)	0.6 (0.41-0.82)	3.58 (2.75-4.64)	2.2 (1.59-2.75)	0.54 (0.38-0.74)	4.11 (3.31-4.87)	2.38 (1.81-2.92)	0.62 (0.44-0.83)	3.89 (3.16-4.64)	1.58 (1.06-2.18)	0.49 (0.33-0.68)	3.24 (2.48-4.2)
**Saint Lucia**	2.01 (1.58-2.54)	0.77 (0.58-1)	2.63 (2.18-3.16)	2.75 (2.27-3.33)	0.77 (0.6-1.01)	3.61 (3.07-4.11)	2.83 (2.37-3.38)	0.91 (0.71-1.17)	3.12 (2.66-3.54)	1.4 (1.08-1.79)	0.6 (0.45-0.78)	2.35 (1.94-2.82)
**Saint Vincent and the Grenadines**	2.15 (1.72-2.69)	0.54 (0.45-0.67)	3.99 (3.41-4.63)	2.9 (2.45-3.46)	0.54 (0.45-0.67)	5.34 (4.7-5.92)	2.99 (2.56-3.52)	0.63 (0.53-0.77)	4.75 (4.18-5.29)	1.52 (1.2-1.94)	0.44 (0.36-0.54)	3.48 (2.96-4.06)
**Suriname**	1.74 (1.33-2.25)	0.79 (0.63-0.99)	2.2 (1.74-2.7)	2.18 (1.72-2.72)	0.78 (0.61-0.97)	2.83 (2.3-3.37)	2.01 (1.6-2.45)	0.86 (0.68-1.05)	2.35 (1.9-2.83)	1.1 (0.82-1.44)	0.63 (0.49-0.78)	1.75 (1.37-2.14)
**Trinidad and Tobago**	1.21 (0.87-1.65)	0.33 (0.23-0.47)	3.73 (2.93-4.56)	1.51 (1.11-2.04)	0.32 (0.22-0.46)	4.82 (3.95-5.6)	1.51 (1.12-1.97)	0.37 (0.26-0.52)	4.13 (3.35-4.83)	0.76 (0.54-1.05)	0.25 (0.18-0.37)	3.03 (2.38-3.7)
**Central Latin America**	2.27 (1.89-2.77)	1.44 (1.15-1.88)	1.6 (1.25-1.95)	2.78 (2.34-3.33)	1.37 (1.08-1.76)	2.06 (1.6-2.48)	2.85 (2.42-3.36)	1.52 (1.24-1.96)	1.89 (1.47-2.28)	1.63 (1.32-2.02)	1.17 (0.94-1.54)	1.4 (1.1-1.72)
**Colombia**	1.79 (1.33-2.36)	1.03 (0.75-1.46)	1.76 (1.32-2.12)	2.13 (1.6-2.78)	0.98 (0.71-1.39)	2.2 (1.64-2.58)	2.22 (1.69-2.85)	1.04 (0.76-1.48)	2.16 (1.61-2.52)	1.36 (1.01-1.81)	0.85 (0.62-1.22)	1.61 (1.19-1.94)
**Costa Rica**	0.89 (0.67-1.17)	0.54 (0.38-0.77)	1.67 (1.29-2.03)	1.07 (0.82-1.41)	0.51 (0.36-0.73)	2.11 (1.64-2.43)	1.05 (0.8-1.37)	0.56 (0.39-0.8)	1.93 (1.5-2.22)	0.62 (0.45-0.82)	0.44 (0.31-0.64)	1.41 (1.09-1.72)
**El Salvador**	1.76 (1.3-2.32)	0.74 (0.55-0.99)	2.39 (1.91-2.85)	1.99 (1.49-2.62)	0.69 (0.51-0.95)	2.89 (2.36-3.4)	2.05 (1.56-2.71)	0.77 (0.57-1.04)	2.68 (2.19-3.17)	1.29 (0.95-1.73)	0.62 (0.46-0.84)	2.11 (1.7-2.52)
**Guatemala**	3.47 (2.64-4.48)	2.06 (1.55-2.82)	1.69 (1.42-1.94)	3.69 (2.85-4.7)	1.85 (1.39-2.54)	2 (1.68-2.22)	3.77 (2.92-4.73)	2.11 (1.58-2.83)	1.8 (1.5-2.01)	2.53 (1.9-3.29)	1.7 (1.27-2.32)	1.5 (1.25-1.72)

**Honduras**	8.38 (6.81-10.46)	7.24 (5.16-12.83)	1.21 (0.62-1.62)	9.02 (7.19-11.37)	6.42 (4.51-11.49)	1.47 (0.77-1.97)	8.73 (7.13-10.88)	7.2 (5.12-12.59)	1.27 (0.67-1.7)	5.57 (4.38-7.03)	5.85 (4.09-10.52)	1 (0.51-1.35)
**Mexico**	1.98 (1.56-2.5)	1.14 (0.89-1.49)	1.77 (1.25-2.39)	2.54 (2.06-3.13)	1.1 (0.84-1.44)	2.35 (1.68-3.16)	2.65 (2.16-3.21)	1.26 (0.98-1.63)	2.13 (1.51-2.9)	1.4 (1.07-1.76)	0.92 (0.69-1.19)	1.55 (1.1-2.1)
**Nicaragua**	3.21 (2.55-3.95)	1.97 (1.6-2.43)	1.64 (1.31-1.99)	3.68 (2.89-4.58)	1.77 (1.42-2.25)	2.09 (1.68-2.46)	3.64 (2.92-4.45)	2.01 (1.66-2.49)	1.82 (1.45-2.17)	2.28 (1.77-2.89)	1.64 (1.3-2.07)	1.4 (1.12-1.69)
**Panama**	3.68 (2.68-4.93)	2.96 (2.06-4.33)	1.26 (0.96-1.54)	4.86 (3.64-6.47)	3.03 (2.1-4.44)	1.63 (1.25-1.91)	4.98 (3.72-6.53)	3.3 (2.3-4.81)	1.53 (1.18-1.81)	2.76 (2.01-3.78)	2.54 (1.74-3.73)	1.1 (0.84-1.35)
**Venezuela (Bolivarian Republic of)**	2.57 (1.8-3.56)	1.9 (1.26-2.74)	1.39 (0.97-2.01)	3.23 (2.31-4.38)	1.86 (1.25-2.66)	1.78 (1.25-2.56)	3.19 (2.28-4.29)	1.99 (1.35-2.86)	1.64 (1.15-2.36)	1.84 (1.3-2.59)	1.55 (1.05-2.25)	1.22 (0.86-1.78)
**Tropical Latin America**	2.34 (2.02-2.73)	1.09 (0.95-1.25)	2.15 (1.82-2.52)	3.22 (2.97-3.48)	1.14 (1-1.3)	2.85 (2.49-3.2)	2.96 (2.74-3.18)	1.14 (1.01-1.28)	2.62 (2.28-2.95)	1.7 (1.44-2)	0.89 (0.76-1.04)	1.92 (1.63-2.23)
**Brazil**	2.31 (1.99-2.69)	1.08 (0.94-1.24)	2.15 (1.81-2.52)	3.16 (2.9-3.41)	1.13 (0.99-1.29)	2.82 (2.45-3.19)	2.92 (2.7-3.13)	1.12 (1-1.27)	2.61 (2.26-2.95)	1.68 (1.43-1.98)	0.88 (0.75-1.03)	1.92 (1.62-2.24)
**Paraguay**	3.57 (2.55-4.93)	1.6 (1.19-2.14)	2.24 (1.78-2.75)	5.74 (4.3-7.66)	1.67 (1.26-2.24)	3.45 (2.85-4.1)	4.73 (3.51-6.23)	1.66 (1.25-2.21)	2.85 (2.35-3.38)	2.33 (1.66-3.25)	1.29 (0.95-1.74)	1.81 (1.44-2.23)
**North Africa and Middle East**	3.36 (2.79-4.08)	3.35 (2.64-5.06)	1.02 (0.64-1.27)	3 (2.45-3.65)	2.89 (2.23-4.34)	1.06 (0.67-1.31)	2.67 (2.19-3.28)	3.28 (2.59-4.98)	0.83 (0.51-1.04)	2.22 (1.8-2.72)	2.8 (2.16-4.23)	0.81 (0.5-1.01)
**North Africa and Middle East**	3.36 (2.79-4.08)	3.35 (2.64-5.06)	1.02 (0.64-1.27)	3 (2.45-3.65)	2.89 (2.23-4.34)	1.06 (0.67-1.31)	2.67 (2.19-3.28)	3.28 (2.59-4.98)	0.83 (0.51-1.04)	2.22 (1.8-2.72)	2.8 (2.16-4.23)	0.81 (0.5-1.01)
**Afghanistan**	23.26 (16.18-32.14)	20.37 (6.47-29.61)	1.32 (0.76-3.94)	19.94 (13.54-28.21)	16.91 (5.51-24.81)	1.36 (0.78-3.99)	19.59 (13.58-27.37)	19.89 (6.3-28.97)	1.14 (0.65-3.41)	16.52 (11.3-23.58)	16.47 (5.37-24.17)	1.16 (0.66-3.39)
**Algeria**	1.54 (1.19-1.96)	1.39 (1.08-1.91)	1.13 (0.72-1.48)	1.39 (1.04-1.79)	1.18 (0.89-1.67)	1.2 (0.76-1.59)	1.15 (0.88-1.45)	1.38 (1.07-1.89)	0.85 (0.53-1.11)	0.92 (0.69-1.16)	1.14 (0.85-1.61)	0.82 (0.52-1.07)
**Bahrain**	2.03 (1.62-2.49)	1.06 (0.82-1.55)	1.95 (1.28-2.44)	1.59 (1.17-2.04)	0.81 (0.58-1.23)	2 (1.31-2.49)	1.65 (1.31-2.06)	1.02 (0.79-1.51)	1.64 (1.09-2.08)	1.13 (0.84-1.48)	0.76 (0.55-1.15)	1.52 (1-1.91)
**Egypt**	1.56 (1.14-2.17)	0.78 (0.5-1.98)	2.18 (0.83-2.95)	1.43 (1.03-2.02)	0.68 (0.44-1.75)	2.27 (0.86-3.09)	1.03 (0.76-1.43)	0.77 (0.5-1.97)	1.45 (0.55-1.98)	0.88 (0.64-1.22)	0.67 (0.43-1.72)	1.42 (0.53-1.93)
**Iran (Islamic Republic of)**	1.28 (1.18-1.39)	0.99 (0.87-1.27)	1.31 (1-1.47)	1.17 (1.04-1.29)	0.86 (0.73-1.13)	1.37 (1.05-1.54)	1.06 (0.96-1.16)	0.97 (0.85-1.24)	1.1 (0.84-1.25)	0.88 (0.77-0.98)	0.82 (0.7-1.08)	1.08 (0.82-1.22)
**Iraq**	4.31 (3.41-5.33)	3.26 (2.48-4.48)	1.34 (1-1.61)	3.78 (2.95-4.76)	2.73 (2.06-3.76)	1.4 (1.06-1.68)	2.92 (2.29-3.69)	3.11 (2.38-4.28)	0.95 (0.7-1.16)	2.33 (1.78-2.99)	2.57 (1.94-3.54)	0.92 (0.68-1.12)
**Jordan**	0.57 (0.45-0.72)	0.46 (0.36-0.62)	1.27 (0.87-1.73)	0.49 (0.37-0.63)	0.39 (0.3-0.54)	1.27 (0.87-1.72)	0.39 (0.3-0.49)	0.42 (0.33-0.57)	0.93 (0.63-1.26)	0.3 (0.23-0.4)	0.36 (0.27-0.49)	0.87 (0.59-1.19)

**Kuwait**	1.27 (0.99-1.59)	1.05 (0.75-1.49)	1.25 (0.8-1.73)	1 (0.75-1.28)	0.86 (0.59-1.23)	1.21 (0.77-1.68)	0.95 (0.74-1.21)	1.02 (0.72-1.44)	0.97 (0.61-1.36)	0.74 (0.55-0.97)	0.83 (0.56-1.19)	0.93 (0.59-1.29)
**Lebanon**	0.82 (0.54-1.35)	0.68 (0.36-1.98)	1.44 (0.33-2.63)	0.77 (0.5-1.27)	0.59 (0.3-1.74)	1.57 (0.36-2.91)	0.58 (0.37-0.94)	0.53 (0.28-1.55)	1.29 (0.3-2.44)	0.43 (0.29-0.72)	0.45 (0.23-1.3)	1.15 (0.27-2.14)
**Libya**	1.96 (1.21-2.6)	1.22 (0.58-1.72)	1.7 (0.91-3.14)	1.64 (1.06-2.22)	1.01 (0.48-1.42)	1.73 (0.93-3.19)	1.42 (0.9-1.91)	1.22 (0.58-1.71)	1.24 (0.68-2.31)	1.13 (0.73-1.53)	0.99 (0.48-1.4)	1.22 (0.66-2.27)
**Morocco**	8.69 (6.45-10.99)	18.1 (13.03-41.02)	0.51 (0.21-0.69)	7.73 (5.65-9.89)	15.56 (10.96-35.64)	0.53 (0.22-0.71)	7.44 (5.58-9.35)	17.9 (12.9-40.62)	0.44 (0.18-0.6)	6.09 (4.47-7.84)	15.38 (10.83-35.24)	0.42 (0.17-0.57)
**Oman**	1.53 (1.13-1.94)	0.91 (0.61-1.63)	1.76 (0.85-2.61)	1.29 (0.94-1.67)	0.78 (0.52-1.42)	1.75 (0.84-2.64)	1.33 (0.98-1.69)	0.9 (0.6-1.6)	1.56 (0.75-2.3)	1.07 (0.79-1.39)	0.76 (0.51-1.38)	1.49 (0.72-2.27)
**Palestine**	0.81 (0.67-0.98)	1.33 (0.75-1.7)	0.63 (0.47-1.1)	0.72 (0.58-0.89)	1.13 (0.65-1.48)	0.66 (0.49-1.14)	0.6 (0.48-0.74)	1.31 (0.74-1.66)	0.47 (0.35-0.78)	0.45 (0.35-0.57)	1.06 (0.6-1.39)	0.44 (0.32-0.74)
**Qatar**	1.49 (1.12-1.93)	0.96 (0.73-1.47)	1.58 (0.95-2.08)	1.1 (0.78-1.51)	0.71 (0.5-1.12)	1.59 (0.95-2.1)	1.28 (0.96-1.68)	0.95 (0.73-1.46)	1.37 (0.81-1.81)	0.89 (0.62-1.22)	0.69 (0.49-1.1)	1.31 (0.78-1.73)
**Saudi Arabia**	7.34 (5.96-9.1)	6.53 (4.76-10.54)	1.16 (0.69-1.49)	6.03 (4.71-7.56)	5.4 (3.93-8.85)	1.15 (0.68-1.48)	6.09 (4.88-7.6)	6.44 (4.68-10.49)	0.97 (0.58-1.26)	4.81 (3.74-6.11)	5.27 (3.83-8.65)	0.94 (0.57-1.23)
**Sudan**	6.9 (3.43-10.85)	3.33 (1.19-4.82)	2.24 (0.99-4.33)	5.91 (2.94-9.24)	2.85 (1.03-4.2)	2.24 (0.99-4.33)	5.22 (2.57-8.24)	3.25 (1.17-4.72)	1.74 (0.75-3.45)	4.47 (2.2-7.09)	2.78 (1.01-4.09)	1.74 (0.75-3.45)
**Syrian Arab Republic**	0.76 (0.57-0.99)	0.61 (0.46-0.91)	1.25 (0.86-1.5)	0.69 (0.51-0.9)	0.53 (0.39-0.79)	1.3 (0.91-1.57)	0.56 (0.42-0.73)	0.59 (0.44-0.89)	0.96 (0.66-1.17)	0.46 (0.34-0.61)	0.51 (0.38-0.76)	0.92 (0.64-1.11)
**Tunisia**	1.27 (0.81-1.84)	0.68 (0.45-1.33)	1.96 (0.86-2.77)	1.15 (0.73-1.69)	0.6 (0.39-1.17)	2.04 (0.88-2.89)	0.92 (0.6-1.33)	0.68 (0.45-1.32)	1.41 (0.61-1.99)	0.67 (0.42-0.98)	0.56 (0.37-1.09)	1.26 (0.56-1.77)
**Turkey**	1.22 (0.96-1.5)	0.59 (0.46-0.75)	2.06 (1.68-2.42)	1.21 (0.94-1.52)	0.55 (0.43-0.71)	2.19 (1.78-2.55)	0.95 (0.74-1.18)	0.58 (0.45-0.74)	1.65 (1.34-1.94)	0.75 (0.58-0.95)	0.49 (0.38-0.62)	1.54 (1.26-1.84)
**United Arab Emirates**	2.51 (0.77-3.77)	1.46 (0.35-2.39)	2.2 (0.44-6.49)	2.14 (0.68-3.25)	1.15 (0.27-1.89)	2.37 (0.47-6.93)	2.2 (0.67-3.31)	1.43 (0.34-2.32)	1.96 (0.39-5.77)	1.55 (0.48-2.36)	1.08 (0.25-1.77)	1.83 (0.37-5.44)
**Yemen**	8.05 (5.64-11.68)	5.51 (2.4-8.53)	1.56 (0.91-3.15)	7.54 (5.24-11.1)	4.97 (2.15-7.68)	1.62 (0.95-3.27)	5.84 (4.09-8.7)	5.04 (2.18-7.82)	1.23 (0.72-2.52)	5.03 (3.48-7.45)	4.48 (1.94-6.99)	1.2 (0.7-2.43)
**South Asia**	36.97 (30.47-44.22)	25.15 (20.28-32.26)	1.49 (1.09-1.96)	40.11 (32.97-47.31)	22.73 (18.32-29.44)	1.79 (1.32-2.31)	35.81 (29.99-42.25)	24.53 (19.85-31.52)	1.48 (1.09-1.93)	24.76 (20.08-30.19)	21.35 (17.2-27.66)	1.18 (0.86-1.54)
**South Asia**	36.97 (30.47-44.22)	25.15 (20.28-32.26)	1.49 (1.09-1.96)	40.11 (32.97-47.31)	22.73 (18.32-29.44)	1.79 (1.32-2.31)	35.81 (29.99-42.25)	24.53 (19.85-31.52)	1.48 (1.09-1.93)	24.76 (20.08-30.19)	21.35 (17.2-27.66)	1.18 (0.86-1.54)
**Bangladesh**	27.91 (22.13-34.54)	15.75 (8.93-29.21)	1.93 (0.95-3.14)	26.05 (20.8-32.37)	14.34 (7.99-26.48)	1.98 (0.97-3.19)	21.14 (16.9-26.44)	15.46 (8.77-28.52)	1.49 (0.72-2.43)	18.54 (14.67-23.63)	13.9 (7.75-25.57)	1.45 (0.7-2.37)

**Bhutan**	16.55 (9.82-30.84)	18.94 (9.01-42.75)	1.01 (0.41-2.34)	16.6 (9.98-30.66)	17.15 (8.02-39.09)	1.12 (0.45-2.57)	16.01 (9.52-28.49)	18.43 (8.74-41.73)	1 (0.41-2.26)	12.76 (7.56-23.06)	16.38 (7.65-37.41)	0.9 (0.37-2.09)
**India**	36.31 (28.71-45.01)	24.77 (18.91-32.16)	1.49 (1.03-2.08)	40.63 (32.19-49.23)	22.35 (16.97-29.27)	1.85 (1.31-2.55)	36.62 (29.31-44.44)	24.17 (18.47-31.77)	1.54 (1.09-2.12)	24.19 (18.78-30.52)	20.89 (15.86-27.48)	1.18 (0.81-1.64)
**Nepal**	38.33 (28.24-49.93)	19.82 (13.48-27.51)	1.98 (1.31-2.93)	42.86 (32.66-53.05)	18.67 (12.78-25.78)	2.35 (1.6-3.51)	37.4 (28.62-46.57)	18.07 (12.4-24.69)	2.12 (1.45-3.15)	25.72 (18.56-33.27)	15.36 (10.62-21.23)	1.71 (1.15-2.52)
**Pakistan**	51.23 (35.15-75.51)	36.72 (28.98-48.53)	1.42 (0.89-2.24)	48.59 (33.36-71.5)	32.91 (25.79-43.5)	1.51 (0.92-2.35)	42.51 (29.65-62.37)	35.81 (28.24-47.23)	1.21 (0.75-1.89)	34.82 (24.01-51.45)	31.75 (24.87-41.95)	1.12 (0.7-1.76)
**Southeast Asia, East Asia, and Oceania**	8.99 (7.79-10.35)	5.06 (4.46-5.8)	1.78 (1.48-2.11)	10.69 (9.56-11.88)	4.81 (4.22-5.57)	2.23 (1.89-2.56)	8.08 (7.13-9.1)	5.09 (4.51-5.87)	1.59 (1.33-1.84)	5.64 (4.79-6.66)	4.41 (3.83-5.09)	1.28 (1.06-1.52)
**East Asia**	2.18 (1.74-2.71)	1.08 (0.89-1.34)	2.04 (1.48-2.62)	3.07 (2.53-3.64)	1.05 (0.87-1.31)	2.95 (2.21-3.72)	2.12 (1.75-2.52)	1.1 (0.91-1.37)	1.95 (1.44-2.48)	1.26 (1-1.57)	0.94 (0.77-1.17)	1.35 (0.98-1.74)
**China**	2 (1.57-2.51)	0.98 (0.79-1.29)	2.07 (1.4-2.72)	2.84 (2.31-3.43)	0.96 (0.77-1.26)	3 (2.1-3.92)	1.93 (1.56-2.32)	1 (0.8-1.33)	1.95 (1.34-2.57)	1.14 (0.89-1.45)	0.85 (0.68-1.11)	1.35 (0.92-1.77)
**Democratic People's Republic of Korea**	11.02 (7.99-14.71)	6.15 (2.82-8.52)	1.89 (1.21-3.83)	14.85 (11.01-19.38)	5.87 (2.63-8.32)	2.67 (1.76-5.64)	10.58 (7.93-13.96)	6.1 (2.85-8.55)	1.83 (1.19-3.86)	6.66 (4.75-8.77)	5.36 (2.42-7.57)	1.31 (0.83-2.67)
**Taiwan (Province of China)**	2.55 (1.88-3.32)	1.06 (0.78-1.39)	2.44 (1.93-3.19)	3.24 (2.51-4.05)	0.98 (0.71-1.3)	3.34 (2.76-4.14)	2.76 (2.18-3.47)	1.09 (0.81-1.43)	2.56 (2.09-3.19)	1.71 (1.24-2.25)	0.92 (0.66-1.23)	1.86 (1.48-2.45)
**Oceania**	23.52 (15.96-32.16)	15.53 (8.71-23.34)	1.57 (0.97-2.5)	22.31 (15.3-30.73)	13.32 (7.39-20.3)	1.74 (1.07-2.74)	21.75 (14.93-30.04)	13.99 (8.02-21.2)	1.62 (1.02-2.54)	15.49 (10.52-21.61)	11.83 (6.72-17.98)	1.36 (0.86-2.19)
**Fiji**	5.41 (4.08-6.91)	4.24 (3.21-5.55)	1.28 (1.01-1.63)	5.05 (3.82-6.64)	3.32 (2.49-4.44)	1.53 (1.23-1.87)	5.37 (4.21-6.74)	4.06 (3.07-5.31)	1.33 (1.08-1.63)	3.1 (2.26-4.07)	3.06 (2.27-4.07)	1.02 (0.8-1.28)
**Kiribati**	96.18 (70.2-128.96)	74.72 (54.57-104.45)	1.31 (0.9-1.79)	83.07 (61.3-112.99)	61.42 (44.15-87.53)	1.38 (0.96-1.87)	65.01 (49.49-85.92)	57.27 (42.33-79.89)	1.16 (0.81-1.56)	44.35 (31.52-60.75)	47.14 (33.83-66.65)	0.96 (0.66-1.3)
**Marshall Islands**	18.1 (13.02-24.22)	16.81 (5.71-24.77)	1.21 (0.73-3.18)	16.39 (12.1-21.8)	12.48 (4.18-18.15)	1.47 (0.93-3.85)	17.86 (13.39-23.36)	16.02 (5.41-23.45)	1.25 (0.78-3.3)	10.44 (7.37-13.95)	11.54 (3.86-16.83)	1.01 (0.61-2.64)
**Micronesia (Federated States of)**	15.41 (10.75-20.45)	12.28 (6.46-17.7)	1.31 (0.89-2.43)	15.19 (10.75-20.27)	9.9 (5.2-14.46)	1.6 (1.1-3)	13.5 (9.48-17.64)	10.47 (5.45-15.11)	1.34 (0.92-2.48)	8.75 (6.2-11.62)	8.39 (4.42-12.07)	1.09 (0.74-2.04)
**Nauru**	9.4 (6.8-12.35)	7.68 (5.13-10.76)	1.26 (0.81-1.95)	11.25 (8.35-14.55)	6.63 (4.38-9.39)	1.75 (1.16-2.71)	10.59 (7.98-13.55)	6.67 (4.43-9.36)	1.63 (1.09-2.53)	5.87 (4.22-7.85)	5.36 (3.57-7.6)	1.13 (0.74-1.75)
**Palau**	3.5 (2.54-4.64)	3.55 (2.63-4.58)	0.99 (0.76-1.26)	3.4 (2.6-4.31)	2.85 (2.08-3.71)	1.2 (0.98-1.46)	3.43 (2.67-4.34)	3.38 (2.5-4.33)	1.02 (0.83-1.25)	2.15 (1.54-2.86)	2.63 (1.91-3.44)	0.82 (0.63-1.06)

**Papua New Guinea**	28.16 (17.92-39.78)	18.45 (9.24-29.53)	1.63 (0.91-2.83)	26.71 (17.14-37.71)	15.94 (8.16-25.66)	1.79 (1.01-3.05)	26.38 (16.84-37.83)	16.69 (8.46-27.07)	1.69 (0.95-2.84)	19.01 (12.21-27.25)	14.22 (7.34-22.86)	1.42 (0.82-2.46)
**Samoa**	7.41 (4.99-10.36)	7.38 (4.95-11.84)	1.05 (0.57-1.62)	7.85 (5.43-11.58)	6.08 (3.89-9.85)	1.35 (0.73-2.02)	6.2 (4.34-8.69)	6.54 (4.39-10.45)	0.99 (0.54-1.5)	3.89 (2.58-5.64)	5.18 (3.35-8.4)	0.78 (0.43-1.22)
**Solomon Islands**	13.52 (10.77-16.72)	8.28 (6.55-10.38)	1.64 (1.33-1.99)	12.43 (9.83-15.31)	7.08 (5.52-8.89)	1.76 (1.46-2.13)	10.85 (8.74-13.23)	7.39 (5.84-9.27)	1.48 (1.22-1.77)	7.72 (6-9.83)	6.22 (4.91-7.85)	1.25 (1.01-1.52)
**Tonga**	6.76 (5.15-8.61)	3.84 (2.71-5.1)	1.79 (1.28-2.42)	6.34 (4.91-7.95)	3.18 (2.2-4.33)	2.03 (1.52-2.72)	5.25 (4.07-6.52)	3.54 (2.51-4.74)	1.51 (1.11-2.02)	3.7 (2.73-4.77)	2.87 (1.99-3.91)	1.31 (0.95-1.77)
**Tuvalu**	10.36 (7.66-13.68)	8.68 (5.19-12.27)	1.23 (0.89-2.04)	10.11 (7.55-13.41)	7.07 (4.24-10.01)	1.47 (1.1-2.39)	9.14 (6.94-11.98)	7.79 (4.62-10.96)	1.2 (0.9-1.97)	6.13 (4.46-8.35)	6.21 (3.66-8.87)	1.01 (0.73-1.7)
**Vanuatu**	23.61 (16.92-31.52)	15.29 (8.17-22.54)	1.64 (1.08-3.02)	22.73 (16.36-30.09)	12.72 (6.77-18.76)	1.9 (1.26-3.42)	22.04 (15.81-28.9)	14.96 (7.97-21.95)	1.56 (1.04-2.84)	15.33 (10.71-20.66)	12.31 (6.53-18.08)	1.32 (0.86-2.49)
**Southeast Asia**	30.17 (25.77-35.15)	17.14 (14.87-19.85)	1.77 (1.44-2.14)	34.27 (30.06-38.83)	16.09 (13.89-18.73)	2.14 (1.76-2.5)	26.22 (22.88-30.05)	17.15 (14.92-19.94)	1.54 (1.26-1.81)	18.76 (15.82-22.35)	14.79 (12.72-17.27)	1.27 (1.03-1.54)
**Cambodia**	44.08 (29.49-60.67)	31.25 (18.86-51)	1.51 (0.76-2.41)	63.23 (44.42-83.15)	30.85 (18.81-51.46)	2.21 (1.09-3.42)	42.67 (29.35-56.26)	32.81 (20.27-54.24)	1.4 (0.69-2.22)	22.76 (15.17-31.96)	25.93 (15.52-43.47)	0.94 (0.47-1.49)
**Indonesia**	50.28 (41.12-59.88)	26.56 (21.72-32.03)	1.91 (1.43-2.46)	47.1 (38.58-56.3)	24.3 (19.82-29.61)	1.96 (1.47-2.5)	35.26 (28.99-42.9)	25.69 (21.12-31.26)	1.39 (1.01-1.76)	31.06 (25.06-38.18)	23.44 (19.1-28.68)	1.34 (0.99-1.72)
**Lao People's Democratic Republic**	41.2 (28.96-56.62)	27.04 (16.2-37.9)	1.56 (1.03-2.46)	52.5 (38.64-67.21)	27.3 (17.33-37.95)	1.98 (1.35-3.05)	39.98 (29.14-52.1)	29.9 (18.43-41.51)	1.37 (0.95-2.13)	23.98 (16.5-33.1)	22.26 (13.62-31.37)	1.11 (0.74-1.73)
**Malaysia**	9.64 (7.53-12.09)	5.02 (3.95-6.55)	1.93 (1.54-2.36)	9.34 (7.17-11.93)	4.38 (3.37-5.74)	2.15 (1.71-2.6)	8.01 (6.26-10.06)	4.99 (3.93-6.51)	1.62 (1.28-1.95)	6.06 (4.6-7.85)	4.18 (3.21-5.46)	1.46 (1.15-1.77)
**Maldives**	6.28 (4.9-7.82)	3.03 (2.39-3.97)	2.09 (1.56-2.62)	5.95 (4.63-7.41)	2.69 (2.09-3.51)	2.23 (1.67-2.76)	4.74 (3.8-5.95)	2.93 (2.3-3.81)	1.63 (1.24-2.05)	3.75 (2.86-4.81)	2.57 (1.99-3.35)	1.48 (1.11-1.85)
**Mauritius**	0.97 (0.71-1.28)	0.36 (0.27-0.48)	2.74 (1.99-3.57)	1.15 (0.86-1.49)	0.31 (0.22-0.42)	3.81 (2.94-4.62)	1.04 (0.8-1.35)	0.37 (0.27-0.49)	2.88 (2.2-3.55)	0.55 (0.39-0.76)	0.28 (0.2-0.39)	1.98 (1.44-2.57)
**Myanmar**	34.57 (25.47-45.45)	19.12 (12.54-26.14)	1.86 (1.25-2.95)	40.94 (31.69-51.97)	17.16 (11.35-23.74)	2.45 (1.71-3.72)	34.63 (26.14-43.86)	18.29 (12.01-25.37)	1.94 (1.34-2.96)	22.87 (16.93-30.35)	15.49 (10.22-21.42)	1.52 (1.02-2.4)
**Philippines**	33.94 (25.24-44.08)	17.74 (13.48-22.6)	1.94 (1.32-2.77)	48.89 (37.47-62.62)	18.42 (13.95-23.66)	2.7 (1.85-3.75)	36.86 (28.55-47.01)	19.05 (14.41-24.09)	1.97 (1.35-2.76)	21.46 (15.96-28.17)	14.81 (11.18-18.99)	1.47 (1-2.08)
**Seychelles**	3.64 (2.78-4.69)	1.97 (1.58-2.51)	1.87 (1.36-2.48)	4.61 (3.68-5.7)	1.7 (1.33-2.16)	2.74 (2.08-3.45)	4.04 (3.36-4.87)	2.04 (1.67-2.55)	2 (1.5-2.54)	1.99 (1.44-2.66)	1.51 (1.19-1.95)	1.33 (0.96-1.77)

**Sri Lanka**	3.72 (2.66-5.14)	1.87 (1.34-2.6)	2 (1.53-2.52)	4.6 (3.36-6.27)	1.62 (1.15-2.27)	2.87 (2.24-3.54)	4.67 (3.39-6.22)	1.96 (1.41-2.74)	2.4 (1.83-2.99)	2.43 (1.7-3.43)	1.49 (1.06-2.11)	1.64 (1.25-2.08)
**Thailand**	6.74 (4.83-9.15)	4.99 (3.71-6.62)	1.36 (1.08-1.68)	9.79 (7.24-12.98)	4.81 (3.55-6.35)	2.05 (1.69-2.43)	8.2 (6.04-10.69)	5.28 (3.95-6.96)	1.56 (1.28-1.87)	4.38 (3.09-6.01)	4.26 (3.12-5.66)	1.03 (0.81-1.3)
**Timor-Leste**	34.67 (20.83-52.27)	26.29 (15.22-38.72)	1.37 (0.76-2.3)	40.84 (25.09-59.28)	24.2 (14.21-35.46)	1.75 (1-2.92)	32.31 (20.15-47.2)	26.31 (15.27-38.87)	1.28 (0.74-2.09)	21.44 (13.03-31.97)	22.69 (13.29-33.43)	0.98 (0.55-1.65)
**Viet Nam**	19.9 (14.97-25.94)	13.45 (10.29-17.51)	1.5 (1.07-1.99)	31.59 (25.74-37.98)	12.44 (9.4-16.34)	2.57 (1.95-3.2)	24.4 (19.93-29.53)	13.8 (10.5-18)	1.79 (1.36-2.26)	11.93 (8.75-15.9)	11.59 (8.78-15.33)	1.04 (0.76-1.4)
**Sub-Saharan Africa**	69.8 (59.39-81.83)	42.41 (35.31-54.04)	1.66 (1.29-1.92)	85.96 (74.93-97.75)	43.86 (36.93-56.03)	1.97 (1.53-2.27)	81.86 (71.45-92.34)	46.44 (39.01-58.99)	1.78 (1.38-2.04)	55.55 (46.83-65.76)	38.27 (31.77-49.27)	1.46 (1.13-1.69)
**Central Sub-Saharan Africa**	104.59 (77.08-136.1)	66.45 (45.37-99.29)	1.62 (1.08-2.15)	119.03 (91.11-153.01)	68.73 (47.75-102.54)	1.79 (1.19-2.32)	118.89 (91.41-151.14)	74.26 (50.96-110.15)	1.65 (1.08-2.18)	81.11 (59.06-104.66)	59.97 (40.69-90.5)	1.4 (0.92-1.85)
**Angola**	70.01 (52.94-90.61)	38.61 (27.37-53.97)	1.84 (1.32-2.45)	100.93 (81.28-124.24)	46.78 (33.4-66.5)	2.2 (1.61-2.87)	93.65 (76.34-114.16)	49.65 (35.39-71.47)	1.92 (1.41-2.51)	50.95 (37.89-66.56)	34.66 (24.47-48.05)	1.49 (1.08-2.01)
**Central African Republic**	319.95 (239.73-411.98)	170.51 (106.11-249.33)	1.95 (1.37-2.6)	355.64 (276-452.77)	173.22 (109.57-250.76)	2.13 (1.52-2.79)	356.37 (277.37-451.22)	187.29 (117.03-270.98)	1.97 (1.39-2.62)	249.2 (186.68-322.7)	153.65 (97.38-223.26)	1.68 (1.17-2.25)
**Congo**	55.22 (37.56-74.56)	38.79 (19.85-57.99)	1.51 (0.92-2.88)	70.77 (49.96-89.69)	44.14 (22.43-63.16)	1.71 (1.05-3.18)	67.94 (47.86-85.97)	48.51 (24.61-69.68)	1.49 (0.9-2.79)	40.69 (27.14-54.63)	34.47 (17.99-51.33)	1.25 (0.75-2.39)
**Democratic Republic of the Congo**	110.27 (75.9-151.83)	72.86 (46-117.3)	1.59 (0.94-2.29)	118.27 (84.5-161.17)	73.04 (46.51-118.05)	1.7 (1-2.41)	120.6 (86.73-162.56)	79.13 (50.73-126.98)	1.6 (0.95-2.28)	87.22 (60.35-119.01)	65.9 (40.93-107.07)	1.39 (0.83-2.01)
**Equatorial Guinea**	27.54 (17.09-38.07)	18.32 (8.72-41.46)	1.68 (0.67-3.11)	38.31 (25.49-51.51)	21.57 (10.05-48.21)	1.98 (0.79-3.6)	37.72 (24.75-50.34)	23.68 (11.07-52.13)	1.78 (0.72-3.22)	20.6 (12.46-28.63)	16.3 (7.63-36.55)	1.41 (0.57-2.62)
**Gabon**	39.25 (25.04-52.16)	15.74 (7.93-23.83)	2.68 (1.58-5.03)	56.81 (37.55-72.63)	19.25 (9.73-28.41)	3.17 (1.92-5.85)	57.86 (38.16-72.86)	21.18 (10.64-31.12)	2.94 (1.79-5.43)	29.21 (18.28-39.12)	13.91 (7.1-20.86)	2.25 (1.32-4.23)
**Eastern Sub-Saharan Africa**	86.35 (70.02-103.71)	53.51 (43.21-70.5)	1.63 (1.2-2)	105.87 (86.91	55.52 (45.13-73.77)	1.93 (1.39-2.36)	99.46 (82.08-	57.7 (47.41-75.76)	1.75 (1.25-2.14)	69.17 (55.82-84.53)	48.62 (39.06-65.39)	1.44 (1.06-1.77)

				-125.68)			116.42)					
**Burundi**	116.83 (73.33-169.95)	103.15 (65.38-176.26)	1.2 (0.62-1.85)	178.41 (121.08-251.42)	108.06 (69.75-182.89)	1.75 (0.92-2.63)	170.38 (115.01-238.54)	113.13 (72.94-192.11)	1.6 (0.83-2.4)	95.96 (60.01-138.16)	94.65 (59.94-162.45)	1.07 (0.56-1.66)
**Comoros**	76.29 (56.15-98.28)	50.01 (32.75-71.75)	1.58 (0.98-2.45)	74.45 (54.66-94.73)	48.25 (31.58-68.98)	1.59 (0.98-2.48)	67.24 (49.69-86.34)	50.14 (32.67-72.1)	1.38 (0.86-2.16)	59.07 (43.34-75.89)	46.15 (29.85-66.27)	1.32 (0.82-2.05)
**Djibouti**	80.65 (58.34-107.99)	40 (22.65-60.97)	2.1 (1.3-3.54)	77.45 (56.86-104.82)	37.64 (21.39-57.06)	2.14 (1.37-3.66)	63.89 (46.5-85.56)	39.16 (22.21-59.27)	1.7 (1.07-2.85)	56.3 (40.36-75.64)	36.01 (20.59-54.5)	1.63 (1-2.72)
**Eritrea**	179.36 (121.71-243.57)	87.25 (54.25-137.98)	2.14 (1.28-3.45)	192.94 (132.39-263.28)	84.82 (53.06-133.05)	2.37 (1.42-3.83)	186.21 (126.85-252.3)	91.08 (56.85-144.13)	2.13 (1.28-3.43)	145.76 (100.37-198.92)	80.58 (50.47-125.06)	1.89 (1.12-3.06)
**Ethiopia**	59.4 (45.18-73.88)	40.79 (31.46-54.86)	1.48 (1.06-1.94)	71.68 (57.3-87.61)	42.31 (33.06-55.93)	1.72 (1.22-2.27)	71.21 (57.01-86.98)	44.7 (35.33-59)	1.62 (1.14-2.14)	51.98 (39.44-65.47)	38.22 (29.44-51.47)	1.38 (0.98-1.81)
**Kenya**	64.58 (43.75-88.41)	37.96 (25.83-55.38)	1.75 (0.97-2.62)	85.63 (60.13-113.83)	39.34 (26.36-57.67)	2.25 (1.27-3.34)	80.57 (57.09-106.36)	40.9 (27.43-59.7)	2.03 (1.14-2.99)	51.72 (35.33-71.72)	35.21 (23.9-51.67)	1.51 (0.84-2.26)
**Madagascar**	94.13 (69.68-126.14)	67.95 (42.19-103.09)	1.44 (0.9-2.11)	105.56 (79.16-141.88)	67.43 (42.14-102.47)	1.62 (1.03-2.4)	100.7 (75.89-134.04)	70.96 (44.09-107.59)	1.47 (0.93-2.19)	77.48 (57.37-103.66)	62.72 (38.48-95.83)	1.28 (0.8-1.89)
**Malawi**	112.1 (85.12-145.16)	49.05 (34.4-74)	2.35 (1.48-3.16)	129.55 (100.2-166.97)	49.99 (35.01-74.61)	2.67 (1.68-3.54)	116.86 (90-150.44)	52.83 (37.34-78.76)	2.28 (1.44-3.04)	81.91 (60.96-108.97)	44.39 (30.95-65.46)	1.9 (1.2-2.56)
**Mozambique**	195.57 (150.26-256.03)	83.28 (57.55-123.87)	2.41 (1.57-3.22)	213 (165.42-279.93)	82.58 (56.46-122.69)	2.65 (1.74-3.48)	200.17 (156.56-259.55)	86.05 (59.84-128.5)	2.39 (1.56-3.16)	151.62 (114.97-199.65)	75.22 (51.44-111.79)	2.07 (1.35-2.74)
**Rwanda**	46.45 (31.54-65.37)	32.86 (22.93-50.83)	1.45 (0.87-2.07)	74.51 (53.75-98.08)	38.48 (27.52-58.17)	2 (1.21-2.84)	63.79 (45.9-84.32)	36.08 (25.67-54.87)	1.82 (1.09-2.61)	34.19 (23.19-48.59)	27.06 (18.78-41.66)	1.3 (0.78-1.82)

**Somalia**	290.84 (218.03-453.54)	177.82 (106.09-341.53)	1.78 (0.86-3.15)	266.91 (202.14-414.34)	165.62 (97.86-317.29)	1.75 (0.84-3.11)	247.96 (186.32-381.05)	173.97 (103.68-334.19)	1.55 (0.74-2.74)	227.78 (169.56-357.33)	162.07 (95.82-311.79)	1.53 (0.73-2.7)
**South Sudan**	118.53 (84.46-158.46)	62.74 (34.19-89.21)	1.98 (1.34-3.22)	113.76 (80.91-152.81)	59.29 (32.31-84.01)	2.01 (1.38-3.25)	106.09 (75.85-142.41)	62.08 (33.63-88.39)	1.79 (1.21-2.95)	94.26 (66.97-127.05)	57.51 (31.18-81.61)	1.72 (1.16-2.81)
**Uganda**	68.66 (47.15-95.13)	37.5 (25.51-58.67)	1.91 (1.01-2.85)	108.73 (74.2-143.04)	44.86 (31.52-68.64)	2.52 (1.37-3.71)	108.12 (74.09-140.43)	47.15 (33.29-72.36)	2.39 (1.3-3.49)	55.58 (36.99-77.78)	33.24 (22.58-51.92)	1.74 (0.92-2.6)
**United Republic of Tanzania**	56.22 (39.21-75.44)	40.21 (29-63.83)	1.43 (0.77-1.9)	84.81 (60.36-110.04)	46.08 (33.74-75.44)	1.89 (1.01-2.49)	72.73 (51.74-93.82)	45.96 (34.14-74.78)	1.62 (0.87-2.15)	42.38 (30.12-57.64)	35.4 (25.22-57.29)	1.23 (0.66-1.63)
**Zambia**	81.1 (54.55-111.61)	41.19 (26.88-62.97)	2.04 (1.09-3.08)	106.99 (71.66-143.12)	45.16 (29.98-67.6)	2.46 (1.29-3.64)	100.05 (66.93-132.74)	45.85 (30.57-69.35)	2.26 (1.2-3.41)	63.35 (43.03-87)	36.19 (23.92-55.34)	1.82 (0.97-2.76)
**Southern Sub-Saharan Africa**	57.31 (48.03-67.98)	25.48 (21.8-29.69)	2.26 (1.91-2.63)	78.83 (69.83-87.81)	26.01 (22.33-30.29)	3.04 (2.65-3.48)	68.35 (61.1-76.07)	27.81 (24.11-32.29)	2.47 (2.14-2.83)	40.33 (33.08-48.54)	21.71 (18.29-25.66)	1.86 (1.58-2.2)
**Botswana**	46.65 (33.16-64.73)	24.67 (14.86-44.9)	2.01 (1.03-3.01)	64.74 (47.51-87.36)	24.47 (14.77-44.26)	2.81 (1.48-4.08)	54.44 (40.69-72.63)	25.75 (15.63-46.88)	2.25 (1.19-3.27)	31.47 (22.02-44.03)	20.74 (12.64-36.62)	1.61 (0.84-2.39)
**Eswatini**	101.16 (72.8-136.29)	30.87 (17.91-47.5)	3.4 (2.33-5.09)	137.06 (101.89-178.86)	31.46 (18.42-48.66)	4.52 (3.22-6.6)	138.73 (104.72-180)	34.17 (19.89-52.83)	4.22 (2.99-6.2)	81.45 (58.84-110.54)	26.88 (15.6-41.44)	3.14 (2.19-4.68)
**Lesotho**	192.47 (143.99-250.43)	73.86 (45.83-106.93)	2.67 (1.97-3.69)	255.92 (200.74-316.14)	75.3 (47.64-108.23)	3.5 (2.6-4.77)	201.48 (156.18-252.09)	80.78 (50.86-115.7)	2.57 (1.87-3.55)	126.28 (93.6-164.35)	64.25 (40.33-92.75)	2.02 (1.47-2.81)
**Namibia**	51 (37.12-67.78)	20.18 (13.48-30.53)	2.59 (1.74-3.31)	75.34 (57.49-95.79)	22.59 (15.27-34.57)	3.42 (2.35-4.39)	71.08 (54.43-89.24)	22.91 (15.74-34.83)	3.19 (2.19-4.07)	39.44 (28.1-53.88)	16.78 (11.19-25.36)	2.41 (1.64-3.08)
**South Africa**	38.5 (32.08-46.61)	18.78 (15.97-22.37)	2.06 (1.69-2.45)	56.41 (50.49	19.76 (16.74-23.38)	2.87 (2.4-3.34)	50.43 (45.5-56)	21.05 (18.1-24.79)	2.41 (1.99-2.81)	27.72 (22.7-33.99)	15.92 (13.34-19.47)	1.75 (1.44-2.09)

				-62.74)								
**Zimbabwe**	156.78 (121.47-198.53)	54.21 (32-75.37)	3.01 (2.1-5.12)	189.77 (148.38-234.14)	51.33 (30.53-71.43)	3.84 (2.71-6.53)	156.34 (122.34-193.41)	55.68 (32.47-76.74)	2.92 (2.05-4.92)	103.68 (77.44-133.81)	45.65 (27.12-64.21)	2.36 (1.63-4.06)
**Western Sub-Saharan Africa**	49.9 (40.21-60.35)	32.35 (22.63-48.14)	1.58 (1.07-2.27)	62.18 (51.63-73.94)	33.43 (23.3-48.83)	1.91 (1.28-2.68)	60.79 (50.31-72.26)	35.82 (24.7-52.59)	1.74 (1.16-2.47)	41.48 (33.67-50.44)	29.36 (20.63-43.11)	1.45 (0.97-2.06)
**Benin**	47.96 (33.85-62.51)	28.02 (15.49-45.09)	1.8 (1.07-3.06)	54.43 (39.52-71.06)	27.78 (15.67-44.85)	2.06 (1.23-3.47)	53.77 (39.09-70.36)	30.32 (16.75-48.51)	1.87 (1.11-3.15)	39.02 (27.86-51.06)	24.95 (14.08-40.52)	1.65 (0.97-2.79)
**Burkina Faso**	54.98 (40.97-71.58)	35.43 (25.76-46.78)	1.57 (1.17-2.1)	77.57 (61.52-95.15)	43.6 (32.74-55.71)	1.8 (1.36-2.36)	77.04 (62.05-94.34)	47.34 (35.39-60.38)	1.65 (1.24-2.15)	46.04 (34.42-61.41)	32.29 (23.49-42.96)	1.44 (1.07-1.93)
**Cabo Verde**	10.21 (7.68-13.24)	9.64 (6.39-17.97)	1.12 (0.54-1.66)	13.26 (10.43-16.38)	9.97 (6.64-18.93)	1.41 (0.67-2.05)	13.46 (10.84-16.57)	11.24 (7.47-21.52)	1.27 (0.61-1.85)	8.21 (6.08-10.8)	8.23 (5.48-15.64)	1.06 (0.51-1.56)
**Cameroon**	41.31 (25.26-57.52)	26.68 (11.51-41.07)	1.68 (0.84-3.37)	56.29 (36.69-76.68)	30.01 (12.77-46.37)	2.03 (1.04-4.09)	54.41 (34.94-72.67)	32.46 (13.78-49.35)	1.82 (0.93-3.63)	33.3 (20.49-46.01)	24.09 (10.56-37.19)	1.5 (0.74-3.02)
**Chad**	89.05 (65.24-117.54)	62.45 (45.47-82.77)	1.45 (1.03-1.97)	99.88 (77.3-129.35)	65 (48.26-85.14)	1.56 (1.12-2.12)	96.65 (74.7-124.61)	70.02 (51.74-91.98)	1.4 (1-1.91)	72.73 (52.85-95.83)	55.78 (41.02-73.93)	1.32 (0.94-1.79)
**Côte d'Ivoire**	49.37 (33.18-66.25)	30.69 (13.51-48.73)	1.75 (0.98-3.46)	66.77 (47.94-86.37)	32.59 (14.58-50.67)	2.22 (1.28-4.45)	62.59 (45.01-80.37)	34.97 (15.45-54.51)	1.95 (1.11-3.9)	38.8 (26.37-52.33)	26.83 (12.21-42.24)	1.57 (0.87-3.1)
**Gambia**	73.65 (54.1-95.7)	41.35 (23.68-59.62)	1.87 (1.21-3.22)	86.88 (65.22-110.27)	41.64 (23.73-59.95)	2.19 (1.44-3.7)	79.57 (60.16-100.87)	45.49 (25.78-65.82)	1.84 (1.21-3.11)	56.66 (41.54-72.85)	37.11 (21.4-52.97)	1.6 (1.04-2.74)
**Ghana**	69.14 (49.98-88.9)	21.34 (14.58-31.54)	3.32 (2.18-4.88)	93.28 (72.74-115.03)	21.98 (15.1-31.89)	4.35 (2.93-6.26)	94.58 (73.65-115.85)	23.95 (16.16-35.43)	4.05 (2.7-5.77)	55.76 (40.34-72.87)	18.85 (13.09-27.82)	3.03 (1.98-4.46)
**Guinea**	76.92 (55.93-102.51)	47.47 (29.31-70.53)	1.68 (1.11-2.64)	80.3 (58.48-107.12)	45.01 (27.75-66.88)	1.85 (1.22-2.89)	70.85 (51.53-93.84)	48.21 (29.49-71.16)	1.53 (1.01-2.35)	57.69 (41.49-77.12)	42.63 (26.49-64)	1.4 (0.93-2.18)
**Guinea-Bissau**	87.55 (64.31-111.94)	48.06 (29.21-78.62)	1.9 (1.14-2.94)	106.38 (81.27-	48.79 (30.07-81.38)	2.28 (1.36-3.43)	106.95 (82.21-133.59)	53.4 (32.55-87.99)	2.09 (1.25-3.17)	73.17 (53.18-92.45)	43.03 (26.49-71.06)	1.77 (1.07-2.75)

				133.47)								
**Liberia**	39.66 (28.76-52.92)	32.12 (21.68-61.77)	1.3 (0.66-1.8)	48.18 (36.67-63.4)	33.07 (22.22-63.19)	1.53 (0.78-2.06)	49.01 (37.37-63.33)	36.64 (24.77-69.42)	1.41 (0.71-1.89)	31.88 (23.18-42.61)	28.07 (18.87-53.62)	1.19 (0.6-1.65)
**Mali**	57.75 (44.63-74.89)	41.32 (28.15-71.47)	1.45 (0.81-2.05)	57.84 (44.51-74.01)	40.55 (27.77-70.69)	1.48 (0.84-2.11)	53.85 (41.64-69.66)	43.88 (29.96-75.9)	1.28 (0.72-1.79)	45.67 (34.94-59.42)	37.02 (25.31-64.46)	1.28 (0.73-1.79)
**Mauritania**	24.46 (15.35-35.22)	21.35 (8.92-36.34)	1.29 (0.63-2.91)	23.02 (14.22-33.14)	19.65 (8.29-33.14)	1.32 (0.65-2.96)	21.5 (13.5-30.9)	20.88 (8.7-35.29)	1.16 (0.57-2.65)	20.21 (12.67-29.1)	19.21 (8.13-32.21)	1.19 (0.58-2.69)
**Niger**	71.86 (52.52-99.22)	43.69 (31.65-59.83)	1.68 (1.17-2.45)	70.52 (51.65-97)	41.36 (30.02-56.62)	1.74 (1.23-2.52)	68.37 (50.31-93.95)	43.97 (32.06-60.45)	1.59 (1.1-2.31)	61.7 (45.06-85)	40.45 (29.46-55.53)	1.56 (1.08-2.25)
**Nigeria**	40.31 (29-54.76)	29.77 (20.09-53.44)	1.42 (0.78-2.25)	53.69 (39.77-73.45)	30.97 (20.68-54.8)	1.82 (0.99-2.91)	53.6 (39.74-72.32)	32.82 (21.96-59.25)	1.72 (0.93-2.77)	35.1 (25.28-48.14)	27.48 (18.48-49.87)	1.34 (0.73-2.1)
**Sao Tome and Principe**	16.01 (11.82-20.58)	10.12 (6.55-18.82)	1.69 (0.81-2.49)	21.46 (16.5-26.54)	11.19 (7.21-21.09)	2.04 (0.99-3.02)	21.91 (17.13-27.11)	12.34 (7.92-23.02)	1.89 (0.91-2.79)	13.13 (9.71-16.82)	8.95 (5.78-16.92)	1.56 (0.75-2.29)
**Senegal**	51.61 (38.69-64.29)	39.37 (27.95-56.96)	1.34 (0.89-1.77)	47.94 (36.04-60.24)	34.89 (24.86-51.59)	1.4 (0.93-1.85)	46.81 (35.51-58.44)	39.55 (28.02-57.34)	1.21 (0.8-1.6)	39.52 (29.97-49.48)	34.11 (24.26-50.49)	1.18 (0.79-1.57)
**Sierra Leone**	47.99 (35.95-63.93)	41.38 (27.5-68.93)	1.2 (0.7-1.68)	62.29 (47.89-80.75)	44.85 (30.14-76.35)	1.44 (0.84-1.97)	55.37 (42.46-71.79)	46.64 (30.83-77.72)	1.23 (0.72-1.69)	38.84 (29.05-51.58)	37.66 (25.15-64.6)	1.07 (0.62-1.5)
**Togo**	62.48 (44.41-82.3)	33.92 (18.04-58.5)	1.99 (1.07-3.55)	73.57 (53.9-95.73)	34.98 (18.68-60.52)	2.27 (1.24-4.01)	66.06 (48.07-85.57)	36.23 (19.15-62.51)	1.97 (1.07-3.5)	48.72 (34.86-64.19)	30.68 (16.26-53.2)	1.71 (0.92-3.08)

eTable 11. Tuberculosis age-standardized risk-deleted mortality rate for drug use, intimate partner violence, unsafe sex, and all three risk factors combined among HIV-positive men and women for 204 countries and territories in 2019

	Drug use			Intimate partner violence			Unsafe sex			All risk factors		
	Male	Female	Ratio	Male	Female	Ratio	Male	Female	Ratio	Male	Female	Ratio
**Global**	2.41 (1.68-3.09)	2.71 (1.91-3.48)	0.89 (0.86-0.93)	-	2.38 (1.65-3.11)	-	0.56 (0.39-0.74)	0.48 (0.34-0.62)	1.17 (1.12-1.23)	0.54 (0.38-0.71)	0.44 (0.31-0.58)	1.21 (1.15-1.28)
**Central Europe, Eastern Europe, and Central Asia**	0.19 (0.11-0.31)	0.15 (0.1-0.23)	1.23 (0.9-1.48)	-	0.2 (0.12-0.3)	-	0.3 (0.19-0.48)	0.07 (0.05-0.12)	4.1 (3.61-4.7)	0.12 (0.07-0.18)	0.05 (0.03-0.08)	2.35 (1.8-2.77)
**Central Asia**	0.07 (0.04-0.11)	0.05 (0.03-0.08)	1.39 (1.2-1.56)	-	0.09 (0.06-0.13)	-	0.17 (0.11-0.25)	0.05 (0.03-0.07)	3.58 (3.34-3.83)	0.05 (0.03-0.08)	0.03 (0.02-0.04)	2.05 (1.74-2.33)
**Armenia**	0.15 (0.09-0.21)	0.06 (0.04-0.09)	2.43 (2.34-2.53)	-	0.07 (0.04-0.09)	-	0.15 (0.09-0.21)	0.02 (0.01-0.03)	8.03 (7.52-8.56)	0.07 (0.05-0.11)	0.02 (0.01-0.02)	4.72 (4.43-5.03)
**Azerbaijan**	0.04 (0.03-0.07)	0.03 (0.02-0.05)	1.33 (1.21-1.45)	-	0.05 (0.03-0.07)	-	0.11 (0.07-0.16)	0.02 (0.02-0.04)	4.6 (4.25-5.08)	0.03 (0.02-0.05)	0.02 (0.01-0.03)	1.99 (1.82-2.2)
**Georgia**	0.14 (0.1-0.16)	0.08 (0.06-0.09)	1.84 (1.71-2.01)	-	0.1 (0.08-0.12)	-	0.28 (0.2-0.31)	0.04 (0.03-0.04)	7.35 (6.76-7.98)	0.1 (0.07-0.11)	0.03 (0.02-0.03)	3.5 (3.24-3.79)
**Kazakhstan**	0.04 (0.02-0.06)	0.03 (0.02-0.05)	1.17 (1.09-1.25)	-	0.04 (0.03-0.06)	-	0.08 (0.05-0.13)	0.02 (0.01-0.02)	5.19 (4.88-5.48)	0.02 (0.01-0.04)	0.01 (0.01-0.02)	2.32 (2.14-2.5)
**Kyrgyzstan**	0.09 (0.06-0.15)	0.09 (0.06-0.14)	1.03 (0.95-1.1)	-	0.14 (0.08-0.21)	-	0.27 (0.17-0.42)	0.07 (0.05-0.11)	3.75 (3.52-3.97)	0.08 (0.05-0.12)	0.05 (0.03-0.07)	1.65 (1.54-1.76)
**Mongolia**	0.02 (0.01-0.03)	0.01 (0-0.01)	2.86 (1.77-5.05)	-	0.01 (0.01-0.02)	-	0.04 (0.02-0.06)	0 (0-0.01)	7.93 (5.48-14.2)	0.01 (0.01-0.02)	0 (0-0)	4.18 (2.72-7.34)
**Tajikistan**	0.02 (0.01-0.03)	0.11 (0.07-0.17)	0.17 (0.15-0.19)	-	0.17 (0.1-0.26)	-	0.06 (0.03-0.09)	0.08 (0.05-0.12)	0.71 (0.65-0.76)	0.01 (0.01-0.02)	0.04 (0.03-0.07)	0.33 (0.29-0.36)

**Turkmenistan**	0.19 (0.09-0.34)	0.1 (0.06-0.16)	1.91 (1.25-2.43)	-	0.16 (0.1-0.25)	-	0.41 (0.25-0.66)	0.08 (0.04-0.12)	5.51 (4.62-6.58)	0.13 (0.07-0.21)	0.04 (0.03-0.07)	3.07 (2.1-3.8)
**Uzbekistan**	0.08 (0.04-0.14)	0.04 (0.02-0.07)	1.91 (1.47-2.26)	-	0.1 (0.06-0.16)	-	0.21 (0.13-0.33)	0.07 (0.04-0.1)	3.24 (2.93-3.64)	0.06 (0.03-0.1)	0.03 (0.01-0.04)	2.34 (1.86-2.68)
**Central Europe**	0.05 (0.03-0.08)	0.03 (0.02-0.05)	1.85 (1.75-1.93)	-	0.03 (0.02-0.05)	-	0.03 (0.02-0.05)	0.01 (0.01-0.02)	3.48 (3.29-3.7)	0.02 (0.01-0.04)	0.01 (0.01-0.01)	2.53 (2.44-2.62)
**Albania**	0.02 (0.01-0.03)	0.01 (0-0.01)	2.04 (2.03-2.05)	-	0.01 (0-0.01)	-	0 (0-0)	0 (0-0)	1.32 (1.26-1.4)	0 (0-0)	0 (0-0)	1.32 (1.26-1.39)
**Bosnia and Herzegovina**	0.02 (0.01-0.02)	0.01 (0.01-0.03)	1.9 (0.79-3.48)	-	0.01 (0-0.02)	-	0 (0-0)	0 (0-0)	1.28 (1.1-1.63)	0 (0-0)	0 (0-0)	1.28 (1.11-1.62)
**Bulgaria**	0 (0-0.01)	0 (0-0)	1.73 (1.66-1.79)	-	0 (0-0)	-	0.01 (0-0.01)	0 (0-0)	5.39 (5.18-5.62)	0 (0-0)	0 (0-0)	2.85 (2.73-2.98)
**Croatia**	0.02 (0.02-0.04)	0.01 (0.01-0.01)	2.74 (2.7-2.78)	-	0.01 (0.01-0.01)	-	0 (0-0.01)	0 (0-0)	2.67 (2.48-2.89)	0 (0-0.01)	0 (0-0)	2.51 (2.36-2.66)
**Czechia**	0.03 (0.02-0.05)	0.01 (0.01-0.02)	2.41 (2.38-2.42)	-	0.01 (0.01-0.02)	-	0.01 (0-0.01)	0 (0-0)	3.19 (3.06-3.33)	0.01 (0-0.01)	0 (0-0)	3.11 (3-3.23)
**Hungary**	0.02 (0.01-0.03)	0 (0-0.01)	4.1 (4.07-4.13)	-	0 (0-0.01)	-	0 (0-0)	0 (0-0)	2.57 (2.23-3.03)	0 (0-0)	0 (0-0)	2.53 (2.21-2.95)
**Montenegro**	0.01 (0.01-0.02)	0.01 (0.01-0.01)	1.62 (1.56-1.66)	-	0.01 (0.01-0.01)	-	0.01 (0-0.01)	0.01 (0-0.01)	1.47 (1.39-1.59)	0.01 (0-0.01)	0.01 (0-0.01)	1.36 (1.32-1.39)
**North Macedonia**	0.02 (0.01-0.03)	0.02 (0.01-0.03)	1.11 (1.08-1.14)	-	0.02 (0.01-0.03)	-	0.01 (0.01-0.02)	0.01 (0.01-0.02)	1.15 (1.12-1.21)	0.01 (0.01-0.02)	0.01 (0.01-0.02)	1.13 (1.11-1.17)
**Poland**	0.03 (0.02-0.05)	0.01 (0.01-0.02)	2.3 (1.91-2.57)	-	0.01 (0.01-0.02)	-	0.02 (0.01-0.03)	0 (0-0.01)	5.3 (4.35-6.75)	0.01 (0.01-0.02)	0 (0-0.01)	3.76 (3.47-4.03)
**Romania**	0.14 (0.08-0.21)	0.1 (0.06-0.16)	1.34 (1.29-1.39)	-	0.11 (0.07-0.17)	-	0.13 (0.08-0.2)	0.04 (0.02-0.06)	3.59 (3.39-3.8)	0.07 (0.04-0.1)	0.03 (0.02-0.05)	2.12 (2.02-2.24)
**Serbia**	0.18 (0.11-0.26)	0.07 (0.04-0.1)	2.73 (2.58-2.94)	-	0.07 (0.04-0.1)	-	0.07 (0.04-0.11)	0.03 (0.02-0.04)	2.57 (2.28-2.78)	0.06 (0.04-0.09)	0.03 (0.02-0.04)	2.21 (2.01-2.34)

**Slovakia**	0 (0-0)	0 (0-0)	2.04 (2.01-2.07)	-	0 (0-0)	-	0 (0-0)	0 (0-0)	1.39 (1.32-1.49)	0 (0-0)	0 (0-0)	1.36 (1.3-1.44)
**Slovenia**	0.01 (0.01-0.02)	0 (0-0)	4.79 (4.69-4.86)	-	0 (0-0)	-	0 (0-0)	0 (0-0)	3.35 (3.08-3.73)	0 (0-0)	0 (0-0)	3.24 (3.01-3.55)
**Eastern Europe**	0.32 (0.17-0.53)	0.26 (0.16-0.4)	1.19 (0.82-1.48)	-	0.33 (0.2-0.51)	-	0.51 (0.31-0.8)	0.12 (0.07-0.19)	4.25 (3.67-4.99)	0.19 (0.11-0.31)	0.08 (0.05-0.13)	2.39 (1.76-2.84)
**Belarus**	0.14 (0.09-0.22)	0.08 (0.05-0.13)	1.7 (1.66-1.75)	-	0.08 (0.05-0.13)	-	0.11 (0.07-0.16)	0.01 (0.01-0.02)	7.12 (6.76-7.49)	0.06 (0.04-0.09)	0.01 (0.01-0.02)	5.17 (4.75-5.7)
**Estonia**	0.27 (0.17-0.42)	0.13 (0.08-0.19)	2.14 (1.95-2.29)	-	0.14 (0.08-0.21)	-	0.26 (0.15-0.39)	0.03 (0.02-0.04)	9.4 (8.5-10.36)	0.12 (0.08-0.19)	0.02 (0.01-0.03)	5.76 (5.11-6.44)
**Latvia**	0.17 (0.11-0.27)	0.28 (0.17-0.42)	0.62 (0.59-0.66)	-	0.34 (0.2-0.52)	-	0.3 (0.18-0.45)	0.1 (0.06-0.16)	2.84 (2.66-3.02)	0.11 (0.07-0.16)	0.07 (0.04-0.11)	1.55 (1.41-1.7)
**Lithuania**	0.02 (0.01-0.04)	0.09 (0.05-0.13)	0.28 (0.26-0.31)	-	0.17 (0.1-0.26)	-	0.09 (0.06-0.14)	0.1 (0.06-0.15)	0.98 (0.92-1.03)	0.02 (0.01-0.03)	0.04 (0.03-0.07)	0.45 (0.42-0.48)
**Republic of Moldova**	0.24 (0.15-0.37)	0.11 (0.07-0.16)	2.23 (2.17-2.28)	-	0.1 (0.06-0.16)	-	0.09 (0.05-0.14)	0.01 (0.01-0.02)	7.92 (7.3-8.51)	0.06 (0.04-0.1)	0.01 (0.01-0.01)	7 (6.35-7.71)
**Russian Federation**	0.28 (0.15-0.47)	0.19 (0.12-0.3)	1.44 (1.02-1.78)	-	0.24 (0.15-0.37)	-	0.45 (0.27-0.71)	0.1 (0.06-0.16)	4.49 (3.83-5.29)	0.17 (0.1-0.27)	0.07 (0.04-0.1)	2.63 (1.9-3.12)
**Ukraine**	0.49 (0.23-0.84)	0.55 (0.33-0.85)	0.89 (0.57-1.14)	-	0.7 (0.42-1.1)	-	0.86 (0.5-1.37)	0.22 (0.13-0.35)	3.99 (3.33-4.86)	0.3 (0.16-0.48)	0.15 (0.09-0.23)	2.02 (1.36-2.49)
**High-income**	0.18 (0.14-0.22)	0.07 (0.05-0.08)	2.6 (2.54-2.67)	-	0.07 (0.05-0.08)	-	0.02 (0.02-0.03)	0.01 (0.01-0.01)	2.66 (2.34-2.9)	0.02 (0.01-0.03)	0.01 (0.01-0.01)	2.68 (2.39-2.89)
**Australasia**	0.02 (0.01-0.04)	0.01 (0-0.01)	3.48 (3.46-3.5)	-	0.01 (0-0.01)	-	0 (0-0)	0 (0-0)	1.65 (1.5-1.83)	0 (0-0)	0 (0-0)	1.67 (1.52-1.85)
**Australia**	0.02 (0.01-0.04)	0.01 (0-0.01)	3.43 (3.42-3.44)	-	0.01 (0-0.01)	-	0 (0-0)	0 (0-0)	1.62 (1.49-1.76)	0 (0-0)	0 (0-0)	1.64 (1.51-1.77)
**New Zealand**	0.03 (0.02-0.04)	0.01 (0-0.01)	3.86 (3.74-3.96)	-	0.01 (0-0.01)	-	0 (0-0)	0 (0-0)	1.87 (1.48-2.52)	0 (0-0)	0 (0-0)	1.89 (1.53-2.51)
**High-income Asia Pacific**	0.07 (0.06-0.09)	0.02 (0.01-0.02)	4.66 (4.22-5.28)	-	0.01 (0.01-0.02)	-	0.01 (0.01-0.01)	0 (0-0)	3.03 (2.72-3.46)	0.01 (0.01-0.01)	0 (0-0)	3.09 (2.76-3.53)
**Brunei Darussalam**	0.1 (0.07-0.16)	0.05 (0.03-0.07)	2.18 (2.18-2.18)	-	0.04 (0.03-0.07)	-	0.02 (0.01-0.03)	0.01 (0.01-0.02)	1.7 (1.58-1.82)	0.02 (0.01-0.03)	0.01 (0.01-0.02)	1.76 (1.63-1.92)

**Japan**	0.04 (0.03-0.06)	0.02 (0.01-0.02)	2.72 (2.69-2.77)	-	0.01 (0.01-0.02)	-	0.01 (0-0.01)	0 (0-0)	2.05 (1.96-2.16)	0.01 (0-0.01)	0 (0-0)	2.07 (1.98-2.19)
**Republic of Korea**	0.14 (0.13-0.16)	0.02 (0.02-0.02)	8.33 (7.49-9.29)	-	0.02 (0.01-0.02)	-	0.02 (0.01-0.02)	0 (0-0)	5.07 (4.28-6.03)	0.02 (0.01-0.02)	0 (0-0)	5.15 (4.34-6.13)
**Singapore**	0.07 (0.04-0.11)	0.01 (0-0.01)	9.55 (9.53-9.57)	-	0.01 (0-0.01)	-	0.01 (0-0.01)	0 (0-0)	5.43 (4.81-6.16)	0.01 (0-0.01)	0 (0-0)	5.73 (5.04-6.58)
**High-income North America**	0.05 (0.03-0.08)	0.02 (0.01-0.03)	2.69 (2.65-2.73)	-	0.02 (0.01-0.03)	-	0.01 (0-0.01)	0 (0-0.01)	2.2 (2.07-2.34)	0.01 (0-0.01)	0 (0-0)	2.49 (2.29-2.73)
**Canada**	0.07 (0.04-0.11)	0.03 (0.02-0.04)	2.65 (2.57-2.75)	-	0.03 (0.02-0.05)	-	0.02 (0.01-0.03)	0.01 (0-0.01)	2.24 (1.99-2.53)	0.01 (0.01-0.02)	0.01 (0-0.01)	2.26 (2.07-2.45)
**United States of America**	0.05 (0.03-0.07)	0.02 (0.01-0.03)	2.7 (2.66-2.74)	-	0.02 (0.01-0.03)	-	0.01 (0-0.01)	0 (0-0.01)	2.2 (2.06-2.35)	0.01 (0-0.01)	0 (0-0)	2.54 (2.32-2.83)
**Southern Latin America**	1.88 (1.47-2)	0.72 (0.55-0.76)	2.63 (2.53-2.73)	-	0.68 (0.52-0.74)	-	0.21 (0.15-0.26)	0.06 (0.04-0.07)	3.68 (2.88-4.13)	0.2 (0.14-0.23)	0.05 (0.04-0.06)	3.65 (2.96-4.03)
**Argentina**	2.05 (1.55-2.21)	0.92 (0.71-0.98)	2.24 (2.14-2.35)	-	0.89 (0.67-0.97)	-	0.29 (0.19-0.35)	0.07 (0.06-0.09)	3.85 (2.91-4.38)	0.25 (0.18-0.31)	0.07 (0.05-0.08)	3.7 (2.91-4.12)
**Chile**	1.73 (1.41-1.81)	0.34 (0.28-0.36)	5.04 (4.83-5.24)	-	0.28 (0.23-0.32)	-	0.05 (0.04-0.06)	0.02 (0.02-0.02)	2.51 (2.31-2.74)	0.05 (0.04-0.06)	0.02 (0.01-0.02)	2.69 (2.43-2.97)
**Uruguay**	0.5 (0.31-0.77)	0.13 (0.08-0.19)	3.97 (3.81-4.09)	-	0.16 (0.1-0.25)	-	0.2 (0.12-0.32)	0.05 (0.03-0.08)	4.19 (3.88-4.57)	0.14 (0.08-0.21)	0.03 (0.02-0.05)	4.31 (4.1-4.55)
**Western Europe**	0.11 (0.07-0.16)	0.04 (0.02-0.06)	2.71 (2.67-2.76)	-	0.04 (0.02-0.06)	-	0.01 (0.01-0.02)	0.01 (0-0.01)	1.94 (1.84-2.05)	0.01 (0.01-0.02)	0.01 (0-0.01)	1.97 (1.85-2.09)
**Andorra**	0.07 (0.04-0.12)	0.03 (0.02-0.05)	2.24 (2.06-3.26)	-	0.03 (0.02-0.05)	-	0.01 (0-0.02)	0 (0-0.01)	2.11 (1.58-3.31)	0.01 (0-0.01)	0 (0-0.01)	2.25 (1.66-3.69)
**Austria**	0.05 (0.03-0.08)	0.02 (0.01-0.03)	3.12 (3.08-3.16)	-	0.02 (0.01-0.03)	-	0.01 (0.01-0.02)	0 (0-0.01)	2.4 (2.28-2.54)	0.01 (0.01-0.01)	0 (0-0.01)	2.4 (2.29-2.52)
**Belgium**	0.07 (0.04-0.11)	0.04 (0.02-0.06)	1.92 (1.91-1.93)	-	0.04 (0.02-0.06)	-	0.01 (0-0.01)	0.01 (0-0.01)	1.13 (1.07-1.18)	0.01 (0-0.01)	0.01 (0-0.01)	1.15 (1.1-1.21)
**Cyprus**	0.03 (0.02-0.05)	0.01 (0.01-0.02)	3.34 (3.32-3.37)	-	0.01 (0.01-0.01)	-	0 (0-0)	0 (0-0)	2.54 (2.08-3.01)	0 (0-0)	0 (0-0)	2.65 (2.19-3.13)
**Denmark**	0.03 (0.02-0.05)	0.01 (0.01-0.02)	2.22 (2.2-2.23)	-	0.01 (0.01-0.02)	-	0 (0-0.01)	0 (0-0)	1.54 (1.45-1.64)	0 (0-0)	0 (0-0)	1.65 (1.53-1.78)

**Finland**	0.04 (0.02-0.05)	0.02 (0.01-0.02)	2.68 (2.57-2.81)	-	0.01 (0.01-0.02)	-	0.01 (0-0.01)	0 (0-0.01)	1.86 (1.72-1.99)	0.01 (0-0.01)	0 (0-0.01)	1.89 (1.76-2.02)
**France**	0.25 (0.15-0.36)	0.1 (0.06-0.14)	2.56 (2.5-2.61)	-	0.09 (0.05-0.13)	-	0.01 (0.01-0.02)	0.01 (0.01-0.01)	1.21 (1.14-1.28)	0.01 (0.01-0.02)	0.01 (0.01-0.01)	1.24 (1.16-1.33)
**Germany**	0.06 (0.03-0.09)	0.02 (0.01-0.03)	3.24 (3.23-3.25)	-	0.02 (0.01-0.03)	-	0.01 (0-0.01)	0 (0-0.01)	1.9 (1.8-2.02)	0.01 (0-0.01)	0 (0-0.01)	1.96 (1.85-2.1)
**Greece**	0.09 (0.08-0.1)	0.03 (0.02-0.03)	3.34 (3.09-3.6)	-	0.04 (0.03-0.04)	-	0.06 (0.05-0.06)	0.02 (0.02-0.02)	3.03 (2.73-3.35)	0.04 (0.03-0.04)	0.01 (0.01-0.02)	2.66 (2.43-2.91)
**Iceland**	0.02 (0.01-0.03)	0 (0-0.01)	3.61 (3.28-3.89)	-	0.01 (0-0.01)	-	0.02 (0.01-0.03)	0 (0-0.01)	4.24 (3.96-4.56)	0.01 (0.01-0.01)	0 (0-0)	3.42 (3.16-3.58)
**Ireland**	0.09 (0.07-0.1)	0.04 (0.03-0.04)	2.58 (2.38-2.8)	-	0.04 (0.03-0.04)	-	0.01 (0.01-0.02)	0.01 (0.01-0.01)	1.92 (1.74-2.12)	0.01 (0.01-0.02)	0.01 (0-0.01)	1.96 (1.77-2.16)
**Israel**	0.03 (0.02-0.04)	0.01 (0.01-0.02)	2.32 (2.29-2.34)	-	0.01 (0.01-0.02)	-	0.01 (0-0.01)	0 (0-0.01)	1.72 (1.65-1.8)	0.01 (0-0.01)	0 (0-0.01)	1.7 (1.63-1.79)
**Italy**	0.04 (0.02-0.06)	0.01 (0.01-0.02)	3.04 (2.98-3.1)	-	0.01 (0.01-0.02)	-	0.01 (0-0.01)	0 (0-0)	2.15 (1.9-2.49)	0 (0-0.01)	0 (0-0)	2.31 (2.07-2.65)
**Luxembourg**	0.02 (0.01-0.04)	0.01 (0.01-0.01)	2.61 (2.57-2.64)	-	0.01 (0.01-0.02)	-	0.01 (0-0.01)	0 (0-0)	2.26 (2.12-2.4)	0.01 (0-0.01)	0 (0-0)	2.33 (2.18-2.49)
**Malta**	0.02 (0.01-0.04)	0.01 (0.01-0.01)	2.74 (2.71-2.77)	-	0.01 (0.01-0.01)	-	0 (0-0.01)	0 (0-0)	2.11 (1.96-2.31)	0 (0-0.01)	0 (0-0)	2.11 (1.96-2.29)
**Monaco**	0.31 (0.16-0.48)	0.19 (0.06-0.33)	1.71 (1.04-3.15)	-	0.19 (0.06-0.34)	-	0.04 (0.01-0.07)	0.03 (0.01-0.05)	1.53 (0.97-2.94)	0.03 (0.01-0.06)	0.02 (0-0.04)	1.67 (1.07-3.18)
**Netherlands**	0.09 (0.05-0.14)	0.04 (0.02-0.06)	2.17 (2.15-2.19)	-	0.04 (0.02-0.06)	-	0 (0-0.01)	0.01 (0-0.01)	0.81 (0.76-0.86)	0 (0-0.01)	0.01 (0-0.01)	0.82 (0.78-0.88)
**Norway**	0.05 (0.03-0.07)	0.02 (0.01-0.03)	2.07 (2.03-2.11)	-	0.02 (0.01-0.03)	-	0.01 (0-0.01)	0 (0-0.01)	1.21 (1.09-1.41)	0 (0-0.01)	0 (0-0.01)	1.22 (1.11-1.39)
**Portugal**	0.67 (0.42-1.03)	0.18 (0.11-0.28)	3.74 (3.71-3.78)	-	0.2 (0.12-0.3)	-	0.12 (0.08-0.19)	0.04 (0.02-0.06)	3.42 (3.26-3.57)	0.11 (0.07-0.16)	0.03 (0.02-0.05)	3.49 (3.32-3.68)
**San Marino**	0.1 (0.06-0.15)	0.06 (0.03-0.1)	1.53 (1.46-2.28)	-	0.06 (0.03-0.1)	-	0.01 (0.01-0.02)	0.01 (0-0.02)	1.33 (1.14-2.27)	0.01 (0.01-0.02)	0.01 (0-0.01)	1.45 (1.26-2.42)
**Spain**	0.13 (0.08-0.2)	0.04 (0.02-0.06)	3.57 (3.55-3.6)	-	0.04 (0.02-0.06)	-	0.02 (0.01-0.03)	0.01 (0-0.01)	2.58 (2.45-2.72)	0.02 (0.01-0.03)	0.01 (0-0.01)	2.58 (2.45-2.72)

**Sweden**	0.04 (0.02-0.06)	0.02 (0.01-0.04)	1.67 (1.61-1.74)	-	0.02 (0.01-0.04)	-	0.01 (0-0.01)	0.01 (0-0.01)	1.35 (1.21-1.54)	0.01 (0-0.01)	0.01 (0-0.01)	1.4 (1.29-1.55)
**Switzerland**	0.04 (0.02-0.06)	0.02 (0.01-0.03)	2.19 (2.18-2.21)	-	0.02 (0.01-0.03)	-	0 (0-0.01)	0 (0-0)	1.47 (1.37-1.56)	0 (0-0.01)	0 (0-0)	1.5 (1.4-1.59)
**United Kingdom**	0.05 (0.03-0.09)	0.03 (0.02-0.05)	1.81 (1.79-1.83)	-	0.03 (0.02-0.04)	-	0.01 (0-0.01)	0.01 (0-0.01)	1.15 (1.04-1.34)	0.01 (0-0.01)	0.01 (0-0.01)	1.18 (1.07-1.35)
**Latin America and Caribbean**	0.83 (0.52-1.25)	0.44 (0.27-0.67)	1.88 (1.81-1.93)	-	0.44 (0.27-0.66)	-	0.2 (0.12-0.31)	0.11 (0.07-0.17)	1.83 (1.7-2)	0.18 (0.11-0.28)	0.1 (0.06-0.15)	1.82 (1.71-1.95)
**Andean Latin America**	1.06 (0.67-1.58)	0.48 (0.3-0.72)	2.21 (2.12-2.35)	-	0.43 (0.27-0.65)	-	0.16 (0.1-0.24)	0.13 (0.08-0.2)	1.24 (1.17-1.34)	0.16 (0.1-0.24)	0.13 (0.08-0.19)	1.26 (1.18-1.37)
**Bolivia (Plurinational State of)**	0.55 (0.24-0.94)	0.3 (0.07-0.59)	2.09 (1.46-4.13)	-	0.26 (0.06-0.52)	-	0.07 (0.01-0.17)	0.06 (0.01-0.15)	1.44 (1.07-2.73)	0.07 (0.01-0.16)	0.05 (0-0.15)	1.53 (1.09-3.02)
**Ecuador**	2.09 (1.29-3.15)	0.69 (0.43-1.04)	3.03 (3.03-3.04)	-	0.63 (0.39-0.95)	-	0.15 (0.09-0.25)	0.08 (0.05-0.13)	1.93 (1.74-2.13)	0.15 (0.09-0.25)	0.08 (0.05-0.12)	2.02 (1.81-2.27)
**Peru**	0.73 (0.45-1.11)	0.44 (0.28-0.68)	1.63 (1.63-1.63)	-	0.41 (0.25-0.62)	-	0.2 (0.13-0.31)	0.19 (0.12-0.29)	1.07 (1.05-1.11)	0.2 (0.13-0.31)	0.19 (0.12-0.28)	1.08 (1.06-1.12)
**Caribbean**	1.49 (0.9-2.28)	1.36 (0.81-2.11)	1.09 (1.05-1.14)	-	1.27 (0.76-1.98)	-	0.38 (0.22-0.6)	0.39 (0.22-0.63)	0.98 (0.91-1.05)	0.37 (0.21-0.59)	0.35 (0.2-0.58)	1.04 (0.96-1.13)
**Antigua and Barbuda**	0.13 (0.08-0.2)	0.05 (0.03-0.08)	2.58 (2.57-2.59)	-	0.05 (0.03-0.07)	-	0.03 (0.02-0.05)	0.01 (0.01-0.02)	2.26 (2.11-2.4)	0.03 (0.02-0.04)	0.01 (0.01-0.02)	2.42 (2.23-2.65)
**Bahamas**	0.98 (0.58-1.52)	0.68 (0.41-1.06)	1.44 (1.43-1.44)	-	0.62 (0.37-0.97)	-	0.21 (0.12-0.35)	0.17 (0.1-0.29)	1.23 (1.12-1.34)	0.2 (0.12-0.33)	0.15 (0.09-0.26)	1.34 (1.21-1.49)
**Barbados**	0.16 (0.1-0.25)	0.08 (0.05-0.13)	1.91 (1.91-1.92)	-	0.08 (0.05-0.12)	-	0.04 (0.02-0.06)	0.02 (0.01-0.04)	1.57 (1.45-1.7)	0.03 (0.02-0.05)	0.02 (0.01-0.03)	1.7 (1.54-1.87)
**Belize**	1.97 (1.19-3.02)	0.98 (0.59-1.5)	2.01 (2.01-2.02)	-	0.93 (0.56-1.41)	-	0.46 (0.27-0.75)	0.26 (0.15-0.42)	1.79 (1.67-1.9)	0.44 (0.26-0.73)	0.23 (0.14-0.38)	1.89 (1.75-2.06)
**Cuba**	0.06 (0.04-0.09)	0.01 (0.01-0.02)	5.26 (5.24-5.27)	-	0.01 (0.01-0.02)	-	0.01 (0.01-0.02)	0 (0-0)	4.74 (4.48-5.01)	0.01 (0.01-0.02)	0 (0-0)	5.17 (4.81-5.64)
**Dominica**	1.3 (0.78-2.02)	0.46 (0.28-0.72)	2.81 (2.8-2.82)	-	0.43 (0.26-0.66)	-	0.32 (0.19-0.52)	0.14 (0.08-0.22)	2.37 (2.2-2.57)	0.32 (0.19-0.51)	0.13 (0.07-0.2)	2.52 (2.3-2.77)
**Dominican Republic**	2.45 (1.49-3.79)	1.4 (0.85-2.17)	1.75 (1.74-1.79)	-	1.31 (0.8-2.04)	-	0.5 (0.29-0.82)	0.32 (0.19-0.53)	1.57 (1.45-1.69)	0.48 (0.28-0.79)	0.29 (0.16-0.48)	1.69 (1.55-1.86)

**Grenada**	0.13 (0.08-0.2)	0.05 (0.03-0.08)	2.46 (2.45-2.47)	-	0.05 (0.03-0.08)	-	0.03 (0.02-0.05)	0.01 (0.01-0.02)	2.11 (1.94-2.28)	0.03 (0.02-0.04)	0.01 (0.01-0.02)	2.27 (2.06-2.51)
**Guyana**	3.54 (2.15-5.5)	2.4 (1.45-3.72)	1.48 (1.47-1.48)	-	2.2 (1.32-3.43)	-	0.81 (0.46-1.34)	0.64 (0.36-1.07)	1.26 (1.18-1.35)	0.78 (0.45-1.29)	0.57 (0.33-0.94)	1.37 (1.26-1.52)
**Haiti**	3.5 (2.05-5.54)	3.9 (2.29-6.18)	0.9 (0.89-0.9)	-	3.63 (2.09-5.74)	-	0.83 (0.48-1.38)	0.99 (0.55-1.68)	0.83 (0.77-0.89)	0.81 (0.46-1.34)	0.9 (0.5-1.5)	0.89 (0.82-0.96)
**Jamaica**	0.22 (0.13-0.33)	0.15 (0.09-0.23)	1.42 (1.42-1.43)	-	0.14 (0.09-0.22)	-	0.05 (0.03-0.08)	0.04 (0.02-0.06)	1.26 (1.17-1.34)	0.05 (0.03-0.08)	0.04 (0.02-0.06)	1.35 (1.25-1.48)
**Saint Kitts and Nevis**	1.19 (0.71-1.86)	0.52 (0.31-0.82)	2.27 (2.26-2.31)	-	0.48 (0.29-0.75)	-	0.26 (0.15-0.43)	0.14 (0.08-0.23)	1.93 (1.8-2.08)	0.25 (0.14-0.42)	0.12 (0.07-0.2)	2.08 (1.92-2.31)
**Saint Lucia**	0.44 (0.27-0.67)	0.25 (0.15-0.38)	1.76 (1.75-1.76)	-	0.23 (0.14-0.35)	-	0.1 (0.06-0.16)	0.06 (0.04-0.1)	1.56 (1.45-1.66)	0.09 (0.06-0.15)	0.06 (0.03-0.09)	1.68 (1.55-1.85)
**Saint Vincent and the Grenadines**	1.01 (0.62-1.54)	0.53 (0.33-0.82)	1.88 (1.87-1.89)	-	0.5 (0.3-0.75)	-	0.23 (0.13-0.37)	0.14 (0.08-0.23)	1.62 (1.5-1.74)	0.22 (0.13-0.36)	0.13 (0.07-0.21)	1.74 (1.59-1.92)
**Suriname**	1.17 (0.72-1.79)	0.77 (0.47-1.18)	1.51 (1.5-1.51)	-	0.71 (0.43-1.1)	-	0.27 (0.15-0.44)	0.22 (0.13-0.35)	1.25 (1.16-1.34)	0.26 (0.15-0.43)	0.2 (0.11-0.32)	1.34 (1.22-1.47)
**Trinidad and Tobago**	0.7 (0.43-1.08)	0.47 (0.29-0.72)	1.5 (1.49-1.51)	-	0.43 (0.26-0.67)	-	0.16 (0.09-0.26)	0.13 (0.08-0.21)	1.24 (1.16-1.34)	0.16 (0.09-0.26)	0.12 (0.07-0.19)	1.34 (1.22-1.47)
**Central Latin America**	0.67 (0.43-1.02)	0.22 (0.14-0.33)	3.03 (2.95-3.08)	-	0.2 (0.13-0.3)	-	0.05 (0.03-0.08)	0.03 (0.02-0.04)	1.88 (1.74-2.05)	0.05 (0.03-0.08)	0.03 (0.02-0.04)	1.96 (1.8-2.16)
**Colombia**	0.57 (0.35-0.87)	0.18 (0.11-0.27)	3.19 (3.18-3.2)	-	0.15 (0.09-0.23)	-	0.04 (0.02-0.06)	0.02 (0.01-0.03)	2.16 (2-2.36)	0.04 (0.02-0.06)	0.02 (0.01-0.02)	2.35 (2.14-2.61)
**Costa Rica**	0.21 (0.13-0.32)	0.07 (0.04-0.1)	3.07 (3.06-3.07)	-	0.06 (0.04-0.09)	-	0.02 (0.01-0.03)	0.01 (0.01-0.02)	1.85 (1.69-2.07)	0.02 (0.01-0.03)	0.01 (0.01-0.01)	1.91 (1.74-2.16)
**El Salvador**	0.37 (0.23-0.58)	0.14 (0.09-0.22)	2.63 (2.62-2.64)	-	0.13 (0.08-0.2)	-	0.05 (0.03-0.07)	0.03 (0.02-0.05)	1.34 (1.27-1.44)	0.05 (0.03-0.07)	0.03 (0.02-0.05)	1.36 (1.29-1.46)
**Guatemala**	1.29 (0.8-1.98)	0.51 (0.32-0.79)	2.52 (2.52-2.52)	-	0.48 (0.3-0.74)	-	0.11 (0.07-0.18)	0.08 (0.05-0.12)	1.45 (1.37-1.56)	0.11 (0.07-0.18)	0.08 (0.05-0.12)	1.47 (1.39-1.59)
**Honduras**	0.58 (0.47-0.75)	0.32 (0.22-0.47)	1.86 (1.29-2.45)	-	0.3 (0.21-0.44)	-	0.05 (0.03-0.09)	0.04 (0.02-0.08)	1.47 (1.1-2.1)	0.05 (0.03-0.09)	0.04 (0.01-0.08)	1.52 (1.12-2.22)
**Mexico**	0.61 (0.38-0.93)	0.18 (0.11-0.27)	3.44 (3.43-3.46)	-	0.16 (0.1-0.24)	-	0.05 (0.03-0.08)	0.02 (0.01-0.03)	2.23 (2.05-2.47)	0.05 (0.03-0.08)	0.02 (0.01-0.03)	2.34 (2.14-2.61)

**Nicaragua**	0.16 (0.1-0.26)	0.1 (0.06-0.15)	1.73 (1.72-1.73)	-	0.09 (0.05-0.13)	-	0.02 (0.01-0.03)	0.01 (0.01-0.02)	1.24 (1.18-1.32)	0.02 (0.01-0.03)	0.01 (0.01-0.02)	1.27 (1.21-1.37)
**Panama**	1.8 (1.08-2.75)	0.59 (0.35-0.9)	3.06 (3.05-3.07)	-	0.53 (0.31-0.8)	-	0.12 (0.07-0.2)	0.05 (0.03-0.09)	2.28 (2.09-2.48)	0.12 (0.07-0.2)	0.05 (0.03-0.08)	2.43 (2.2-2.71)
**Venezuela (Bolivarian Republic of)**	0.88 (0.53-1.36)	0.31 (0.19-0.48)	2.81 (2.8-2.82)	-	0.28 (0.17-0.43)	-	0.06 (0.03-0.1)	0.03 (0.02-0.05)	1.86 (1.71-2.04)	0.06 (0.03-0.1)	0.03 (0.02-0.05)	1.97 (1.79-2.19)
**Tropical Latin America**	0.79 (0.48-1.22)	0.48 (0.29-0.73)	1.66 (1.55-1.72)	-	0.52 (0.32-0.79)	-	0.32 (0.19-0.51)	0.13 (0.08-0.21)	2.46 (2.26-2.74)	0.24 (0.15-0.37)	0.11 (0.07-0.17)	2.2 (2.01-2.35)
**Brazil**	0.79 (0.48-1.21)	0.47 (0.29-0.71)	1.68 (1.57-1.74)	-	0.51 (0.31-0.78)	-	0.31 (0.18-0.5)	0.12 (0.07-0.2)	2.53 (2.31-2.82)	0.23 (0.14-0.36)	0.1 (0.06-0.16)	2.28 (2.06-2.44)
**Paraguay**	0.86 (0.53-1.33)	0.68 (0.42-1.04)	1.27 (1.18-1.33)	-	0.75 (0.45-1.15)	-	0.51 (0.3-0.8)	0.3 (0.18-0.46)	1.67 (1.56-1.84)	0.4 (0.24-0.6)	0.27 (0.17-0.42)	1.45 (1.39-1.49)
**North Africa and Middle East**	0.05 (0.03-0.08)	0.07 (0.04-0.12)	0.67 (0.56-0.79)	-	0.08 (0.05-0.13)	-	0.07 (0.04-0.1)	0.03 (0.02-0.05)	1.96 (1.68-2.32)	0.03 (0.02-0.05)	0.03 (0.02-0.04)	1.29 (1.07-1.52)
**North Africa and Middle East**	0.05 (0.03-0.08)	0.07 (0.04-0.12)	0.67 (0.56-0.79)	-	0.08 (0.05-0.13)	-	0.07 (0.04-0.1)	0.03 (0.02-0.05)	1.96 (1.68-2.32)	0.03 (0.02-0.05)	0.03 (0.02-0.04)	1.29 (1.07-1.52)
**Afghanistan**	0.01 (0-0.02)	0.01 (0-0.01)	1.29 (0.88-3.82)	-	0.01 (0-0.01)	-	0.01 (0-0.02)	0 (0-0.01)	5.73 (2.94-22.66)	0.01 (0-0.01)	0 (0-0.01)	3.3 (1.77-12.61)
**Algeria**	0.01 (0.01-0.02)	0.02 (0.01-0.03)	0.81 (0.44-0.95)	-	0.02 (0.01-0.02)	-	0.02 (0.01-0.03)	0 (0-0.01)	4.17 (1.9-5.96)	0.01 (0-0.01)	0 (0-0.01)	2.36 (1.27-3.42)
**Bahrain**	0.01 (0.01-0.02)	0.01 (0.01-0.02)	0.91 (0.83-0.99)	-	0.01 (0.01-0.02)	-	0.03 (0.02-0.04)	0.01 (0-0.01)	4.06 (3.53-4.55)	0.01 (0-0.01)	0 (0-0.01)	1.78 (1.61-1.95)
**Egypt**	0.01 (0-0.01)	0.01 (0-0.01)	1.1 (0.95-1.23)	-	0.01 (0-0.01)	-	0.01 (0.01-0.02)	0 (0-0)	4.21 (3.72-5.17)	0.01 (0-0.01)	0 (0-0)	2.38 (2.07-2.78)
**Iran (Islamic Republic of)**	0.13 (0.08-0.2)	0.16 (0.1-0.23)	0.87 (0.72-1.06)	-	0.22 (0.14-0.31)	-	0.25 (0.17-0.36)	0.15 (0.1-0.21)	1.71 (1.44-2.11)	0.11 (0.07-0.15)	0.1 (0.06-0.14)	1.12 (0.94-1.32)
**Iraq**	0 (0-0)	0 (0-0)	0.35 (0.33-0.36)	-	0 (0-0)	-	0 (0-0)	0 (0-0)	1 (0.95-1.06)	0 (0-0)	0 (0-0)	0.85 (0.8-0.9)
**Jordan**	0 (0-0)	0 (0-0)	1.37 (1.31-1.42)	-	0 (0-0)	-	0 (0-0)	0 (0-0)	1.92 (1.77-2.1)	0 (0-0)	0 (0-0)	1.7 (1.62-1.79)
**Kuwait**	0 (0-0)	0 (0-0)	0.48 (0.41-0.53)	-	0 (0-0)	-	0 (0-0)	0 (0-0)	1.12 (1.03-1.23)	0 (0-0)	0 (0-0)	0.68 (0.63-0.72)

**Lebanon**	0.02 (0.01-0.04)	0.02 (0-0.05)	0.98 (0.66-3.27)	-	0.02 (0-0.05)	-	0.02 (0.01-0.05)	0.01 (0-0.02)	3.76 (1.89-20.01)	0.01 (0-0.03)	0.01 (0-0.02)	2.25 (1.29-11.05)
**Libya**	0.05 (0.01-0.11)	0.09 (0-0.2)	0.7 (0.21-2.44)	-	0.09 (0-0.2)	-	0.06 (0.01-0.14)	0.02 (0-0.06)	3.47 (1.04-11.28)	0.03 (0-0.07)	0.02 (0-0.05)	2.03 (0.72-6.23)
**Morocco**	0.12 (0.01-0.32)	0.16 (0.01-0.45)	0.87 (0.42-2.35)	-	0.16 (0.01-0.44)	-	0.14 (0.01-0.37)	0.06 (0-0.19)	3.95 (1.35-13.44)	0.08 (0.01-0.23)	0.05 (0-0.17)	2.33 (1.07-7.41)
**Oman**	0.02 (0.01-0.03)	0.01 (0-0.01)	2.25 (2.14-2.34)	-	0.01 (0-0.01)	-	0 (0-0.01)	0 (0-0)	6.65 (5.73-7.7)	0 (0-0.01)	0 (0-0)	5.91 (5.22-6.69)
**Palestine**	0 (0-0.01)	0 (0-0.01)	0.82 (0.69-0.92)	-	0 (0-0.01)	-	0 (0-0.01)	0 (0-0)	2.95 (2.7-3.28)	0 (0-0)	0 (0-0)	1.8 (1.59-1.95)
**Qatar**	0 (0-0)	0 (0-0)	0.75 (0.65-0.83)	-	0 (0-0)	-	0 (0-0)	0 (0-0)	3.07 (2.82-3.38)	0 (0-0)	0 (0-0)	1.87 (1.67-2.06)
**Saudi Arabia**	0.06 (0.04-0.1)	0.15 (0.09-0.24)	0.41 (0.34-0.47)	-	0.15 (0.09-0.23)	-	0.08 (0.05-0.12)	0.03 (0.02-0.05)	2.38 (2.04-2.89)	0.04 (0.02-0.06)	0.03 (0.02-0.04)	1.43 (1.19-1.74)
**Sudan**	0.17 (0.08-0.29)	0.43 (0.21-0.71)	0.39 (0.31-0.46)	-	0.4 (0.2-0.66)	-	0.22 (0.11-0.38)	0.08 (0.04-0.14)	2.73 (2.38-3.15)	0.1 (0.05-0.17)	0.06 (0.03-0.11)	1.61 (1.28-1.92)
**Syrian Arab Republic**	0.01 (0-0.01)	0.01 (0-0.01)	1.07 (1.05-1.08)	-	0 (0-0.01)	-	0 (0-0)	0 (0-0)	1.35 (1.27-1.46)	0 (0-0)	0 (0-0)	1.36 (1.28-1.47)
**Tunisia**	0.03 (0.01-0.05)	0.03 (0.01-0.04)	1.08 (0.7-1.5)	-	0.02 (0.01-0.04)	-	0.02 (0.01-0.03)	0.01 (0-0.01)	4.03 (2.15-6.63)	0.01 (0.01-0.02)	0 (0-0.01)	3.14 (1.82-5.12)
**Turkey**	0.01 (0.01-0.02)	0.01 (0-0.01)	1.74 (1.72-1.75)	-	0.01 (0-0.01)	-	0 (0-0)	0 (0-0)	1.38 (1.3-1.51)	0 (0-0)	0 (0-0)	1.38 (1.31-1.5)
**United Arab Emirates**	0.04 (0.01-0.1)	0.01 (0-0.02)	3.51 (0.77-6.14)	-	0.01 (0-0.02)	-	0.06 (0.01-0.14)	0 (0-0.01)	16.17 (3.59-28.8)	0.02 (0-0.05)	0 (0-0.01)	7.95 (1.99-13.34)
**Yemen**	0.13 (0.02-0.31)	0.06 (0-0.15)	2.98 (1.15-9.77)	-	0.05 (0-0.13)	-	0.02 (0-0.05)	0.02 (0-0.05)	1.46 (0.93-4.44)	0.02 (0-0.05)	0.02 (0-0.05)	1.56 (0.96-4.88)
**South Asia**	0.68 (0.51-0.85)	0.57 (0.42-0.72)	1.2 (1.12-1.28)	-	0.59 (0.44-0.75)	-	0.24 (0.17-0.33)	0.24 (0.16-0.33)	1.01 (0.9-1.11)	0.22 (0.16-0.3)	0.2 (0.14-0.28)	1.07 (0.97-1.18)
**South Asia**	0.68 (0.51-0.85)	0.57 (0.42-0.72)	1.2 (1.12-1.28)	-	0.59 (0.44-0.75)	-	0.24 (0.17-0.33)	0.24 (0.16-0.33)	1.01 (0.9-1.11)	0.22 (0.16-0.3)	0.2 (0.14-0.28)	1.07 (0.97-1.18)
**Bangladesh**	0.07 (0-0.34)	0.05 (0-0.27)	2.55 (0.29-11.03)	-	0.05 (0-0.26)	-	0.02 (0-0.12)	0.02 (0-0.12)	1.97 (0.36-7.98)	0.02 (0-0.11)	0.02 (0-0.1)	2.17 (0.42-8.44)

**Bhutan**	0.14 (0.04-0.28)	0.06 (0.01-0.12)	2.46 (1.38-4.9)	-	0.06 (0.01-0.12)	-	0.03 (0.01-0.05)	0.02 (0-0.04)	1.48 (1.1-2.71)	0.03 (0.01-0.05)	0.02 (0-0.04)	1.5 (1.11-2.79)
**India**	0.82 (0.61-1.02)	0.71 (0.52-0.9)	1.16 (1.09-1.23)	-	0.73 (0.54-0.93)	-	0.28 (0.2-0.38)	0.29 (0.2-0.41)	0.96 (0.87-1.06)	0.26 (0.19-0.34)	0.25 (0.18-0.34)	1.02 (0.93-1.12)
**Nepal**	0.67 (0.06-1.35)	0.18 (0.01-0.41)	4.58 (1.23-12.52)	-	0.18 (0.01-0.4)	-	0.19 (0.02-0.42)	0.07 (0-0.17)	3.56 (1.03-10)	0.18 (0.02-0.39)	0.06 (0-0.15)	3.87 (1.13-10.77)
**Pakistan**	0.14 (0.01-0.31)	0.08 (0-0.17)	2.52 (0.61-8.86)	-	0.12 (0-0.25)	-	0.14 (0.01-0.31)	0.09 (0-0.19)	2.25 (0.49-8.18)	0.09 (0.01-0.19)	0.05 (0-0.11)	2.32 (0.64-7.6)
**Southeast Asia, East Asia, and Oceania**	0.43 (0.32-0.58)	0.36 (0.26-0.46)	1.22 (1.06-1.42)	-	0.35 (0.25-0.45)	-	0.3 (0.21-0.39)	0.08 (0.06-0.11)	3.53 (2.81-4.43)	0.2 (0.14-0.26)	0.08 (0.06-0.11)	2.48 (2.07-2.99)
**East Asia**	0.12 (0.07-0.19)	0.06 (0.04-0.1)	1.91 (1.63-2.13)	-	0.06 (0.04-0.1)	-	0.08 (0.05-0.13)	0.01 (0.01-0.02)	6.09 (5.12-7.37)	0.05 (0.03-0.07)	0.01 (0.01-0.02)	3.99 (3.61-4.39)
**China**	0.12 (0.07-0.19)	0.06 (0.04-0.1)	1.91 (1.64-2.11)	-	0.06 (0.04-0.09)	-	0.08 (0.05-0.12)	0.01 (0.01-0.02)	6.21 (5.29-7.47)	0.05 (0.03-0.07)	0.01 (0.01-0.02)	4.07 (3.73-4.36)
**Democratic People's Republic of Korea**	0.3 (0.02-0.64)	0.18 (0.01-0.4)	1.9 (0.46-5.36)	-	0.18 (0.01-0.39)	-	0.23 (0.02-0.5)	0.05 (0-0.12)	6.23 (1.55-26.87)	0.13 (0.01-0.3)	0.05 (0-0.11)	3.92 (1.21-15.7)
**Taiwan (Province of China)**	0.1 (0.06-0.16)	0.02 (0.01-0.02)	6.15 (5.14-6.93)	-	0.02 (0.01-0.02)	-	0.07 (0.04-0.11)	0 (0-0.01)	19.14 (16.17-23.25)	0.04 (0.02-0.06)	0 (0-0.01)	11.44 (10.05-12.35)
**Oceania**	2.49 (1.33-4.13)	3.09 (1.64-5.13)	0.8 (0.75-0.82)	-	2.59 (1.39-4.37)	-	0.44 (0.21-0.82)	0.39 (0.19-0.68)	1.13 (0.9-1.56)	0.43 (0.2-0.77)	0.34 (0.16-0.6)	1.25 (1-1.69)
**Fiji**	0.14 (0.08-0.21)	0.15 (0.09-0.23)	0.93 (0.93-0.94)	-	0.13 (0.08-0.2)	-	0.04 (0.02-0.06)	0.04 (0.02-0.06)	0.97 (0.92-1.02)	0.04 (0.02-0.06)	0.04 (0.02-0.06)	1 (0.95-1.05)
**Kiribati**	0.42 (0.27-0.56)	0.5 (0.33-0.68)	0.83 (0.78-0.91)	-	0.46 (0.3-0.62)	-	0.18 (0.11-0.24)	0.18 (0.11-0.24)	0.99 (0.92-1.09)	0.18 (0.11-0.24)	0.17 (0.11-0.23)	1.02 (0.96-1.11)
**Marshall Islands**	0.18 (0.01-0.37)	0.22 (0.01-0.46)	1.04 (0.13-3.55)	-	0.2 (0.01-0.42)	-	0.04 (0-0.08)	0.04 (0-0.07)	1.22 (0.28-4.09)	0.04 (0-0.08)	0.03 (0-0.07)	1.27 (0.31-4.05)
**Micronesia (Federated States of)**	2 (0.1-3.54)	1.27 (0.08-2.22)	1.84 (0.27-5.81)	-	1.09 (0.07-1.97)	-	0.32 (0.05-0.59)	0.17 (0.04-0.31)	1.94 (0.65-3.97)	0.31 (0.05-0.56)	0.15 (0.04-0.27)	2.08 (0.71-4.26)
**Nauru**	0.5 (0.07-0.79)	0.27 (0.03-0.45)	2.09 (1.24-6.37)	-	0.24 (0.02-0.39)	-	0.08 (0.01-0.14)	0.04 (0-0.07)	2.34 (1.4-5.72)	0.08 (0.01-0.14)	0.03 (0-0.06)	2.54 (1.52-6.2)

**Palau**	0.38 (0.06-0.59)	0.21 (0.03-0.35)	2.05 (1.29-6.02)	-	0.18 (0.02-0.31)	-	0.06 (0.01-0.11)	0.03 (0-0.05)	2.29 (1.49-6.14)	0.06 (0.01-0.1)	0.03 (0-0.05)	2.49 (1.59-6.74)
**Papua New Guinea**	3.19 (1.7-5.37)	4 (2.11-6.73)	0.8 (0.75-0.81)	-	3.34 (1.77-5.69)	-	0.55 (0.25-1.03)	0.49 (0.23-0.85)	1.14 (0.9-1.58)	0.53 (0.24-0.97)	0.42 (0.2-0.75)	1.27 (1-1.72)
**Samoa**	0.27 (0.01-0.62)	0.32 (0.01-0.71)	1.2 (0.11-4.67)	-	0.29 (0.01-0.66)	-	0.05 (0-0.11)	0.04 (0-0.1)	1.36 (0.26-4.81)	0.05 (0-0.11)	0.04 (0-0.09)	1.42 (0.28-5.09)
**Solomon Islands**	0.26 (0.01-0.58)	0.34 (0.01-0.76)	1.06 (0.12-4.02)	-	0.29 (0.01-0.65)	-	0.05 (0-0.12)	0.05 (0-0.12)	1.22 (0.26-4.17)	0.05 (0-0.12)	0.05 (0-0.11)	1.34 (0.28-4.59)
**Tonga**	0.15 (0.09-0.24)	0.09 (0.05-0.13)	1.78 (1.7-1.97)	-	0.08 (0.05-0.12)	-	0.03 (0.02-0.05)	0.02 (0.01-0.03)	1.75 (1.43-2.4)	0.03 (0.01-0.04)	0.01 (0.01-0.02)	1.81 (1.48-2.51)
**Tuvalu**	0.73 (0.07-1.17)	0.37 (0.03-0.64)	2.47 (1.07-8.52)	-	0.32 (0.02-0.57)	-	0.11 (0.01-0.21)	0.05 (0-0.09)	2.65 (1.25-8.9)	0.11 (0.01-0.2)	0.05 (0-0.09)	2.88 (1.36-9.5)
**Vanuatu**	0.49 (0.01-1.22)	0.57 (0.01-1.41)	1.58 (0.11-8.3)	-	0.53 (0.01-1.34)	-	0.16 (0-0.4)	0.16 (0-0.41)	1.51 (0.14-6.97)	0.14 (0-0.36)	0.13 (0-0.34)	1.65 (0.17-7.68)
**Southeast Asia**	1.1 (0.81-1.43)	0.94 (0.71-1.22)	1.16 (0.99-1.39)	-	0.93 (0.69-1.2)	-	0.79 (0.57-1.01)	0.2 (0.14-0.27)	3.94 (3.08-5.07)	0.5 (0.37-0.64)	0.19 (0.13-0.25)	2.7 (2.21-3.36)
**Cambodia**	2.61 (1.68-3.83)	1.99 (1.3-2.94)	1.32 (1.03-1.68)	-	1.95 (1.24-2.83)	-	1.52 (0.95-2.25)	0.33 (0.19-0.5)	4.74 (3.56-7.04)	1.02 (0.66-1.47)	0.31 (0.18-0.47)	3.4 (2.61-5)
**Indonesia**	0.88 (0.68-1.17)	0.76 (0.59-1.03)	1.17 (0.97-1.41)	-	0.72 (0.54-0.97)	-	0.47 (0.33-0.67)	0.25 (0.16-0.38)	1.91 (1.55-2.54)	0.4 (0.28-0.54)	0.25 (0.16-0.37)	1.62 (1.4-1.98)
**Lao People's Democratic Republic**	2.63 (0.02-14.71)	0.65 (0.01-3.94)	6.08 (0.49-22.3)	-	0.61 (0.01-3.61)	-	0.28 (0-1.44)	0.17 (0-1.01)	5.34 (1.01-31.95)	0.27 (0-1.41)	0.17 (0-1.01)	5.38 (1.02-32.81)
**Malaysia**	0.6 (0.37-0.92)	0.66 (0.41-1)	0.92 (0.87-0.97)	-	0.73 (0.45-1.12)	-	0.8 (0.49-1.21)	0.39 (0.24-0.6)	2.03 (1.93-2.4)	0.46 (0.28-0.7)	0.36 (0.21-0.55)	1.28 (1.22-1.4)
**Maldives**	0.02 (0.01-0.02)	0.11 (0.07-0.15)	0.15 (0.14-0.16)	-	0.1 (0.06-0.14)	-	0.01 (0.01-0.01)	0.01 (0.01-0.02)	0.77 (0.7-0.87)	0.01 (0-0.01)	0.01 (0.01-0.01)	0.7 (0.66-0.75)
**Mauritius**	0.17 (0.1-0.26)	0.05 (0.03-0.07)	3.64 (3.06-4.05)	-	0.04 (0.03-0.07)	-	0.07 (0.04-0.12)	0 (0-0.01)	18.55 (14.4-24.53)	0.05 (0.03-0.07)	0 (0-0.01)	13.81 (12.25-15.62)
**Myanmar**	1.74 (1.41-2.19)	3.63 (2.23-5.29)	0.51 (0.33-0.9)	-	3.53 (2.13-5.1)	-	1.54 (1.21-2.1)	0.58 (0.3-0.88)	2.89 (1.63-5.93)	0.83 (0.68-1.05)	0.48 (0.24-0.73)	1.88 (1.14-3.8)
**Philippines**	0.09 (0.06-0.14)	0.06 (0.04-0.09)	1.51 (1.44-1.55)	-	0.06 (0.04-0.09)	-	0.02 (0.01-0.03)	0.01 (0.01-0.02)	1.63 (1.45-1.97)	0.02 (0.01-0.03)	0.01 (0.01-0.02)	1.55 (1.42-1.74)

**Seychelles**	0.1 (0.06-0.16)	0.25 (0.15-0.37)	0.41 (0.35-0.46)	-	0.26 (0.16-0.4)	-	0.13 (0.08-0.2)	0.05 (0.03-0.08)	2.57 (2.12-2.94)	0.06 (0.03-0.08)	0.04 (0.02-0.06)	1.38 (1.05-1.67)
**Sri Lanka**	0.04 (0.03-0.07)	0.03 (0.02-0.04)	1.68 (1.65-1.78)	-	0.02 (0.01-0.04)	-	0.01 (0-0.01)	0.01 (0-0.01)	1.23 (1.17-1.43)	0.01 (0-0.01)	0.01 (0-0.01)	1.23 (1.17-1.43)
**Thailand**	2.34 (1.43-3.55)	1.76 (1.08-2.67)	1.33 (1.23-1.38)	-	1.76 (1.08-2.69)	-	0.81 (0.48-1.29)	0.15 (0.08-0.23)	5.7 (4.63-7.93)	0.6 (0.37-0.9)	0.14 (0.08-0.21)	4.45 (3.79-6.05)
**Timor-Leste**	0.34 (0.07-0.62)	0.29 (0.05-0.53)	1.29 (0.48-2.8)	-	0.26 (0.05-0.47)	-	0.16 (0.03-0.3)	0.06 (0.01-0.12)	3.24 (1.29-12.52)	0.13 (0.03-0.23)	0.06 (0-0.12)	2.58 (1.18-9.25)
**Viet Nam**	1.43 (0.9-1.95)	0.27 (0.17-0.37)	5.37 (4.9-6.08)	-	0.31 (0.2-0.42)	-	2.03 (1.24-2.75)	0.08 (0.05-0.11)	26.45 (23.16-30.16)	0.77 (0.47-1.04)	0.05 (0.03-0.07)	14.39 (12.66-16.8)
**Sub-Saharan Africa**	21.29 (14.66-27.35)	22.59 (15.59-28.95)	0.94 (0.89-0.99)	-	19.05 (12.94-25.32)	-	2.3 (1.56-3.18)	2.13 (1.47-2.87)	1.08 (1.03-1.17)	2.25 (1.53-3.07)	1.91 (1.32-2.53)	1.17 (1.1-1.28)
**Central Sub-Saharan Africa**	7.67 (6.14-9.58)	13.85 (10.76-17.73)	0.55 (0.51-0.61)	-	11.36 (8.42-14.67)	-	0.85 (0.64-1.11)	0.99 (0.74-1.29)	0.87 (0.81-0.93)	0.84 (0.63-1.09)	0.89 (0.65-1.15)	0.95 (0.88-1.04)
**Angola**	7.99 (4.79-12.72)	16.52 (9.9-26.36)	0.48 (0.48-0.49)	-	13.3 (7.74-21.5)	-	0.77 (0.45-1.24)	0.99 (0.59-1.6)	0.78 (0.74-0.83)	0.76 (0.45-1.22)	0.87 (0.51-1.41)	0.88 (0.81-0.96)
**Central African Republic**	50.21 (39.89-64.92)	73.24 (57.77-96.03)	0.69 (0.56-0.88)	-	61.5 (45.85-83.7)	-	3.57 (2.59-4.93)	3.91 (2.87-5.4)	0.92 (0.8-1.08)	3.5 (2.55-4.81)	3.42 (2.43-4.78)	1.03 (0.88-1.22)
**Congo**	11.22 (6.3-18.34)	19.1 (10.73-31.11)	0.59 (0.58-0.59)	-	15.73 (8.5-25.74)	-	1.6 (0.89-2.61)	1.67 (0.92-2.76)	0.96 (0.92-0.99)	1.59 (0.88-2.58)	1.54 (0.84-2.52)	1.03 (0.98-1.09)
**Democratic Republic of the Congo**	4.58 (3.52-6.03)	8.57 (6.48-11.31)	0.54 (0.45-0.65)	-	7.05 (5.16-9.46)	-	0.67 (0.5-0.87)	0.76 (0.56-0.99)	0.89 (0.81-0.97)	0.66 (0.49-0.87)	0.69 (0.51-0.91)	0.96 (0.88-1.05)
**Equatorial Guinea**	10.08 (5.45-17.2)	13.26 (7.16-22.6)	0.76 (0.76-0.76)	-	10.58 (5.42-18.04)	-	1 (0.54-1.76)	1.04 (0.56-1.82)	0.97 (0.91-1.04)	0.99 (0.53-1.74)	0.93 (0.5-1.62)	1.06 (0.99-1.16)
**Gabon**	9.5 (5.35-15.05)	20.79 (11.68-32.95)	0.46 (0.45-0.46)	-	16.2 (8.93-26.16)	-	1.02 (0.57-1.62)	1.33 (0.75-2.15)	0.76 (0.72-0.81)	1 (0.56-1.6)	1.16 (0.66-1.87)	0.87 (0.8-0.94)
**Eastern Sub-Saharan Africa**	25.25 (17.85-31.25)	25.1 (17.7-31.26)	1.01 (0.91-1.12)	-	20.99 (14.34-26.58)	-	2.65 (1.85-3.46)	2.52 (1.75-3.25)	1.05 (1.02-1.1)	2.63 (1.84-3.43)	2.38 (1.66-3.08)	1.1 (1.06-1.16)
**Burundi**	10.16 (7.95-13.77)	11.1 (8.51-14.57)	0.92 (0.73-1.21)	-	9.46 (7.1-12.62)	-	1.04 (0.71-1.46)	0.95 (0.7-1.24)	1.08 (0.95-1.24)	1.04 (0.71-1.46)	0.93 (0.68-1.2)	1.11 (0.98-1.27)
**Comoros**	0.15 (0-1.33)	0.05 (0-0.4)	4.81 (0.73-17.48)	-	0.04 (0-0.38)	-	0.01 (0-0.07)	0 (0-0.05)	2.4 (1.11-6.66)	0.01 (0-0.07)	0 (0-0.05)	2.43 (1.12-6.81)

**Djibouti**	7.92 (4.61-12.84)	12.38 (7.21-20.06)	0.64 (0.64-0.64)	-	10.18 (5.65-16.71)	-	0.27 (0.15-0.45)	0.29 (0.16-0.47)	0.95 (0.92-0.98)	0.27 (0.15-0.45)	0.28 (0.15-0.45)	0.99 (0.96-1.03)
**Eritrea**	6.4 (3.95-9.16)	10.93 (6.72-15.49)	0.59 (0.54-0.63)	-	9.23 (5.63-13.37)	-	0.5 (0.29-0.76)	0.55 (0.33-0.82)	0.91 (0.86-0.95)	0.5 (0.29-0.76)	0.52 (0.3-0.78)	0.96 (0.91-1.01)
**Ethiopia**	5.54 (3.38-8.06)	8.71 (5.32-12.73)	0.64 (0.62-0.65)	-	7.32 (4.42-10.85)	-	0.76 (0.47-1.14)	0.79 (0.49-1.17)	0.97 (0.94-1)	0.76 (0.47-1.14)	0.76 (0.46-1.14)	1 (0.97-1.04)
**Kenya**	20.76 (12.25-31.13)	21.14 (12.46-31.03)	0.98 (0.93-1.05)	-	18.45 (10.95-27.54)	-	3.32 (1.94-5.11)	2.71 (1.6-4.11)	1.22 (1.14-1.33)	3.07 (1.8-4.67)	2.24 (1.32-3.4)	1.37 (1.25-1.53)
**Madagascar**	0.47 (0.28-0.72)	0.73 (0.44-1.13)	0.64 (0.64-0.64)	-	0.61 (0.36-0.95)	-	0.04 (0.02-0.06)	0.04 (0.03-0.07)	0.93 (0.89-0.96)	0.04 (0.02-0.06)	0.04 (0.02-0.06)	0.97 (0.93-1.02)
**Malawi**	48.08 (37.85-63.41)	49.61 (39.43-60.13)	0.98 (0.69-1.48)	-	40.74 (31.49-51.57)	-	3.71 (2.82-4.89)	3.4 (2.52-4.51)	1.09 (0.99-1.23)	3.7 (2.81-4.87)	3.26 (2.4-4.34)	1.14 (1.03-1.29)
**Mozambique**	123.15 (82.37-163.83)	112.25 (76.03-146.89)	1.1 (0.89-1.44)	-	95.59 (63.02-124.94)	-	12.47 (8.21-16.73)	12.2 (8.02-16.14)	1.02 (0.97-1.09)	12.45 (8.19-16.7)	11.9 (7.85-15.71)	1.05 (0.99-1.11)
**Rwanda**	10.99 (8.01-14.11)	15.8 (11.33-19.53)	0.7 (0.55-0.9)	-	12.92 (8.88-16.54)	-	0.58 (0.42-0.75)	0.61 (0.44-0.77)	0.95 (0.87-1.05)	0.58 (0.41-0.74)	0.56 (0.4-0.71)	1.03 (0.94-1.14)
**Somalia**	6.98 (4.66-9.97)	10.23 (6.88-13.62)	0.68 (0.57-0.81)	-	8.61 (5.75-11.66)	-	0.67 (0.43-1)	0.72 (0.47-1.06)	0.94 (0.87-1)	0.67 (0.43-1)	0.69 (0.45-1.03)	0.98 (0.91-1.04)
**South Sudan**	12.95 (7.63-20.15)	16.54 (9.81-25.79)	0.78 (0.71-0.83)	-	13.83 (8.14-21.61)	-	0.69 (0.39-1.08)	0.72 (0.42-1.15)	0.95 (0.89-1.01)	0.68 (0.39-1.08)	0.68 (0.38-1.08)	1.01 (0.94-1.09)
**Uganda**	35.86 (25.94-48.88)	27.35 (20.87-36.92)	1.34 (0.84-2.06)	-	21.43 (15.61-28.9)	-	2.18 (1.65-2.78)	1.99 (1.48-2.56)	1.1 (1.02-1.18)	2.18 (1.65-2.78)	1.94 (1.44-2.48)	1.13 (1.05-1.21)
**United Republic of Tanzania**	24.35 (14.92-33.67)	19.54 (11.91-26.81)	1.25 (1.07-1.49)	-	16.62 (10.26-23.15)	-	2.08 (1.27-2.96)	1.84 (1.14-2.63)	1.13 (1.04-1.24)	2.07 (1.26-2.94)	1.7 (1.05-2.43)	1.22 (1.12-1.35)
**Zambia**	69.07 (46.04-89.58)	61.66 (40.78-79.44)	1.13 (0.86-1.5)	-	48.28 (31.44-63.16)	-	6.56 (4.33-8.91)	6.21 (4-8.59)	1.06 (0.99-1.14)	6.55 (4.32-8.9)	5.99 (3.87-8.34)	1.09 (1.03-1.19)
**Southern Sub-Saharan Africa**	73.5 (50.35-98.9)	76.46 (53.47-101.62)	0.96 (0.89-1.04)	-	62.9 (42.42-85.54)	-	7.17 (4.96-10)	6.5 (4.77-8.69)	1.1 (1.01-1.24)	7.03 (4.88-9.76)	5.87 (4.27-7.83)	1.19 (1.09-1.38)
**Botswana**	76.49 (43.83-113.4)	65.78 (37.24-100.77)	1.17 (1.02-1.24)	-	51.14 (27.93-81.44)	-	3.92 (2.14-6.38)	2.82 (1.56-4.5)	1.39 (1.23-1.61)	3.81 (2.08-6.15)	2.26 (1.23-3.62)	1.69 (1.43-2.03)
**Eswatini**	167.43 (127.42-223.2)	136.56 (108.38-159.79)	1.24 (0.9-1.76)	-	113.08 (85.69-135.49)	-	13.98 (10.97-17.79)	11.64 (8.93-14.14)	1.21 (1.01-1.5)	13.74 (10.76-17.37)	10.7 (8.08-12.95)	1.29 (1.09-1.61)

**Lesotho**	251.7 (198.14-325.16)	260.32 (200.86-337.56)	0.99 (0.7-1.48)	-	209.42 (154.53-278.53)	-	20.49 (16.72-25.5)	18.94 (14.79-23.92)	1.09 (0.91-1.38)	20.13 (16.46-24.84)	16.96 (13.14-21.22)	1.2 (1-1.51)
**Namibia**	54.93 (32.22-80.75)	53.82 (31.15-80.32)	1.02 (0.88-1.09)	-	46.21 (26.43-69.77)	-	5.49 (3.16-8.34)	4.83 (2.79-7.32)	1.14 (1.05-1.24)	5.41 (3.13-8.16)	4.52 (2.62-6.85)	1.2 (1.1-1.32)
**South Africa**	67 (39.12-97.76)	69.57 (40.45-100.86)	0.96 (0.91-1.03)	-	57.16 (32.86-84.23)	-	6.42 (3.72-9.95)	5.38 (3.14-8.25)	1.19 (1.09-1.36)	6.27 (3.63-9.58)	4.77 (2.74-7.14)	1.32 (1.18-1.53)
**Zimbabwe**	74.69 (60.64-96.61)	83.74 (69.55-98.9)	0.9 (0.66-1.33)	-	69.42 (55.92-84.74)	-	7.73 (6.4-9.51)	8.32 (6.87-9.93)	0.93 (0.82-1.12)	7.62 (6.33-9.37)	7.75 (6.41-9.17)	0.99 (0.87-1.18)
**Western Sub-Saharan Africa**	9.16 (5.77-13.07)	10.07 (6.26-14.57)	0.91 (0.87-0.94)	-	9.25 (5.7-13.49)	-	1.56 (0.97-2.36)	1.38 (0.87-2.1)	1.13 (1.05-1.24)	1.42 (0.91-2.12)	1.16 (0.73-1.73)	1.23 (1.13-1.36)
**Benin**	5.75 (3.36-8.65)	4.96 (2.9-7.57)	1.16 (1.12-1.18)	-	4.37 (2.52-6.67)	-	0.69 (0.41-1.07)	0.6 (0.35-0.92)	1.16 (1.1-1.24)	0.68 (0.4-1.05)	0.55 (0.32-0.85)	1.22 (1.15-1.32)
**Burkina Faso**	8.63 (6.59-10.97)	6.3 (4.73-8.15)	1.38 (1.12-1.78)	-	5.9 (4.37-7.71)	-	1.05 (0.78-1.33)	0.83 (0.61-1.07)	1.26 (1.13-1.44)	1.02 (0.77-1.29)	0.8 (0.59-1.02)	1.29 (1.16-1.47)
**Cabo Verde**	1.27 (0.76-2.06)	0.97 (0.59-1.56)	1.31 (1.3-1.37)	-	0.86 (0.51-1.39)	-	0.17 (0.1-0.28)	0.15 (0.09-0.24)	1.15 (1.1-1.22)	0.17 (0.1-0.28)	0.14 (0.08-0.23)	1.19 (1.14-1.27)
**Cameroon**	17.86 (9.56-29.13)	22.16 (12-36.03)	0.81 (0.81-0.81)	-	17.61 (9.25-28.96)	-	0.99 (0.53-1.62)	1.03 (0.55-1.69)	0.96 (0.93-0.99)	0.98 (0.53-1.61)	0.93 (0.49-1.53)	1.06 (1-1.12)
**Chad**	20.04 (14.32-27.66)	17.41 (11.75-23.9)	1.16 (0.88-1.55)	-	15.62 (10.33-21.79)	-	1.94 (1.35-2.63)	1.66 (1.12-2.28)	1.18 (1.01-1.41)	1.89 (1.32-2.54)	1.52 (1.03-2.1)	1.25 (1.07-1.49)
**Côte d'Ivoire**	12.7 (7.12-20.15)	14.37 (8.05-22.99)	0.88 (0.88-0.89)	-	12.35 (6.86-19.85)	-	1.58 (0.9-2.53)	1.5 (0.86-2.43)	1.05 (1.01-1.1)	1.54 (0.88-2.49)	1.37 (0.77-2.2)	1.13 (1.07-1.21)
**Gambia**	11.23 (6.56-17.5)	10.32 (6.03-16.19)	1.09 (1.08-1.09)	-	9.39 (5.43-14.86)	-	0.91 (0.52-1.49)	0.76 (0.43-1.23)	1.21 (1.12-1.32)	0.88 (0.5-1.43)	0.68 (0.39-1.1)	1.29 (1.18-1.43)
**Ghana**	11.5 (6.76-17.69)	16.03 (9.41-24.63)	0.72 (0.7-0.72)	-	13.23 (7.57-21)	-	1.07 (0.63-1.65)	1.21 (0.72-1.88)	0.88 (0.84-0.91)	1.04 (0.61-1.6)	1.03 (0.6-1.61)	1.01 (0.93-1.1)
**Guinea**	14.55 (9.2-20.14)	17.12 (10.79-23.9)	0.85 (0.73-1.02)	-	14.91 (9.29-20.78)	-	1.37 (0.85-1.94)	1.36 (0.85-1.94)	1.01 (0.9-1.12)	1.33 (0.83-1.88)	1.21 (0.76-1.7)	1.1 (0.98-1.25)
**Guinea-Bissau**	35.42 (21.21-52.07)	23.96 (14.4-35.55)	1.49 (1.07-2.01)	-	20.94 (12.6-30.99)	-	2.99 (1.81-4.55)	2.05 (1.22-3.05)	1.46 (1.17-1.86)	2.89 (1.76-4.33)	1.84 (1.08-2.73)	1.58 (1.27-2.01)
**Liberia**	3.06 (1.76-4.84)	2.77 (1.6-4.38)	1.1 (1.09-1.11)	-	2.3 (1.31-3.73)	-	0.25 (0.14-0.42)	0.21 (0.12-0.34)	1.21 (1.12-1.33)	0.24 (0.14-0.4)	0.18 (0.1-0.29)	1.37 (1.24-1.54)

**Mali**	6.85 (4.06-10.46)	8.18 (4.87-12.56)	0.84 (0.81-0.85)	-	7.39 (4.36-11.32)	-	0.69 (0.41-1.08)	0.7 (0.42-1.08)	0.98 (0.93-1.06)	0.67 (0.4-1.04)	0.64 (0.38-0.99)	1.05 (0.98-1.14)
**Mauritania**	0.31 (0.01-2.43)	0.07 (0-0.4)	4.69 (0.99-15.4)	-	0.06 (0-0.35)	-	0.02 (0-0.16)	0.01 (0-0.04)	4.53 (1.32-13.79)	0.02 (0-0.15)	0.01 (0-0.04)	5.08 (1.43-15.66)
**Niger**	4.69 (3.24-6.41)	3.91 (2.55-5.35)	1.21 (0.93-1.74)	-	3.44 (2.21-4.74)	-	0.39 (0.27-0.53)	0.31 (0.2-0.42)	1.28 (1.06-1.67)	0.38 (0.26-0.51)	0.27 (0.18-0.38)	1.39 (1.15-1.81)
**Nigeria**	7.62 (4.84-11.09)	8.4 (5.22-12.46)	0.91 (0.83-0.96)	-	8.57 (5.28-12.91)	-	2.25 (1.39-3.59)	1.87 (1.15-3.01)	1.2 (1.1-1.35)	1.84 (1.15-2.82)	1.47 (0.92-2.29)	1.25 (1.15-1.37)
**Sao Tome and Principe**	0.11 (0.06-0.18)	0.03 (0.02-0.05)	3.66 (2.54-4.98)	-	0.03 (0.01-0.05)	-	0.01 (0-0.03)	0 (0-0.01)	2.96 (2-4.46)	0.01 (0-0.03)	0 (0-0.01)	3.13 (2.02-4.82)
**Senegal**	2.67 (1.58-4.09)	3.9 (2.31-5.89)	0.69 (0.67-0.69)	-	3.42 (2.02-5.22)	-	0.28 (0.17-0.43)	0.31 (0.19-0.48)	0.9 (0.86-0.96)	0.27 (0.16-0.42)	0.28 (0.17-0.43)	0.99 (0.92-1.08)
**Sierra Leone**	6.58 (3.8-10.47)	5.98 (3.45-9.51)	1.1 (1.1-1.1)	-	4.88 (2.81-7.73)	-	0.45 (0.26-0.73)	0.44 (0.26-0.72)	1.02 (0.95-1.09)	0.45 (0.26-0.72)	0.39 (0.22-0.63)	1.16 (1.05-1.27)
**Togo**	8.55 (4.79-13.84)	8.62 (4.84-13.9)	0.99 (0.98-1)	-	7.81 (4.4-12.84)	-	0.83 (0.47-1.37)	0.75 (0.43-1.22)	1.1 (1.04-1.19)	0.81 (0.45-1.32)	0.69 (0.39-1.11)	1.17 (1.1-1.28)

References

1. Vos T, Lim SS, Abbafati C, Abbas KM, Abbasi M, Abbasifard M, et al. Global burden of 369 diseases and injuries in 204 countries and territories, 1990–2019: a systematic analysis for the Global Burden of Disease Study 2019. Lancet [Internet]. 2020 Oct;396(10258):1204–22. Available from: https://linkinghub.elsevier.com/retrieve/pii/S0140673620309259
2. Murray CJL, Aravkin AY, Zheng P, Abbafati C, Abbas KM, Abbasi-Kangevari M, et al. Global burden of 87 risk factors in 204 countries and territories, 1990–2019: a systematic analysis for the Global Burden of Disease Study 2019. Lancet [Internet]. 2020 Oct;396(10258):1223–49. Available from: https://linkinghub.elsevier.com/retrieve/pii/S0140673620307522
3. Kyu HH, Maddison ER, Henry NJ, Ledesma JR, Wiens KE, Reiner R, et al. Global, regional, and national burden of tuberculosis, 1990–2016: results from the Global Burden of Diseases, Injuries, and Risk Factors 2016 Study. Lancet Infect Dis [Internet]. 2018 Dec;18(12):1329–49. Available from: https://linkinghub.elsevier.com/retrieve/pii/S147330991830625X
4. Kyu HH, Maddison ER, Henry NJ, Mumford JE, Barber R, Shields C, et al. The global burden of tuberculosis: results from the Global Burden of Disease Study 2015. Lancet Infect Dis [Internet]. 2018 Mar;18(3):261–84. Available from: https://linkinghub.elsevier.com/retrieve/pii/S147330991730703X
5. Naghavi M, Makela S, Foreman K, O'Brien J, Pourmalek F, Lozano R. Algorithms for enhancing public health utility of national causes-of-death data. Popul Health Metr [Internet]. 2010 Dec 10;8(1):9. Available from: https://pophealthmetrics.biomedcentral.com/articles/10.1186/1478-7954-8-9
6. Adegbola RA, Falade AG, Sam BE, Aidoo M, Baldeh I, Hazlett D, et al. The etiology of pneumonia in malnourished and well-nourished Gambian children. Pediatr Infect Dis J [Internet]. 1994 Nov;13(11):975–82. Available from: http://www.ncbi.nlm.nih.gov/pubmed/7845751
7. Chisti MJ, Graham SM, Duke T, Ahmed T, Ashraf H, Faruque ASG, et al. A prospective study of the prevalence of tuberculosis and bacteraemia in Bangladeshi children with severe malnutrition and pneumonia including an evaluation of Xpert MTB/RIF assay. PLoS One [Internet]. 2014;9(4):e93776. Available from: http://www.ncbi.nlm.nih.gov/pubmed/24695758
8. Madhi SA, Petersen K, Madhi A, Khoosal M, Klugman KP. Increased disease burden and antibiotic resistance of bacteria causing severe community-acquired lower respiratory tract infections in human immunodeficiency virus type 1-infected children. Clin Infect Dis [Internet]. 2000 Jul;31(1):170–6. Available from: http://www.ncbi.nlm.nih.gov/pubmed/10913417
9. McNally LM, Jeena PM, Gajee K, Thula SA, Sturm AW, Cassol S, et al. Effect of age, polymicrobial disease, and maternal HIV status on treatment response and cause of severe pneumonia in South African children: a prospective descriptive study. Lancet [Internet]. 2007 Apr;369(9571):1440–51. Available from: https://linkinghub.elsevier.com/retrieve/pii/S0140673607606709
10. Moore DP, Klugman KP, Madhi SA. Role of streptococcus pneumoniae in hospitalization for acute community-acquired pneumonia associated with culture-confirmed

mycobacterium tuberculosis in Children. Pediatr Infect Dis J [Internet]. 2010 Dec;29(12):1099–104. Available from: http://journals.lww.com/00006454-201012000-00009
11. Nantongo JM, Wobudeya E, Mupere E, Joloba M, Ssengooba W, Kisembo HN, et al. High incidence of pulmonary tuberculosis in children admitted with severe pneumonia in Uganda. BMC Pediatr [Internet]. 2013 Dec 31;13(1):16. Available from: http://bmcpediatr.biomedcentral.com/articles/10.1186/1471-2431-13-16
12. Zar HJ, Hanslo D, Tannenbaum E, Klein M, Argent A, Eley B, et al. Aetiology and outcome of pneumonia in human immunodeficiency virus-infected children hospitalized in South Africa. Acta Paediatr [Internet]. 2001 Feb;90(2):119–25. Available from: http://www.ncbi.nlm.nih.gov/pubmed/11236037
13. Moore DP, Higdon MM, Hammitt LL, Prosperi C, DeLuca AN, Da Silva P, et al. The incremental value of repeated induced sputum and gastric aspirate samples for the diagnosis of pulmonary tuberculosis in young children with acute community-acquired pneumonia. Clin Infect Dis [Internet]. 2017 Jun 15;64(suppl_3):S309–16. Available from: http://academic.oup.com/cid/article/64/suppl_3/S309/3858220/The-Incremental-Value-of-Repeated-Induced-Sputum
14. Foreman KJ, Lozano R, Lopez AD, Murray CJ. Modeling causes of death: an integrated approach using CODEm. Popul Health Metr [Internet]. 2012 Dec 6;10(1):1. Available from: http://pophealthmetrics.biomedcentral.com/articles/10.1186/1478-7954-10-1
15. Cox JA, Lukande RL, Lucas S, Nelson AM, Van Marck E, Colebunders R. Autopsy causes of death in HIV-positive individuals in sub-Saharan Africa and correlation with clinical diagnoses. AIDS Rev [Internet]. 2010;12(4):183–94. Available from: http://www.ncbi.nlm.nih.gov/pubmed/21179183
16. Ford N, Matteelli A, Shubber Z, Hermans S, Meintjes G, Grinsztejn B, et al. TB as a cause of hospitalization and in-hospital mortality among people living with HIV worldwide: a systematic review and meta-analysis. J Int AIDS Soc [Internet]. 2016 Jan;19(1):20714. Available from: http://doi.wiley.com/10.7448/IAS.19.1.20714
17. Zheng P, Aravkin AY, Barber R, Sorensen RJD, Murray CJL. Trimmed constrained mixed effects models: formulations and algorithms. 2019 Sep 23; Available from: http://arxiv.org/abs/1909.10700
18. Fullman N, Yearwood J, Abay SM, Abbafati C, Abd-Allah F, Abdela J, et al. Measuring performance on the Healthcare Access and Quality Index for 195 countries and territories and selected subnational locations: a systematic analysis from the Global Burden of Disease Study 2016. Lancet [Internet]. 2018 Jun;391(10136):2236–71. Available from: https://linkinghub.elsevier.com/retrieve/pii/S0140673618309942
19. National Tuberculosis Institute. Tuberculosis in a rural population of South India: a five-year epidemiological study. Bull World Health Organ [Internet]. 1974;51(5):473–88. Available from: http://www.ncbi.nlm.nih.gov/pubmed/4549498
20. Japan International Cooperation Agency, National Center for Tuberculosis and Leprosy Control (CENAT) (Cambodia) RI of TA-TA (RIT/JATA). Cambodia National Tuberculosis Prevalence Survey 2002.
21. Japan International Cooperation Agency, Ministry of Health (Cambodia), National Center for Tuberculosis and Leprosy Control (CENAT) (Cambodia), Research Institute of Tuberculosis/Japan Anti-Tuberculosis Association (RIT/JATA) WHO (WHO). Cambodia National Tuberculosis Prevalence Survey 2010-2011.

22. Ministry of Health (Myanmar), Research Institute of Tuberculosis/Japan Anti-Tuberculosis Association (RIT/JATA) WHO (WHO). Myanmar National Tuberculosis Prevalence Survey 2009-2010.
23. Hong YP, Kim SJ, Lew WJ, Lee EK, Han YC. The seventh nationwide tuberculosis prevalence survey in Korea, 1995. Int J Tuberc Lung Dis [Internet]. 1998 Jan;2(1):27–36. Available from: http://www.ncbi.nlm.nih.gov/pubmed/9562108
24. Centers for Disease Control and Prevention (CDC), KNCV Tuberculosis Foundation, Ministry of Health (Rwanda), National TB Control Program (Rwanda), Rwanda Biomedical Center (RBC), University Teaching Hospital of Kigali (CHUK) WHO (WHO). Rwanda National Tuberculosis Prevalence Survey 2012.
25. Datta M, Radhamani MP, Sadacharam K, Selvaraj R, Rao DL, Rao RS, et al. Survey for tuberculosis in a tribal population in North Arcot District. Int J Tuberc Lung Dis [Internet]. 2001 Mar;5(3):240–9. Available from: http://www.ncbi.nlm.nih.gov/pubmed/11326823
26. National Leprosy and Tuberculosis Control Program (NTLP) (The Gambia) MRC (Gambia). Gambia National Tuberculosis Prevalence Survey 2011-2013 (GAMSTEP).
27. Berhe G, Enqueselassie F, Hailu E, Mekonnen W, Teklu T, Gebretsadik A, et al. Population-based prevalence survey of tuberculosis in the Tigray region of Ethiopia. BMC Infect Dis [Internet]. 2013 Dec 28;13(1):448. Available from: https://bmcinfectdis.biomedcentral.com/articles/10.1186/1471-2334-13-448
28. Chinese Center for Disease Control and Prevention (CCDC), Ministry of Health (China) WHO (WHO). China National Tuberculosis Prevalence Survey 2010.
29. Chinese Center for Disease Control and Prevention. China National Tuberculosis Prevalence Survey 2000.
30. Ministry of Public Health (China), National Tuberculosis Control and Research Center, Beijing (China), National Tuberculosis Control and Research Subcenter S (China). China National Tuberculosis Prevalence Survey 1984-1985.
31. Ethiopian Health and Nutrition Research Center (EHNRI), Ministry of Health (Ethiopia) WHO (WHO). Ethiopia Tuberculosis Prevalence Survey 2010-2011.
32. (China) M of PH. China National Tuberculosis Prevalence Survey 1990.
33. Tupasi TE, Radhakrishna S, Chua JA, Mangubat N V, Guilatco R, Galipot M, et al. Significant decline in the tuberculosis burden in the Philippines ten years after initiating DOTS. Int J Tuberc Lung Dis [Internet]. 2009 Oct;13(10):1224–30. Available from: http://www.ncbi.nlm.nih.gov/pubmed/19793426
34. Hoa NB, Sy DN, Nhung NV, Tiemersma EW, Borgdorff MW, Cobelens FG. National survey of tuberculosis prevalence in Viet Nam. Bull World Health Organ [Internet]. 2010 Apr 1;88(4):273–80. Available from: http://www.who.int/bulletin/volumes/88/4/09-067801.pdf
35. Central TB Division, Directorate General of Health Services (India), Ministry of Health and Family Welfare (India). Delhi, India: Central TB Division DG of HS (India). India Revised National Tuberculosis Control Program Annual Report 2013. 2013.
36. Health and Family Welfare Department Government of Gujarat. Population based survey for assessing prevalence of pulmonary tuberculosis cases in the state of Gujarat, India (2011-2012). 2013.
37. Gothi G, Narayan R, Nair S, Chakraborty A, Srikantaramu N. Estimation of prevalence of bacillary tuberculosis on the basis of chest X-ray and/or symptomatic screening. Indian J

Med Res. 1976;64(8):1150–9.
38. Chadha VK, Kumar P, Anjinappa SM, Singh S, Narasimhaiah S, Joshi M V., et al. Prevalence of Pulmonary Tuberculosis among Adults in a Rural Sub-District of South India. Pai M, editor. PLoS One [Internet]. 2012 Aug 15;7(8):e42625. Available from: https://dx.plos.org/10.1371/journal.pone.0042625
39. Pg G, Sadacharam K, Narayanan P. Yield of pulmonary tuberculosis cases by employing two screening methods in a community survey. Int J Tuberc Lung Dis. 2006 Apr 1;10:343–5.
40. Datta M, Pg G, Appegowda B, Rao K, Gopalan B. Tuberculosis in north Arcot district of Tamil Nadu – a sample survey. Indian J Tuberc. 2000 Jan 1;47.
41. Tiemersma EW, van der Werf MJ, Borgdorff MW, Williams BG, Nagelkerke NJD. Natural history of tuberculosis: duration and fatality of untreated pulmonary tuberculosis in HIV negative patients: a systematic review. Pai M, editor. PLoS One [Internet]. 2011 Apr 4;6(4):e17601. Available from: https://dx.plos.org/10.1371/journal.pone.0017601

SDI Analysis[1]

The Socio-demographic Index (SDI) is a composite indicator of background social and economic conditions that influence health outcomes in each location. In short, it is the geometric mean of 0 to 1 indices of total fertility rate (TFR) for those younger than 25 years old (TFU25), mean education for those 15 years old and older (EDU15+), and lag-distributed income (LDI) per capita. For GBD 2019, after calculating SDI, values were multiplied by 100 for a scale of 0 to 100.

Development of revised SDI indicator [1]

SDI was originally constructed for GBD 2015 by using the Human Development Index (HDI) methodology, wherein a 0 to 1 index value was determined for each of the original three covariate inputs (TFR in ages 15 to 49 years, EDU15+, and LDI per capita) by using the observed minima and maxima over the estimation period to set the scales.

In response to feedback from collaborators and the evolution of the GBD, we have refined the indicator with each GBD cycle. Beginning in GBD 2017, along with our expanded estimation of age-specific fertility, we replaced TFR with TFU25 as one of the three component indices. The TFU25 provides a better measure of women's status in society because it focuses on ages at which childbearing disrupts the pursuit of education and entrance into the workforce. In addition, we observed that in highly developed countries, the TFU25 has tended to decline consistently over time despite rebounds in TFR driven by increasing fertility at older ages. The concordance correlation coefficient between SDI based on the GBD 2016 method and the updated method for GBD 2017 was 0.981.

During GBD 2016, we moved from using relative index scales to using absolute scales to enhance the stability of SDI interpretation over time because we noticed that the measure was highly sensitive to the addition of subnational units that tended to stretch the empirical minima and maxima.[21] We selected the minima and maxima of the scales by examining the relationships each of the inputs had with life expectancy at birth and under-5 mortality and by identifying points of limiting returns at both high and low values if they occurred before theoretical limits (eg, a TFU25 of 0) were reached.

Thus, for each covariate input, an index score of 0 represents the minimum level of each covariate input past which selected health outcomes can get no worse, and an index score of 1 represents the maximum level of each covariate input past which selected health outcomes cease to improve. As a composite, a location with an SDI of 0 would have a theoretical minimum level of sociodemographic development relevant to these health outcomes, and a location with an SDI of 1 (before multiplying by 100 for reporting) would have a theoretical maximum level of sociodemographic development relevant to these health outcomes.
We computed the index scores underlying SDI as follows:

$$I_{Cly} = \max\left(\frac{C_{ly} - C_{low}}{C_{high} - C_{low}}, 0.005\right)$$

Where:

I_{cly} is the index for covariate *C*, location *l*, and year *y* and is equal to the difference between the value of that covariate in that location-year and the lower bound of the covariate divided by the difference between the upper and lower bounds for that covariate

If the values of input covariates fell outside the upper or lower bounds, they were mapped to the respective upper or lower bounds. We also note that the index value for TFU25 was computed as $1 - I_{TFU25ly}$ because lower TFU25s correspond to higher levels of development and thus higher index scores. For GBD 2019, we expanded the computation of SDI to 1062 national and subnational locations spanning the time period 1950–2019.

The composite SDI is the geometric mean of these three indices for a given location-year. The cut-off values used to determine quintiles for analysis were then computed by using country-level estimates of SDI for the year 2019, excluding countries with populations less than 1 million.
For GBD 2019, final SDI values were multiplied by 100 for reporting, in order to improve understanding of and broader engagement with the values. As such, GBD 2019 SDI is calculated as it was in 2017, but multiplied by 100 at the end (see example calculation below). Final reporting values are on a 0 to 100 scale.

Example calculation
We present the equation used to calculate SDI for a hypothetical country in the year 2010:

$$TFU25 = 1.09;\ Mean\ educ\ yrs\ pc = 8.23;\ lnLDI = 9.60$$

$$I_{TFU25} = 1 - \frac{1.09 - 0}{3 - 0} = 0.637$$

$$I_{Educ} = \frac{8.23 - 0}{17 - 0} = 0.484$$

$$I_{lnLDI} = \frac{9.60 - 5.52}{11.00 - 5.52} = 0.744$$

$$SDI = \sqrt[3]{I_{TFU25} * I_{Educ} * I_{lnLDI}} = \sqrt[3]{.637 * .484 * .744} = 0.611$$

$$I_{lnLDI} = \frac{9.58 - 5.52}{11.00 - 5.52} = 0.741$$

$$SDI = \sqrt[3]{I_{TFR} * I_{Educ} * I_{lnLDI}} = \sqrt[3]{.855 * .543 * .741} = 0.701$$

$$\text{GBD 2019 reporting } SDI = 0.701 * 100 = 70.1$$

Alcohol use[2]

Flowchart

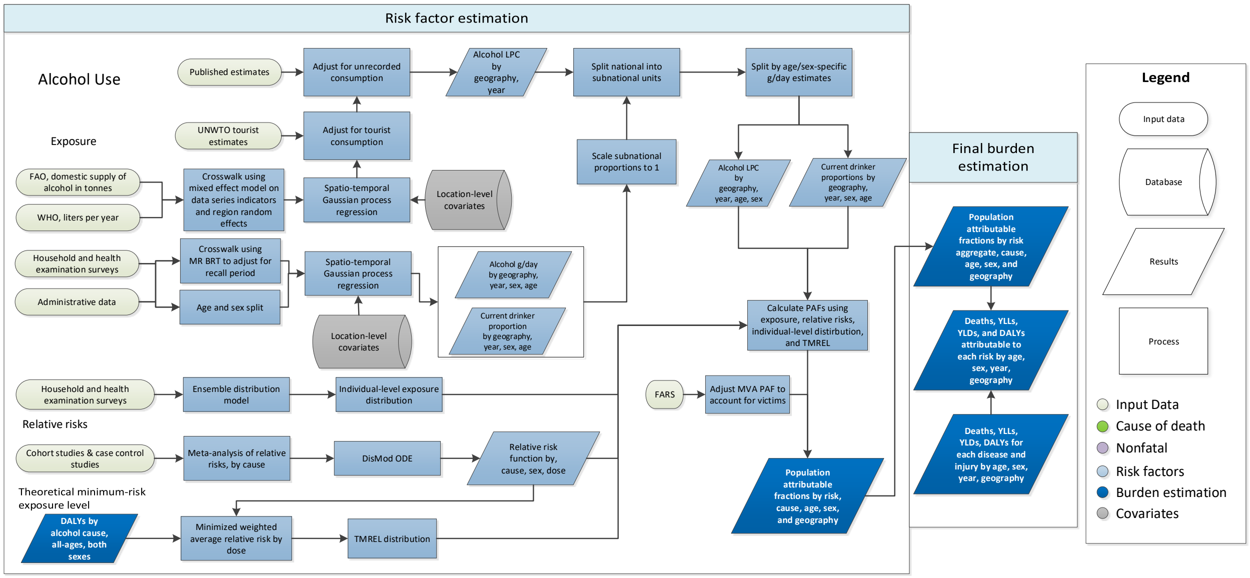

Input data and methodological summary

Definition

Exposure

We defined exposure as the grams per day of pure alcohol consumed among current drinkers. We constructed this exposure using the indicators outlined below:

1. Current drinkers, defined as the proportion of individuals who have consumed at least one alcoholic beverage (or some approximation) in a 12-month period.
2. Alcohol consumption (in grams per day), defined as grams of alcohol consumed by current drinkers, per day, over a 12-month period.
3. Alcohol litres per capita stock, defined in litres per capita of pure alcohol, over a 12-month period.

We also used three additional indicators to adjust alcohol exposure estimates to account for different types of bias:

1. Number of tourists within a location, defined as the total amount of visitors to a location within a 12-month period.
2. Tourists' duration of stay, defined as the number of days resided in a hosting country.
3. Unrecorded alcohol stock, defined as a percentage of the total alcohol stock produced outside established markets.

Input data

A systematic review of the literature was performed to extract data on our primary indicators. The Global Health Exchange (GHDx), IHME's online database of health-related data, was searched for

population survey data containing participant-level information from which we could formulate the required alcohol use indicators on current drinkers and alcohol consumption. Data sources were included if they captured a sample representative of the geographical location under study. We documented relevant survey variables from each data source in a spreadsheet and extracted using STATA 13.1 and R 3.3. A total of 6172 potential data sources were available in the GHDx, of which 5091 have been screened and 1125 accepted.

Table 1: Data inputs for exposure for alcohol use.

Input data	Exposure	Relative risk
Sources (total)	10513	495
Number of countries with data	199	-

Estimates of current drinking prevalence were split by age and sex where necessary. First, studies that reported prevalence for both sexes were split using a region-specific sex ratio estimated using MR-BRT. Second, where studies reported estimates across non-GBD age groups, these were split into standard five-year age groups using the global age pattern estimated by ST-GPR.

Table 2: MR-BRT sex splitting adjustment factors for current drinking

Data input	Gamma	Beta coefficient, log (95% CI)	Adjustment factor*
Female: Male	0	-0.16 (-0.17, -0.14)	0.85
Age < 50	0	0.06 (0.06, 0.06)	1.07
East Asia	0.36	-1.02 (-1.74, -0.29)	0.36
Southeast Asia	0.64	-1.06 (-2.34, 0.22)	0.35
Central Asia	0.41	-0.35 (-1.16, 0.46)	0.70
Central Europe	0.18	-0.21 (-0.58, 0.14)	0.80
Eastern Europe	0.10	-0.07 (-0.28, 0.14)	0.93
High-income Asia Pacific	1.27	-1.11 (-4.90, 2.68)	0.33
Western Europe	0.08	0.03 (-0.14, 0.20)	1.03
Southern Latin America	1.26	-0.67 (-4.18, 2.84)	0.51
High-income North America	0.09	-0.07 (-0.26, 0.11)	0.93
Caribbean	0.25	-0.52 (-1.02, -0.03)	0.59
Andean Latin America	0.76	-0.16 (-1.66, 1.34)	0.85
Central Latin America	0.30	-0.52 (-1.12, 0.08)	0.59
Tropical Latin America	0.08	-0.61 (-0.79, -0.44)	0.54
North Africa and Middle East	1.21	-1.44 (-3.91, 1.03)	0.24
South Asia	0.71	-1.17 (-2.57, 0.23)	0.31
Eastern sub-Saharan Africa	0.28	-0.53 (-1.10, 0.03)	0.58
Southern sub-Saharan Africa	0.20	-0.16 (-0.56, 0.23)	0.85
Western sub-Saharan Africa	0.32	-0.19 (-0.83, 0.45)	0.83
Oceania	0.94	-0.54 (-2.42, 1.34)	0.58

**Adjustment factor is the transformed beta coefficient in normal space and can be interpreted as the factor by which the alternative case definition is adjusted to reflect the ratio by which both-sex data points were split.*

To allow for the inclusion of data that did not meet our reference definition for current drinking, two crosswalks were performed using MR-BRT. The first crosswalk converted estimates of one-month drinking prevalence to what they would be if data represented estimates of 12-month drinking prevalence. This crosswalk incorporated two binary covariates: male and age ≥ 50. The second crosswalk converted estimates of one-week drinking prevalence to 12-month drinking prevalence. This crosswalk incorporated age < 20 and male as covariates. The covariates utilised in both crosswalks were included as both x and z covariates. A uniform prior of 0 was set as the upper bound for the beta coefficients to enforce the logical constraint that one-month and one-week prevalence could not be greater than 12-month prevalence.

Table 3: MR-BRT crosswalk adjustment factors for alcohol use current drinking model

Data input	Reference or alternative case definition	Gamma	Beta coefficient, logit (95% CI)
12-month prevalence	Ref	---	---
1-month prevalence	Alt	0.22	-0.60 (-1.05, -0.16)
Age ≥ 50		0.13	0.16 (-0.10, 0.43)
Male		0.29	0.01 (-0.57, 0.59)
1-week prevalence	Alt	0.46	-1.51 (-2.42, -0.59)
Age < 20		0.47	-0.29 (-1.34, 0.76)
Male		0.00	0.38 (0.15, 0.60)

The methods for modelling supply-side-level data were changed substantially from those used in GBD 2017. The raw data are domestic supply (WHO GISAH; FAO) and retail supply (Euromonitor) of litres of pure ethanol consumed. Domestic supply is calculated as the sum of production and imports, subtracting exports. The WHO and FAO sources were combined, so that FAO data were only used if there were no data available for that location-year from WHO. This was done because the WHO source takes into consideration FAO values when available. Since the WHO data are given in more granular alcohol types, the following adjustments were made:

$$LPC\ Pure\ Ethanol = 0.13 * \left(\frac{Wine}{0.973}\right)$$

$$LPC\ Pure\ Ethanol = 0.05 * \left(\frac{Beer}{0.989}\right)$$

$$LPC\ Pure\ Ethanol = 0.4 * \left(\frac{Spirits}{0.91}\right)$$

Three outliering strategies are used to omit implausible datapoints and data that created implausible model fluctuations. First, estimates from the current drinking model are used to calculate the grams of alcohol consumed per drinker per day. A point is outliered if the grams of pure ethanol per drinker per day for a given source-location-year is greater than 100 (approximately ten drinks). These thresholds

were chosen by using expert knowledge about reasonable consumption levels. In the second round of outliering, the mean liters per capita value over a ten-year window is calculated. If a point is over 70% of that mean value away from the mean value, it is outliered. The 70% limit was chosen using histograms of these distances. Additionally, some manual outliering is performed to account for edge cases. Finally, data smoothing is performed by taking a three-year rolling mean over each location-year.

Next, an imputation to fill in missing years is performed for all series to remove compositional bias from our final estimates. Since the data from our main sources cover different time periods, by imputing a complete time series for each data series, we reduce the probability that compositional bias of the sources is leading to biased final estimates. To impute the missing years for each series, we model the log ratio of each pair of sources as a function of an intercept and nested random effects on super-region, region, and location. The appropriate predicted ratio is multiplied by the source that we do have, which generates an estimated value for the missing source. For some locations where there was limited overlap between series, the predicted ratio did not make sense, and a regional ratio was used.

Finally, variance was calculated both across series (within a location-year) as well as across years (within a location-source). Additionally, if a location-year had one imputed point, the variance was multiplied by 2. If a location-year had two imputed points, the variance was multiplied by 4. The average estimates in each location-year were the input to an ST-GPR model. This uses a mixed-effects model modelled in log space with nested location random effects.

We obtained data on the number of tourists and their duration of stay from the UNWTO.[3] We applied a crosswalk across different tourist categories, similar to the one used for the litres per capita data, to arrive at a consistent definition (ie, visitors to a country).

We obtained estimates on unrecorded alcohol stock from data available in WHO GISAH database,[2] consisting of 189 locations. For locations with no data available, the national or regional average was used.

For relative risks, in GBD 2016 we performed a systematic literature review of all cohort and case-control studies reporting a relative risk, hazard ratio, or odds ratio for any risk-outcome pairs studied in GBD 2016. Studies were included if they reported a categorical or continuous dose for alcohol consumption, as well as uncertainty measures for their outcomes, and the population under study was representative.

Modelling strategy

While population-based surveys provide accurate estimates of the prevalence of current drinkers, they typically underestimate real alcohol consumption levels.[10-12] As a result, we considered the litre per capita input to be a better estimate of overall volume of consumption. Per capita consumption, however, does not provide age- and sex-specific consumption estimates needed to compute alcohol-attributable burden of disease. Therefore, we use the age-sex pattern of consumption among drinkers modelled from the population survey data and the overall volume of consumption from FAO, GISAH, and Euromonitor to determine the total amount of alcohol consumed within a location. In the paragraphs below, we outline how we estimated each primary input in the alcohol exposure model, as well as how we combined these inputs to arrive at our final estimate of grams per day of pure alcohol. We estimated all models below using 1000 draws.

For data obtained through surveys, we used spatiotemporal Gaussian process regression (ST-GPR) to construct estimates for each location/year/age/sex. We chose to use ST-GPR due to its ability to leverage information across the nearby locations or time periods. We also modelled the alcohol litres per capita (LPC) data, as well as the total number of tourists, using ST-GPR.

Given the heterogeneous nature of the estimates on unrecorded consumption, as well as the wide variation across countries and time periods, we took 1000 draws from the uniform distribution of the lowest and highest estimates available for a given country. We did this to incorporate the diffuse uncertainty within the unrecorded estimates reported. We used these 1000 draws in the equation below.

We adjusted the alcohol LPC for unrecorded consumption using the following equation:

$$Alcohol\ LPC = \frac{Alcohol\ LPC}{(1 - \%\ Unrecorded)}$$

We then adjusted the estimates for alcohol LPC for tourist consumption by adding in the per capita rate of consumption abroad and subtracting the per capita rate of tourist consumption domestically.

$$Alcohol\ LPC_d = Unadjusted\ Alcohol\ LPC_d + Alcohol\ LPC_{Domestic\ consumption\ abroad} - Alcohol\ LPC_{Tourist\ consumption\ domestically}$$

$$Alcohol\ LPC_i = \frac{\sum_l Tourist\ Population_l * Proportion\ of\ tourists_{i,l} * Unadjusted\ Alcohol\ LPC_l * \frac{Average\ length\ of\ stay_{i,l}}{365} *}{Population_d}$$

where:

l *is the set of all locations*, i *is either* Domestic consumption abroad *or* Tourist consumption domestically, *and* d *is a domestic location.*

After adjusting alcohol LPC by tourist consumption and unrecorded consumption for all location/years reported, sex-specific and age-specific estimates were generated by incorporating estimates modelled in ST-GPR for percentage of current drinkers within a location/year/sex/age, as well as consumption trends modelled in the ST-GPR grams per day model. We do this by first calculating the proportion of total consumption for a given location/year by age and sex, using the estimates of alcohol consumed per day, the population size, and the percentage of current drinkers. We then multiply this proportion of total stock for a given location/year/sex/age by the total stock for a given location/year to calculate the consumption in terms of litres per capita for a given location/year/sex/age. We then convert these estimates to be in terms of grams/per day. The following equations describe these calculations:

$$Proportion\ of\ total\ consumption_{l,y,s,a} = \frac{Alcohol\ \ g/day_{\ l,y,s,a} * Population_{\,l,y,s,a} * \%\ Current\ drinkers_{\,l,y,s,a}}{\sum_{s,a} Alcohol\ \ g/day_{\,l,y,s,a} * Population_{\,l,y,s,a} * \%\ Current\ drinkers_{\,l,y,s,a}}$$

$$Alcohol\ LPC_{\,l,y,s,a} = \frac{Alcohol\ LPC_{\,l,y} * Population_{\,l,y} * Proportion\ of\ total\ consumption_{\,l,y,s,a}}{\%\ Current\ drinkers_{\,l,y,s,a} * Population_{\,l,y,s,a}}$$

$$Alcohol\ g/day_{\,l,y,s,a} = Alcohol\ LPC_{\,l,y,s,a} * \frac{1000}{365}$$

where:

$l\ is\ a\ location, y\ is\ a\ year, s\ is\ a\ sex, and\ a\ is\ an\ age\ group.$

We then used the gamma distribution to estimate individual-level variation within location, year, sex, age drinking populations, following the recommendations of other published alcohol studies.[7,8] We chose parameters of the gamma distribution based on the mean and standard deviation of the 1,000 draws of alcohol g/day exposure for a given population. Standard deviation was calculated using the following formula.[15] We tested several alternative models using our data and found this model performed best.

$$standard\ deviation = mean * (0.087 * female + \ 1.171\ )$$

Theoretical minimum-risk exposure level

We calculated TMREL by first calculating the overall risk attributable to alcohol. We did this by weighting each relative risk curve by the share of overall DALYs for a given cause. We then took the minimum of this overall-risk curve as the TMREL of alcohol use. More formally,

$$TMREL = argmin\ average\ overall\ risk_{\omega}(g/day)$$

$$Average\ overall\ risk_{\omega}(g/day) = \sum_{i}^{\omega} RR_i(g/day) * \frac{DALY_i}{\sum_i^{\omega} DALY_i}$$

Where:

$\omega\ is\ the\ set\ of\ causes\ associated\ with\ alcohol, i\ is\ a\ given\ cause\ from\ that\ set,$
$DALY\ is\ the\ global\ DALY\ rate\ in\ 2010, and\ RR\ is\ the\ dose\ response\ curve\ for\ a\ given\ cause$
$and\ exposure\ level\ in\ grams\ per\ day.$

In other words, we chose TMREL as being the exposure that minimises your risk of suffering burden from any given cause related to alcohol. We weight the risk for a particular cause in our aggregation by the proportion of DALYs due to that cause (eg, since more observed people die from ischaemic heart disease [IHD], we weight the risk for IHD more in the above calculation of average risk compared to, say, diabetes, even if both have the same relative risk for a given level of consumption).

Relative risks

We used the studies identified through the systematic review to calculate a dose-response, modelled using DisMod ODE. We chose DisMod ODE rather than a conventional mixed effects meta-regression because of its ability to estimate nonparametric splines over doses (ie, for most alcohol causes, there is a non-linear relationship with different doses) and incorporate heterogeneous doses through dose-integration (ie, most studies report doses categorically in wide ranges. DisMod ODE estimates specific doses when categories overlap across studies, through an integration step.). We used the results of the meta-regression to estimate a non-parametric curve for all doses between zero and 150 g/day and their corresponding relative risks. For TB, we assumed the relative risk was the same for all ages and sexes.

Table 4: Data inputs for relative risks for alcohol use.

Input data	Relative risk
Sources (total)	495

Table 5: All-ages and both sexes relative risks for tuberculosis mortality by grams of pure alcohol consumed per day (g/day) from continuous dose-response meta-regression

Dose	Relative risk
0 g/day	1.0<br>(1.0 to 1.0)
12 g/day	1.101<br>(0.815 to 1.425)
24 g/day	1.531<br>(1.165 to 1.98)
36 g/day	2.058<br>(1.485 to 2.795)
48 g/day	2.535<br>(1.701 to 3.51)
60 g/day	2.994<br>(1.972 to 4.204)
72 g/day	3.507<br>(2.596 to 4.474)

Population attributable fraction

For all causes, we defined PAF as:

$$PAF(x) = \frac{P_A + \int_0^{150} P(x) * RR_C(x)\,dx - 1}{P_A + \int_0^{150} P(x) * RR_C(x)\,dx} \qquad P(x) = P_C * \Gamma(\boldsymbol{p})$$

where:

P_C $is\ the\ prevalence\ of\ current\ drinkers, P_a\ is\ the\ prevalence\ of\ abstainers, RR_c(x)\ is\ the\ relative$

$risk\ function\ for\ current\ drinkers, and\ \boldsymbol{p}\ are\ parameters\ determined\ by\ the\ mean\ and\ sd\ of\ exposure$

We performed the above equation for 1000 draws of the exposure and relative risk models. We then used the estimated PAF draws to calculate YLL, YLDs, and DALYs, as per the other risk factors.

References

1. Food and Agriculture Organization of the United Nations (FAO). FAOSTAT Food Balance Sheets, October 2014. Rome, Italy: Food and Agriculture Organization of the United Nations (FAO).
2. World Health Organization (WHO). WHO Global Health Observatory - Recorded adult per capita alcohol consumption, Total per country. Geneva, Switzerland: World Health Organization (WHO).
3. UN World Tourism Organization (UNWTO). UN World Tourism Organization Compendium of Tourism Statistics 2015 [Electronic]. Madrid, Spain: UN World Tourism Organization (UNWTO), 2016.
4. Ramstedt, Mats. “How much alcohol do you buy? A comparison of self-reported alcohol purchases with actual sales.” *Addiction* 105.4 (2010): 649-654.
5. Stockwell, Tim, et al. “Under-reporting of alcohol consumption in household surveys: a comparison of quantity–frequency, graduated–frequency and recent recall.” *Addiction* 99.8 (2004): 1024-1033.
6. Kerr, William C., and Thomas K. Greenfield. “Distribution of alcohol consumption and expenditures and the impact of improved measurement on coverage of alcohol sales in the 2000 National Alcohol Survey.” *Alcoholism: Clinical and Experimental Research* 31.10 (2007): 1714-1722.
7. Taylor, Bruce, et al. “The more you drink, the harder you fall: a systematic review and meta-analysis of how acute alcohol consumption and injury or collision risk increase together.” *Drug and alcohol dependence* 110.1 (2010): 108-116.
8. Vinson, Daniel C., Guilherme Borges, and Cheryl J. Cherpitel. “The risk of intentional injury with acute and chronic alcohol exposures: a case-control and case-crossover study.” *Journal of studies on alcohol* 64.3 (2003): 350-357.
9. Vinson, Daniel C., et al. “A population-based case-crossover and case-control study of alcohol and the risk of injury.” *Journal of studies on alcohol*64.3 (2003): 358-366.
10. Fatal Accident Reporting System (FARS). National Highway Traffic Safety Administration, National Center for Statistics and Analysis Data Reporting and Information Division (NVS-424); 1985, 1990, 1995, 2000, 2005, 2010, 2015
11. Chen, Li-Hui, Susan P. Baker, and Guohua Li. “Drinking history and risk of fatal injury: comparison among specific injury causes.” Accident Analysis & Prevention 37.2 (2005): 245-251.
12. Bell, Nicole S., et al. “Self-reported risk-taking behaviors and hospitalization for motor vehicle injury among active duty army personnel.” American journal of preventive medicine 18.3 (2000): 85-95.

13. Margolis, Karen L., et al. “Risk factors for motor vehicle crashes in older women.” The Journals of Gerontology Series A: Biological Sciences and Medical Sciences 57.3 (2002): M186-M191.
14. Sorock, Gary S., et al. “Alcohol-drinking history and fatal injury in older adults.” Alcohol 40.3 (2006): 193-199.
15. Kehoe, Tara et al. “Determining the best population-level alcohol consumption model and its impact on estimates of alcohol-attributable harms.” Population health metrics 10 6. (2012)

Smoking[2]

Flowchart

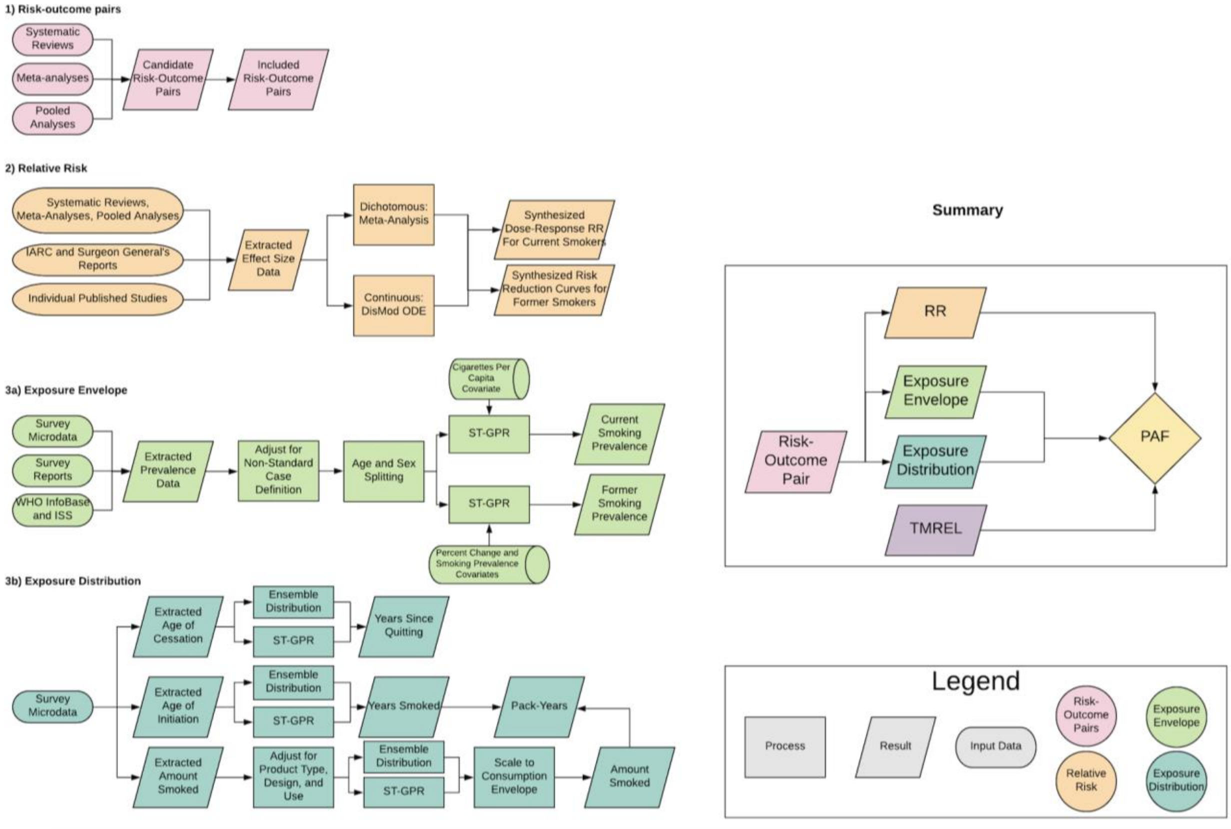

Input data and methodological summary

Definition

Exposure

As in GBD 2017, we estimated the prevalence of current smoking and the prevalence of former smoking using data from cross-sectional nationally representative household surveys. We defined current smokers as individuals who currently use any smoked tobacco product on a daily or occasional basis. We defined former smokers as individuals who quit using all smoked tobacco products for at least six months, where possible, or according to the definition used by the survey.

Input data

Our extraction method has not changed from GBD 2017. We extracted primary data from individual-level microdata and survey report tabulations. We extracted data on current, former, and/or ever smoked tobacco use reported as any combination of frequency of use (daily, occasional, and unspecified, which includes both daily and occasional smokers) and type of smoked tobacco used (all smoked tobacco, cigarettes, hookah, and other smoked tobacco products such as cigars or pipes),

resulting in 36 possible combinations. Other variants of tobacco products, for example hand-rolled cigarettes, were grouped into the four type categories listed above based on product similarities.

For microdata, we extracted relevant demographic information, including age, sex, location, and year, as well as survey metadata, including survey weights, primary sampling units, and strata. This information allowed us to tabulate individual-level data in the standard GBD five-year age-sex groups and produce accurate estimates of uncertainty. For survey report tabulations, we extracted data at the most granular age-sex group provided.

Table 1: Data inputs for exposure for smoking.

Input data	Exposure
Source count (total)	3439
Number of countries with data	201

Table 2: Data inputs for relative risks for smoking.

Input data	Relative risk
Source count (total)	673
Number of countries with data	16

Crosswalk

Our GBD smoking case definitions were current smoking of any tobacco product and former smoking of any tobacco product. All other data points were adjusted to be consistent with either of these definitions. Some sources contained information on more than one case definition and these sources were used to develop the adjustment coefficient to transform alternative case definitions to the GBD case definition. The adjustment coefficient was the beta value derived from a linear model with one predictor and no intercept. We used the same crosswalk adjustment coefficients as in GBD 2017, and thus we have not included a methods explanation in this appendix, as it has been detailed previously.

Age and sex splitting

As in GBD 2017, we split data reported in broader age groups than the GBD 5-year age groups or as both sexes combined by adapting the method reported in Ng et al[1] to split using a sex- geography- time-specific reference age pattern. We separated the data into two sets: a training dataset, with data already falling into GBD sex-specific 5-year age groups, and a split dataset, which reported data in aggregated age or sex groups. We then used spatiotemporal Gaussian process regression (ST-GPR) to estimate sex-geography-time-specific age patterns using data in the training dataset. The estimated age patterns were used to split each source in the split dataset.

The ST-GPR model used to estimate the age patterns for age-sex splitting used an age weight parameter value that minimises the effect of any age smoothing. This parameter choice allowed the estimated age pattern to be driven by data, rather than being enforced by any smoothing parameters of the model.

Because these age-sex split data points were to be incorporated in the final ST-GPR exposure model, we did not want to doubly enforce a modelled age pattern for a given sex-location-year on a given aggregate data point.

Modelling strategy

Smoking prevalence modelling

We used ST-GPR to model current and former smoking prevalence. The model is nearly identical to that in GBD 2017. Full details on the ST-GPR method are reported elsewhere in the appendix. Briefly, the mean function input to GPR is a complete time series of estimates generated from a mixed effects hierarchical linear model plus weighted residuals smoothed across time, space, and age. The linear model formula for current smoking, fit separately by sex using restricted maximum likelihood in R, is:

$$logit(p_{g,a,t}) = \beta_0 + \beta_1 CPC_{g,t} + \sum_{k=2}^{19} \beta_k I_{A[a]} + \alpha_s + \alpha_r + \alpha_g + \epsilon_{g,a,t}$$

Where $CPC_{g,t}$ is the tobacco consumption covariate by geography g and time t, described above, $I_{A[a]}$ is a dummy variable indicating specific age group A that the prevalence point $p_{g,a,t}$ captures, and α_s, α_r, and α_g are super-region, region, and geography random intercepts, respectively. Random effects were used in model fitting but not in prediction.

The linear model formula for former smoking is:

$$logit(p_{g,a,t}) = \beta_0 + \beta_1 PctChange_{A[a],g,t} + \beta_3 CSP_{A[a],g,t} + \sum_{k=3}^{20} \beta_k I_{A[a]} + \alpha_s + \alpha_r + \alpha_g + \epsilon_{g,a,t}$$

Where $PctChange_{A[a],g,t}$ is the percentage change in current smoking prevalence from the previous year, and $CSP_{A[a],g,t}$ is the current smoking prevalence by specific age group A, geography g, and time t that point $p_{g,a,t}$ captures, both derived from the current smoking ST-GPR model defined above.

Supply-side estimation

The methods for modelling supply-side-level data were changed substantially from those used in GBD 2017. The raw data were domestic supply (USDA Global Surveillance Database and UN FAO) and retail supply (Euromonitor) of tobacco. Domestic supply was calculated as production + imports - exports. The data went through three rounds of outliering. First, they were age-sex split using daily smoking prevalence to generate number of cigarettes per smoker per day for a given location-age-sex-year. If more than 12 points for a particular source-location-year (equal to over 1/3 of the split points) were above the given thresholds, that source-location-year was outliered. A point would not be outliered if it was (in cigarettes per smoker): under five (10–14 year olds); under 20 (males, 15–19 year olds); under 18 (females, 15–19 year olds); under 38/35 and over three (males/females, 20+ year olds). These thresholds were chosen by visualising histograms of the data for each age-sex, as well as with expert knowledge about reasonable consumption levels. In the second round of outliering, the mean tobacco

per capita value over a 10-year window was calculated. If a point was over 70% of that mean value away from the mean value, it was outliered. The 70% limit was chosen using histograms of these distances. Additionally, some manual outliering was performed to account for edge cases. Finally, data smoothing was performed by taking a three-year rolling mean over each location-year.

Next, a simple imputation to fill in missing years was performed for all series to remove compositional bias from our final estimates. Since the data from our main sources covered different time periods, by imputing a complete time series for each data series, we reduced the probability that compositional bias of the sources was leading to biased final estimates. To impute the missing years for each series, we modelled the log ratio of each pair of sources as a function of an intercept and nested random effects on super-region, region, and location. The appropriate predicted ratio was multiplied by each source that we did have, and then the predictions were averaged to get the final imputed value. For example, if source A was missing for a particular location-year, but sources B and C were present, then we predicted A twice: once from the modelled ratio of A to B, and again from the modelled ratio of A to C. These two predictions were then averaged. For some locations where there was limited overlap between series, the predicted ratio did not make sense, and a regional ratio was used.

Finally, variance was calculated both across series (within a location-year) as well as across years (within a location-source). Additionally, if a location-year had one imputed point was, the variance was multiplied by 2. If a location-year had two imputed points, the variance was multiplied by 4. The average estimates in each location-year were the input to an ST-GPR model. For this, we used a simple mixed effects model, which was modelled in log space with nested location random effects. Subnational estimates were then further modelled by splitting the country-level estimates using current smoking prevalence.

Theoretical minimum-risk exposure level

The theoretical minimum-risk exposure level is 0.

Exposure among current and former smokers

Identical to GBD 2017, we estimated exposure among current smokers for two continuous indicators: cigarettes per smoker per day and pack-years. Pack-years incorporates aspects of both duration and amount. One pack-year represents the equivalent of smoking one pack of cigarettes (assuming a 20-cigarette pack) per day for one year. Since the pack-years indicator collapses duration and intensity into a single dimension, one pack-year of exposure can reflect smoking 40 cigarettes per day for six months or smoking 10 cigarettes per day for two years.

To produce these indicators, we simulated individual smoking histories based on distributions of age of initiation and amount smoked. We informed the simulation with cross-sectional survey data capturing these indicators, modelled at the mean level for all locations, years, ages, and sexes using ST-GPR. We rescaled estimates of cigarettes per smoker per day to an envelope of cigarette consumption based on supply-side data. We estimated pack-years of exposure by summing samples from age- and time-specific distributions of cigarettes per smoker for a birth cohort in order to capture both age trends and time trends and avoid the common assumption that the amount someone currently smokes is the amount they have smoked since they began smoking. All distributions were age-, sex-, and region- specific ensemble distributions, which were found to outperform any single distribution.

We estimated exposure among former smokers using years since cessation. We utilised ST-GPR to model mean age of cessation using cross-sectional survey data capturing age of cessation. Using these estimates, we generated ensemble distributions of years since cessation for every location, year, age group, and sex.

Relative risk

Dose-response risk curves

We examined the smoking-tuberculosis risk-outcome pair using the same input data for relative risks as in GBD 2017. We synthesised effect sizes by cigarettes per smoker per day, pack-years, and years since quitting from cohort and case-control studies to produce nonlinear dose-response curves using a Bayesian meta-regression model. For outcomes with significant differences in effect size by sex or age, we produced sex- or age-specific risk curves.

We estimated risk curves of former smokers compared to never smokers taking into account the rate of risk reduction among former smokers seen in the cohort and case-control studies, and the cumulative exposure among former smokers within each age, sex, location, and year group.

Table 3: All-age and both sex relative risks for tuberculosis mortality by the number of cigarettes per day from continuous dose-response meta-regression

Dose	Relative risk
0 Cigarette-Equivalents	1.00 (1.00 - 1.00)
1 Cigarette-Equivalents	1.12 (1.07 - 1.17)
5 Cigarette-Equivalents	1.60 (1.34 - 1.86)
10 Cigarette-Equivalents	2.10 (1.68 - 2.52)
20 Cigarette-Equivalents	2.47 (2.03 - 2.97)
30 Cigarette-Equivalents	3.36 (2.61 - 4.34)

Population attributable fraction (PAF)

As in GBD 2017, we estimated PAFs based on the following equation:

$$PAF = \frac{p(n) + p(f) \int \exp(x) * rr(x) + p(c) \int \exp(y) * rr(y) - 1}{p(n) + p(f) \int \exp(x) * rr(x) + p(c) \int \exp(y) * rr(y)}$$

where $p(n)$ is the prevalence of never smokers, $p(f)$ is the prevalence of former smokers, $p(c)$ is the prevalence of current smokers, $\exp(x)$ is a distribution of years since quitting among former smokers, $rr(x)$ is the relative risk for years since quitting, $\exp(y)$ is a distribution of cigarettes per smoker per day or pack-years, and $rr(y)$ is the relative risk for cigarettes per smoker per day or pack-years.

We used cigarettes per smoker per day as the exposure definition for tuberculosis.

References

1. Ng M, Freeman MK, Fleming TD, Robinson M, Dwyer-Lindgren L, Thomson B, et al. Smoking Prevalence and Cigarette Consumption in 187 Countries, 1980–2012. *JAMA*. 2014 Jan 8;311(2):183–92.

High fasting plasma glucose/Diabetes[2]

Flowchart

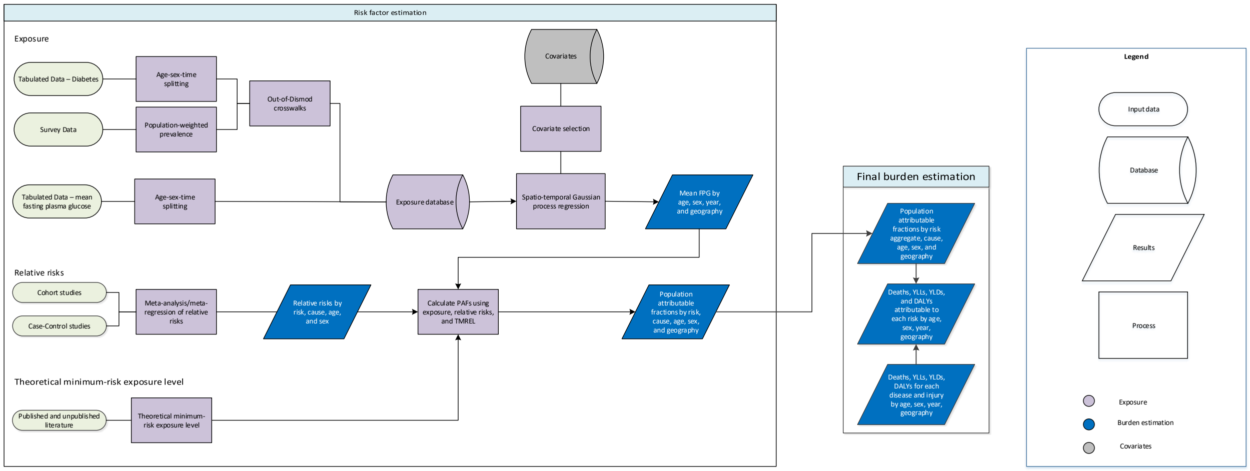

Case definition

High fasting plasma glucose (FPG) is measured as the mean FPG in a population, where FPG is a continuous exposure in units of mmol/L. Since FPG is along a continuum, we define high FPG as any level above the TMREL, which is 4.8-5.4 mmol/L.

Data seeking

Exposure

We conducted a systematic review for FPG and diabetes in GBD 2019. We use all available sources on FPG and prevalence of diabetes in the FPG model.

1. Search terms:

Diabetes Mellitus search string: (diabetes[TI] AND (prevalence[TIAB] OR incidence[TIAB])) OR ('Diabetes Mellitus'[MeSH Terms] AND 'epidemiology'[MeSH Terms]) OR (diabetes[TI] AND 'epidemiology'[MeSH Terms]) NOT gestational[All Fields] NOT ('neoplasms'[MeSH Terms] OR 'neoplasms'[All Fields] OR 'cancer'[All Fields]) NOT ('mice'[MeSH Terms] OR 'mice'[All Fields]) NOT ('schizophrenia'[MeSH Terms] OR 'schizophrenia'[All Fields]) NOT ('emigrants and immigrants'[MeSH Terms] OR ('emigrants'[All Fields] AND 'immigrants'[All Fields]) OR 'emigrants and immigrants'[All Fields] OR 'immigrants'[All Fields]) NOT ('pregnancy'[MeSH Terms] OR 'pregnancy'[All Fields] OR 'gestation'[All Fields]) NOT ('rats'[MeSH Terms] OR 'rats'[All Fields] OR 'rat'[All Fields]) NOT ('kidney'[MeSH Terms] OR 'kidney'[All Fields]) NOT renal[All Fields] NOT ('vitamins'[Pharmacological Action] OR 'vitamins'[MeSH Terms] OR 'vitamins'[All Fields] OR 'vitamin'[All Fields])

And

FPG search string: ((“glucose”[Mesh] OR “hyperglycemia”[Mesh] OR “prediabetic state”[Mesh]) AND "Geographic Locations"[Mesh] NOT "United States"[Mesh]) AND ("humans"[Mesh] AND "adult"[MeSH])

AND ("Data Collection"[Mesh] OR "Health Services Research"[Mesh] OR "Population Surveillance"[Mesh] OR "Vital statistics"[Mesh] OR "Population"[Mesh] OR "Epidemiology"[Mesh] OR surve*[TiAb]) NOT Comment[ptyp] NOT Case Reports[ptyp]) NOT "hospital"[TiAb]

Search date: October 17, 2018. The search took place for the following dates: 10/15/2017-10/16/2018. The number of studies returned was 717, and the number of studies extracted was 36.

Figure 1: PRISMA diagram of data sources used in GBD 2019 high fasting plasma glucose model

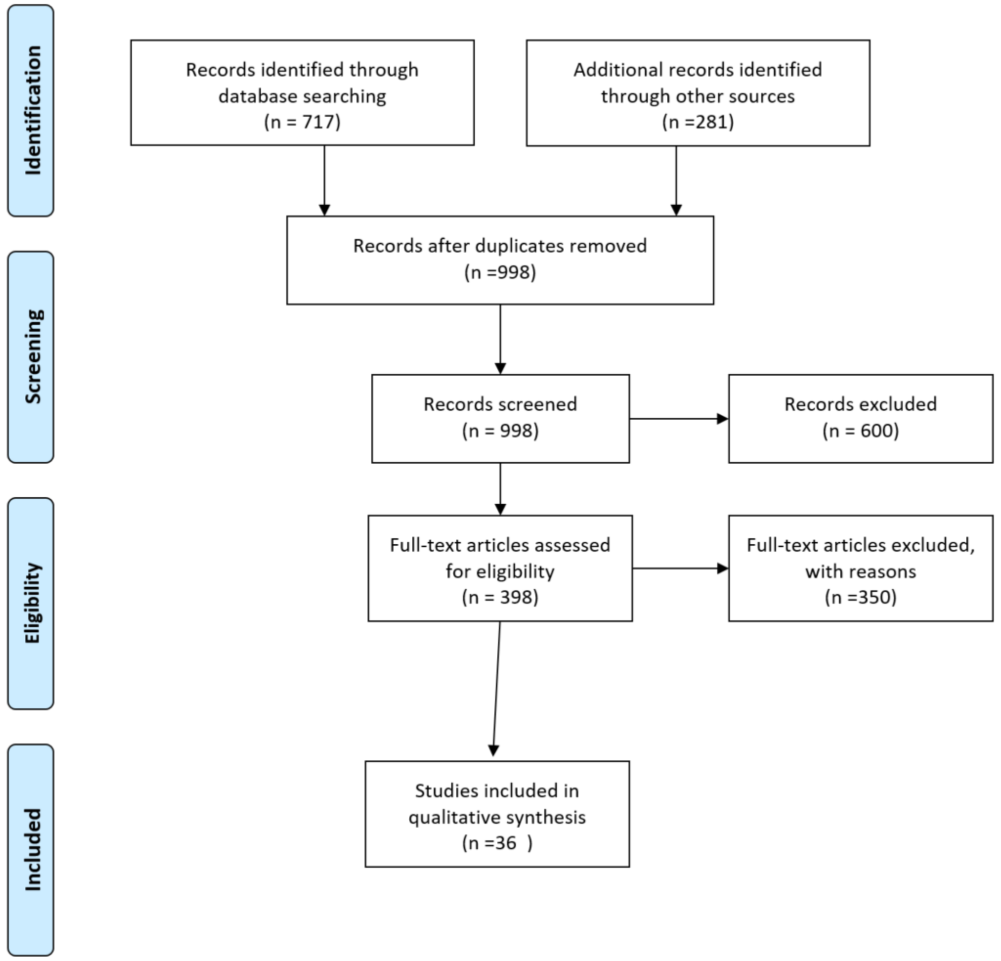

Data inputs

Data inputs come from 3 sources:

- Estimates of mean FPG in a representative population
- Individual-level data of fasting plasma glucose measured from surveys
- Estimates of diabetes prevalence in a representative population

Data sources that did not report mean FPG or prevalence of diabetes are excluded from analysis. When a study reported both mean fasting plasma glucose (FPG) and prevalence of diabetes, we use the mean FPG for exposure estimates. Where possible, individual-level data supersede any data described in a study. Individual-level data are aggregated to produce estimates for each 5-year age group, sex, location, and year of a survey.

Table 1: Number of sources used in exposure and relative risk models in GBD 2019

Measure	Total sources	Countries with data
Total	549	127
Relative risk	20	-
Exposure	529	127

Data processing

We perform several processing steps to the data in order to address sampling and measurement inconsistencies that will ensure the data are comparable.

1. *Small sample size*
   Estimates in a sex and age group with a sample size <30 persons is considered a small sample size. In order to avoid small sample size problems that may bias estimates, data are collapsed into the next age group in the same study till the sample size reach at least 30 persons. The intent of collapsing the data is to preserve as much granularity between age groups as possible. If the entire study sample consists of <30 persons and did not include a population-weight, the study is excluded from the modelling process.

2. *Crosswalks*
   We predicted mean FPG from diabetes prevalence using an ensemble distribution. We characterized the distribution of FPG using individual-level data. Details on the ensemble distribution can be found elsewhere in the Appendix. Before predicting mean FPG from prevalence of diabetes, we ensured that the prevalence of diabetes was based on the reference case definition: fasting plasma glucose (FPG) >126 mg/dL (7 mmol/L) or on treatment. For more details on how the case-definition crosswalk is conducted, please see the diabetes mellitus appendix in *Global, regional, and national incidence, prevalence, and years lived with disability for 354 diseases and injuries for 195 countries, 1990–2019: a systematic analysis for the Global Burden of Disease Study 2019*.

Exposure modelling

Exposure estimates are produced for every year between 1980 to 2019 for each national and subnational location, sex, and for each 5-year age group starting from 25 years. As in previous rounds of GBD, we used a Spatio-Temporal Gaussian Process Regression (ST-GPR) framework to model the mean fasting plasma glucose at the location-, year-, age-, and sex- level. Updates to the ST-GR modelling framework for GBD 2019 are detailed elsewhere in the Appendix.

Fasting plasma glucose is frequently tested or reported in surveys aiming at assessing the prevalence of diabetes mellitus. In these surveys, the case definition of diabetes may include both a glucose test and questions about treatment for diabetes. People with positive history of diabetes treatment may be excluded from the FPG test. Thus, the mean FPG in these surveys would not represent the mean FPG in the entire population. In this event, we estimated the prevalence of diabetes assuming a definition of

FPG>126 mg/dL (7mmol/L), then crosswalked it to our reference case definition, and then predicted mean FPG.

To inform our estimates in data-sparse countries, we systematically tested a range of covariates and selected age specific prevalence of obesity as a covariate based on direction of the coefficient and significance level.

Mean FPG is estimated using a mixed-effects linear regression, run separately by sex:

$$\mathrm{logit}(\mathrm{FPG}_{c,a,t}) = \beta_0 + \beta_1 p_{\mathrm{overweight}_{c,a,t}} + \sum_{k=2}^{16} \beta_k I_{A[a]} + \alpha_s + \alpha_r + \alpha_c + \epsilon_{c,a,t}$$

where $p_{\mathrm{overweight}_{c,a,t}}$ is the prevalence of overweight, $I_{A[a]}$ is an indicator variable for a fixed effect on a given 5-year age group, and α_s α_r α_c are random effects at the super-region, region, and country level, respectively. The estimates were then propagated through the ST-GPR framework to obtain 1000 draws for each location, year, age, and sex.

Theoretical minimum-risk exposure level

The theoretical minimum-risk exposure level (TMREL) for FPG is 4.8-5.4 mmol/L. This was calculated by taking the person-year weighted average of the levels of FPG that were associated with the lowest risk of mortality in the pooled analyses of prospective cohort studies.[1]

Relative risks

Relative risks for Diabetes mellitus (Categorical risk)

Age-specific relative risks for tuberculosis were obtained from meta-analysis of cohort studies. The results are in the table below:

Table 2: Categorical relative risks (both-sexes) for Diabetes mellitus used by age for Tuberculosis mortality

Age group	Not diabetic relative risk	Diabetic relative risk
25 to 29 years	1.0<br>(1.0 to 1.0)	2.73<br>(1.973 to 3.602)
30 to 34 years	1.0<br>(1.0 to 1.0)	2.801<br>(2.056 to 3.658)
35 to 39 years	1.0<br>(1.0 to 1.0)	2.871<br>(2.047 to 3.698)
40 to 44 years	1.0<br>(1.0 to 1.0)	2.798<br>(1.97 to 3.63)
45 to 49 years	1.0<br>(1.0 to 1.0)	2.581<br>(1.91 to 3.268)
50 to 54 years	1.0<br>(1.0 to 1.0)	2.364<br>(1.817 to 2.943)

55 to 59 years	1.0<br>(1.0 to 1.0)	2.147<br>(1.692 to 2.673)
60 to 64 years	1.0<br>(1.0 to 1.0)	1.93<br>(1.485 to 2.44)
65 to 69 years	1.0<br>(1.0 to 1.0)	1.713<br>(1.231 to 2.317)
70 to 74 years	1.0<br>(1.0 to 1.0)	1.598<br>(1.125 to 2.239)
75 to 79 years	1.0<br>(1.0 to 1.0)	1.587<br>(1.184 to 2.115)
80 to 84 years	1.0<br>(1.0 to 1.0)	1.559<br>(1.182 to 2.174)
85 to 89 years	1.0<br>(1.0 to 1.0)	1.559<br>(1.182 to 2.174)
90 to 94 years	1.0<br>(1.0 to 1.0)	1.559<br>(1.182 to 2.174)
95+ years	1.0<br>(1.0 to 1.0)	1.559<br>(1.182 to 2.174)

References

1 . Singh GM, Danaei G, Farzadfar F, *et al.* The age-specific quantitative effects of metabolic risk factors on cardiovascular diseases and diabetes: a pooled analysis. *PloS One* 2013; **8**: e65174.

Unsafe sex[2]

Flowchart

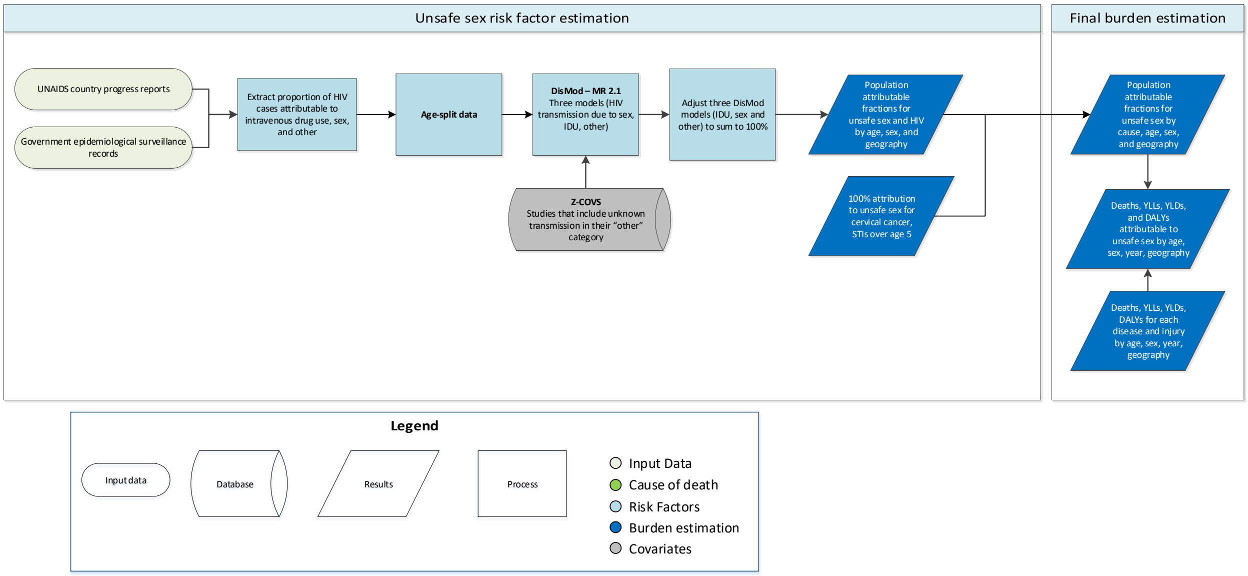

Input data and methodological summary

Definition

Exposure

Unsafe sex is defined as the risk of disease due to sexual transmission. The outcomes associated with unsafe sex that we estimate for GBD include HIV, cervical cancer, and all sexually transmitted diseases (STDs) except for those in neonates from vertical transmission, including HIV, *Opthalmia neonatorum* and neonatal syphilis. We modelled the proportion of HIV incidence occurring through sexual transmission to estimate the attributable burden for HIV due to unsafe sex. The theoretical minimum level (TMREL) for unsafe sex is defined as the absence of disease transmission due to sexual contact.

Input data

To be used in our models, sources must report HIV cases attributable to various modes of transmission. We screened UNAIDS country progress reports and searched government epidemiological surveillance records for these data. The primary data sources we used were UNAIDS, the European CDC, and the US CDC.

We excluded all extractions where the "other" category for HIV transmissions accounted for greater than 25% of all cases. We believe that such high proportions raise concerns about the quality of reporting.

Input data	Exposure
Source count (total)	948

Number of countries with data	97

Modelling strategy

We modelled the proportion of HIV cases attributable to unsafe sex. To do this we collected and cleaned data, ran three DisMod-MR models (HIV attributable to sex, HIV attributable to injection drug use, HIV attributable to other routes of transmission), adjusted results of the three DisMod-MR models to sum to one, and then assigned the proportions as direct PAFs.

No country-level covariates were included in the models. We tested an injection drug use (IDU) covariate – an opioid use covariate in the proportion HIV due to drug use model – but found no significant coefficients, so excluded them from the final model.

Since all-age and both-sex datapoints represent the vast majority of the available data, we derived an age-sex pattern for the HIV-IDU transmission model from the age-sex pattern present in the GBD 2017 population attributable fraction for hepatitis B attributable to IDU (the model for injecting drug use and hepatitis estimates the cumulative exposure to injecting drug use to capture all infections in people with a history of injecting even if in a more distant past). Assuming the proportion of HIV due to other transmission is constant over age and by sex, the age-sex pattern for the proportion of HIV due to sexual transmission was set to be the complement to 1 of the age-sex pattern for the proportion of HIV due to IDU. The all-age and both-sex data were split according to these age-sex patterns, and the three HIV transmission DisMod-MR models were run on the age- and sex-split data. In previous GBD rounds, only age-splitting had used this approach, while sex-splitting occurred within DisMod-MR. Since most data are for both sexes combined, using the sex ratio – in addition to the age pattern from the IDU-Hepatitis B PAF – is much more informative. The impact of this change resulted in general increases in proportion HIV due to sexual transmission among females, as they generally had lower IDU rates compared to males.

In GBD 2019, we also changed the proportion HIV due to sex DisMod-MR model to run in complement (1-proportion) space. Since proportions were high in most countries, modelling in complement space resulted in a better model fit. Additional priors were set to inform an age pattern: zero proportion HIV transmission due to IDU before age 15, zero proportion HIV transmission due to sex before age 10 (100 in complement space), and 100% transmission due to other before age 10. The results from these HIV transmission models were adjusted to sum to 100% for a given country-year-age-sex group at each of 1,000 draws.

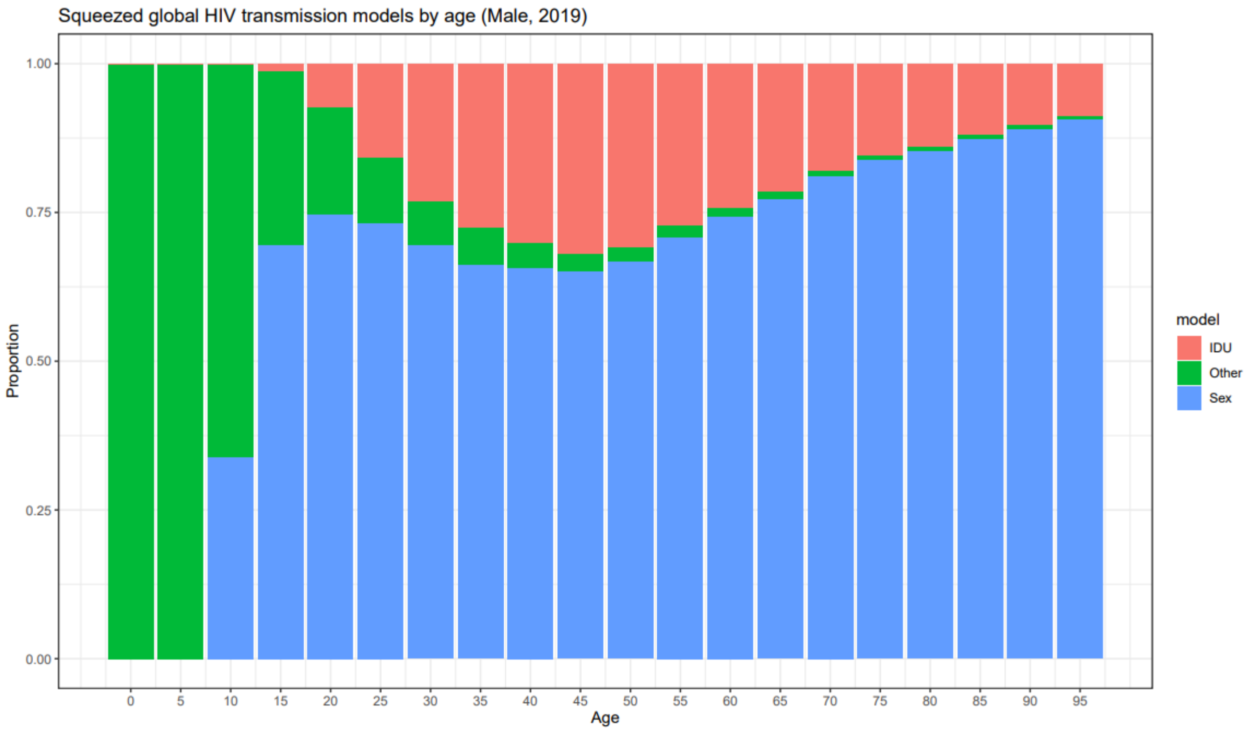

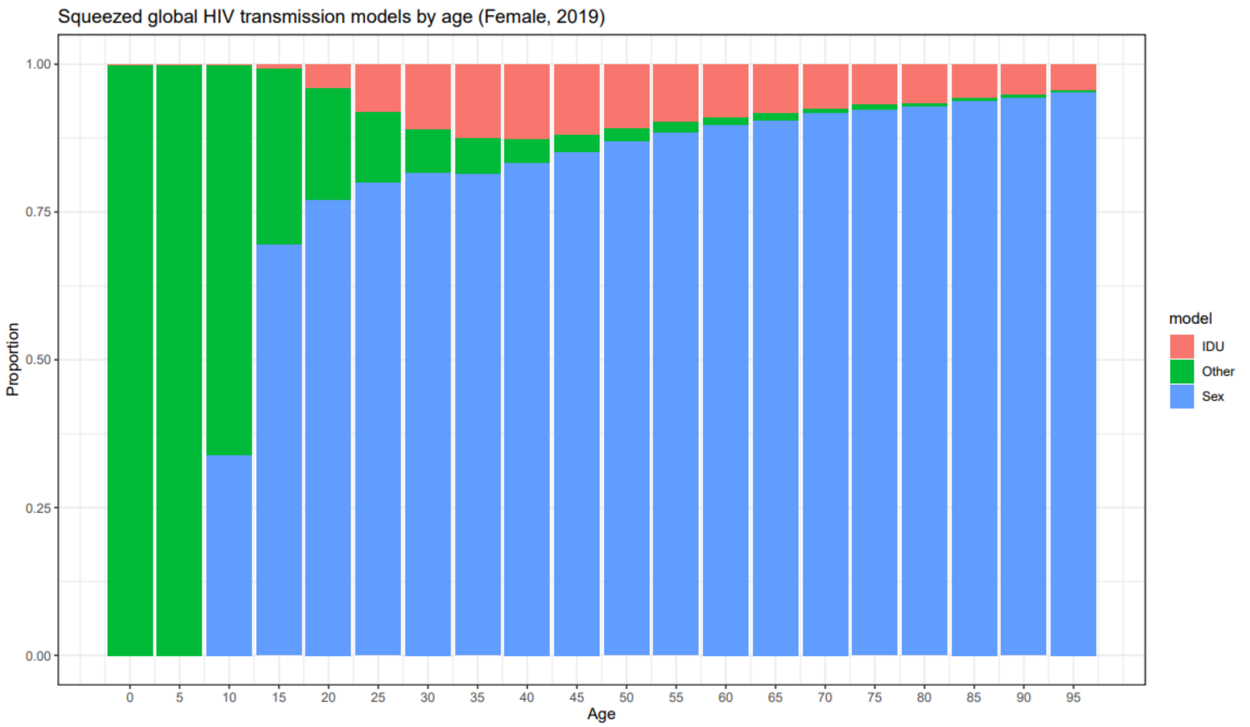

Theoretical minimum-risk exposure level

The theoretical minimum level used for unsafe sex is the absence of disease transmission due to sexual contact.

Population attributable fraction calculations

For HIV, the results from the single parameter proportion DisMod-MR model for HIV transmission due to sex after squeezing were used directly as the population attributable fraction.

Drug use[2]

Flowchart

Drug Use

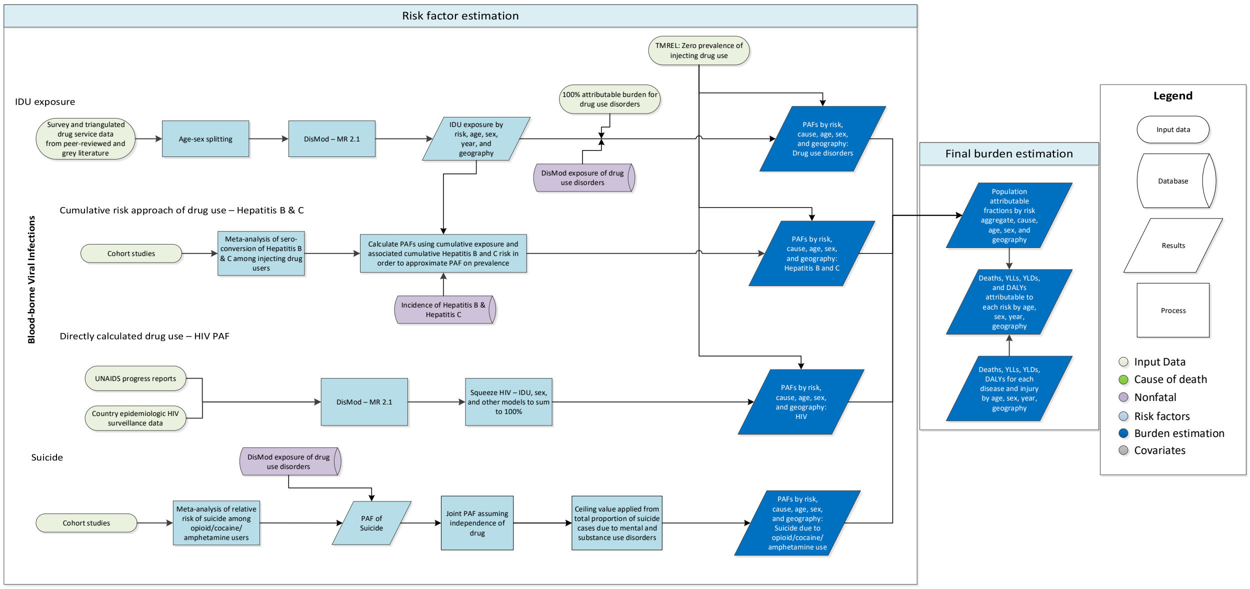

Input data and methodological summary

Exposure definition

The drug use risk factor includes four dimensions of exposure. First, we include 100% attribution of drug use disorder estimates. Second, estimates of prevalence of opioid, amphetamine, and cocaine use disorder are used as exposures for risk of suicide. These drug use disorders are defined based on DSM or ICD diagnostic criteria. Third, instead of starting with an exposure model to estimate the proportion of HIV cases due to injection drug use (IDU), we model the PAF directly, alongside proportion of HIV cases due to sexual transmission and other routes of transmission, which mainly includes blood transfusions. Injecting drug users are at high risk of bloodborne infections due to the use of shared needles and injection equipment. Injecting drug use is defined as current injection drug use among individuals aged 15 to 64. The theoretical minimum-risk exposure level (TMREL) for drug use is defined as zero exposure to drug use.

Input data

To estimate the burden of HIV cases attributable to IDU, we extracted data on the proportion of notified HIV cases by transmission route – sexual intercourse, injecting drug use, and other – from a number of agencies that conduct surveillance of HIV across the globe.[1-8]

The prevalence of current injecting drug use was estimated using data from a multistage process of systematic review. It involved multiple stages of peer and expert review, including review by the

Reference Group to the UN on HIV and injecting drug use,[9] with searches of the peer-reviewed literature in addition to an extensive review of online grey literature databases in the drug and alcohol and HIV fields.

Input data	Exposure
Source count (total)	174
Number of countries with data	75

Modelling strategy

Burden of HIV attributable to injecting drug use

We estimated the proportion of HIV cases attributable to three transmission categories (sex, IDU, and other) for all country-time periods using DisMod-MR 2.1. In previous rounds, data for estimating the proportion of HIV cases attributable to IDU were age-split using the age pattern of the IDU exposure model and sex-split in DisMod. In GBD 2019, these data were age- and sex-split using the estimated IDU exposure age-sex pattern, resulting in increases in the proportion of HIV due to IDU among men and decreases among women. We scaled the proportions from each of the three transmission models (sex, IDU, and other) to ensure that they fit the total HIV transmission envelope by country, year, age and sex. Scaled estimates are used as direct population attributable fractions, meaning that the proportion coming from the model is the proportion of HIV deaths or DALYs attributable to IDU.

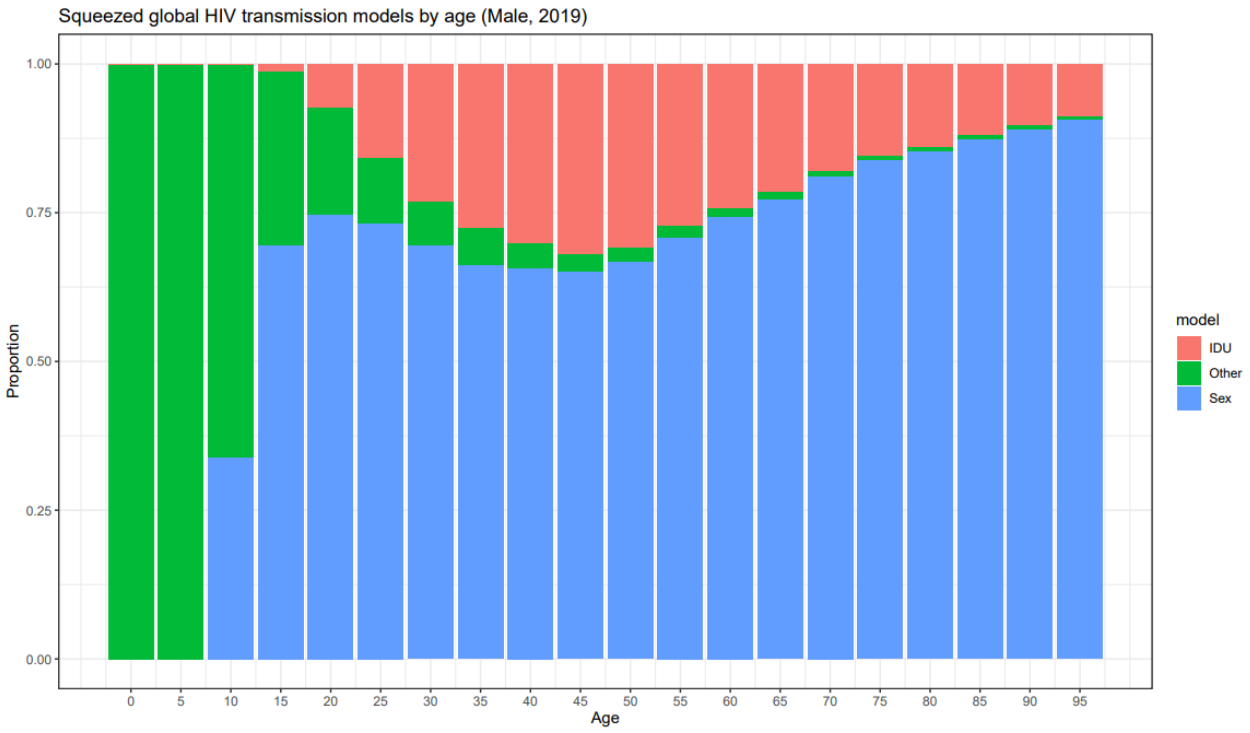

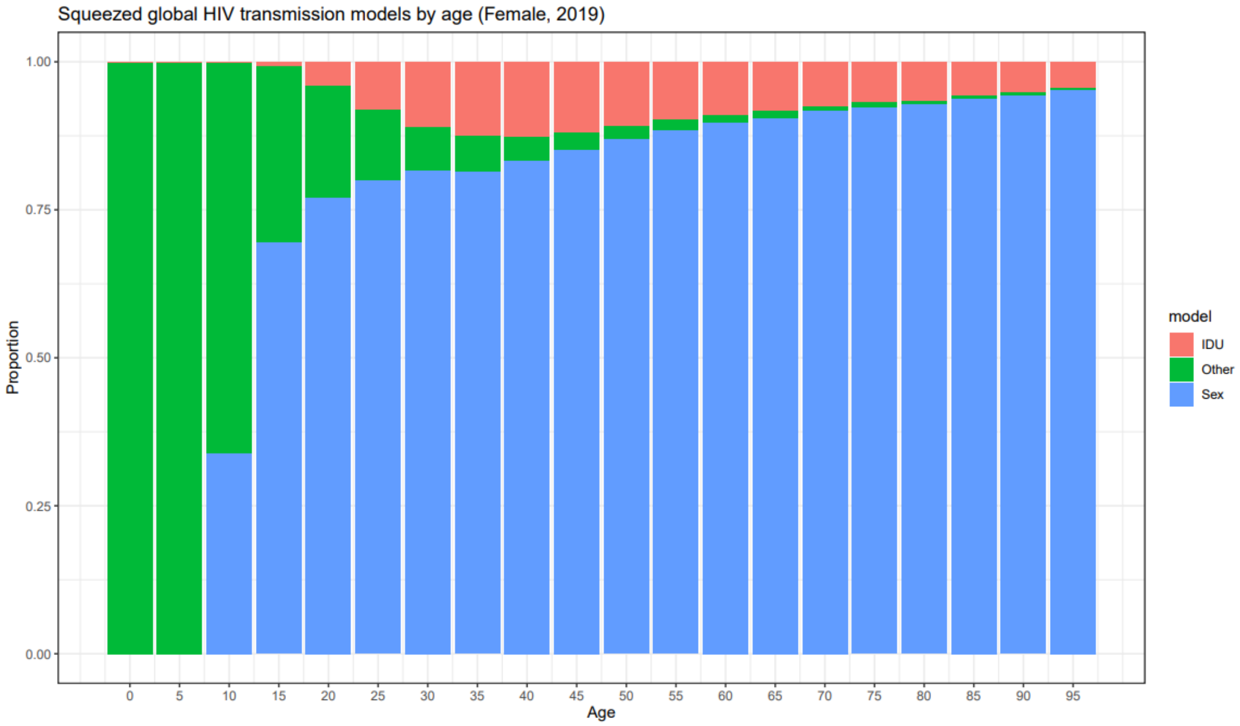
Squeezed global HIV transmission models by age (Female, 2019)
Proportion
1.00
0.75
0.50
0.25
0.00
0 5 10 15 20 25 30 35 40 45 50 55 60 65 70 75 80 85 90 95
Age
model
IDU
Other
Sex

References

1. European Centre for Disease Prevention. HIV/AIDS surveillance in Europe 2014 Solna, Sweden. http://ecdc.europa.eu/en/publications/surveillance_reports/HIV_STI_and_blood_borne_viruses/Pages/HIV_STI_and_blood_borne_viruses.aspx: ECDC, 2014.

2. Family Health International, Bureau of AIDS TB and STIs Department of Disease Control. The Asian Epidemic Model (AEM) Projections for HIV/AIDS in Thailand:2005-2025. Bangkok: Family Health International (FHI) and Bureau of AIDS, TB and STIs, Department of Disease Control, Ministry of Public Health, Thailand, 2008.

3. Kirby Institute. 2015 Annual Surveillance Report of HIV, viral hepatitis, STIs. Sydney, New South Wales. https://kirby.unsw.edu.au/surveillance/2015-annual-surveillance-report-hiv-viral-hepatitis-stis: Kirby Institute, UNSW Australia, 2015.

4. Kirby Institute. Australian NSP survey national data report 2015. Sydney, New South Wales: Kirby Institute, University of New South Wales, 2015.

5. Country reports for Global AIDS Response Progress Reporting [Internet]. UNAIDS. 2014.

6. UNAIDS. UNAIDS Country reports. Geneva: Joint United Nations Programme on HIV/AIDS. http://www.unaids.org/en/regionscountries/countries, 2015.

7. United States Center for Disease Control and Prevention. HIV/AIDS Statistics. Atlanta, Georgia: US CDC. http://www.cdc.gov/hiv/statistics/index.html, 2015.

8. Gouws E, White PJ, Stover J, Brown T. Short term estimates of adult HIV incidence by mode of transmission: Kenya and Thailand as examples. Sex Transm Infect. 2006;82 Suppl 3:iii51-5.

9. Mathers BM, Degenhardt L, Phillips B, Wiessing L, Hickman M, Strathdee SA, et al. Global epidemiology of injecting drug use and HIV among people who inject drugs: a systematic review. Lancet. 2008;372(9651):1733-45.

Intimate partner violence[2]

Flowchart

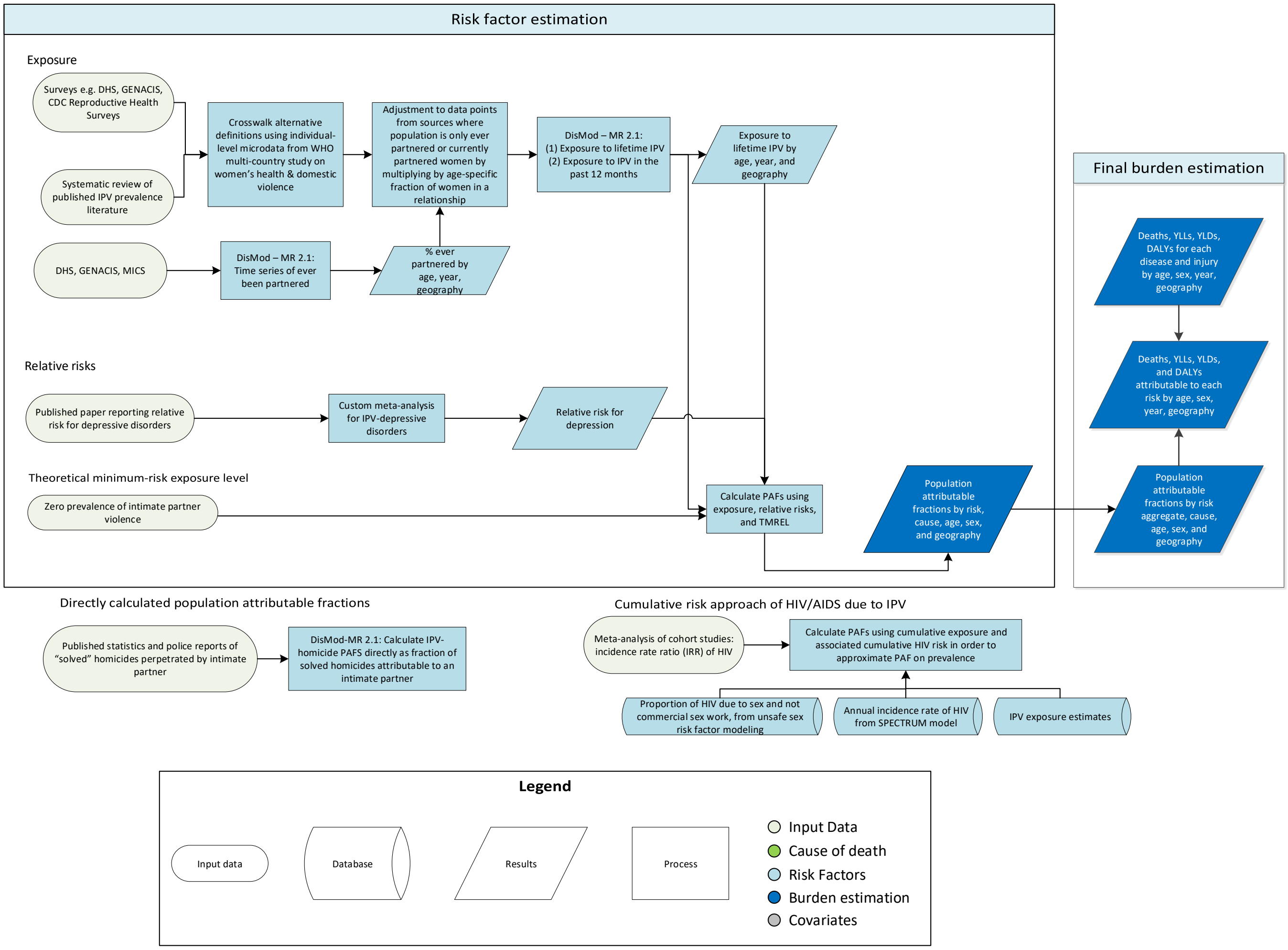

Input data and methodological summary

Definition

Exposure

The case definition for intimate partner violence (IPV) is ever experienced one or more acts of physical and/or sexual violence by a current or former intimate partner since the age of 15 years. Estimated in females only because evidence of risk-outcomes for males does not meet our criteria.

Physical violence is defined as “being slapped or having something thrown at you that could hurt you, being pushed or shoved, being hit with a fist or something else that could hurt, being kicked, dragged, or beaten up, being choked or burnt on purpose, and/or being threatened with or actually having a gun, knife, or other weapon used on you.”

Sexual violence is defined as “being physically forced to have intercourse when you did not want to, having sexual intercourse because you were afraid of what your partner might do, and/or being forced to do something that you found humiliating or degrading” (the definition of humiliating and degrading may vary across studies depending on the regional and cultural setting).

Intimate partner is defined as “a partner to whom you are married or with whom you cohabit.” In countries where people date, dating partners will also be considered (a partner with whom you have an intimate [sexual] relationship with but are not married to or cohabiting).

Theoretical Minimum Risk Exposure Level

The associated Theoretical Minimum Risk Exposure Level (TMREL) is zero exposure to IPV.

Input data

In addition to incorporating new sources shared with us by collaborators for both our IPV exposure model and our IPV direct PAF model (described in Modelling strategy), we conducted a systematic review of the fraction of homicides against women attributable to an intimate partner for GBD 2019 in Pubmed and EMBASE. We used the following search strings:

PubMed

((IPV[All Fields] OR ("intimate partner violence"[MeSH Terms] OR ("intimate"[All Fields] AND "partner"[All Fields] AND "violence"[All Fields]) OR "intimate partner violence"[All Fields])) AND (("homicide"[MeSH Terms] OR "homicide"[All Fields]) OR femicide[All Fields])) AND ("2013/01/01"[PDAT] : "3000/12/31"[PDAT])

Date searched: 12/25/2018

Number of hits: 155

EMBASE

((‘IPV’:ti,ab OR (‘partner violence’/syn OR (‘intimate’:ti,ab AND ‘partner’:ti,ab AND ‘violence’:ti,ab) OR ‘intimate partner violence’:ti,ab)) AND ((‘homicide’/syn OR ‘homicide’:ti,ab) OR ‘femicide’:ti,ab)) AND [2013-3000]/py

Date searched: 01/03/2019

Number of hits: 199

Number of hits duplicate with pubmed search: 109

Of these, 44 passed a title and abstract review, leading to nine sources passing a full text review and being extracted.

We included all sources that provided population-representative data on the proportion of women who have ever experienced physical or sexual violence by a current or former intimate partner. We also accepted sources reporting on the following non-reference populations:

Women who have ever experienced any physical IPV

Women who have ever experienced any sexual IPV

Women who have ever experienced severe IPV

Women who have experienced IPV in the past year

Women who have ever had an intimate partner who have experienced physical or sexual IPV

Women who currently have an intimate partner who have experienced IPV

Women who have experienced intimate partner violence by a spouse

Women who have experienced intimate partner violence by a current spouse

For the alternate definitions of IPV (just physical, just sexual, and severe), we ran a logit-difference meta-regression with the MR-BRT tool to estimate correction factors.

Table 1: Data inputs for exposure for intimate partner violence.

Input data	Exposure
Source count (total)	601
Number of countries with data	132

Table 2: Data inputs for relative risks for intimate partner violence.

Input data	Relative risk
Source count (total)	9
Number of countries with data	7

Table 3: MR-BRT adjustment factors for restricted definitions of intimate partner violence

Data input	Status	Gamma	Beta coefficient, logit (95% CI)	Adjustment factor*
Women who have ever experienced any physical or sexual IPV	Ref	0.21	---	---
Women who have ever experienced any physical IPV	Alt		-0.43 (-0.85 to -0.0042)	0.39
Women who have ever experienced any severe IPV	Alt		-0.44 (-0.86 to -0.02)	0.39
Women who have ever experienced any sexual IPV	Alt		-0.89 (-1.32 to -0.47)	0.29

**Adjustment factor is the inverse-logit transformed beta coefficient; <0.5 represents that alternative is adjusted upward; >0.5 represents alternative is adjusted downward.*

For sources using one-year instead of lifetime recall, we used a logit-difference regression with a cubic spline on age to estimate age-specific correction factors. We introduced these methods in lieu of a random effects meta regression, as was used in GBD 2017.

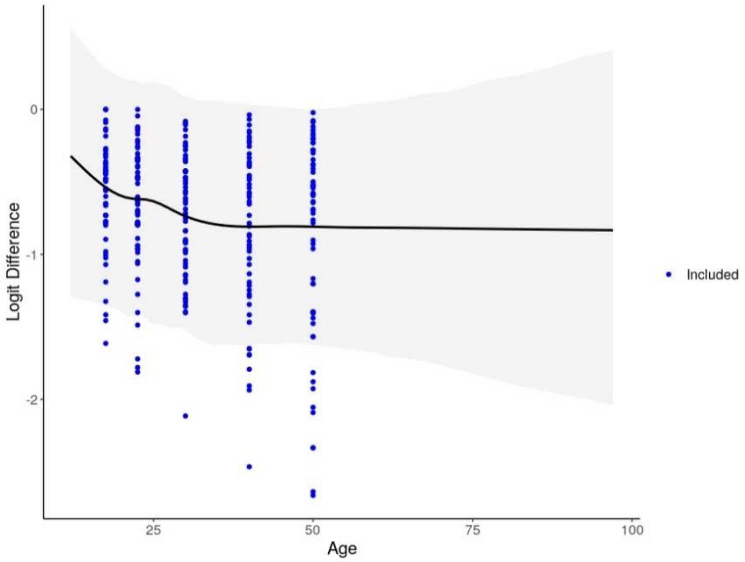

Graph 1: MR-BRT adjustment factors for intimate partner violence in the past year

To correct for studies reporting only on ever or currently partnered women, we multiplied estimates from these studies by the age-specific fraction of women who had ever been partnered. We generated ever-partnered estimates using MICS and DHS data in a single parameter DisMod model to reflect the most recent data on proportion of women who have ever been partnered.

For studies restricting the perpetrator to spouses or current spouses, due to insufficient data comparing our reference and alternate populations in specific age-location-years, we refrained from calculating under-informed correction factors.

Modelling strategy

We use three distinct approaches to estimate burden attributable to IPV, including 1) the traditional exposure and relative risk (RR) to percentage attributable fraction (PAF) method for depression; 2) the direct PAF approach for estimating the proportion of homicides that are perpetrated by an intimate partner; and 3) a cumulative risk approach for estimating the burden of HIV/AIDS attributable to IPV. Note that while we estimated the PAF of abortion attributable to IPV for GBD 2017, we stopped calculating this PAF for GBD 2019, due to insufficient evidence of a causal relationship between the risk and outcome.

Cumulative risk approach for PAF of HIV/AIDS due to IPV

The approach that we used to assess burden attributable to intimate partner violence was a cumulative risk approach to measure the burden of HIV/AIDS attributable to IPV.

The approach itself remained the same in GBD 2019 but included newly estimated relative risk, updated intimate partner violence exposure numbers from the DisMod-MR 2.1 model described above, as well as revised HIV incidence numbers.

As we measure burden based on deaths and prevalence, we needed to quantify attributable fractions for prevalence and death rather than incidence. To get a PAF for prevalence we needed to consider the history of exposure to IPV and the accumulated associated risk of incident HIV due to IPV, relative to the overall risk of HIV at the population level. The ratio of cumulative IPV-attributable HIV incidence to total HIV incidence was an approximation of the relevant PAF for HIV prevalence and we assumed this PAF can also be applied to mortality.

$$\frac{\text{Cumulative HIV incidence due to IPV}}{\text{Cumulative HIV incidence overall}} = \frac{1\text{-} \prod_{a=o}^{a=n}\left(1\text{-PAF}_{ay}{*}\text{I}_{ay}\right)}{1\text{-} \prod_{a=o}^{a=n}(1\text{-I}_{ay})}$$

where:

I = annual incidence rate of HIV

a = age (15-95)

y = year (1980-2016)

$$\text{PAF}_{\text{HIV incidence}} = \frac{[\text{Prevalence of IPV}]_{ay}{*}\ (\text{IRR-1})}{[\text{Prevalence of IPV}]_{ay}{*}\ (\text{IRR-1})+1}$$

Theoretical minimum-risk exposure level

The theoretical minimum-risk exposure level is zero exposure to intimate partner violence, as defined above.

Relative risk

HIV incidence

From two cohort studies (Jewkes et al, Lancet 2010 & Kouyoumdijian et al, AIDS 2013) we calculated the relative risk of HIV incidence as 1.60 (95% UI 1.31–1.93) using a regression with MR-BRT.

Citations

Ackard DM, Eisenberg ME, Neumark-Sztainer D. Long-term impact of adolescent dating violence on the behavioral and psychological health of male and female youth. J Pediatr. 2007; 151(5): 476-81.

Bourassa D, Bérubé J. The prevalence of intimate partner violence among women and teenagers seeking abortion compared with those continuing pregnancy. J Obstet Gynaecol Can. 2007; 29(5): 415-23.

Chowdhary N, Patel V. The effect of spousal violence on women's health: findings from the Stree Arogya Shodh in Goa, India. J Postgrad Med. 2008; 54(4): 306–12.

Han KM, Jee HJ, An H, Shin C, Yoon HK, Ko YH, Ham BJ, Kim YK, Han C. Intimate partner violence and incidence of depression in married women: A longitudinal study of a nationally representative sample. J Affect Disord. 2019; 245():305-311

Jewkes RK, Dunkle K, Nduna M, Shai N. Intimate partner violence, relationship power inequity, and incidence of HIV infection in young women in South Africa: a cohort study. Lancet. 2010; 41-48.

Kouyoumdjian FG, Calzavara LM, Bondy SJ, O'Campo P, Serwadda D, Nalugoda F, Kagaayi J, Kigozi G, Wawer M, Gray R. Intimate partner violence is associated with incident HIV infection in women in Uganda. AIDS. 2013; 27(8): 1331-8.

Leung TW, Leung WC, Chan PL, Ho PC. A comparison of the prevalence of domestic violence between patients seeking termination of pregnancy and other general gynecology patients. Int J Gynaecol Obstet. 2002; 77(1): 47-54.

Lipsky S, Caetano R, Roy-Byrne P. Racial and ethnic disparities in police-reported intimate partner violence and risk of hospitalization among women. Womens Health Issues. 2009; 19(2):109–118.

Loxton D, Schofield M, Hussain R. Psychological health in midlife among women who have ever lived with a violent partner or spouse. J Interpers Violence. 2006; 21(8): 1092-107.

Ouellet-Morin I, Fisher HL, York-Smith M, Fincham-Campbell S, Moffitt TE, Arseneault L. Intimate partner violence and new-onset depression: a longitudinal study of women's childhood and adult histories of abuse. Depression and anxiety. 2015;32(5):316-324.

Romito P, Escribà-Agüir V, Pomicino L, Lucchetta C, Scrimin F, Molzan Turan J. Violence in the lives of women in Italy who have an elective abortion. Womens Health Issues. 2009; 19(5): 335-43.

Suglia SF, Duarte CS, Sandel MT. Housing quality, housing instability, and maternal mental health. J Urban Health. 2011; 88(6): 1105–16.

Taft AJ, Watson LF. Termination of pregnancy: associations with partner violence and other factors in a national cohort of young Australian women. Aust N Z J Public Health. 2007; 31(2): 135-42.

Author Contributions

Providing data or critical feedback on data sources

Jorge R Ledesma, Hassan Abolhassani, Akine Eshete Abosetugn, Eman Abu-Gharbieh, Oladimeji M Adebayo, Qorinah Estiningtyas Sakilah Adnani, Saira Afzal, Bright Opoku Ahinkorah, Sajjad Ahmad Ahmad, Sepideh Ahmadi, Tarik Ahmed Rashid, Yusra Ahmed Salih, Addis Aklilu, Chisom Joyqueenet Akunna, Hanadi Al Hamad, Fares Alahdab, Yosef Alemayehu, Kefyalew Addis Alene, Beriwan Abdulqadir Ali, Liaqat Ali, Hesam Alizade, Nelson Alvis-Guzman, Saeed Amini, Carl Abelardo T Antonio, Jalal Arabloo, Mulusew A Asemahagn, Atif Amin Baig, Maciej Banach, Amadou Barrow, Sanjay Basu, Akshaya Srikanth Bhagavathula, Natalia V Bhattacharjee, Boris Bikbov, Archith Boloor, Sharath Burugina Nagaraja, Souranshu Chatterjee, Soosanna Kumary Chattu, Vijay Kumar Chattu, Dinh-Toi Chu, Lalit Dandona, Rakhi Dandona, Parnaz Daneshpajouhnejad, Aso Mohammad Darwesh, Mostafa Dianatinasab, Linh Phuong Doan, Emerito Jose A Faraon, Farshad Farzadfar, Irina Filip, Tushar Garg, Keyghobad Ghadiri, Ahmad Ghashghaee, Mahaveer Golechha, Bhawna Gupta, Veer Bala Gupta, Vivek Kumar Gupta, Asif Hanif, Shafiul Haque, Hadi Hassankhani, Kamal Hezam, Mehdi Hosseinzadeh, Mowafa Househ, Segun Emmanuel Ibitoye, Seyed Sina Naghibi Irvani, Nahlah Elkudssiah Ismail, Vardhmaan Jain, Sathish Kumar Jayapal, Shubha Jayaram, Jost B Jonas, Farahnaz Joukar, Zubair Kabir, Himal Kandel, Gbenga A Kayode, Parkes J Kendrick, Amene Abebe Kerbo, Himanshu Khajuria, Rovshan Khalilov, Khaled Khatab, Abdullah T Khoja, Min Seo Kim, Yun Jin Kim, Adnan Kisa, Sezer Kisa, Soewarta Kosen, Sindhura Lakshmi Koulmane Laxminarayana, Kewal Krishan, Burcu Kucuk Bicer, G Anil Kumar, Dharmesh Kumar Lal, Savita Lasrado, Shaun Wen Huey Lee, Wei-Chen Lee, Christine Lin, Xuefeng Liu, Shilpashree Madhava Kunjathur, Francisco Rogerlândio Martins-Melo, Walter Mendoza, Ritesh G Menezes, Nour Mheidly Mheidly, Prasanna Mithra, Masoud Moghadaszadeh, Mokhtar Mohammadi, Shafiu Mohammed, Mariam Molokhia, Ahamarshan Jayaraman Nagarajan, Sreenivas Narasimha Swamy, Javad Nazari, Sandhya Neupane Kandel, Trang Huyen Nguyen, Chukwudi A Nnaji, Bogdan Oancea, Andrew T Olagunju, Mahesh P A, Fatemeh Pashazadeh Kan, Shrikant Pawar, Veincent Christian Filipino Pepito, Maarten J Postma, Amir Radfar, Alireza Rafiei, Fakher Rahim, Vafa Rahimi-Movaghar, Mahfuzar Rahman, Amir Masoud Rahmani, Chythra R Rao, David Laith Rawaf, Salman Rawaf, Marissa B Reitsma, Andre M N Renzaho, Nima Rezaei, Siamak Sabour, KM Saif-Ur-Rahman, Nasir Salam, Abdallah M Samy, Satish Saroshe, Brijesh Sathian, Subramanian Senthilkumaran, Allen Seylani, Masood Ali Shaikh, Jae Il Shin, Jitendra Kumar Singh, Valentin Yurievich Skryabin, Anna Aleksandrovna Skryabina, Chandrashekhar T Sreeramareddy, Takahiro Tabuchi, Zemenu Tamir, Elvis Enowbeyang

Tarkang, Bach Xuan Tran, Berhan Tsegaye, Gebiyaw Wudie Tsegaye, Anayat Ullah, Bay Vo, Giang Thu Vu, Yasir Waheed, Siyan Yi, Naohiro Yonemoto, Chuanhua Yu, Mikhail Sergeevich Zastrozhin, Zhi-Jiang Zhang, Alimuddin Zumla, Ali H Mokdad, Stephen S Lim, Mohsen Naghavi, Theo Vos, and Hmwe Hmwe Kyu

Development of methods or computational machinery
Jorge R Ledesma, Dana Bryazka, Deepa Jahagirdar, Saira Afzal, Tarik Ahmed Rashid, Yusra Ahmed Salih, Hesam Alizade, Saeed Amini, Mulusew A Asemahagn, Austin Carter, Xiaochen Dai, Aso Mohammad Darwesh, Mostafa Dianatinasab, Farshad Farzadfar, Tahvi D Frank, Mehdi Hosseinzadeh, Mowafa Househ, Jalil Jaafari, Rovshan Khalilov, Adnan Kisa, Sezer Kisa, Masoud Moghadaszadeh, Mokhtar Mohammadi, Mohammad Ali Ali Moni, Javad Nazari, Amir Masoud Rahmani, Abdallah M Samy, Bay Vo, Magdalene K Walters, Mesfin Agachew Woldekidan, Ali H Mokdad, Robert C Reiner Jr, Mohsen Naghavi, Theo Vos, Christopher J L Murray, and Hmwe Hmwe Kyu

Providing critical feedback on methods or results
Jorge R Ledesma, Kate E LeGrand, Jennifer M Ross, Dana Bryazka, Hassan Abolhassani, Akine Eshete Abosetugn, Eman Abu-Gharbieh, Oladimeji M Adebayo, Qorinah Estiningtyas Sakilah Adnani, Saira Afzal, Bright Opoku Ahinkorah, Sajjad Ahmad Ahmad, Tarik Ahmed Rashid, Yusra Ahmed Salih, Addis Aklilu, Chisom Joyqueenet Akunna, Hanadi Al Hamad, Fares Alahdab, Yosef Alemayehu, Kefyalew Addis Alene, Liaqat Ali, Vahid Alipour, Hesam Alizade, Rajaa M Al-Raddadi, Nelson Alvis-Guzman, Saeed Amini, Arianna Maever L Amit, Sofia Androudi, Razique Anwer, Jalal Arabloo, Asrat Arja, Mulusew A Asemahagn, Sachin R Atre, Gulrez Shah Azhar, Darshan B B, Atif Amin Baig, Maciej Banach, Hiba Jawdat Barqawi, Fabio Barra, Amadou Barrow, Sanjay Basu, Uzma Iqbal Belgaumi, Akshaya Srikanth Bhagavathula, Nikha Bhardwaj, Pankaj Bhardwaj, Natalia V Bhattacharjee, Krittika Bhattacharyya, Ali Bijani, Boris Bikbov, Archith Boloor, Danilo Buonsenso, Sharath Burugina Nagaraja, Zahid A Butt, Jaykaran Charan, Souranshu Chatterjee, Soosanna Kumary Chattu, Vijay Kumar Chattu, Devasahayam J Christopher, Dinh-Toi Chu, Mareli M Claassens, Omid Dadras, Amare Belachew Dagnew, Xiaochen Dai, Lalit Dandona, Rakhi Dandona, Aso Mohammad Darwesh, Deepak Dhamnetiya, Mostafa Dianatinasab, Daniel Diaz, Linh Phuong Doan, Sahar Eftekharzadeh, Muhammed Elhadi, Emerito Jose A Faraon, Farshad Farzadfar, Lorenzo Ferro Desideri, Irina Filip, Florian Fischer, Masoud Foroutan, Tahvi D Frank, Alberto L Garcia-Basteiro, Christian Garcia-Calavaro, Tushar Garg, Biniyam Sahiledengle Geberemariyam, Mahaveer Golechha, Bhawna Gupta, Sapna Gupta, Veer Bala Gupta, Vivek Kumar Gupta, Mohammad Rifat Haider, Samer Hamidi, Asif Hanif, Shafiul Haque, Harapan Harapan, Arief Hargono, Ahmed I Hasaballah, Abdiwahab Hashi, Shoaib Hassan, Hadi Hassankhani, Khezar Hayat, Ramesh Holla, Mehdi Hosseinzadeh, Mihaela Hostiuc, Mowafa Househ, Rabia Hussain, Segun Emmanuel Ibitoye, Irena M Ilic, Milena D Ilic, Seyed Sina Naghibi Irvani, Nahlah Elkudssiah Ismail, Ramaiah Itumalla, Jalil Jaafari, Kathryn H Jacobsen, Vardhmaan Jain, Sathish Kumar Jayapal, Shubha Jayaram, Ravi Prakash Jha, Jost B

Jonas, Nitin Joseph, Farahnaz Joukar, Zubair Kabir, Ashwin Kamath, Tanuj Kanchan, Himal Kandel, Patrick DMC Katoto, Gbenga A Kayode, Amene Abebe Kerbo, Himanshu Khajuria, Rovshan Khalilov, Khaled Khatab, Jagdish Khubchandani, Min Seo Kim, Yun Jin Kim, Adnan Kisa, Sezer Kisa, Sindhura Lakshmi Koulmane Laxminarayana, Ai Koyanagi, Kewal Krishan, Avinash Kumar, G Anil Kumar, Narinder Kumar, Nithin Kumar, Alexander Kwarteng, Hassan Mehmood Lak, Dharmesh Kumar Lal, Savita Lasrado, Shaun Wen Huey Lee, Wei-Chen Lee, Xuefeng Liu, Daiane Borges Machado, Shilpashree Madhava Kunjathur, Deepak Madi, Preetam Bhalchandra Mahajan, Azeem Majeed, Ahmad Azam Malik, Francisco Rogerlândio Martins-Melo, Saurabh Mehta, Walter Mendoza, Ritesh G Menezes, Hayimro Edemealem Merie, Amanual Getnet Mersha, Mohamed Kamal Mesregah, Tomislav Mestrovic, Nour Mheidly Mheidly, Sanjeev Misra, Prasanna Mithra, Masoud Moghadaszadeh, Mokhtar Mohammadi, Abdollah Mohammadian-Hafshejani, Shafiu Mohammed, Mariam Molokhia, Mohammad Ali Ali Moni, Catrin E Moore, Ahamarshan Jayaraman Nagarajan, Sanjeev Nair, Suma Nair, Atta Abbas Naqvi, Sreenivas Narasimha Swamy, Biswa Prakash Nayak, Javad Nazari, Sandhya Neupane Kandel, Trang Huyen Nguyen, Molly R Nixon, Chukwudi A Nnaji, Bogdan Oancea, Andrew T Olagunju, Eyal Oren, Mahesh P A, Ramakrishnan Parthasarathi, Sanjay M Pattanshetty, Rajan Paudel, Pintu Paul, Shrikant Pawar, Veincent Christian Filipino Pepito, Majid Pirestani, Maarten J Postma, Akram Pourshams, Akila Prashant, Dimas Ria Angga Pribadi, Amir Radfar, Alireza Rafiei, Fakher Rahim, Vafa Rahimi-Movaghar, Mahfuzar Rahman, Mosiur Rahman, Amir Masoud Rahmani, Priyanga Ranasinghe Ranasinghe, Chythra R Rao, David Laith Rawaf, Salman Rawaf, Marissa B Reitsma, Andre M N Renzaho, Melese Abate Reta, Nima Rezaei, Omid Rezahosseini, Mohammad sadegh Rezai, Aziz Rezapour, Gholamreza Roshandel, Denis O Roshchin, Siamak Sabour, KM Saif-Ur-Rahman, Nasir Salam, Mehrnoosh Samaei, Abdallah M Samy, Satish Saroshe, Benn Sartorius, Brijesh Sathian, Susan M Sawyer, Allen Seylani, Omid Shafaat, Masood Ali Shaikh, Kiomars Sharafi, Ranjitha S Shetty, Mika Shigematsu, Jae Il Shin, João Pedro Silva, Jitendra Kumar Singh, Smriti Sinha, Valentin Yurievich Skryabin, Anna Aleksandrovna Skryabina, Chandrashekhar T Sreeramareddy, Paschalis Steiropoulos, Mu'awiyyah Babale Sufiyan, Eyayou Girma Tadesse, Zemenu Tamir, Elvis Enowbeyang Tarkang, Yohannes Tekalegn, Belay Tessema, Rekha Thapar, Imad I Tleyjeh, Ruoyan Tobe-Gai, Bach Xuan Tran, Berhan Tsegaye, Gebiyaw Wudie Tsegaye, Anayat Ullah, Chukwuma David Umeokonkwo, Bay Vo, Giang Thu Vu, Yasir Waheed, Mesfin Agachew Woldekidan, Befikadu Legesse Wubishet, Taklo Simeneh Yazie Yazie, Yigizie Yeshaw, Siyan Yi, Naohiro Yonemoto, Chuanhua Yu, Ismaeel Yunusa, Mikhail Sergeevich Zastrozhin, Anasthasia Zastrozhina, Zhi-Jiang Zhang, Alimuddin Zumla, Ali H Mokdad, Joshua A Salomon, Stephen S Lim, Mohsen Naghavi, Theo Vos, Simon I Hay, Christopher J L Murray, and Hmwe Hmwe Kyu

Drafting the manuscript or revising it critically for important intellectual content
Jorge R Ledesma, Amanda Novotney, Kate E LeGrand, Jennifer M Ross, Hassan Abolhassani, Akine Eshete Abosetugn, Eman Abu-Gharbieh, Oladimeji M Adebayo, Saira Afzal, Bright Opoku Ahinkorah, Sajjad Ahmad Ahmad, Sepideh Ahmadi, Fares Alahdab, Yosef Alemayehu, Kefyalew

Addis Alene, Liaqat Ali, Hesam Alizade, Nelson Alvis-Guzman, Sofia Androudi, Carl Abelardo T Antonio, Catherine M Antony, Jalal Arabloo, Mulusew A Asemahagn, Gulrez Shah Azhar, Darshan B B, Zaheer-Ud-Din Babar, Atif Amin Baig, Maciej Banach, Hiba Jawdat Barqawi, Fabio Barra, Amadou Barrow, Sanjay Basu, Krittika Bhattacharyya, Boris Bikbov, Nikolay Ivanovich Briko, Danilo Buonsenso, Sharath Burugina Nagaraja, Felix Carvalho, Souranshu Chatterjee, Soosanna Kumary Chattu, Vijay Kumar Chattu, Devasahayam J Christopher, Dinh-Toi Chu, Mareli M Claassens, Deepak Dhamnetiya, Mostafa Dianatinasab, Daniel Diaz, Linh Phuong Doan, Sahar Eftekharzadeh, Muhammed Elhadi, Shymaa Enany, Emerito Jose A Faraon, Eduarda Fernandes, Lorenzo Ferro Desideri, Irina Filip, Masoud Foroutan, Alberto L Garcia-Basteiro, Christian Garcia-Calavaro, Tushar Garg, Biniyam Sahiledengle Geberemariyam, Bhawna Gupta, Sapna Gupta, Veer Bala Gupta, Vivek Kumar Gupta, Shafiul Haque, Harapan Harapan, Ahmed I Hasaballah, Abdiwahab Hashi, Shoaib Hassan, Kamal Hezam, Ramesh Holla, Mowafa Househ, Segun Emmanuel Ibitoye, Irena M Ilic, Milena D Ilic, Seyed Sina Naghibi Irvani, Nahlah Elkudssiah Ismail, Ramaiah Itumalla, Kathryn H Jacobsen, Vardhmaan Jain, Shubha Jayaram, Ravi Prakash Jha, Jost B Jonas, Nitin Joseph, Himal Kandel, Patrick DMC Katoto, Gbenga A Kayode, Amene Abebe Kerbo, Himanshu Khajuria, Rovshan Khalilov, Khaled Khatab, Abdullah T Khoja, Jagdish Khubchandani, Yun Jin Kim, Adnan Kisa, Sezer Kisa, Parvaiz A Koul, Sindhura Lakshmi Koulmane Laxminarayana, Ai Koyanagi, Kewal Krishan, Avinash Kumar, Narinder Kumar, Alexander Kwarteng, Hassan Mehmood Lak, Iván Landires, Savita Lasrado, Platon D Lopukhov, Daiane Borges Machado, Shilpashree Madhava Kunjathur, Preetam Bhalchandra Mahajan, Azeem Majeed, Ahmad Azam Malik, Francisco Rogerlândio Martins-Melo, Ziad A Memish, Walter Mendoza, Ritesh G Menezes, Mohamed Kamal Mesregah, Tomislav Mestrovic, Nour Mheidly Mheidly, Masoud Moghadaszadeh, Abdollah Mohammadian-Hafshejani, Shafiu Mohammed, Mariam Molokhia, Mohammad Ali Ali Moni, Ahmed Al Montasir, Catrin E Moore, Ahamarshan Jayaraman Nagarajan, Sanjeev Nair, Sreenivas Narasimha Swamy, Biswa Prakash Nayak, Sandhya Neupane Kandel, Trang Huyen Nguyen, Molly R Nixon, Mpiko Ntsekhe, Virginia Nuñez-Samudio, Oluwakemi Ololade Odukoya, Andrew T Olagunju, Eyal Oren, Mahesh P A, Pintu Paul, Shrikant Pawar, Veincent Christian Filipino Pepito, Norberto Perico, Roman V Polibin, Maarten J Postma, Amir Radfar, Fakher Rahim, Vafa Rahimi-Movaghar, Mahfuzar Rahman, Priyanga Ranasinghe Ranasinghe, Chythra R Rao, David Laith Rawaf, Salman Rawaf, Giuseppe Remuzzi, Andre M N Renzaho, Nima Rezaei, Gholamreza Roshandel, Denis O Roshchin, Siamak Sabour, KM Saif-Ur-Rahman, Nasir Salam, Hossein Samadi Kafil, Mehrnoosh Samaei, Abdallah M Samy, Susan M Sawyer, Allen Seylani, Mika Shigematsu, João Pedro Silva, Jitendra Kumar Singh, Smriti Sinha, Valentin Yurievich Skryabin, Anna Aleksandrovna Skryabina, Chandrashekhar T Sreeramareddy, Paschalis Steiropoulos, Mu'awiyyah Babale Sufiyan, Takahiro Tabuchi, Eyayou Girma Tadesse, Fisaha Haile Tesfay, Imad I Tleyjeh, Bach Xuan Tran, Anayat Ullah, Sahel Valadan Tahbaz, Giang Thu Vu, Yasir Waheed, Seyed Hossein Yahyazadeh Jabbari, Yigizie Yeshaw, Vahit Yiğit, Naohiro Yonemoto, Mikhail Sergeevich Zastrozhin, Anasthasia Zastrozhina, Alimuddin Zumla, Ali H Mokdad, Joshua A Salomon, Mohsen Naghavi, Theo Vos, Simon I Hay, Christopher J L Murray, and Hmwe Hmwe Kyu

Extracting, cleaning, or cataloging data; designing or coding figures and tables
Jorge R Ledesma, Jianing Ma, Avina Vongpradith, Emilie R Maddison, Kate E LeGrand, Rachel Feldman, Bright Opoku Ahinkorah, Hesam Alizade, Jason A Anderson, Tushar Garg, Ahmed I Hasaballah, Sathish Kumar Jayapal, Rovshan Khalilov, Masoud Moghadaszadeh, Shrikant Pawar, Abdallah M Samy, Emma Elizabeth Spurlock, Joanna L Whisnant, Vahit Yiğit, Mikhail Sergeevich Zastrozhin, Anasthasia Zastrozhina, Ali H Mokdad, and Mohsen Naghavi

Management of the overall research enterprise
Jorge R Ledesma, Amanda Novotney, Saira Afzal, Liaqat Ali, Hesam Alizade, Saeed Amini, Lalit Dandona, Amir Emami, Fatemeh Javanmardi, Rovshan Khalilov, Rafael Lozano, Masoud Moghadaszadeh, Javad Nazari, Molly R Nixon, Mahesh P A, Pintu Paul, Jitendra Kumar Singh, Ali H Mokdad, Mohsen Naghavi, Simon I Hay, Christopher J L Murray, and Hmwe Hmwe Kyu
